# Sterics and Hydrogen Bonding Control Stereochemistry and Self-Sorting in BINOL-Based Assemblies

**DOI:** 10.1021/jacs.1c05172

**Published:** 2021-06-14

**Authors:** You-Quan Zou, Dawei Zhang, Tanya K. Ronson, Andrew Tarzia, Zifei Lu, Kim E. Jelfs, Jonathan R. Nitschke

**Affiliations:** § Department of Chemistry, University of Cambridge, Cambridge CB2 1EW, United Kingdom; ‡ Department of Chemistry, Molecular Sciences Research Hub, White City Campus, Imperial College London, London W12 0BZ, United Kingdom

## Abstract

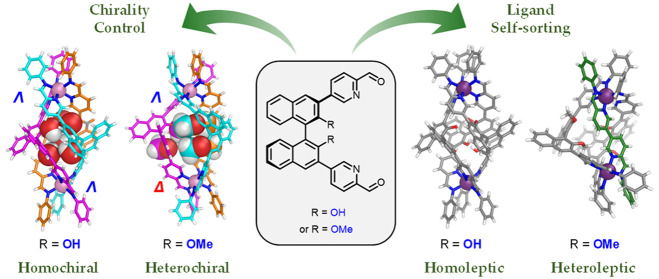

Here we demonstrate how the hydrogen-bonding ability of a BINOL-based dialdehyde subcomponent dictated the stereochemical outcome of its subsequent self-assembly into one diastereomeric helicate form when bearing free hydroxy groups, and another in the case of its methylated congener. The presence of methyl groups also altered the self-sorting behavior when mixed with another, short linear dialdehyde subcomponent, switching the outcome of the system from narcissistic to integrative self-sorting. In all cases, the axial chirality of the BINOL building block also dictated helicate metal center handedness during stereospecific self-assembly. A new family of stereochemically pure heteroleptic helicates were thus prepared using the new knowledge gained. We also found that switching from Fe^II^ to Zn^II^, or the incorporation of a longer linear ligand, favored heteroleptic structure formation.

Coordination-driven self-assembly enables the rapid and efficient construction of intricate and functional architectures,^1,2^ which have found an increasing number of uses.^3−5^ Studies of the propagation of stereochemical information within supramolecular assemblies^6^ have not only helped to elucidate possible origins of biological homochirality, but also opened new opportunities for chiral sensing and asymmetric transformation.^7^ Enantiopure ligands have been used successfully to control the chirality of assemblies, whereby fixed ligand stereocenters dictate the stereochemical configurations of metal centers, and thus the overall chirality of an assembly.^8^ A deeper understanding of the interplay between stereochemical elements during complex self-assembly processes allows the construction of increasingly complex structures with greater control over their stereochemistry.

Heteroleptic assemblies may result when ligands are designed to specifically match with other ligands within a multicomponent mixture.^9^ Just as biological systems exhibit this phenomenon, self-sorting is prevalent in supramolecular chemistry.^10,11^ Two self-sorting modes, narcissistic self-sorting^12^ and integrative self-sorting,^13^ are used to describe the extreme cases, which respectively give rise to homoleptic and heteroleptic architectures.^14^ Designing systems to undergo integrative self-sorting remains a major challenge, and switching self-sorting behavior between the two modes in a controllable manner is of great interest.

In this work, we demonstrate how the methylation of a subcomponent built around the 1,1′-bi-2-naphthol (BINOL)^15^ moiety alters the stereochemical outcome of subcomponent self-assembly (Figure 1).^16,17^ Methylation also changed the course of self-assembly within a system containing both a BINOL-based subcomponent and a linear bis(formyl)pyridine from narcissistic to integrative self-sorting. Integrative self-sorting within this system enabled the stereoselective construction of a new family of enantiopure heteroleptic helicates.

**Figure 1:**
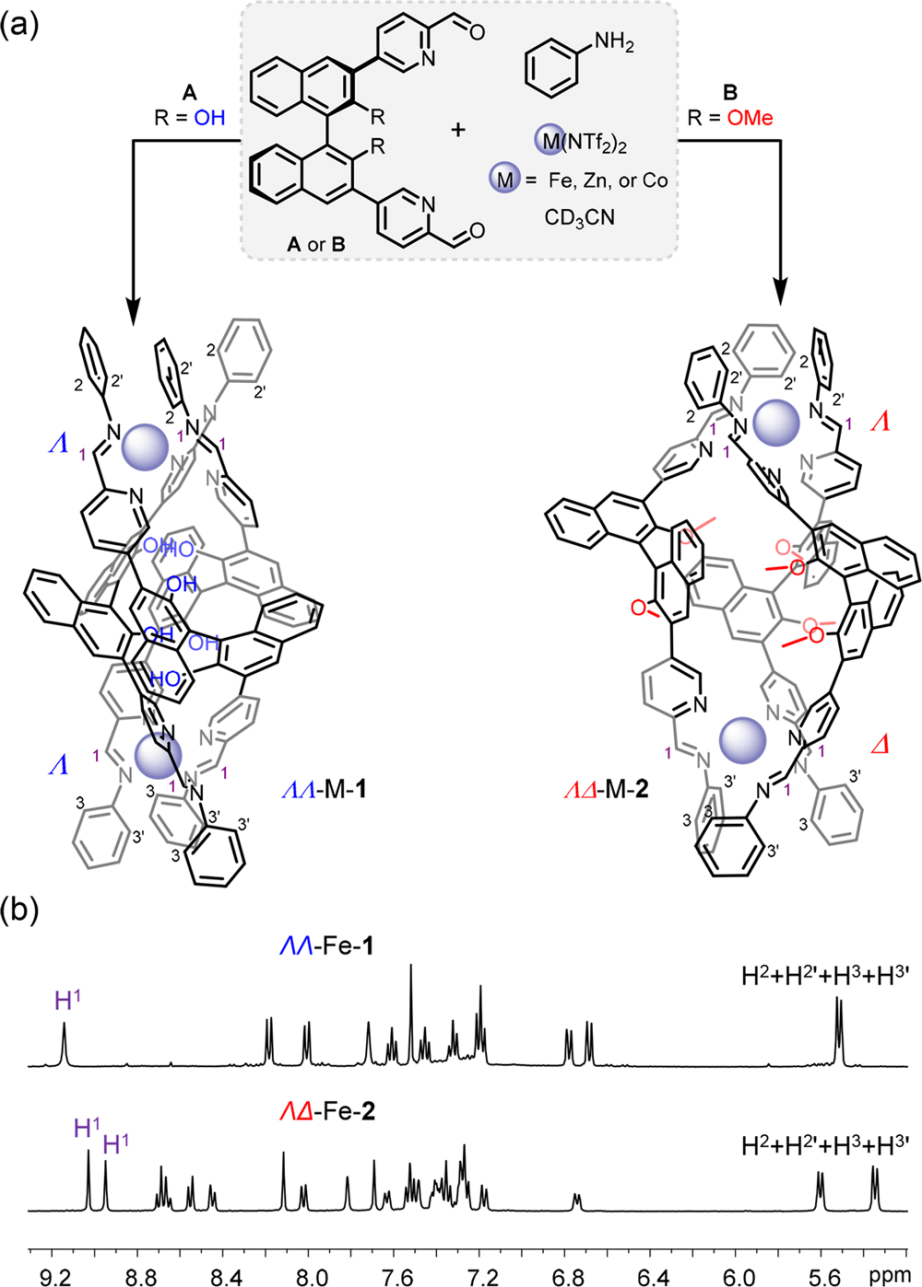
(a) Subcomponent self-assembly of **A** (R = OH) and **B** (R = OMe) with aniline and Co^II^, Zn^II^, or Fe^II^ afforded enantiopure helicates ΛΛ-M-**1** and ΛΔ-M-**2**, respectively. (b) ^1^H NMR spectra of ΛΛ-Fe-**1** and ΛΔ-Fe-**2**. The imine protons (H^1^) and *ortho*-protons of the aniline moieties (H^2^, H^2′^, H^3^, H^3′^) are indicated by purple and black labels, respectively.

Axially chiral subcomponents **A** (R = OH) and **B** (R = OMe) (Figure 1) were prepared in enantiopure form from commercially available (*R*)-BINOL as described in the Supporting Information (SI), sections 2.1 and 2.2. The reaction of **A** or **B** (3 equiv) with iron(II) bis(trifluoromethanesulfonyl)imide (Fe(NTf_2_)_2_, 2 equiv) and aniline (6 equiv) in acetonitrile at 70 °C produced enantiopure helicates Fe-**1** and Fe-**2**, respectively (Figure 1a). The ^1^H NMR spectrum of Fe-**1** displayed only one set of ligand signals (Figures 1b and S16), consistent with the formation of a helicate with *D*_3_ symmetry, where both iron(II) centers adopted the same Λ or Δ handedness.

In contrast, the ^1^H NMR spectrum of Fe-**2**, prepared from methylated **B**, exhibited two distinct ligand environments, as reflected in the presence of two imine peaks (Figures 1b and S68). This spectrum is consistent with the formation of an Fe_2_L_3_ helicate with overall *C*_3_ symmetry, having metal vertices of opposite handedness. Analogous assemblies formed from subcomponents **A** and **B** when Zn(NTf_2_)_2_ or Co(NTf_2_)_2_ were used in place of the iron(II) salt, resulting in helicates Zn-**1**, Zn-**2**, Co-**1**, and Co-**2** with ^1^H NMR spectral features similar to those of Fe-**1** and Fe-**2** (Figures S9, S23, S59, and S77).

Following slow diffusion of diethyl ether into solutions of Zn-**1**, Co-**1**, and Co-**2** in acetonitrile, crystals suitable for X-ray diffraction were obtained. As shown in Figure 2a,b, both metal centers in Zn-**1** and Co-**1** adopted the same Λ handedness. However, opposite configurations (Λ and Δ) were observed for the two metal centers of helicate Co-**2** (Figure 2c). These solid-state observations are consistent with the results obtained from the solution NMR spectra described above.

**Figure 2:**
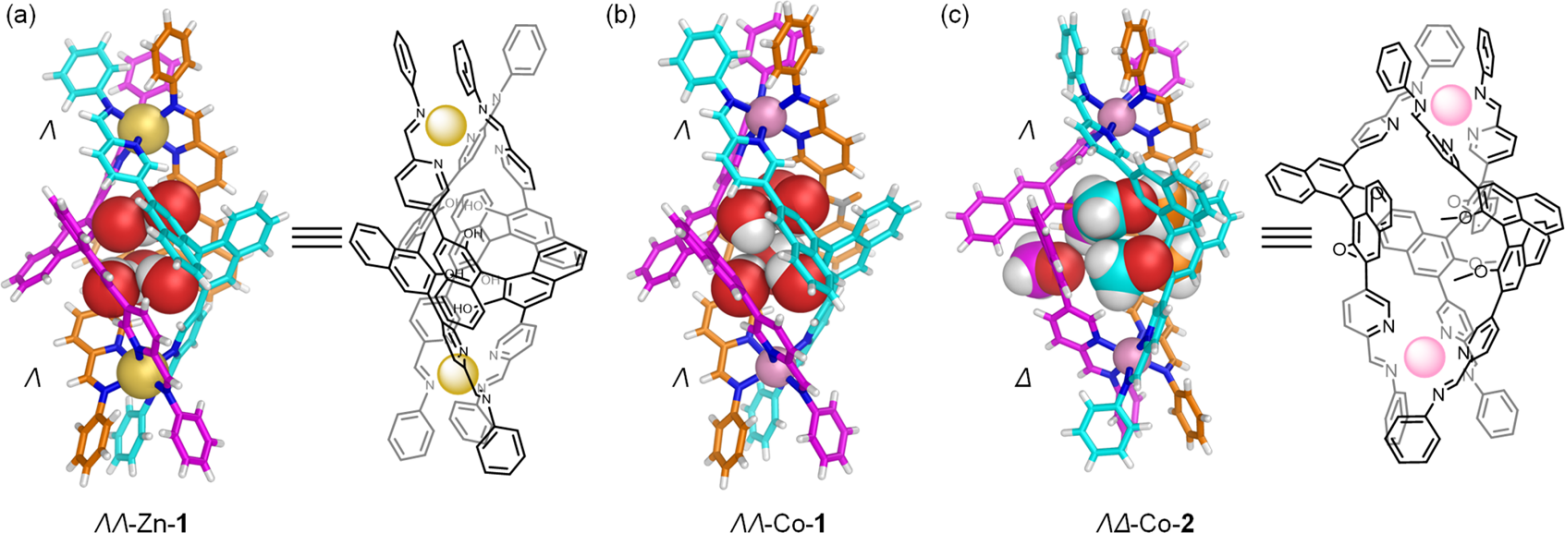
Crystal structures of (a) ΛΛ-Zn-**1**, (b) ΛΛ-Co-**1**, and (c) ΛΔ-Co-**2**. Hydroxy and methoxy groups are shown in space-filling mode, N is blue, H is white, and the carbon atoms of each independent ligand are drawn in fuchsia, cyan, and orange. Counteranions, disorder, and solvent of crystallization are omitted for clarity. [X-ray data for ΛΛ-Zn-**1**, ΛΛ-Co-**1**, and ΛΔ-Co-**2** are deposited as CCDC 2060204, 2060201, and 2060202, respectively.]

The crystal structures help to explain how the presence or absence of methyl groups directed the outcome of self-assembly. Comparison of the structures of ΛΛ-Co-**1** and ΛΔ-Co-**2** (Figure 2b, 2c) reveals structure **1** to be more enclosed than its methylated congener, with aromatic stacking interactions between neighboring ligands (3.6–4.0 Å). Each of the three equatorial −OH groups form intramolecular hydrogen bonds (O···O distance 2.9–3.1 Å), stabilizing the configuration of the helicate. The bulkier methoxy groups in **B** force the helicates to adopt a *pseudo*-meso conformation. This configuration has a more open center to accommodate its six methoxy groups (Figure 2c). A comparison of the relative density functional theory (DFT)-calculated energies of the ΛΛ and ΛΔ structures for the Zn-**1** and Zn-**2** assemblies further supports these findings (SI, section 6). The ΛΛ assembly is more stable by 8.7 kJ mol^–1^ for Zn-**1**, while the ΛΔ assembly is more stable by 20.4 kJ mol^–1^ for Zn-**2**.

The difference in methylation between **A** and **B** led to distinct self-sorting behavior in the presence of another linear subcomponent and metal salts. When **B** (2 equiv) was mixed with 6,6′-diformyl-3,3′-bipyridine **C** (1 equiv), Fe(NTf_2_)_2_ (2 equiv), and aniline (6 equiv) in acetonitrile, heteroleptic helicate Fe-**4** was formed cleanly (Figure 3a, bottom). Fe-**4** contains two residues of **B** and one of **C**, as indicated by its ^1^H NMR (Figure S80) and ESI-MS spectra (Figure S88), indicating that a thermodynamically favored integrative self-sorting process occurred. Trace amounts of homoleptic Fe-**2** were observed initially during self-assembly, which converted to Fe-**4** upon heating (Figure S80).

The structure of Fe-**4** was unambiguously confirmed by X-ray diffraction. As shown in Figure 3b, the two iron(II) centers are bridged by two residues of **B** and one of **C**, each condensed into a diimine ligand with two anilines. Both Fe^II^ centers adopt the same Λ handedness, generating a structure with *C*_2_ symmetry. The metal centers are separated by 9.7280(8) Å, significantly shorter than the Co···Co distance of 13.351(1) Å in homoleptic Co-**2** but only slightly greater than the Fe···Fe distance of ca. 9.5 Å previously observed for Fe-**3**, which assembled from subcomponent **C** with aniline and Fe^II^.^18^

**Figure 3:**
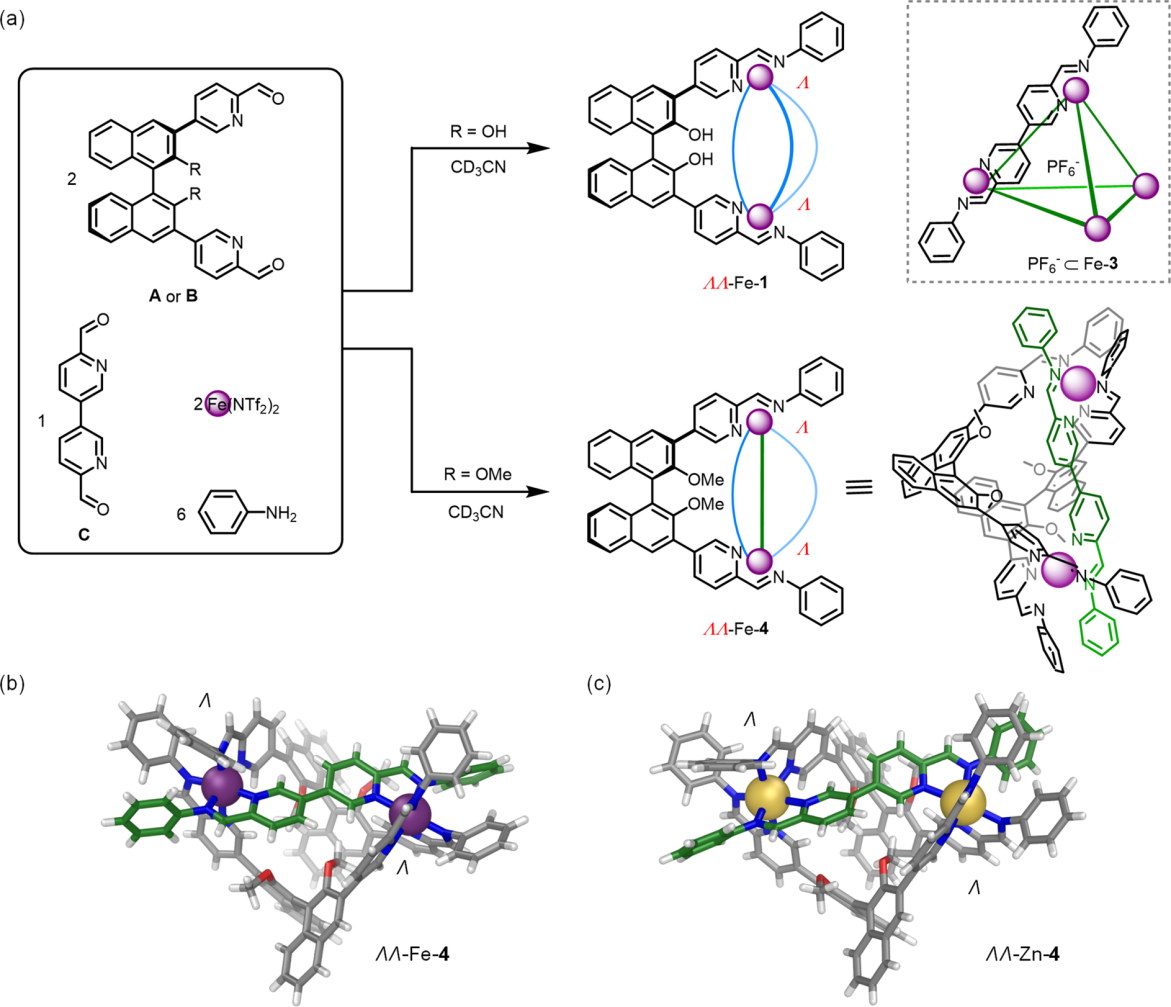
(a) Substituent effects on ligand self-sorting behavior. Bent **A** underwent narcissistic self-sorting with linear **C** (top), resulting in the simultaneous formation of Fe-**1** and Fe-**3**. Methylated **B**, in contrast, underwent integrative self-sorting with **C** (bottom). Crystal structures of (b) ΛΛ-Fe-**4** and (c) ΛΛ-Zn-**4**. Counteranions, disorder, and solvent molecules are omitted for clarity. [X-ray data for ΛΛ-Fe-**4** and ΛΛ-Zn-**4** are deposited as CCDC 2060203 and 2060205, respectively.]

Fe-**4** is the first member of a new class of heteroleptic helicates, consisting of two bent ligands and one linear ligand. We infer that the avoidance of steric eclipsing between the methoxy groups in homoleptic **2** (Figure 2c) drives the integrative self-sorting process, leading to **4**.

Narcissistic self-sorting was observed for the mixture of **A**, **C**, iron(II) triflimide, and aniline (Figure 3a, top), in contrast to the situation involving **B** and **C** described above. The products of this reaction were homoleptic helicate ΛΛ-Fe-**1** and tetrahedron Fe-**3** (SI, section 3.2.1).^18,19^

Intriguingly, ^1^H NMR peaks corresponding to heteroleptic Fe-**5**, an analog of Fe-**4** that incorporated two **A** residues and one **C**, were also observed after heating at 70 °C for 1 h, but these gradually disappeared in favor of peaks corresponding to homoleptic Fe-**1** (Figure S24). We were able to obtain metastable Fe-**5** in pure form by conducting the reaction at room temperature (SI, section 3.2.1). Its CD spectrum indicated ΛΛ stereochemistry of the two Fe^II^ centers (Figure S33), as with heteroleptic Fe-**4**.

We hypothesize that a driving force for the thermodynamically favored narcissistic self-sorting synthesis of Fe-**1** is the hydrogen-bonding interactions among the six −OH groups in ΛΛ-Fe-**1**, which stabilize the homoleptic structure.

Self-sorting outcomes were also found to be strongly affected by the identity of the metal salt and the length of the linear ligand. When Zn^II^ was used in place of Fe^II^, both **A** and **B** underwent integrative self-sorting, giving rise to heteroleptic helicates as the thermodynamically favored products (SI, sections 3.2.2 and 4.2.2). The structure of ΛΛ-Zn-**4**, prepared from **B** and **C**, was further confirmed by X-ray crystallography (Figure 3c).

The integrative self-sorting behavior displayed by Zn^II^ may be driven by the inability of Zn^II^ and **C** to form a stable homoleptic structure in the absence of anion templates.^20^ The more flexible coordination sphere of the larger Zn^II^ centers^21^ may be better able to disperse the strain arising from differences in the preferred metal–metal distances of the two ligands, compared to the more rigid Fe^II^, compensating for the stabilizing hydrogen-bonding interactions in homoleptic ΛΛ-Zn-**1** (Figure 2a).

When longer linear subcomponent **D** (Figure 4a) was used instead of **C** together with either **A** or **B**, Fe(NTf_2_)_2_, and aniline in acetonitrile, the heteroleptic helicates Fe-**6** and Fe-**7** (SI, sections 3.2.3 and 4.2.3) were formed via integrative self-sorting, along with the generation of the previously reported^22^ homoleptic Fe_4_L^**D**^_6_ assembly from **D** as a minor product (Figures 4a, S46, and S99). CD spectra of Fe-**6** and Fe-**7** were consistent with Δ handedness of the metal vertices for both complexes (Figures S55 and S108). Structures of the ΔΔ and ΛΛ assemblies of Fe-**6** and Fe-**7** were modeled using DFT (SI, section 6 describes the DFT methodology; Figures 4b,c and S121), providing Fe^II^···Fe^II^ distances of 12.4 Å (Fe-**6**) and 12.3 Å (Fe-**7**), respectively, significantly longer than the metal–metal distance in Fe-**4** (9.7280(8) Å, Figure 3b). Similar results were obtained with Zn(NTf_2_)_2_ (SI, sections 3.2.4 and 4.2.4).

**Figure 4:**
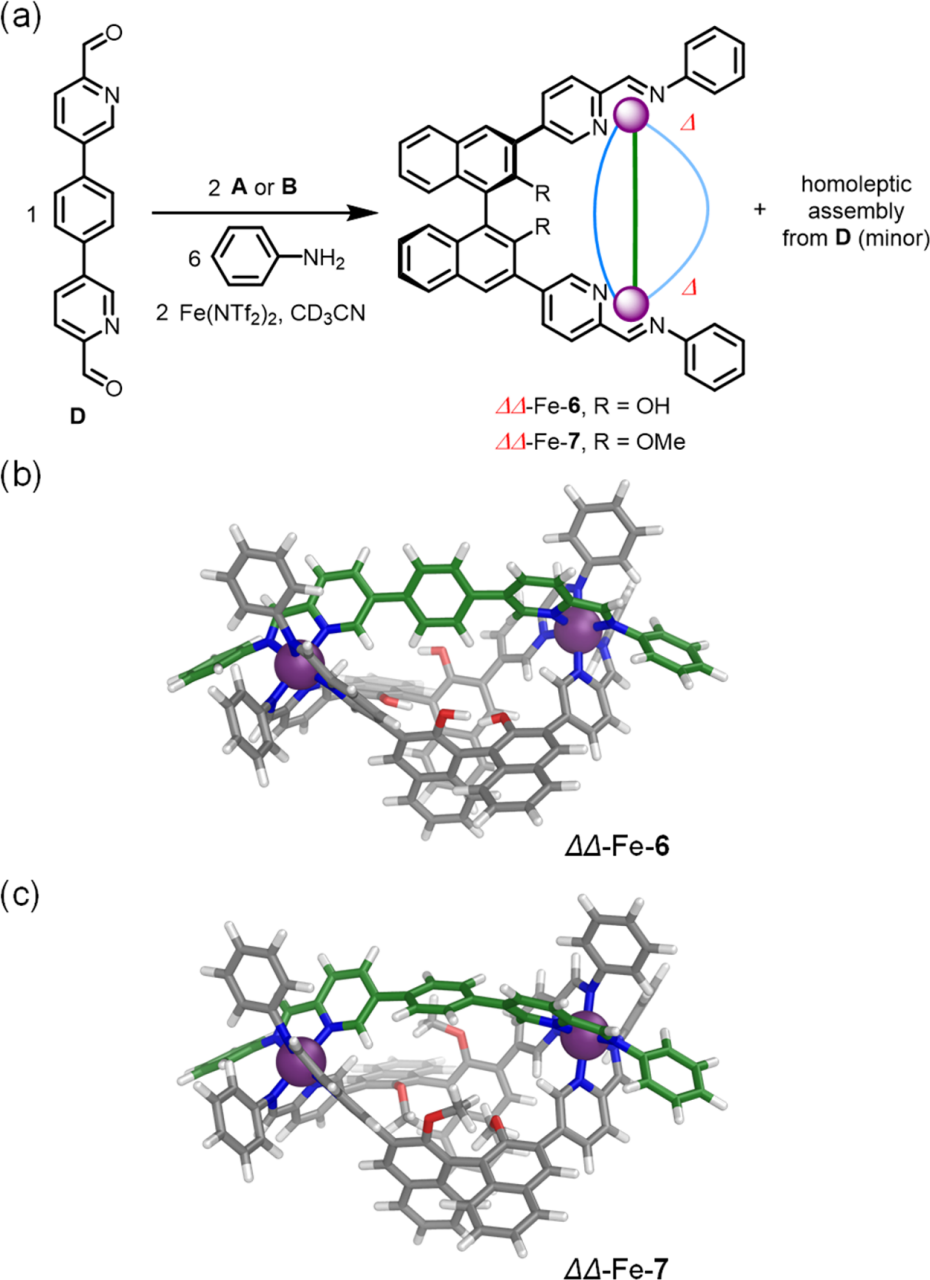
(a) Subcomponent self-assembly of **A** or **B** with longer **D**, aniline, and Fe(NTf_2_)_2_ generated the heteroleptic helicates ΔΔ-Fe-**6** and ΔΔ-Fe-**7**. DFT (PBE0/def2-SVP/D4)-optimized molecular models of (b) ΔΔ-Fe-**6** and (c) ΔΔ-Fe-**7**.

We hypothesize that the longer length of **D** as opposed to **C** brought the methoxy groups of **B** out of steric clash with each other as **B** scissored apart during heteroleptic self-assembly. This expansion of **B** thus enabled it to match the longer preferred distance between binding sites of **D**, whereas these methoxy groups eclipsed each other during heteroleptic assembly with shorter **C**. Relative DFT energies show that the ΛΛ assembly is more stable by 47.6 kJ mol^–1^ for Fe-**4**, while the ΔΔ structures are more stable by 63.8 and 16.3 kJ mol^–1^ for Fe-**6** and Fe-**7**, respectively (see SI, section 6 for details).

The self-assembly rules uncovered here provide new means of directing the formation of heteroleptic assemblies with controlled chirality. The new assemblies reported herein expose functionality—methoxy or hydroxyl groups—to their chirotopic interiors.^22^ The inward orientation of such groups around a well-defined volume has been demonstrated to achieve reactivity modulation. Structures such as **1** and **2** may also be seen as a new class of enantiopure supramolecular receptor, which may be capable of binding cations tightly and selectively if their central cavities are shielded from the cations that knit the structures together.

## Supporting Information Available

Experimental and spectroscopic details, including Figures S1–S121 and Tables S1–S3 (PDF)

## Accession Codes

CCDC 2060201–2060205 contain the supplementary crystallographic data for this paper. These data can be obtained free of charge via www.ccdc.cam.ac.uk/data_request/cif, or by emailing data_request@ccdc.cam.ac.uk, or by contacting The Cambridge Crystallographic Data Centre, 12 Union Road, Cambridge CB2 1EZ, UK; fax: +44 1223 336033.
