# Supplementary material for: Sterics and Hydrogen Bonding Control Stereochemistry and Self-Sorting in BINOL-Based Assemblies
Source: J Am Chem Soc. 2021 Jun 14;143(24):9009–15. doi: 10.1021/jacs.1c05172 (PMC8227477; doi:10.1021/jacs.1c05172)
Supplement: Supplementary file 1 — ja1c05172_si_001.pdf [file ja1c05172_si_001.pdf]

Supporting Information for

Sterics and Hydrogen Bonding Control Stereochemistry and Self-Sorting in BINOL-based Assemblies

You-Quan Zou+, Dawei Zhang+, Tanya K. Ronson, Andrew Tarzia, Zifei Lu, Kim E. Jelfs* and Jonathan R. Nitschke*

+These authors contributed equally to this work.

1. General information ...3

2. Synthesis and characterization of subcomponents ...4

2.1 Synthesis of subcomponent **B** ...4
2.2 Synthesis of subcomponent **A** ...8
2.3 Synthesis of subcomponent **D** ...10

3. Self-assembly using subcomponent **A** ...13

3.1 Sole subcomponent **A** ...13
3.1.1 with Zn(II) salt ...13
3.1.2 with Fe(II) salt ...18
3.1.3 with Co(II) salt ...23
3.2 Mixed subcomponents ...25
3.2.1 using subcomponents **A** and **C** with Fe(II) salt ...25
3.2.2 using subcomponents **A** and **C** with Zn(II) salt ...33
3.2.3 using subcomponents **A** and **D** with Fe(II) salt ...39
3.2.4 using subcomponents **A** and **D** with Zn(II) salt ...46

4. Self-assembly using subcomponent **B** ...48

4.1 Sole subcomponent **B** ...48
4.1.1 with Zn(II) salt ...48
4.1.2 with Fe(II) salt ...54
4.1.3 with Co(II) salt ...60
4.2 Mixed subcomponents ...63
4.2.1 using subcomponents **B** and **C** with Fe(II) salt ...63
4.2.2 using subcomponents **B** and **C** with Zn(II) salt ...70
4.2.3 using subcomponents **B** and **D** with Fe(II) salt ...75
4.2.4 using subcomponents **B** and **D** with Zn(II) salt ...82

5 X-ray crystallography ...88

6 Density functional theory calculations ...94

6.1 General 94
6.2 Structure generation and optimisation.. 94
6.3 Strain in heteroleptic helicates 96

7. References 98

1. General Information

All experiments were carried out under inert atmosphere of purified nitrogen or using standard Schlenk techniques. Reactions were stirred using Teflon-coated magnetic stir bars. Elevated temperatures were maintained using Thermostat-controlled silicone oil baths. Organic solutions were concentrated using a rotary evaporator with a diaphragm vacuum pump. Analytical TLC was performed on Merck silica gel 60 F_{254} plates. The TLC plates were visualized by either ultraviolet light or treatment with a potassium permanganate ($KMnO_4$) stain followed by gentle heating. Purification of products was accomplished by flash column chromatography on silica gel 60 (Merck, particle diameter 40-63 μm). Unless otherwise specified, all reagents were purchased from commercial sources and used as received.

NMR spectra were recorded at room temperature either on a Bruker 400 MHz Avance III HD Smart Probe or Bruker 500 MHz Avance III HD Smart Probe (^{1}H, ^{13}C and 2D experiments). Chemical shifts of the NMR spectra are reported relative to $CDCl_3$ (^{1}H NMR: δ = 7.26 ppm, ^{13}C NMR: δ = 77.16 ppm), DMSO-d_6 (^{1}H NMR: δ = 2.50 ppm, ^{13}C NMR: δ = 39.52 ppm) or CD_3CN (^{1}H NMR: δ = 1.94 ppm, ^{13}C NMR: δ = 1.32; 118.26 ppm).[1] Data for ^{1}H NMR spectra were reported as follows: chemical shift (ppm), peak shape (s = singlet, d = doublet, t = triplet, m = multiplet), coupling constant (Hz), and integration. Data for ^{13}C NMR were reported in terms of chemical shift (ppm). DOSY experiments were performed on a Bruker 400 MHz Avance III HD Smart Probe. UV-vis measurements were employed to fine-tune the solution concentration for subsequent CD measurements, and performed on a Cary 300 UV-vis spectrophotometer with a 1 mm path-length cuvette at 298 K. Circular Dichroism was performed on an Applied Photophysics Chirascan qCD spectrometer using a 1 mm path-length cuvette. Experiments were performed at 298 K, maintained using a Peltier temperature controller. Measurements were background subtracted (blank solvent) in an identical cuvette, and concentrations were selected to maintain a HV below 800 Volts.

A microwave reactor from Discover SP-D 80-CEM Corporation was used for the stereochemical induction experiments. Centrifugation of samples was carried out using a Grant-Bio LMC-3000 low speed benchtop centrifuge. High-resolution mass spectra were acquired using a Thermofisher LTQ Orbitrap XL.

2. Synthesis and characterization of subcomponents

2.1 Synthesis of subcomponents B

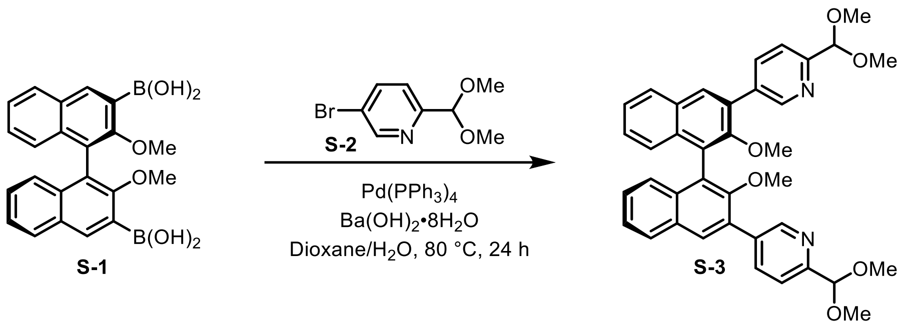

Compounds **S-1**[2,3] and **S-2**[4] were synthesized according to reported procedures.

Compound **S-3**: 5-Bromo-2-(dimethoxymethyl)pyridine (**S-2**) (5.2 g, 22.4 mmol, 4.0 eq.), $Ba(OH)_2 \cdot 8H_2O$ (7.1 g, 22.4 mmol, 4.0 eq.) and $Pd(PPh_3)_4$ (641.7 mg, 0.56 mmol, 10 mol%) were added to a solution of (*R*)-(2,2'-dimethoxy-[1,1'-binaphthalene]-3,3'-diyl)diboronic acid (**S-1**) (2.25 g, 5.6 mmol. 1.0 eq.) in degassed dioxane/water (80 mL, v/v = 3:1). The reaction mixture was stirred at 80 °C for 24 hours. After cooling to room temperature, the solvent was removed in vacuo, and the crude product was dissolved in dichloromethane (50 mL) and water (50 mL). The orgnaic phase was washed with brine, dried over Na_2SO_4, filtered, and concentrated in vacuo. The crude product was purified by flash column chromatography (SiO_2, pentane/dichloromethane/acetone = 10:1:1→5:1:1→3:1:1) to afford (*R*)-5,5'-(2,2'-dimethoxy-[1,1'-binaphthalene]-3,3'-diyl)bis(2-(dimethoxymethyl)pyridine) (**S-3**) as a pale yellow solid (1.45 g, 42% yield).

TLC: R_f = 0.25 (pentane/dichloromethane/acetone = 5:1:1) [UV].

^{1}H NMR (400 MHz, $CDCl_3$, 298 K) δ (ppm) = 9.0 (d, 2H), 8.17 (m, 2H), 8.01 (s, 2H), 7.95 (d, *J* = 8.2 Hz, 2H), 7.64 (d, *J* = 8.1 Hz, 2H), 7.47 – 7.42 (m, 2H), 7.31 (m, 2H), 7.22 (d, *J* = 8.4 Hz, 2H), 5.47 (s, 2H), 3.47 (s, 12H), 3.17 (s, 6H).

^{13}C{^{1}H} NMR (100 MHz, $CDCl_3$, 298 K) δ (ppm) = 156.1, 154.0, 149.3, 137. 6, 134.4, 134.1, 131.4, 131.0, 130.9, 128.4, 127.1, 126.0, 125.9, 125.6, 120.9, 104.1, 60.9, 53.9.

HRMS (ESI) calcd for $C_{38}H_{36}N_2O_6$ $[M + H]^+$: 617.2652; found: 617.2646.

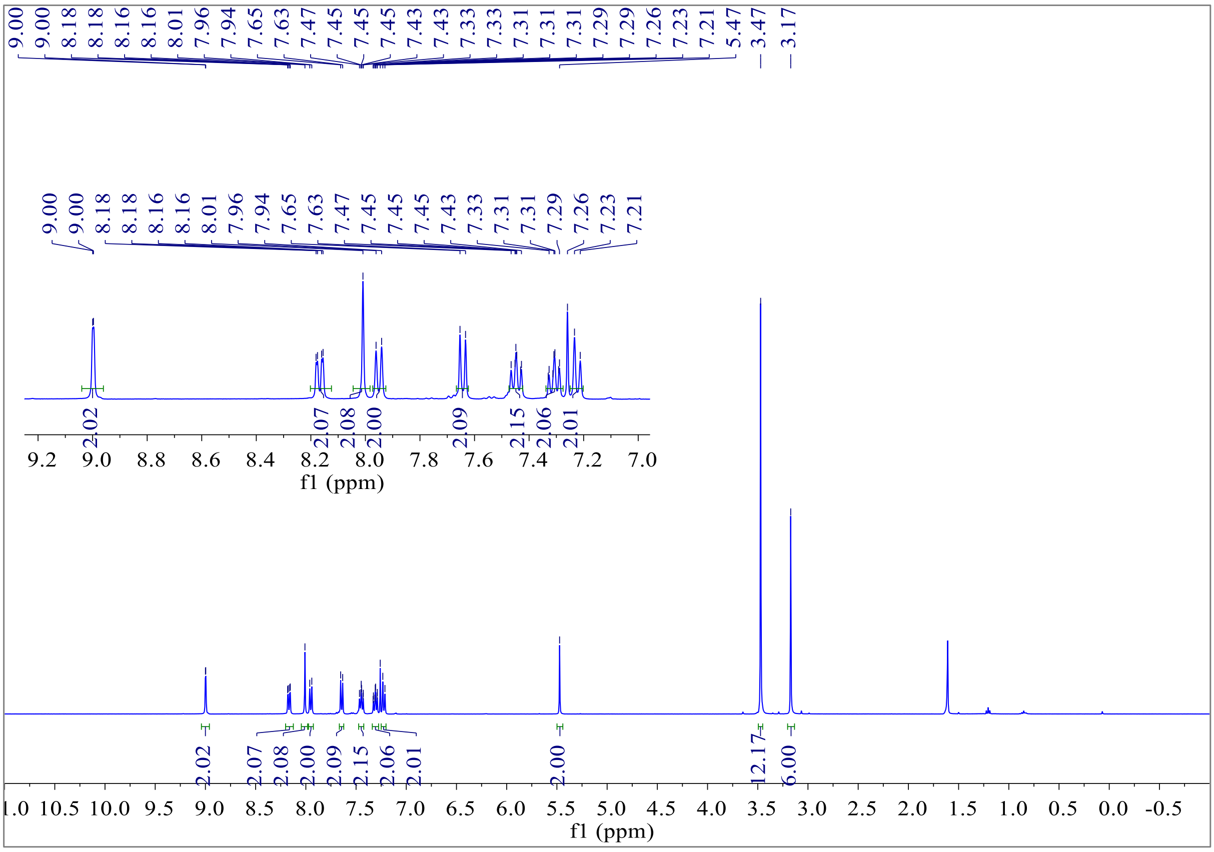

Figure S1. ^{1}H NMR spectrum of **S-3** (400 MHz, $CDCl_3$, 298 K).

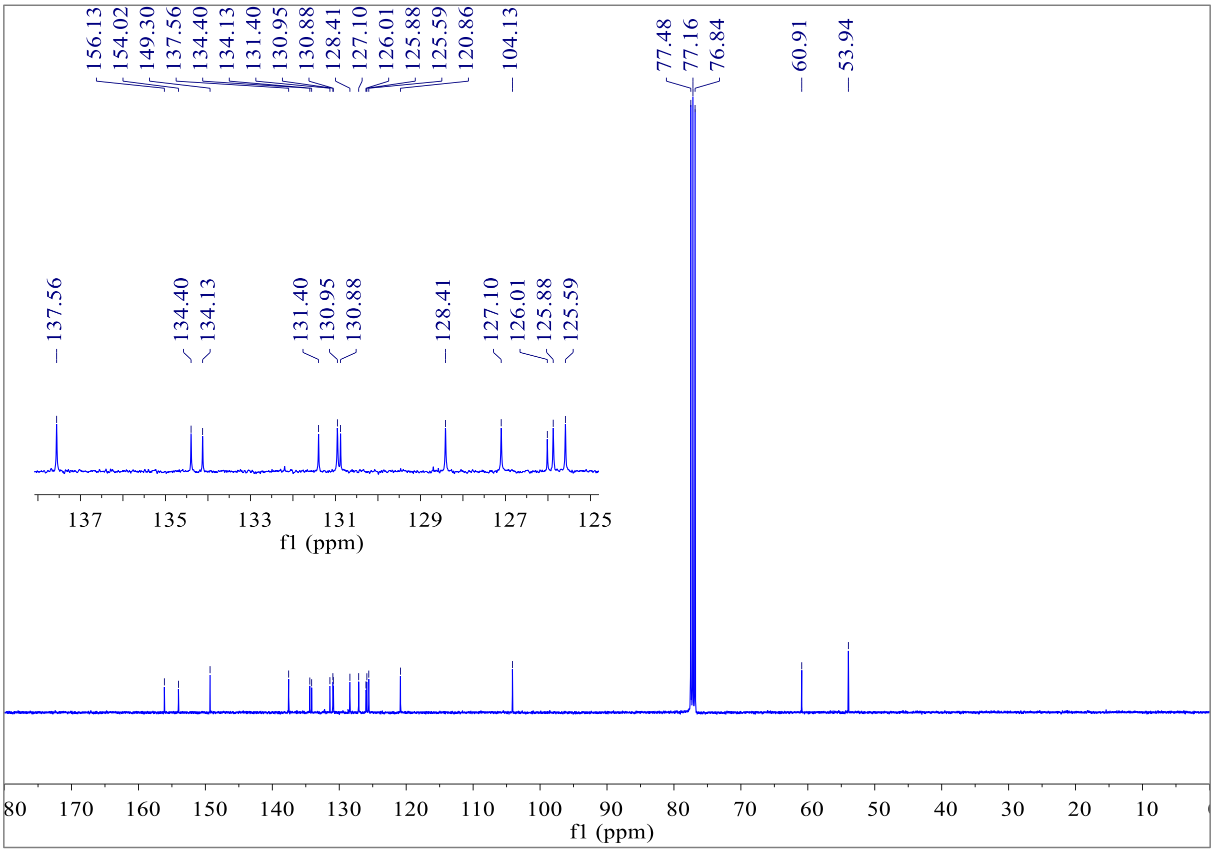

Figure S2. ^{13}C NMR spectrum of **S-3** (100 MHz, $CDCl_3$, 298 K).

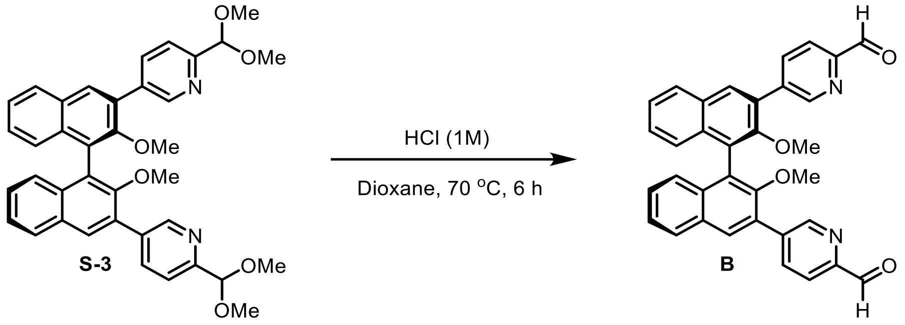

Subcomponent **B**: (*R*)-5,5'-(2,2'-Dimethoxy-[1,1'-binaphthalene]-3,3'-diyl)bis(2-(dimethoxymethyl)pyridine) (**S-3**) (61.7 mg, 0.1 mmol, 1.0 eq.) was dissolved in dioxane (2.5 mL). Then 1M HCl (1.5 mL) was added at room temperature. The reaction mixture was stirred at 70 °C for 6 hours. After cooling to 0 °C, saturated solution of Na_2CO_3 was added slowly to adjust the pH to ~ 7 followed by extraction with dichlomethane. The organic layers were combined, washed with brine, dried over Na_2SO_4, filtered, and concentrated in vacuo. The crude product was purified by flash column chromatography (SiO_2, pentane/dichloromethane/acetone = 5:1:1) to afford (*R*)-5,5'-(2,2'-dimethoxy-[1,1'-binaphthalene]-3,3'-diyl)dipicolinaldehyde (**B**) as a pale yellow solid (43.1 mg, 82% yield).

TLC: R_f = 0.35 (pentane/dichloromethane/acetone = 5:1:1) [UV].

^{1}H NMR (400 MHz, $CDCl_3$, 298 K) δ (ppm) = 10.17 (s, 2H), 9.19 (s, 2H), 8.31 (m, 2H), 8.08 (m, 4H), 7.99 (d, *J* = 8.2 Hz, 2H), 7.49 (t, *J* = 7.5 Hz, 2H), 7.36 (m, 2H), 7.24 (d, *J* = 8.5 Hz, 2H), 3.19 (s, 6H).

^{13}C{^{1}H} NMR (100 MHz, $CDCl_3$, 298 K) δ (ppm) = 193.3, 153.8, 151.7, 150.5, 138.9, 137.8, 134.4, 131.5, 130.9, 130.6, 128.7, 127.7, 126.1, 126.0, 125.9, 121.6, 61.1.

HRMS (ESI) calcd for $C_{34}H_{24}N_2O_4$ $[M + H]^+$: 525.1814; found 525.1819.

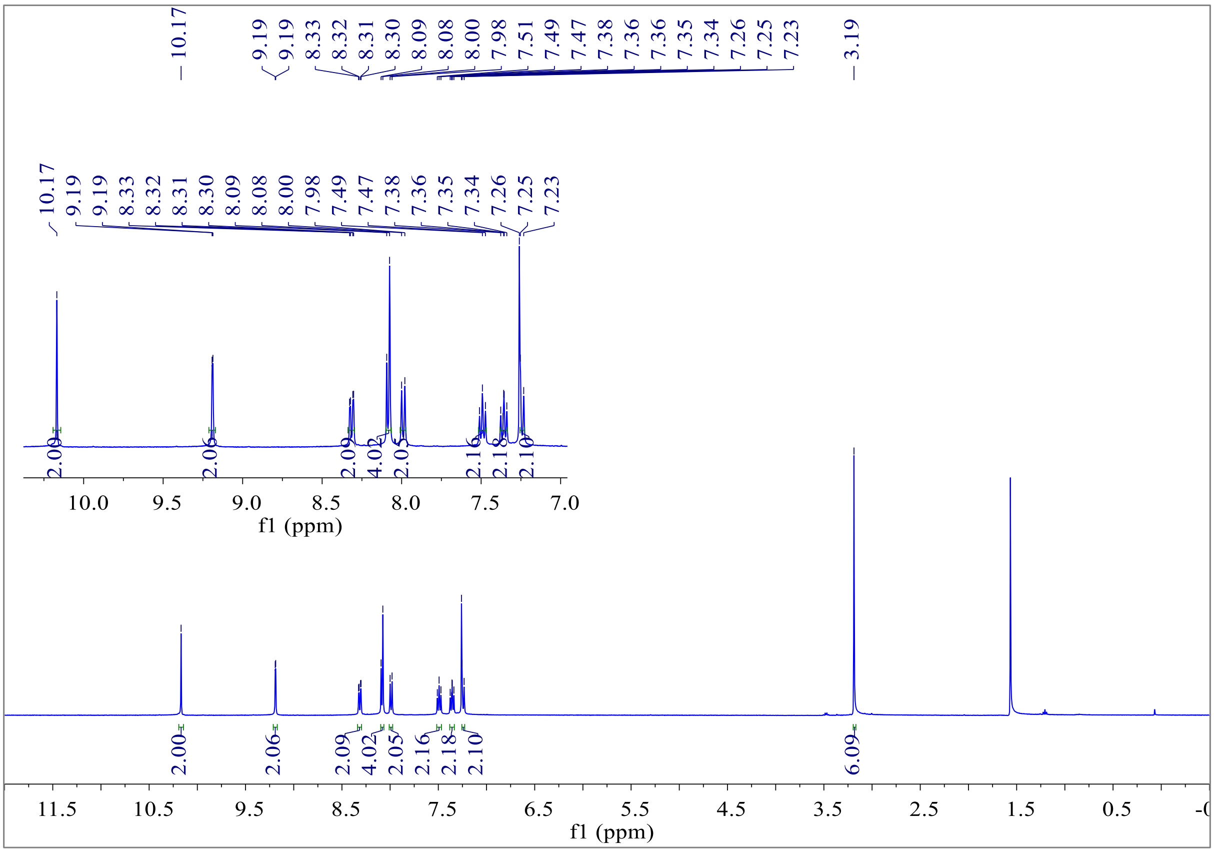

Figure S3. ^{1}H NMR spectrum of **B** (400 MHz, $CDCl_3$, 298 K).

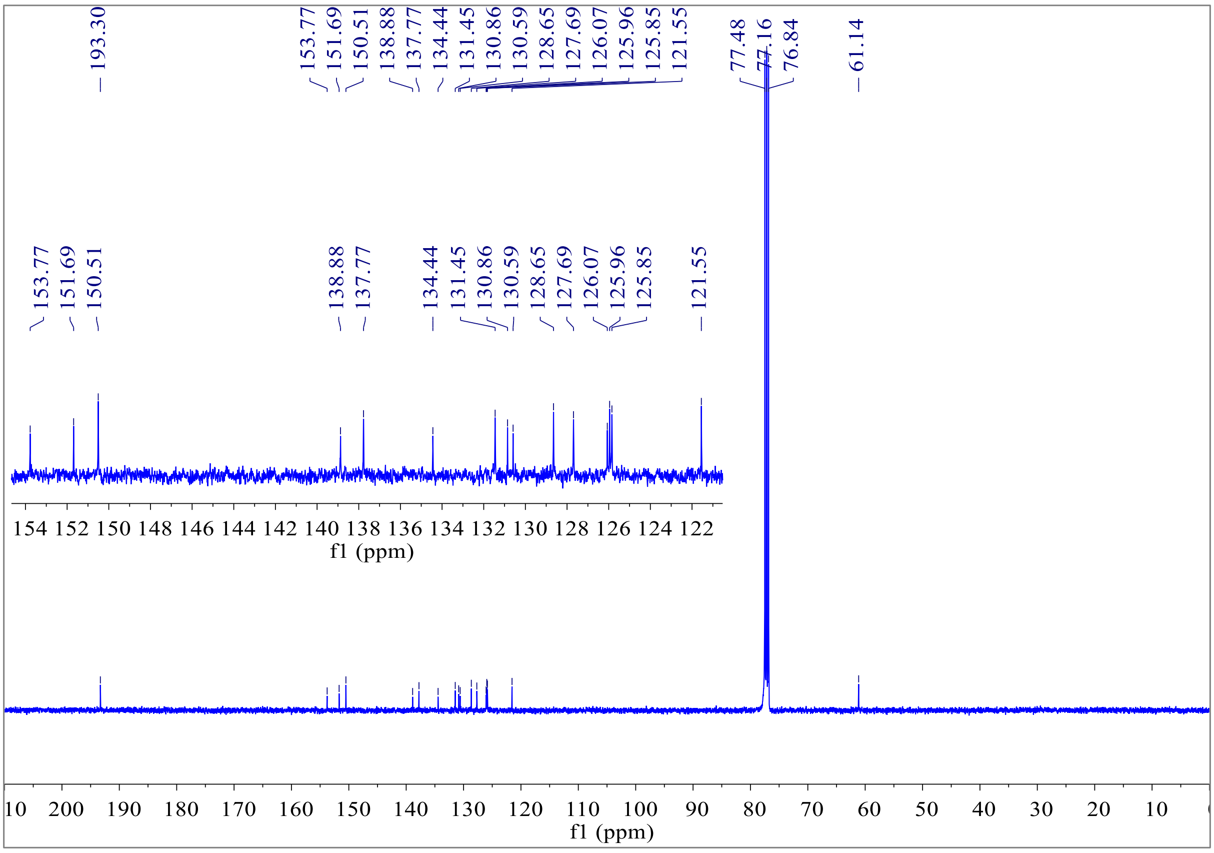

Figure S4. ^{13}C NMR spectrum of **B** (100 MHz, $CDCl_3$, 298 K).

2.2 Synthesis of subcomponent A

Method A: Subcomponent **A** was synthesized from subcomponent **B**.

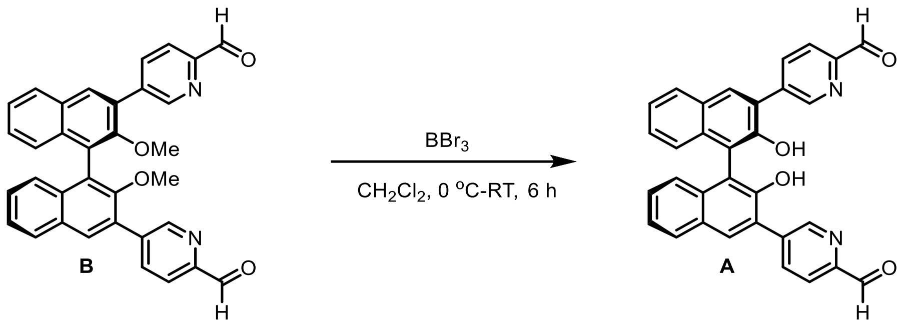

Subcomponent **A**: (*R*)-5,5'-(2,2'-dimethoxy-[1,1'-binaphthalene]-3,3'-diyl)dipicolinaldehyde (**B**) (10.5 mg, 0.02 mmol, 1.0 eq.) was dissolved in CH_2Cl_2 (2.0 mL), boron tribromide (1.0 M in CH_2Cl_2, 0.12 mL, 0.12 mmol, 6.0 eq.) was added dropwise at 0 °C. The reaction mixture was allowed to warm to room temperature and stirred for six hours. Water was slowly added at 0 °C to quench the reaction followed by extraction with CH_2Cl_2. The combined organic layers were dried over Na_2SO_4, filtered and concentrated in vacuo. The crude product was purified by flash column chromatography (SiO_2, pentane/dichloromethane/acetone = 5:1:1→3:1:1) to afford (*R*)-5,5'-(2,2'-dihydroxy-[1,1'-binaphthalene]-3,3'-diyl)dipicolinaldehyde (**A**) as a pale yellow solid (7.2 mg, 73% yield).

Method B: Subcomponent **A** was synthesized from **S-3**.

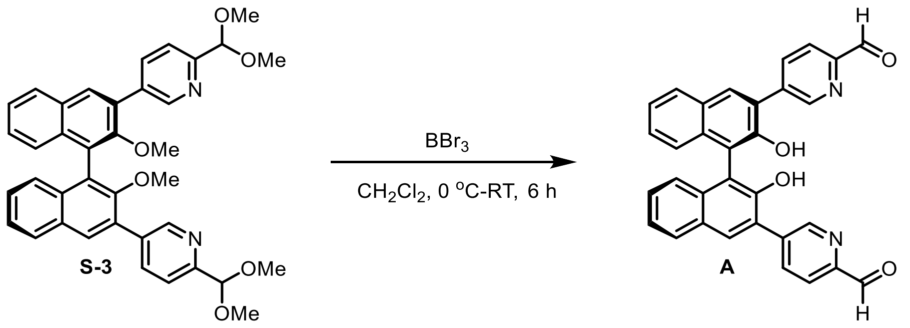

Subcomponent **A**: (*R*)-5,5'-(2,2'-Dimethoxy-[1,1'-binaphthalene]-3,3'-diyl)bis(2-(dimethoxymethyl)pyridine) (**S-3**) (616.7 mg, 1.0 mmol, 1.0 eq.) was dissolved in CH_2Cl_2 (30 mL), boron tribromide (1.0 M in CH_2Cl_2, 6 mL, 6 mmol, 6.0 eq.) was added dropwise at 0 °C. The reaction mixture was allowed to warm to room temperature and stirred for six hours. Water was slowly added at 0 °C to quench the reaction followed by extraction with CH_2Cl_2. The combined organic layers were dried over Na_2SO_4, filtered and concentrated in vacuo. The crude product was purified by flash column chromatography (SiO_2, pentane/dichloromethane/acetone = 5:1:1→3:1:1) to afford (*R*)-5,5'-(2,2'-dihydroxy-[1,1'-binaphthalene]-3,3'-diyl)dipicolinaldehyde (**A**) as a pale yellow solid (392.3 mg, 79% yield).

TLC: R_f = 0.1 (pentane/dichloromethane/acetone = 5:1:1) [UV].

^{1}H NMR (400 MHz, $CDCl_3$, 298 K) δ (ppm) = 10.45 (s, 2H), 9.40 (s, 2H), 8.81 (s, 2H), 7.93 (d, *J* = 7.9 Hz, 2H), 7.57 (m, 4H), 7.34 – 7.28 (m, 4H), 7.25 (d, *J* = 6.8 Hz, 2H), 7.16 (d, *J* = 8.1 Hz, 2H).

^{13}C{^{1}H} NMR (100 MHz, $CDCl_3$, 298 K) δ (ppm) = 188.0, 150.4, 149.6, 147.8, 137.5, 135.8, 134.0, 129.6, 127.9, 127.6, 127.1, 127.0, 123.7, 123.6, 119.8, 114.4.

HRMS (ESI) calcd for $C_{32}H_{20}N_2O_4$ $[M + H]^+$: 497.1501; found: 497.1515.

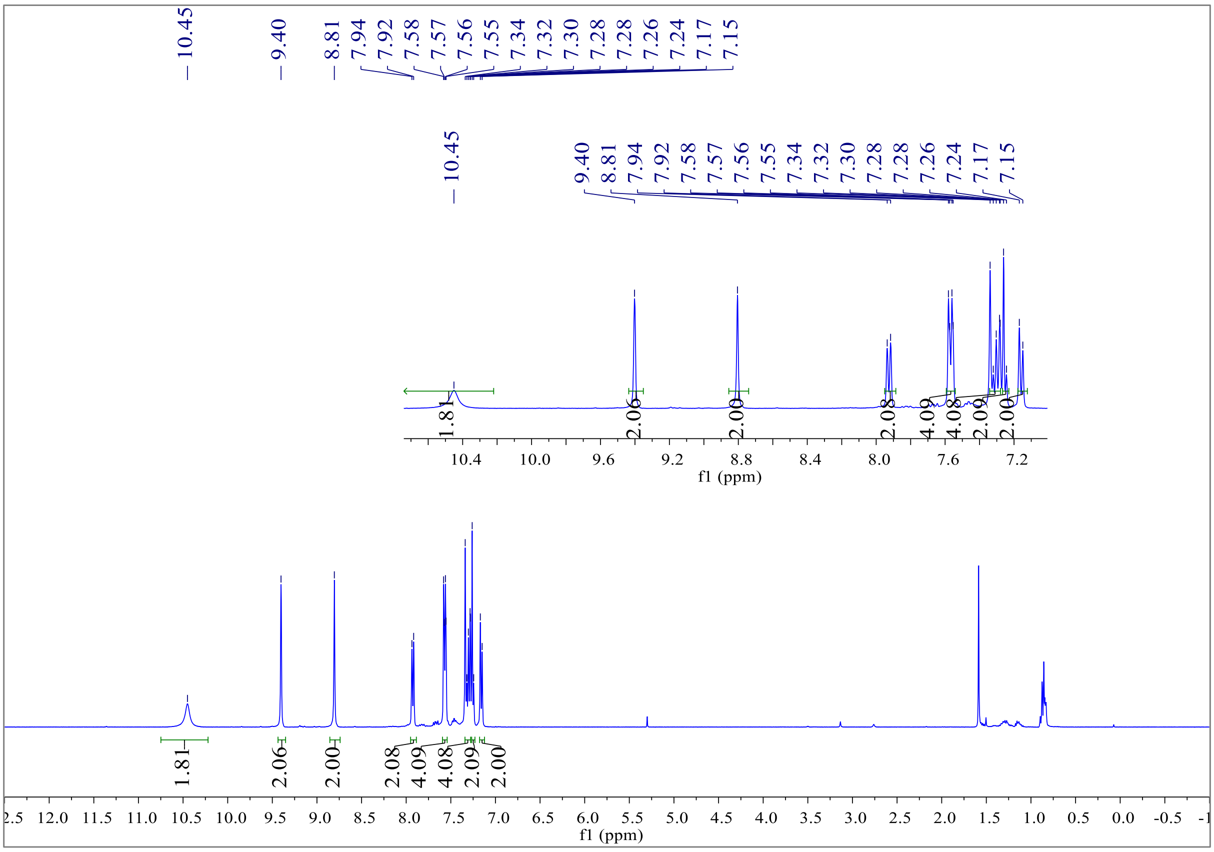

Figure S5. ^{1}H NMR spectrum of **A** (400 MHz, $CDCl_3$, 298 K).

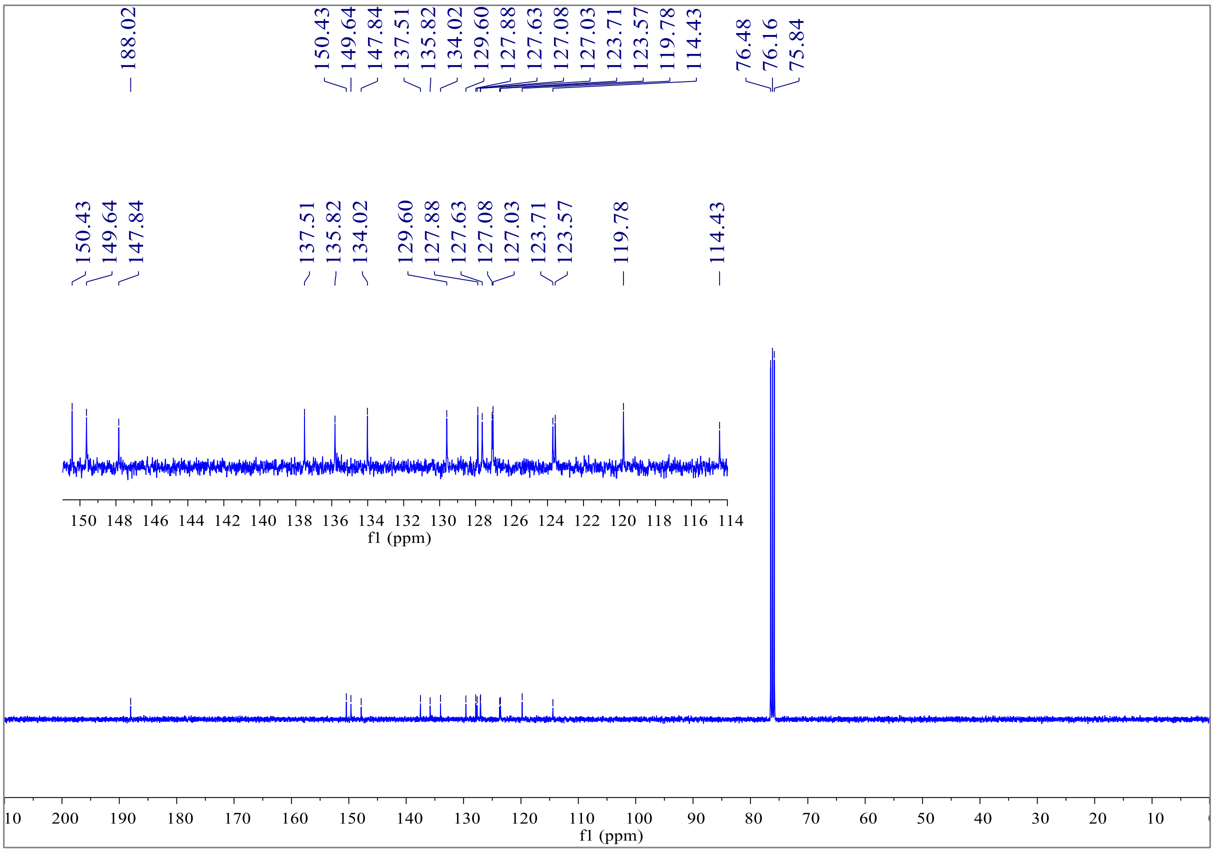

Figure S6. ^{13}C NMR spectrum of **A** (100 MHz, $CDCl_3$, 298 K).

2.3 Synthesis of subcomponent D

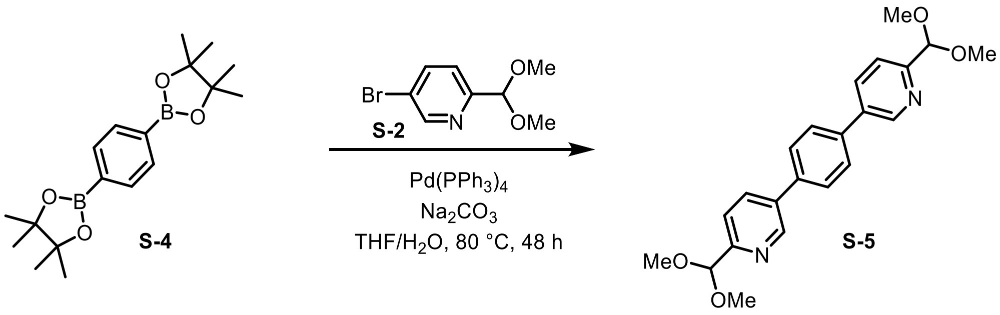

Compound **S-2**[4] was synthesized according to reported procedure. Compound **S-4** was purchased from Fluorochem and used as received.

Compound **S-5**: 5-Bromo-2-(dimethoxymethyl)pyridine (**S-2**) (1.2 g, 5.0 mmol, 5.0 eq.), Na_2CO_3 (530 mg, 5.0 mmol, 5.0 eq.) and $Pd(PPh_3)_4$ (57.8 mg, 0.05 mmol, 0.1 mol%) were added to a solution of 1,4-benzenediboronic acid bis(pinacol) ester (**S-4**) (330 mg, 1.0 mmol. 1.0 eq.) in degassed tetrahydrofuran/water (20 mL, v/v = 3:1). The reaction mixture was stirred at 80 °C for 48 hours. After cooling to room temperature, the solvent was removed in vacuo, and the crude product was suspended in dichloromethane (100 mL) and water (25 mL). The orgnaic phase was washed with brine and concentrated in vacuo to afford 1,4-bis(6-(dimethoxymethyl)pyridin-3-

yl)benzene (**S-5**) as a pale yellow solid (crude product, 320 mg, 42% yield). **S-5** was used directly for the next step.

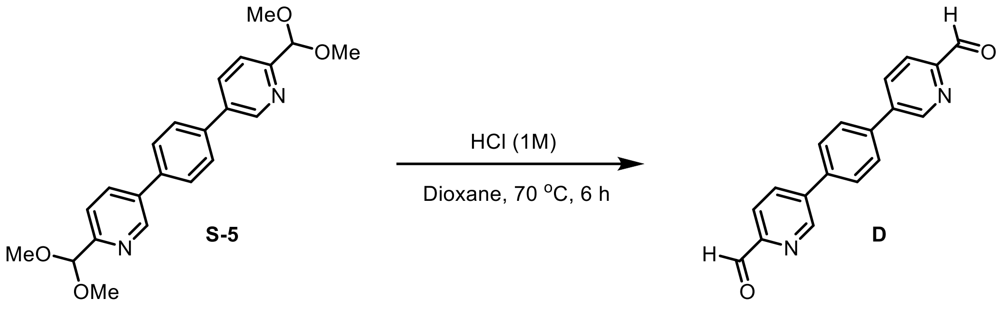

Subcomponent **D**: 1,4-Bis(6-(dimethoxymethyl)pyridin-3-yl)benzene (**S-3**) (320 mg, 0.84 mmol, 1.0 eq.) was dissolved in dioxane (20 mL). Then 1M HCl (12 mL) was added at room temperature. The reaction mixture was stirred at 70 °C for 6 hours. After cooling to 0 °C, saturated solution of Na_2CO_3 was added slowly to adjust the pH to ~ 7. Subsequently, the solvent was reomoved under vacuo. Then water and dichloromethane were added, and the organic layer was washed with brine. After removing the solvent under vacuo, the crude product was washed with dichloromethane, methanol and pentane, followed by further dried in vacuo to afford 5,5'-(1,4-phenylene)dipicolinaldehyde (**D**) as a pale yellow solid (193.8 mg, 80% yield).

^{1}H NMR (400 MHz, DMSO-d_6, 298 K) δ (ppm) = 10.06 (s, 2H), 9.27 (s, 1H), 9.26 (s, 1H), 8.46 (d, *J* = 2.2 Hz, 1H), 8.44 (d, *J* = 2.2 Hz, 1H), 8.07 – 8.04 (m, 6H).

^{13}C{^{1}H} NMR (100 MHz, DMSO-d_6, 298 K) δ (ppm) = 193.3, 151.4, 148.4, 138.7, 136.5, 135.5, 128.3, 128.2, 127.7, 122.0.

The ^{1}H and ^{13}C{^{1}H} NMR spectra are consistent with those reported in the literature.[5]

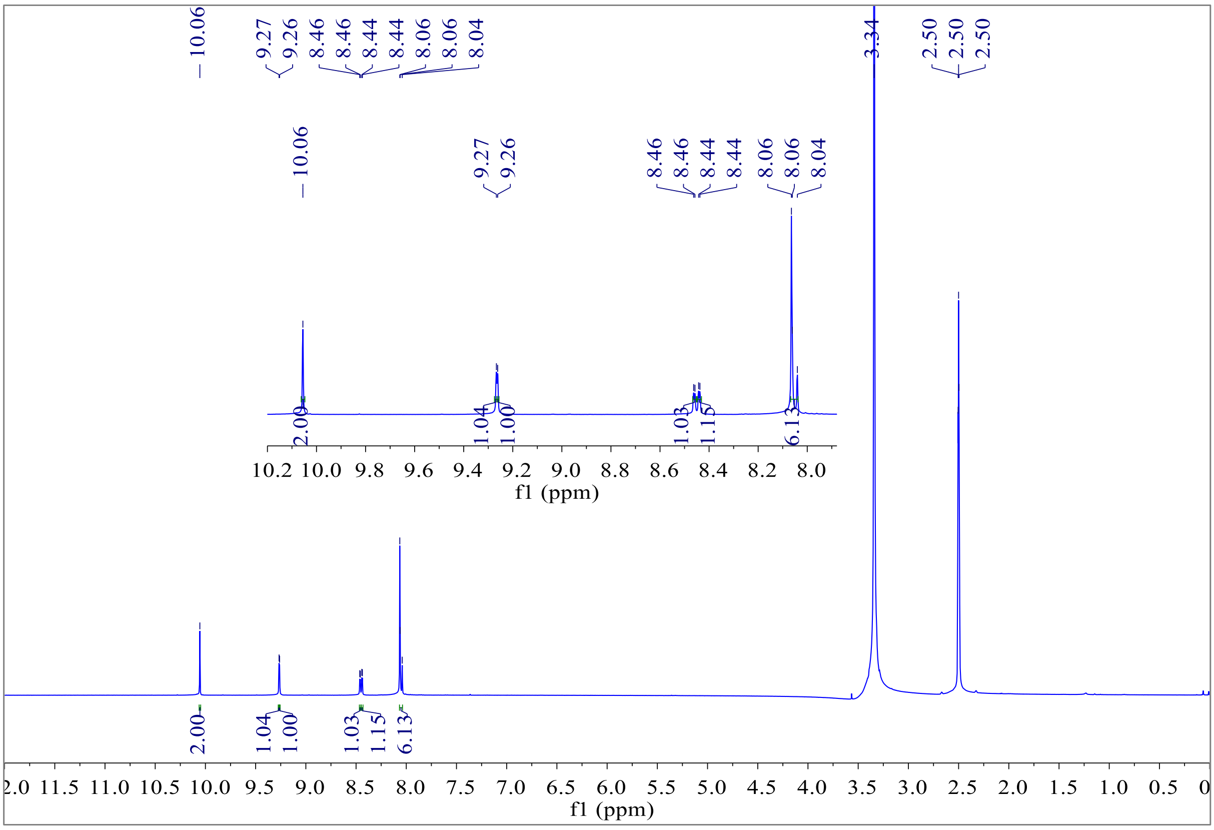

Figure S7. ^{1}H NMR spectrum of **D** (400 MHz, DMSO-d_6, 298 K).

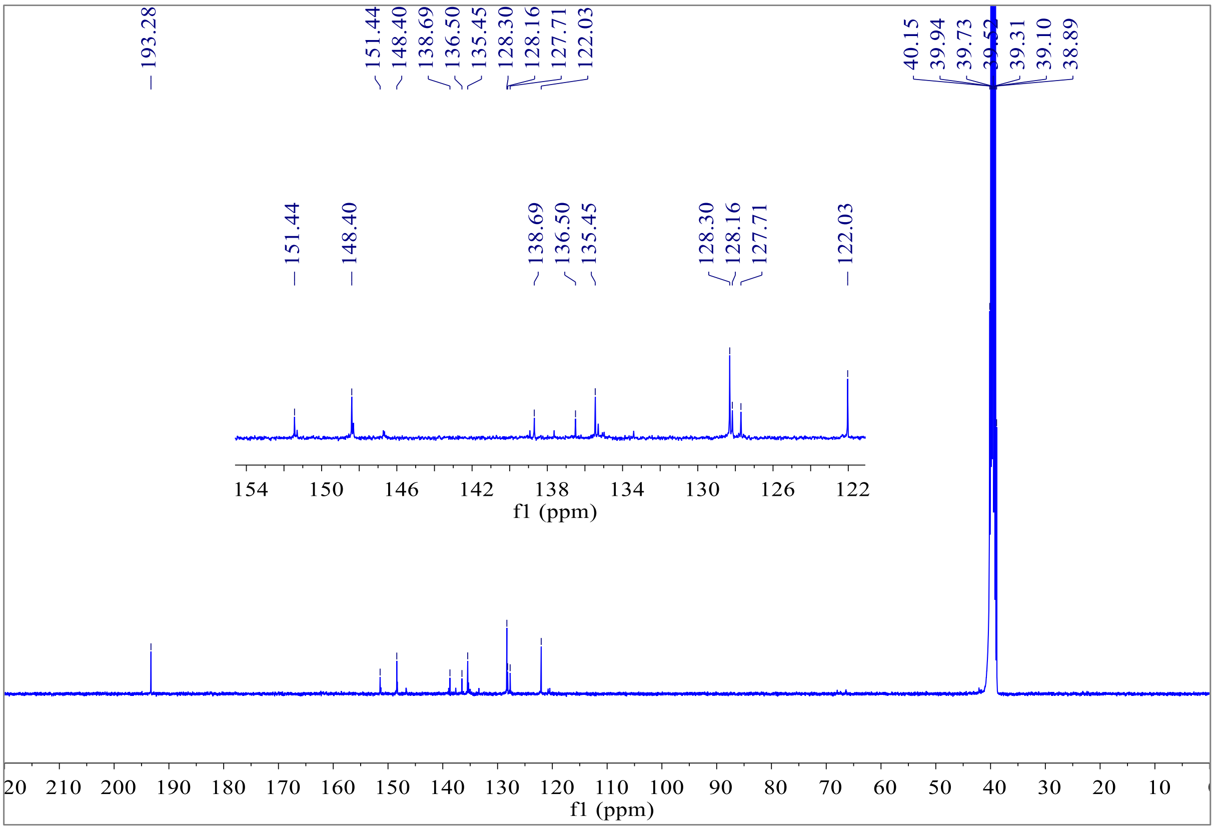

Figure S8. ^{13}C NMR spectrum of **D** (100 MHz, DMSO-d_6, 298 K).

3. Self-assembly using subcomponent A

3.1 Sole subcomponent A

3.1.1 with Zn(II) salt

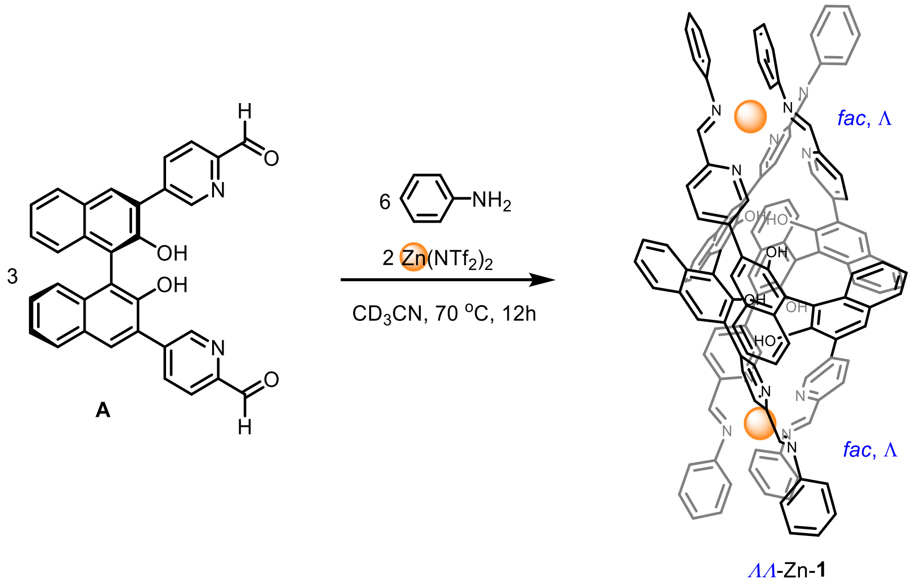

Subcomponent **A** (1.9 mg, 3.8 μmol, 1.5 equiv) was added to CD_3CN (0.6 mL) together with $Zn(NTf_2)_2$ (1.6 mg, 2.5 μmol, 1.0 equiv) and aniline (0.7 mg, 7.5 μmol, 3.0 equiv). The reaction mixture was stirred at 70 °C for 12h. After cooling to room temperature, the solvent was evaporated and diethyl ether was then added. The residue resuspended and then centrifuged and the diethyl ether decanted. This was repeated three times with fresh diethyl ether. The residue was then dried in vacuo to afford the desired product (*ΛΛ*-Zn-**1**) as a pale yellow solid (3.0 mg, 75% yield).

Characterization of *ΛΛ*-Zn-1:

^{1}H NMR (400 MHz, CD_3CN, 298 K) δ (ppm) = 8.53 (s, 6H), 8.01 (d, *J* = 8.2 Hz, 6H), 7.77 (d, *J* = 8.0 Hz, 6H), 7.61 (t, *J* = 7.5 Hz, 6H), 7.48 – 7.40 (m, 18H), 7.29 (t, *J* = 7.4 Hz, 6H), 7.21 (t, *J* = 7.6 Hz, 12H), 6.71 (m, 18H), 6.61 (m, 6H).

^{13}C{^{1}H} DEPT-135 (100 MHz, CD_3CN, 298 K) δ (ppm) = 164.0, 151.2, 139.4, 130.6, 130.58, 130.4, 129.8, 129.3, 128.6, 125.2, 125.1, 122.7.

ESI-MS *m/z* 517.6 $[M]^{4+}$.

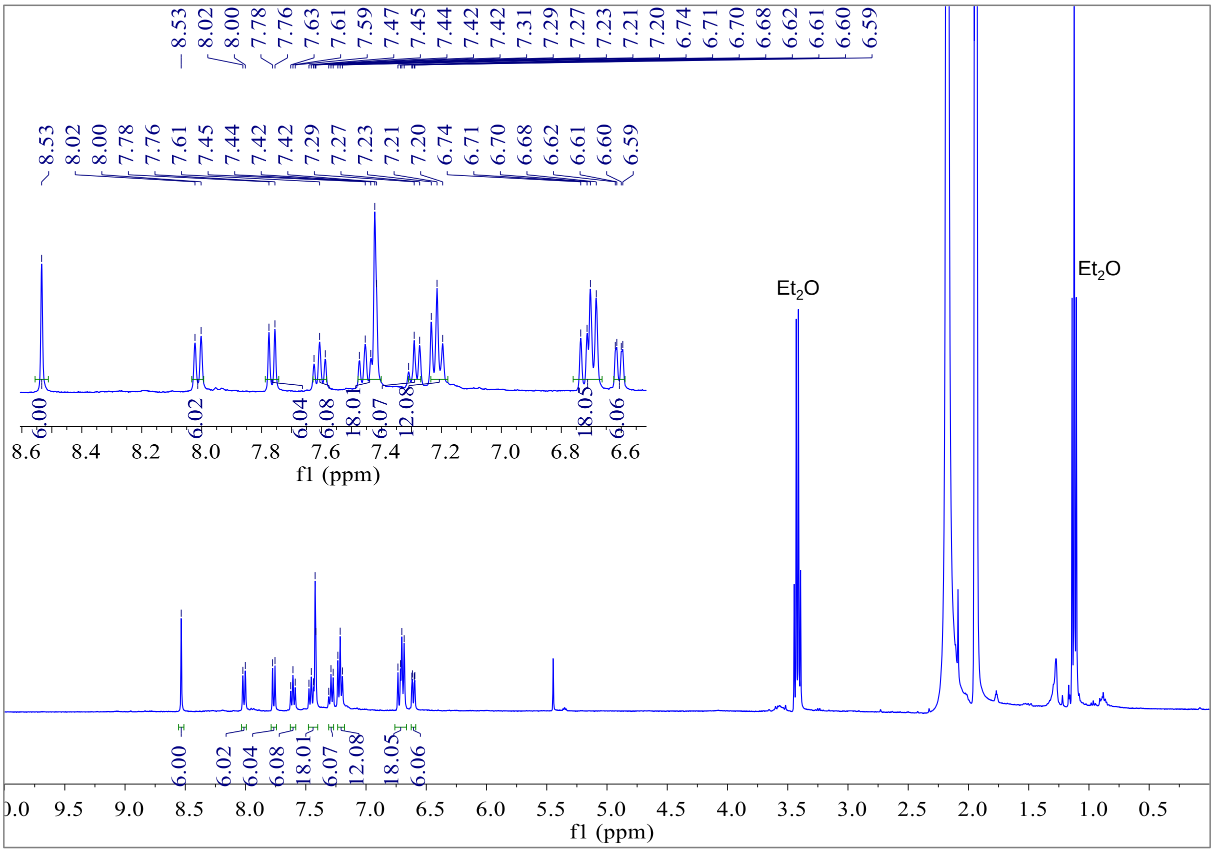

Figure S9. ^{1}H NMR spectrum of *ΛΛ*-Zn-**1** (400 MHz, CD3CN, 298 K).

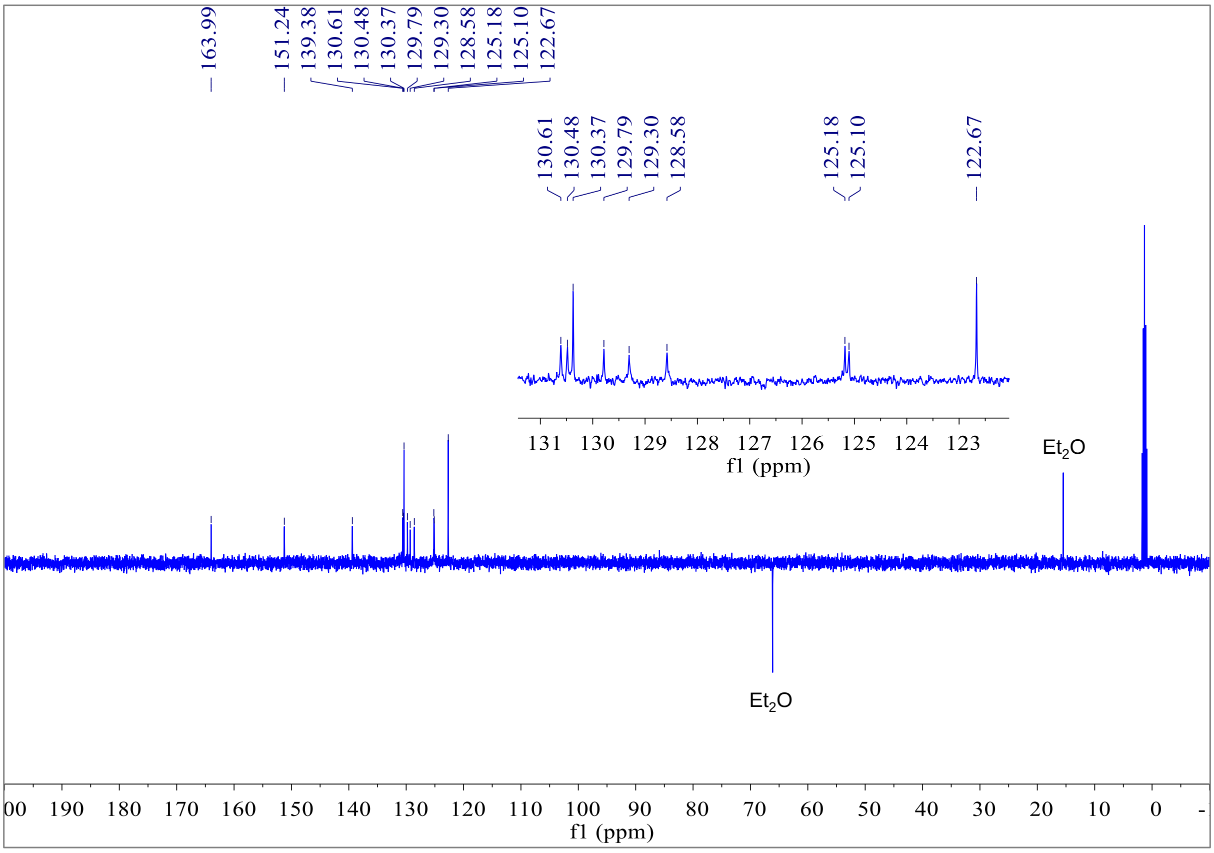

Figure S10. ^{13}C{^{1}H} DEPT-135 NMR spectrum of *ΛΛ*-Zn-**1** (100 MHz, CD3CN, 298 K).

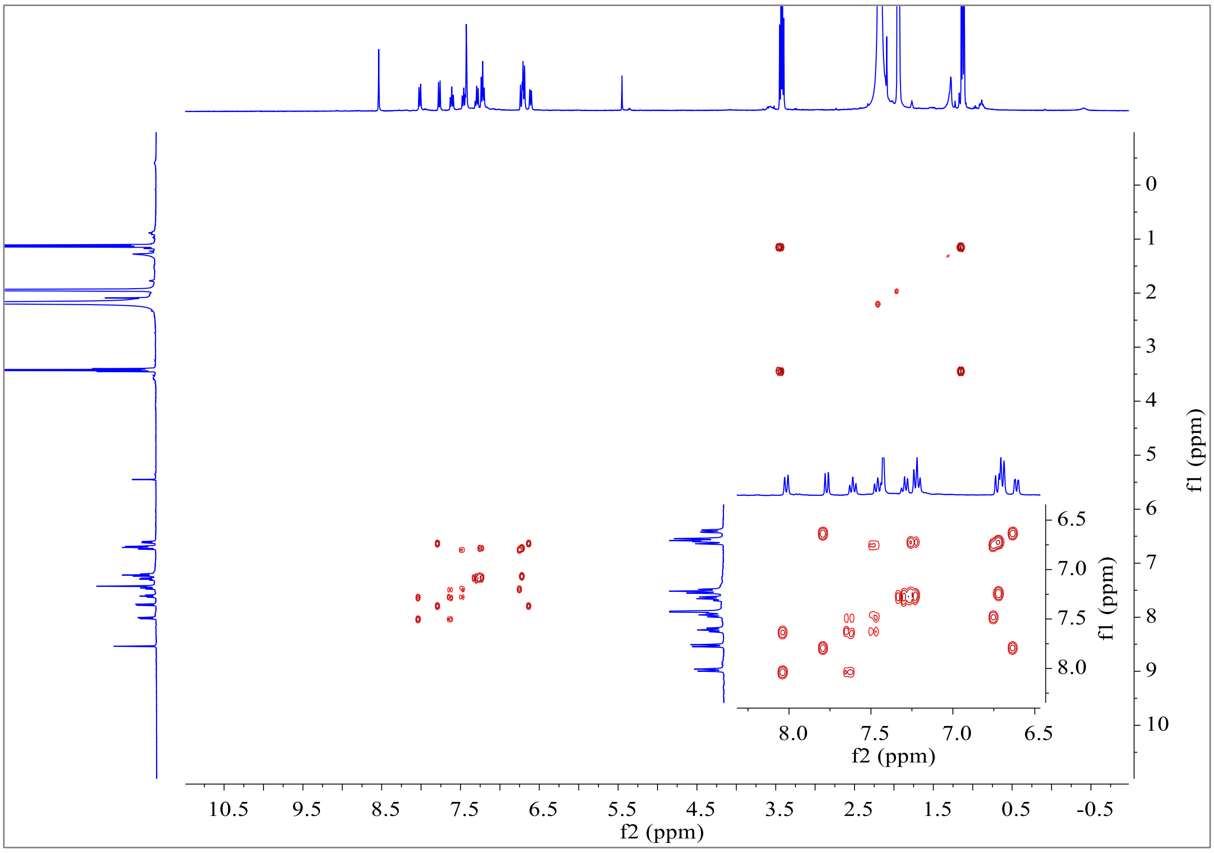

Figure S11. ^{1}H{^{1}H} COSY NMR spectrum of *ΛΛ*-Zn-**1** (400 MHz, CD_3CN, 298 K).

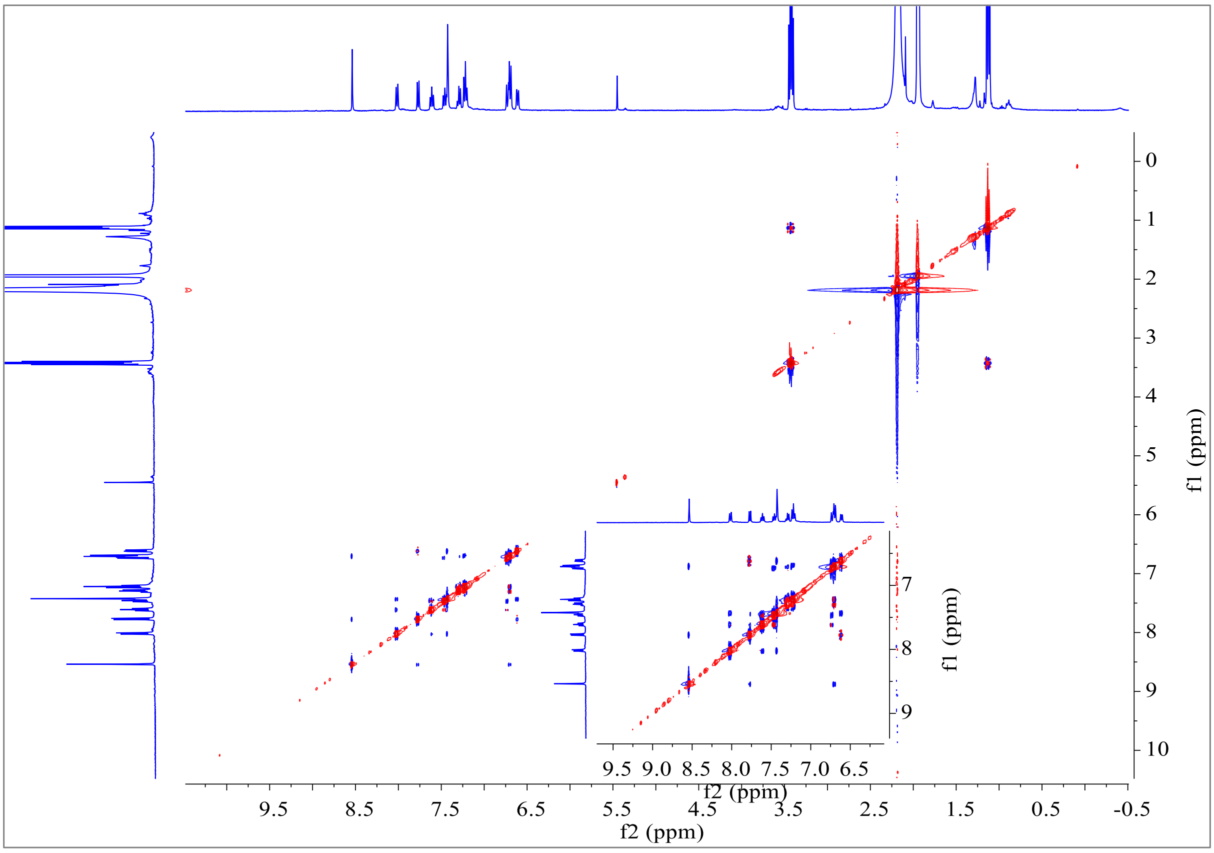

Figure S12. ^{1}H{^{1}H} NOESY NMR spectrum of *ΛΛ*-Zn-**1** (400 MHz, CD_3CN, 298 K).

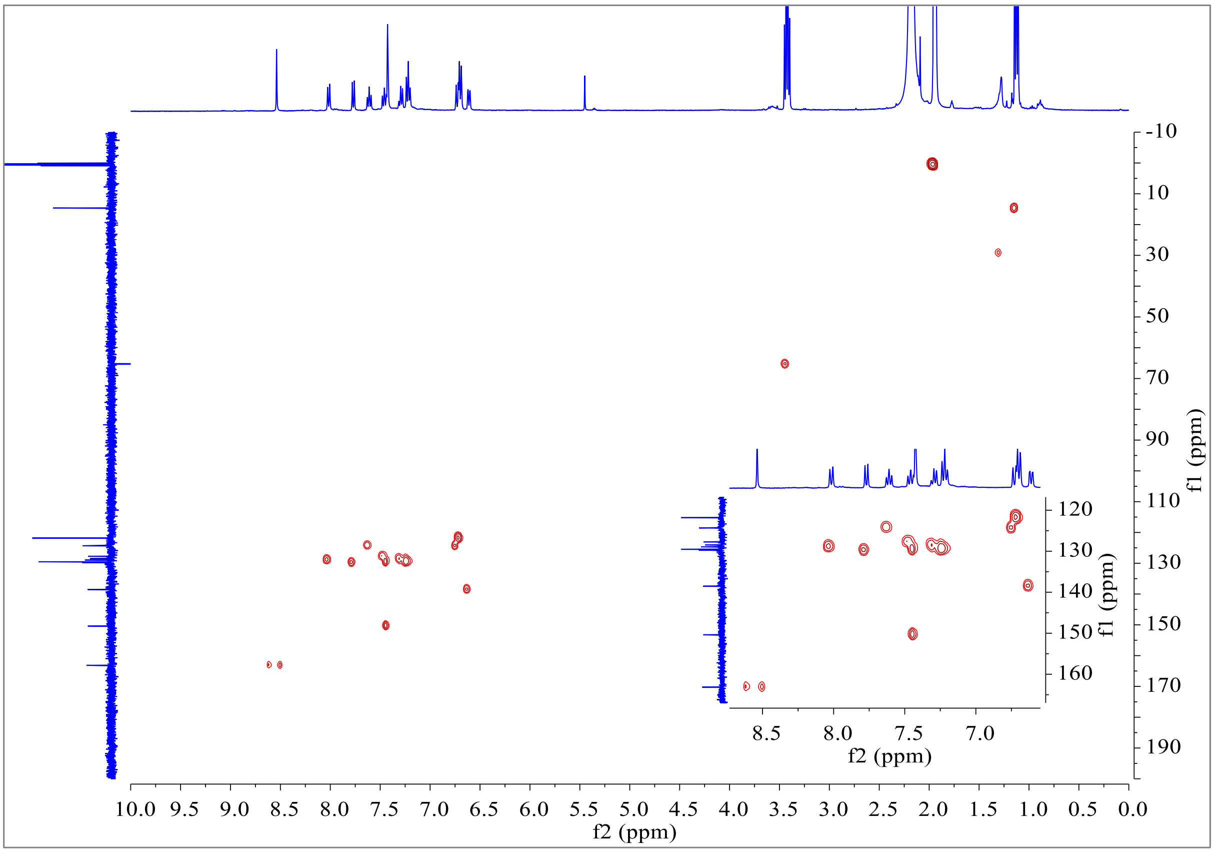

Figure S13. $^{1}H\{^{13}C\}$ HSQC NMR spectrum of *ΛΛ*-Zn-**1** (400 MHz, CD_3CN, 298 K).

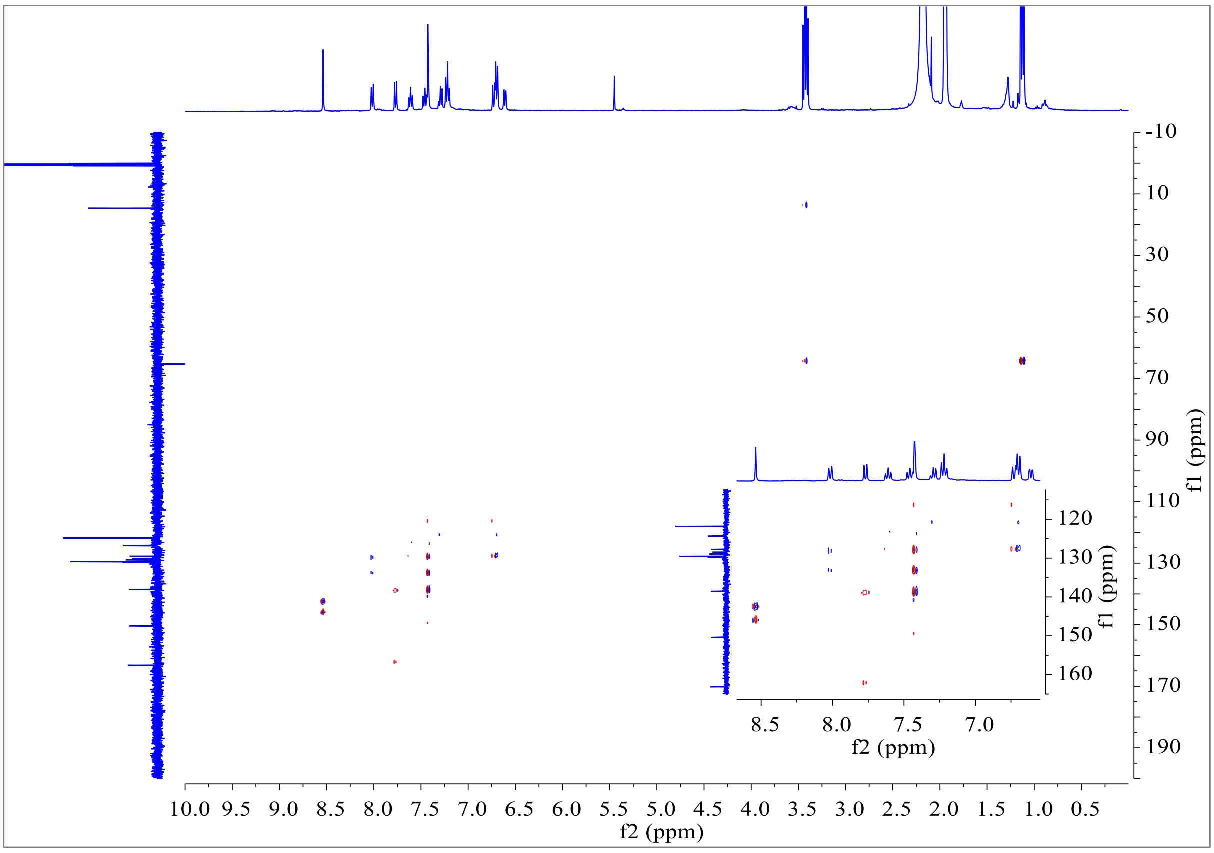

Figure S14. $^{1}H\{^{13}C\}$ HMBC NMR spectrum of *ΛΛ*-Zn-**1** (400 MHz, CD_3CN, 298 K).

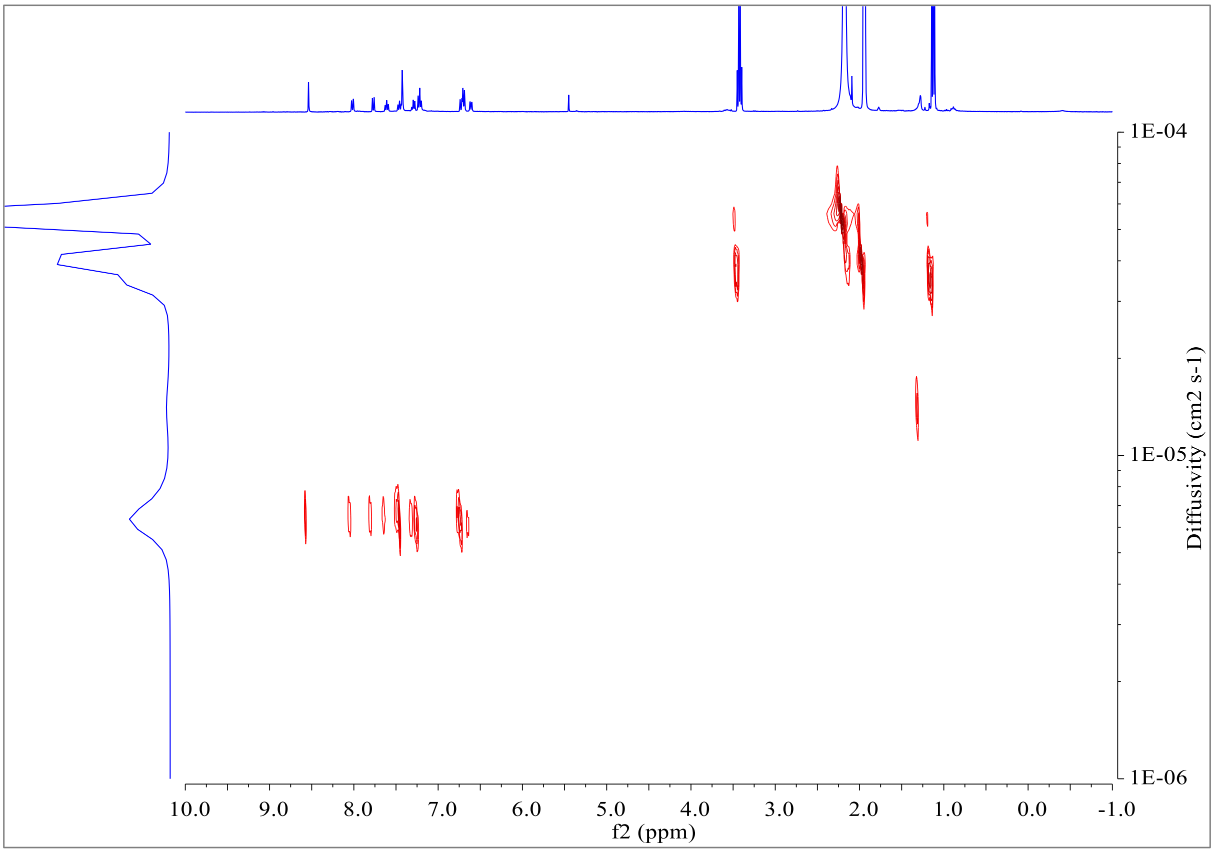

Figure S15. ^{1}H DOSY NMR spectrum of *ΛΛ*-Zn-**1** (400 MHz, CD_3CN, 298 K).

3.1.2 with Fe(II) salt

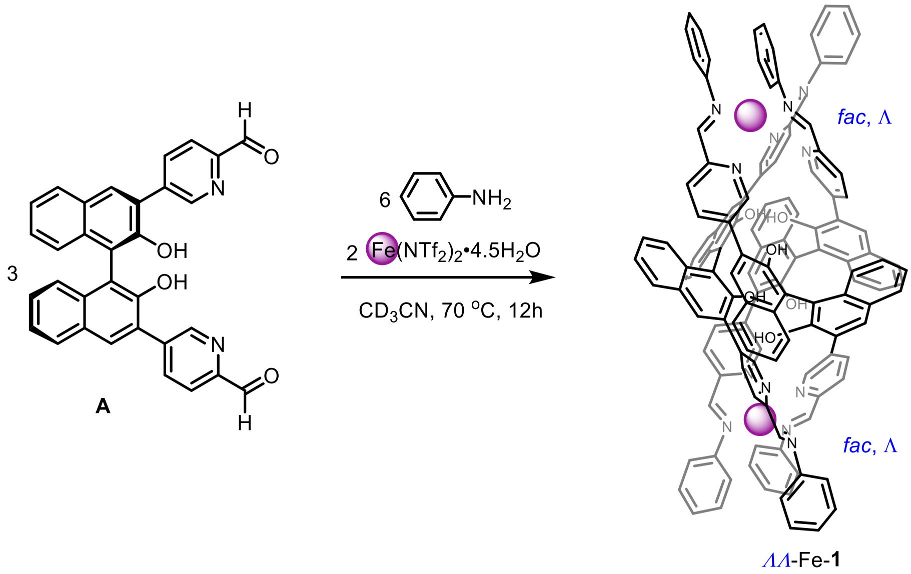

Subcomponent **A** (1.9 mg, 3.8 μmol, 1.5 equiv) was added to CD_3CN (0.6 mL) together with $Fe(NTf_2)_2 \cdot 4.5H_2O$ (1.7 mg, 2.5 μmol, 1.0 equiv) and aniline (0.7 mg, 7.5 μmol, 3.0 equiv). The reaction mixture was stirred at 70 °C for 12h. After cooling to room temperature, the solvent was evaporated and diethyl ether was then added. The residue resuspended and then centrifuged and the diethyl ether decanted. This was repeated three times with fresh diethyl ether. The residue was then dried in vacuo to afford the desired product (*ΛΛ*-Fe-**1**) as a purple solid (3.2 mg, 80% yield).

Characterization of *ΛΛ*-Fe-1:

^{1}H NMR (400 MHz, CD_3CN, 298 K) δ (ppm) = 9.14 (s, 6H), 8.18 (d, *J* = 8.1 Hz, 6H), 8.00 (d, *J* = 8.1 Hz, 6H), 7.71 (s, 6H), 7.61 (d, *J* = 7.4 Hz, 6H), 7.52 (s, 6H), 7.44 (d, *J* = 7.6 Hz, 6H), 7.31 (d, *J* = 7.4 Hz, 6H), 7.19 (t, *J* = 7.7 Hz, 12H), 6.77 (dd, *J* = 8.1, 1.5 Hz, 6H), 6.68 (d, *J* = 8.4 Hz, 6H), 5.51 (d, *J* = 7.8 Hz, 12H).

^{13}C{^{1}H} DEPT-135 (100 MHz, CD_3CN, 298 K) δ (ppm) = 172.6, 157.8, 136.7, 131.8, 130.4, 130.0, 129.8, 129.3, 128.7, 125.1, 125.0, 122.7.

ESI-MS *m/z* 512.8 $[M]^{4+}$.

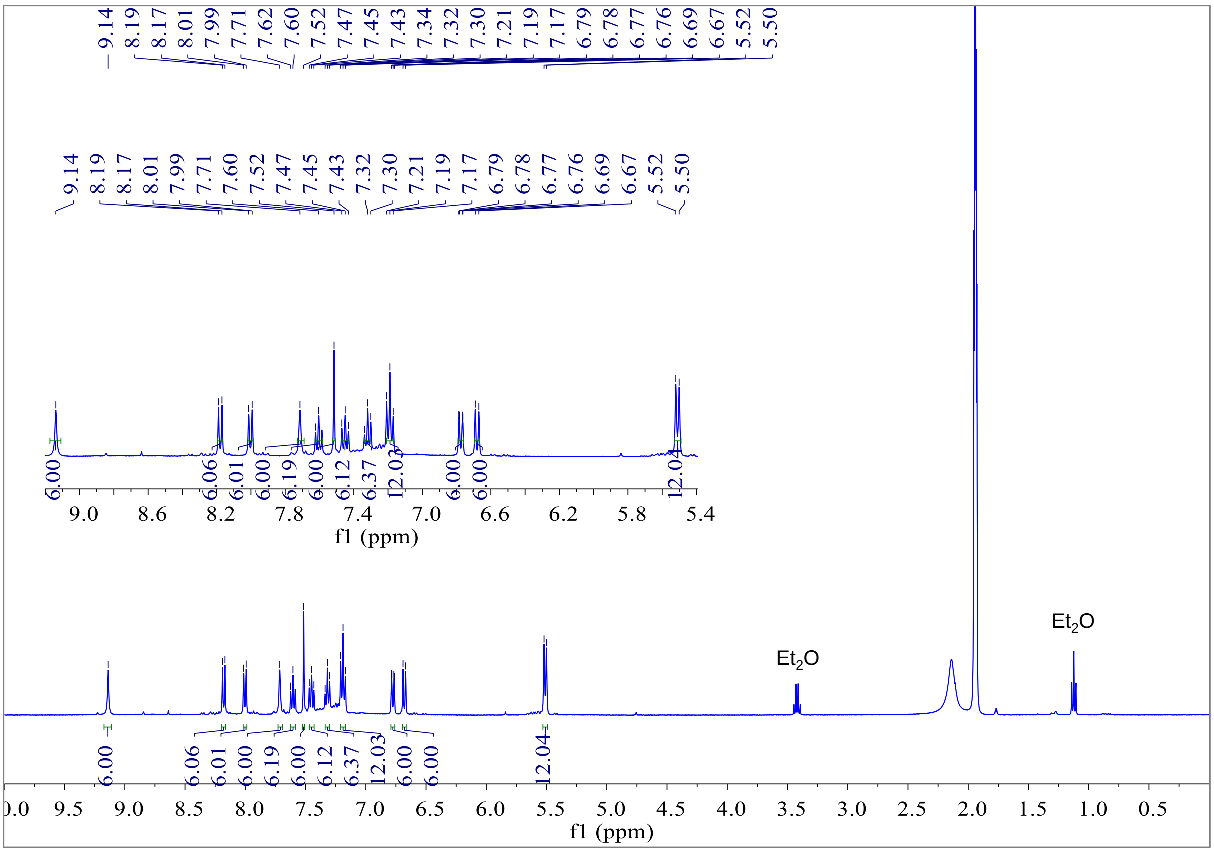

Figure S16. ^{1}H NMR spectrum of *ΛΛ*-Fe-**1** (400 MHz, CD_3CN, 298 K).

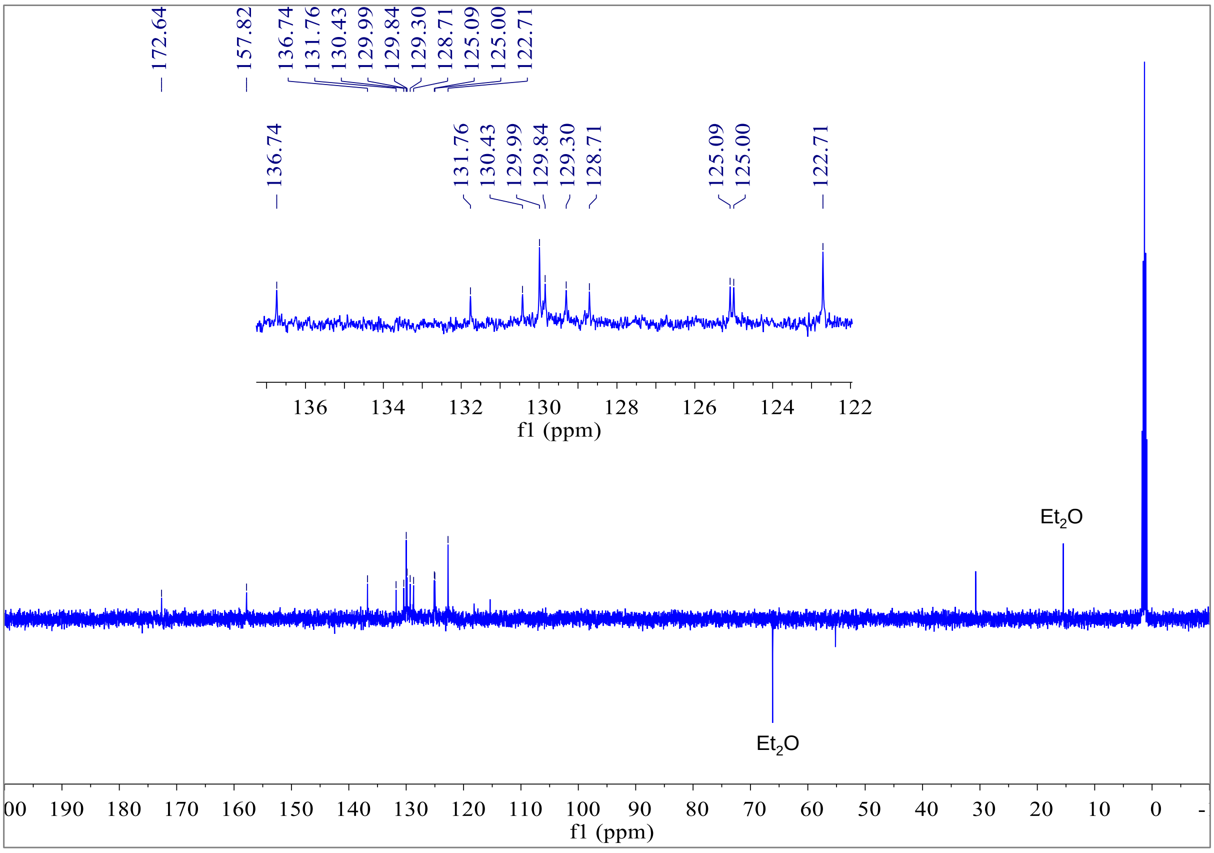

Figure S17. $^{13}C\{^{1}H\}$ DEPT-135 NMR spectrum of *ΛΛ*-Fe-**1** (100 MHz, CD_3CN, 298 K).

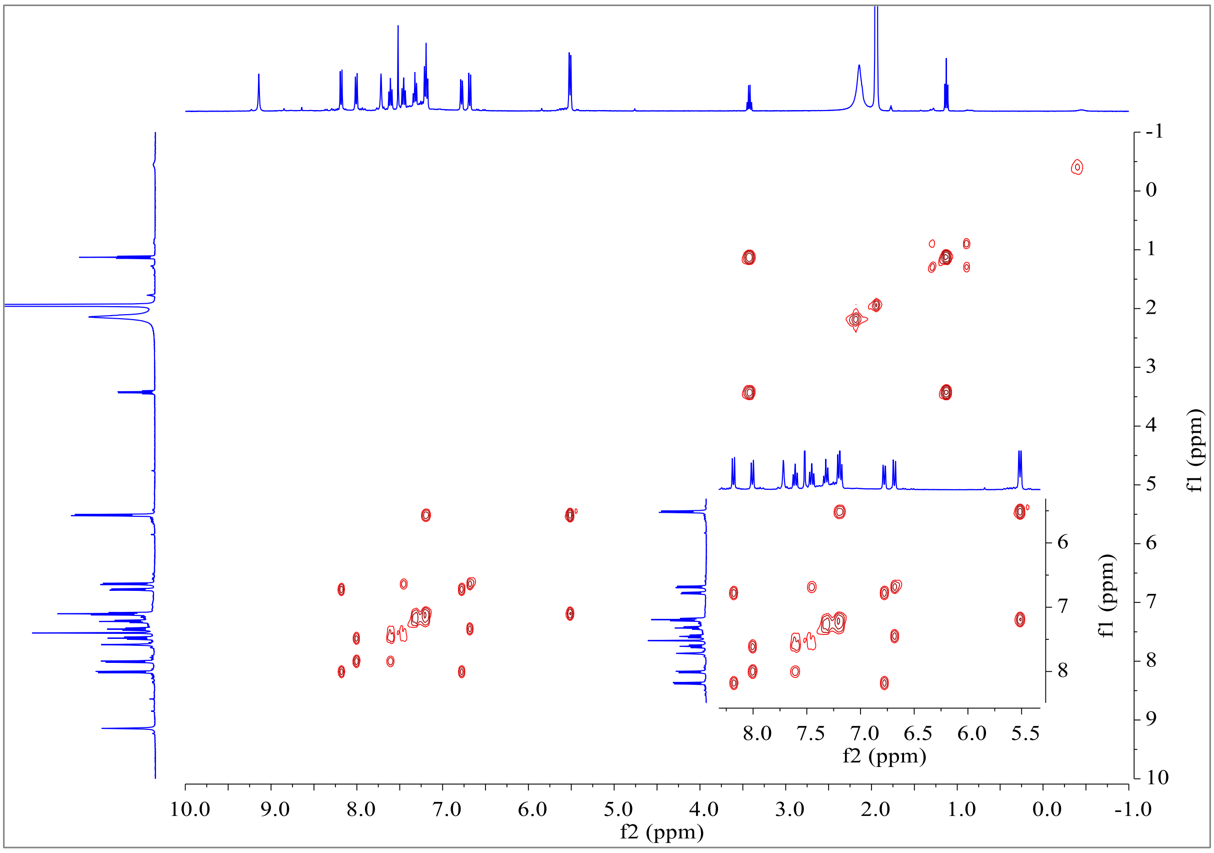

Figure S18. $^{1}H\{^{1}H\}$ COSY NMR spectrum of *ΛΛ*-Fe-**1** (400 MHz, CD_3CN, 298 K).

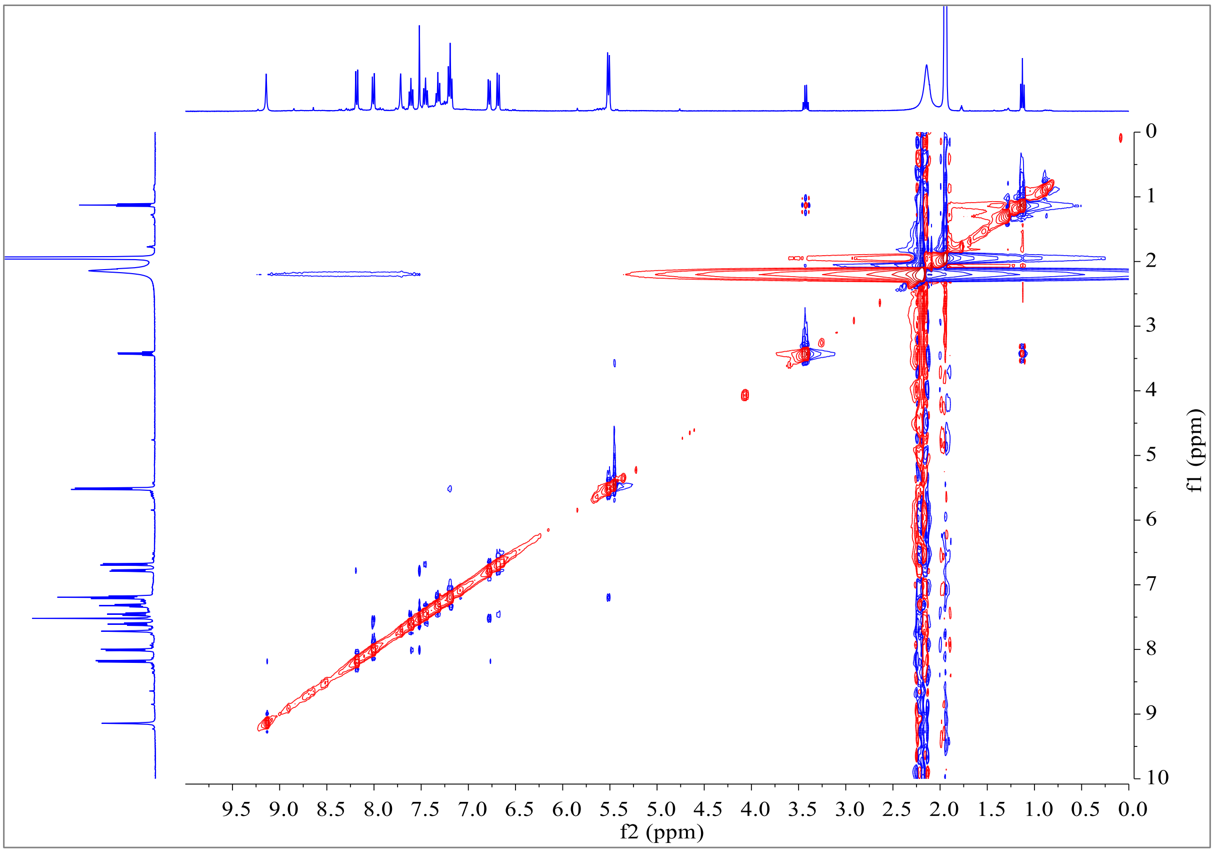

Figure S19. $^{1}H\{^{1}H\}$ NOESY NMR spectrum of *ΛΛ*-Fe-**1** (400 MHz, CD_3CN, 298 K).

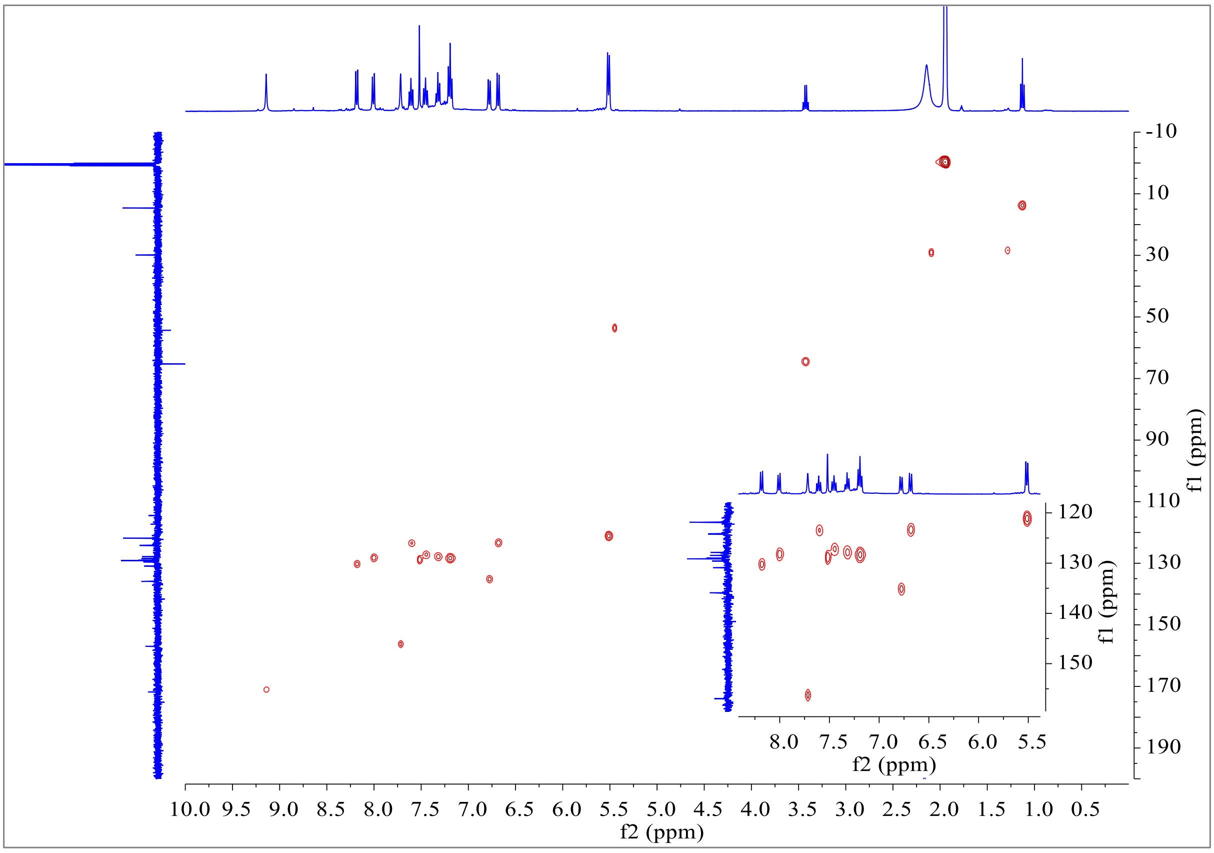

Figure S20. $^{1}H\{^{13}C\}$ HSQC NMR spectrum of *ΛΛ*-Fe-**1** (400 MHz, CD_3CN, 298 K).

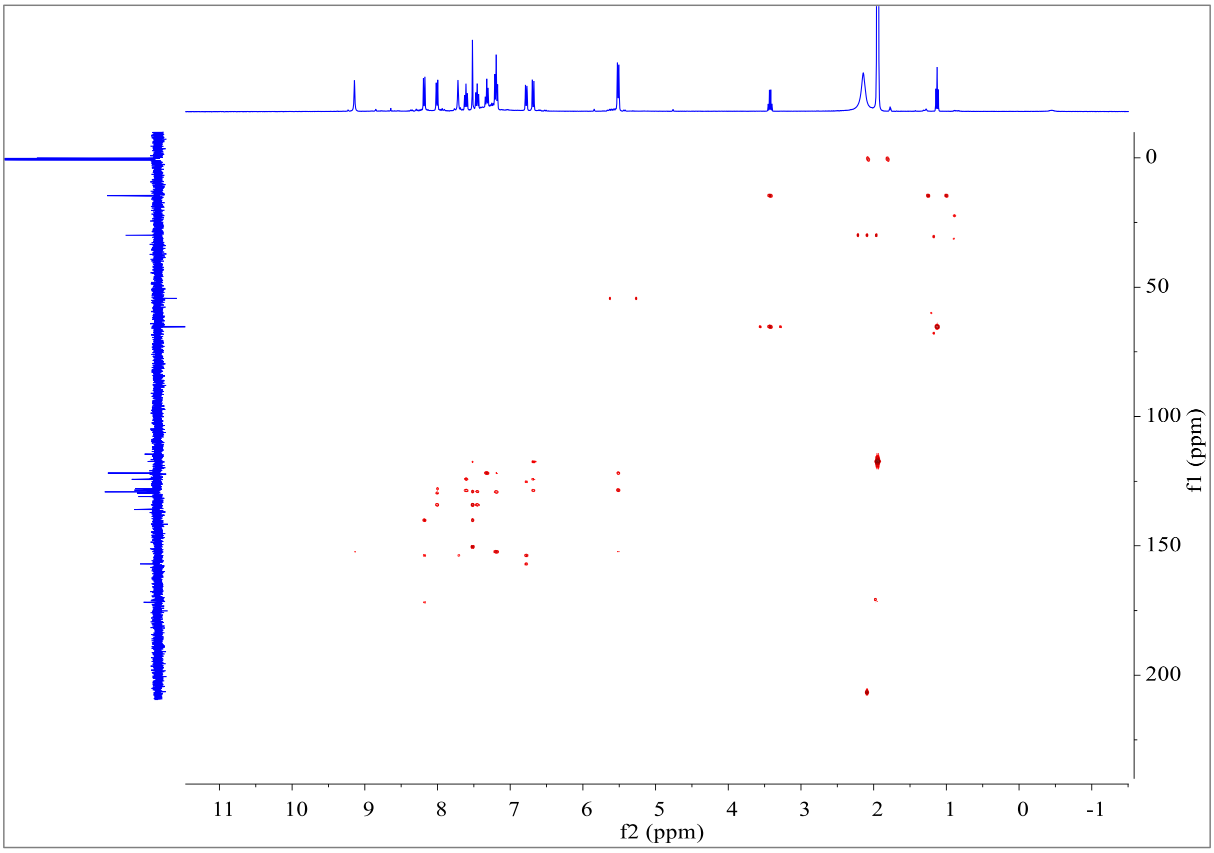

Figure S21. $^{1}H\{^{13}C\}$ HMBC NMR spectrum of *ΛΛ*-Fe-**1** (500 MHz, CD_3CN, 298 K).

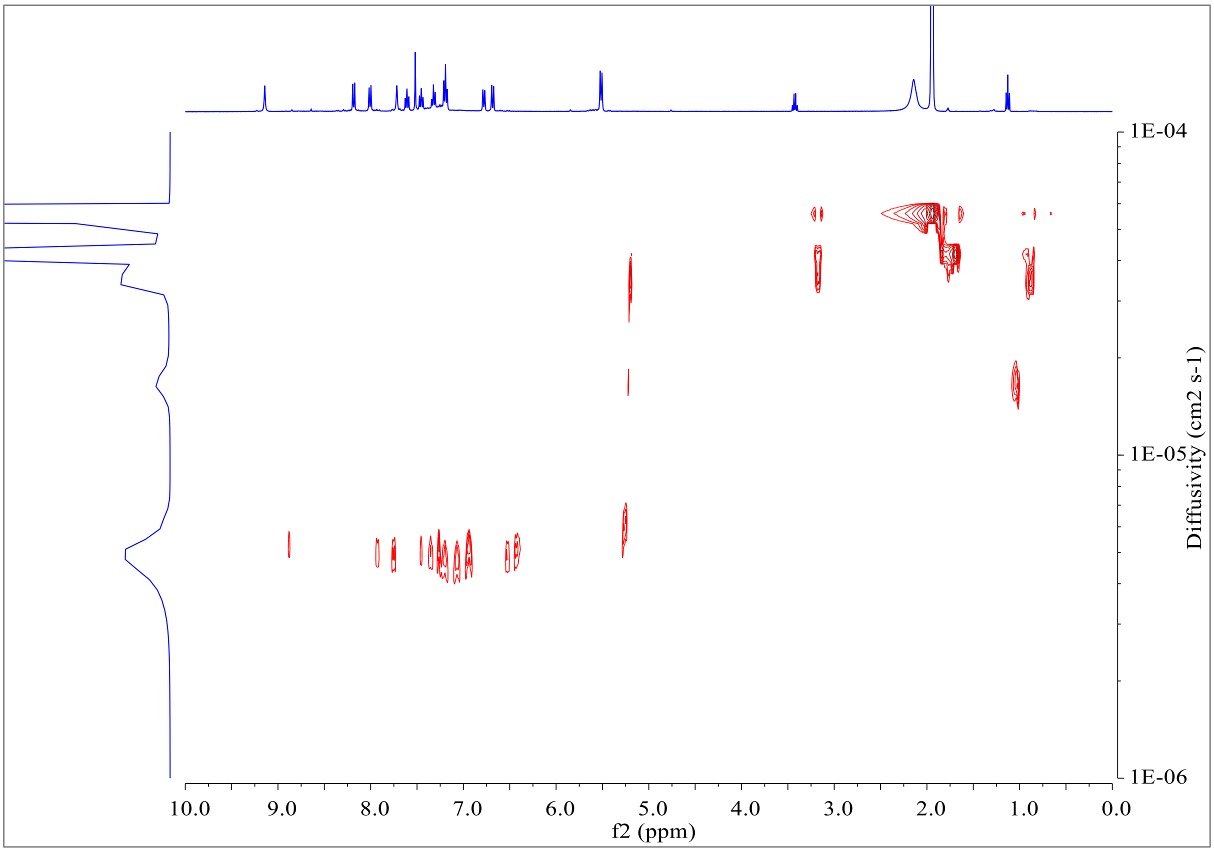

Figure S22. ^{1}H DOSY NMR spectrum of *ΛΛ*-Fe-**1** (400 MHz, CD_3CN, 298 K).

3.1.3 with Co(II) salt

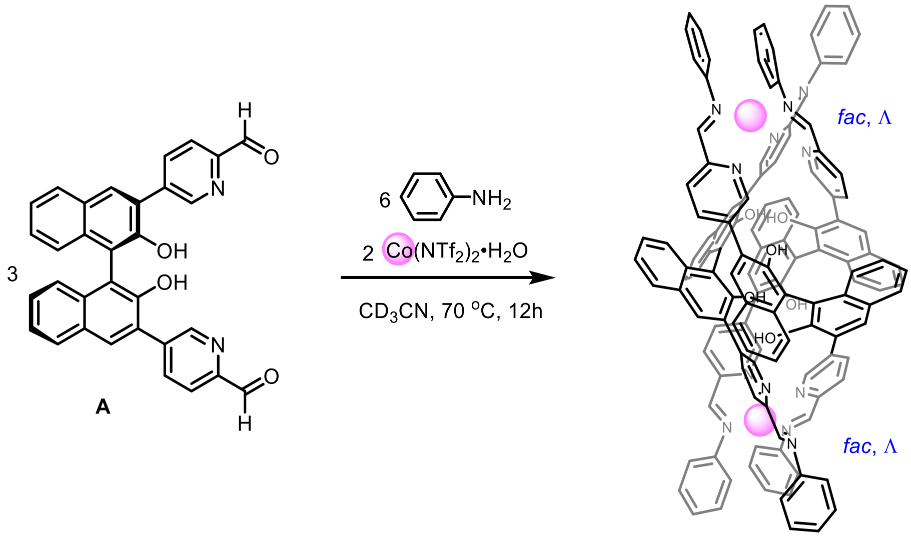

ΛΛ-Co-**1**

Subcomponent **A** (1.9 mg, 3.8 μmol, 1.5 equiv) was added to CD_3CN (0.6 mL) together with $Co(NTf_2)_2 \cdot H_2O$ (1.6 mg, 2.5 μmol, 1.0 equiv) and aniline (0.7 mg, 7.5 μmol, 3.0 equiv). The reaction mixture was stirred at 70 °C for 1h. After cooling to room temperature, the solvent was evaporated and diethyl ether was then added. The residue resuspended and then centrifuged and the diethyl ether decanted. This was repeated three times with fresh diethyl ether. The residue was then dried in vacuo to afford the desired product (*ΛΛ*-Co-**1**) as a pale yellow solid (2.9 mg, 73% yield).

Characterization of ***ΛΛ*****-Co-1:**

^{1}H NMR (400 MHz, CD_3CN, 298 K) δ (ppm) = 1H NMR (400 MHz, CD_3CN) δ 253.0, 68.8, 8.2, 7.5, 7.2, 5.9, 3.0, 1.3, 0.8, -13.3, -22.1, -38.0.

ESI-MS *m/z* 515.0 $[M]^{4+}$.

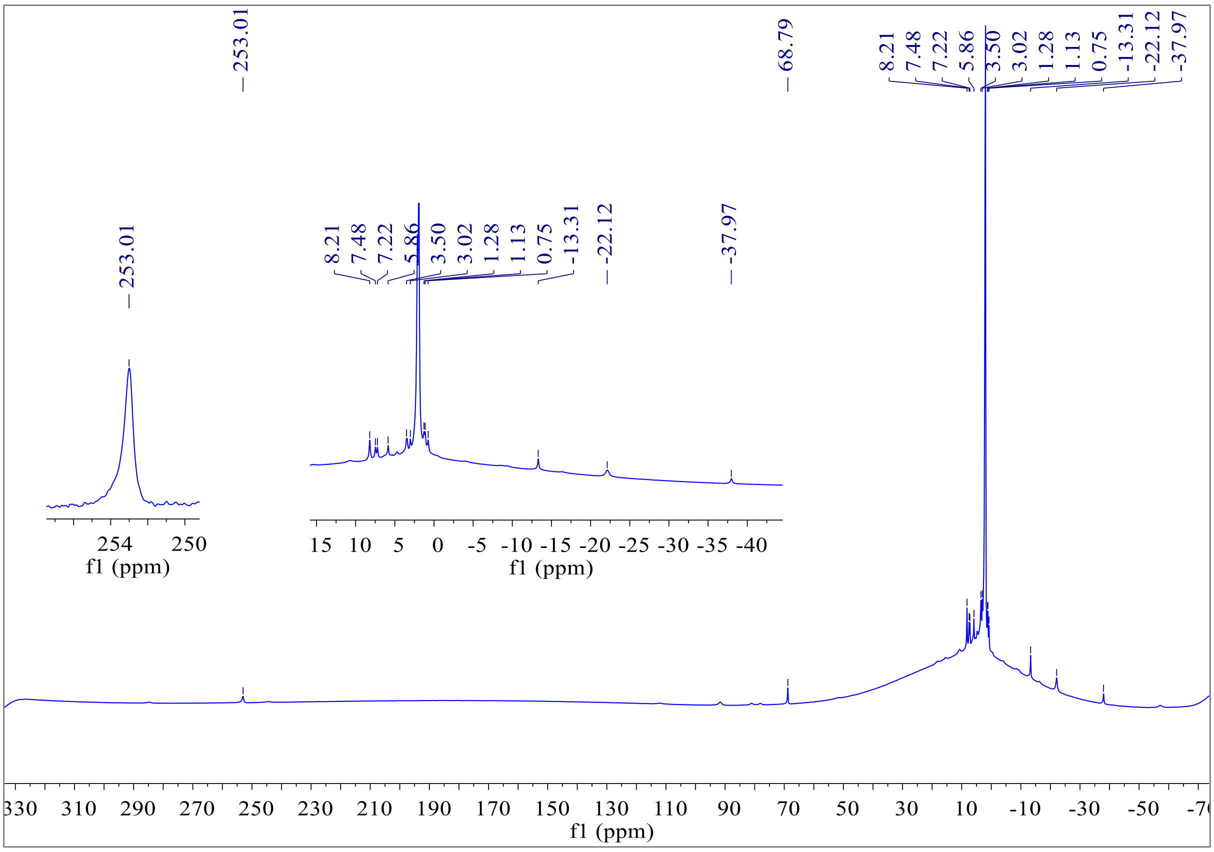

Figure S23. ^{1}H NMR spectrum of *ΛΛ*-Co-**1** (400 MHz, CD_3CN, 298 K).

3.2 Mixed subcomponents

3.2.1 using subcomponents A and C with Fe(II) salt

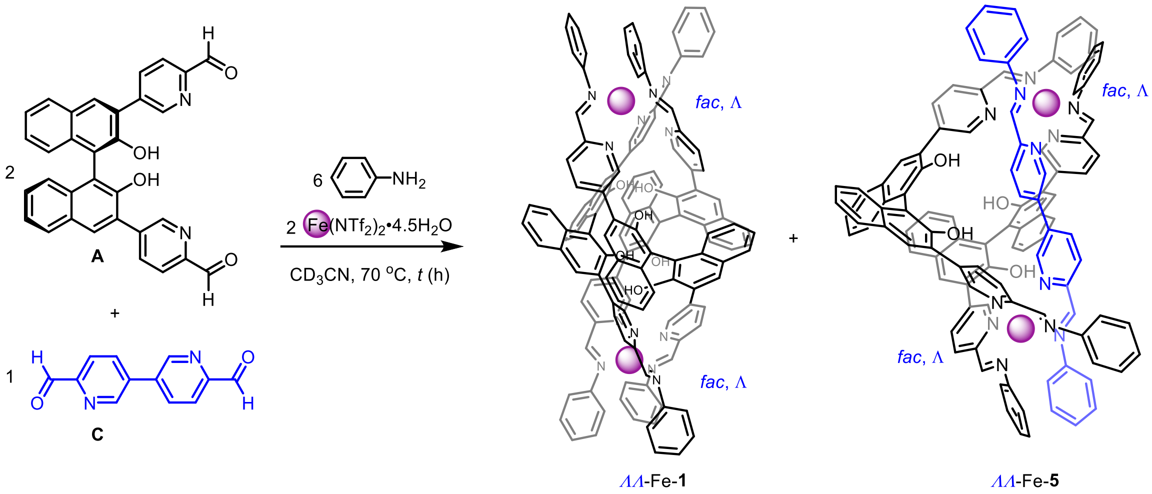

Subcomponents **A** (1.2 mg, 2.5 μmol, 1.0 equiv) and **C** (0.3 mg, 1.25 μmol, 0.5 equiv) were added to CD_3CN (0.6 mL) together with $Fe(NTf_2)_2{\cdot}4.5H_2O$ (1.7 mg, 2.5 μmol, 1.0 equiv) and aniline (0.7 mg, 7.5 μmol, 3.0 equiv). The reaction mixture was stirred at 70 °C and monitored by ^1H NMR. As shown in Figure S24, *ΛΛ*-Fe-**1** (blue square) and a new species (red triangle) appeared at δ = 5.96 (d), 5.44 (d), 5.13 (d) and 5.07 (d) ppm were formed after 1 hour. The structure of this new formed species was futher confirmed to be the *ΛΛ*-Fe-**5**, which assembled from two equivalents of **A** and one equivalent of **C** at room temperature (see below). Interestingly, as the reaction proceeded, *ΛΛ*-Fe-**5** gradually converted to *ΛΛ*-Fe-**1**. After 96 hours, there was only a small amout of *ΛΛ*-Fe-**5** left in the reaction mixture. After treatment of the resulting reaction mixture in a microwave reactor at 120 ºC for 2.5 hours, *ΛΛ*-Fe-**5** almost fully converted to *ΛΛ*-Fe-**1**. (Note: Tetrahedron Fe-**3** assembled from **C** was formed as a PF_6^- inclusion complex $PF_6^- \subset$ Fe-**3** together with *ΛΛ*-Fe-**1** when performing the reaction with Me_4NBF_6 as a PF_6^- internal template anion, see Figure S35).

ΛΛ-Fe-**5** could be easily isolated by performing the above self-assembly at room temperature for 12h. Then the solvent was evaporated and diethyl ether was then added. The residue resuspended and then centrifuged and the diethyl ether decanted. This was repeated three times with fresh diethyl ether. The residue was then dried in vacuo to afford the desired product (*ΛΛ*-Fe-**5**) as a purple solid (2.5 mg, 69% yield).

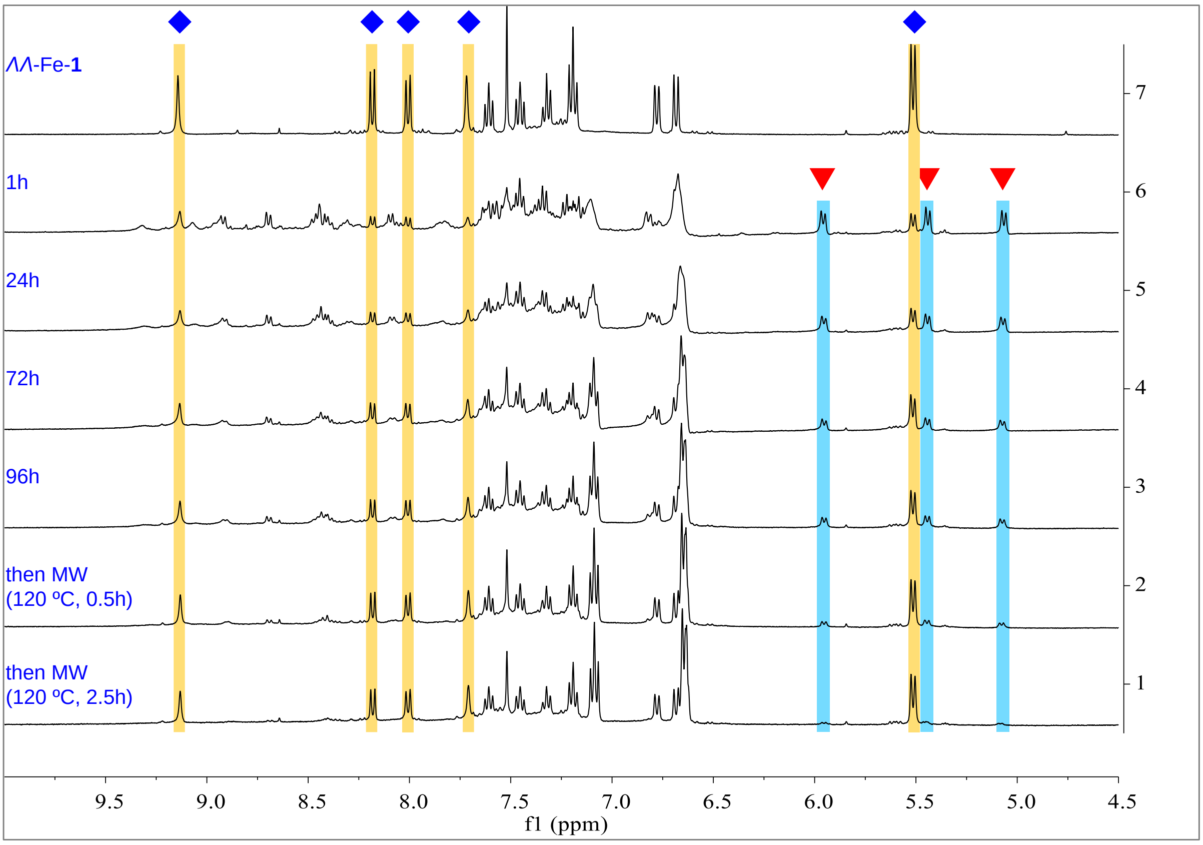

Figure S24. Crude ^{1}H NMR spectrum of self-assembly of **A**, **C** and aniline with $Fe(NTf_2)_2$ (400 MHz, CD_3CN, 298 K) (blue square: *ΛΛ*-Fe-**1**; red triangle: *ΛΛ*-Fe-**5**).

Characterization of *ΛΛ*-Fe-5:

^{1}H NMR (400 MHz, CD_3CN, 298 K) δ (ppm) = 9.23 (s, 2H), 9.04 (s, 2H), 8.98 (s, 2H), 8.90 – 8.85 (m, 4H), 8.69 (d, *J* = 8.1 Hz, 2H), 8.45 (m, 4H), 8.40 (m, 4H), 8.10 (d, *J* = 8.3 Hz, 2H), 7.71 (s, 4H), 7.58 (m, 8H), 7.49 – 7.43 (m, 8H), 7.34 (m, 8H), 7.21 (s, 4H), 7.15 (d, *J* = 7.7 Hz, 4H), 6.82 (d, *J* = 9.0 Hz, 4H), 6.07 (s, 2H, O*H*), 5.97 (d, *J* = 7.7 Hz, 4H), 5.49 – 5.41 (m, 5H, containg O*H*), 5.06 (d, *J* = 7.9 Hz, 4H).

^{13}C{^{1}H} DEPT-135 (100 MHz, CD_3CN, 298 K) δ (ppm) = 174.9, 174.4, 173.1, 160.1, 155.6, 153.5, 142.1, 139.6, 136.1, 133.5, 133.0, 132.9, 132.7, 131.7, 130.8, 130.5, 130.2, 130.2, 129.9, 129.8, 129.7, 129.7, 129.3, 129.2, 126.3, 125.7, 125.6, 125.3, 123.1, 122.7, 121.8.

ESI-MS *m/z* 441.7 $[M]^{4+}$, 682.3 $[M+NTf_2]^{3+}$, 1163.5 $[M+2NTf_2]^{2+}$.

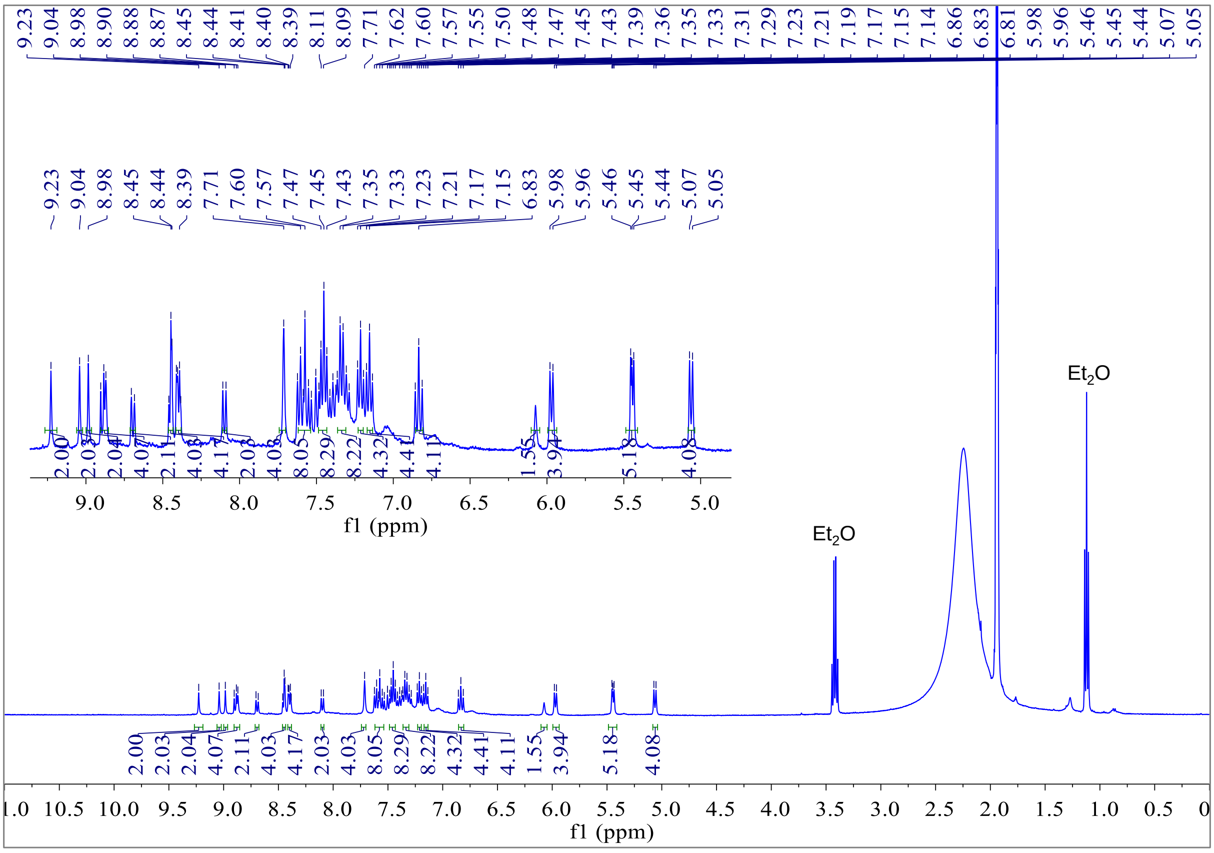

Figure S25. ^{1}H NMR spectrum of *ΛΛ*-Fe-**5** (400 MHz, CD_3CN, 298 K).

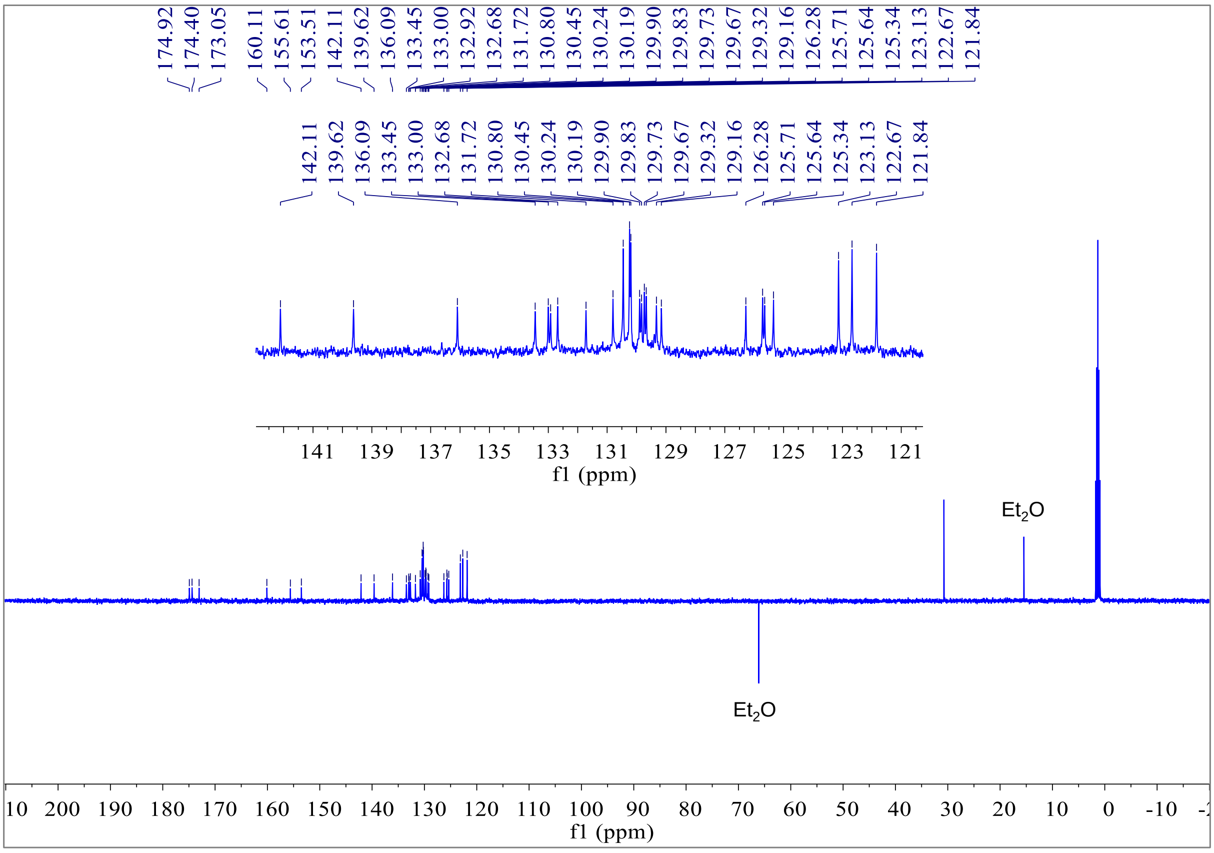

Figure S26. $^{13}C\{^{1}H\}$ DEPT-135 NMR spectrum of *ΛΛ*-Fe-**5** (100 MHz, CD_3CN, 298 K).

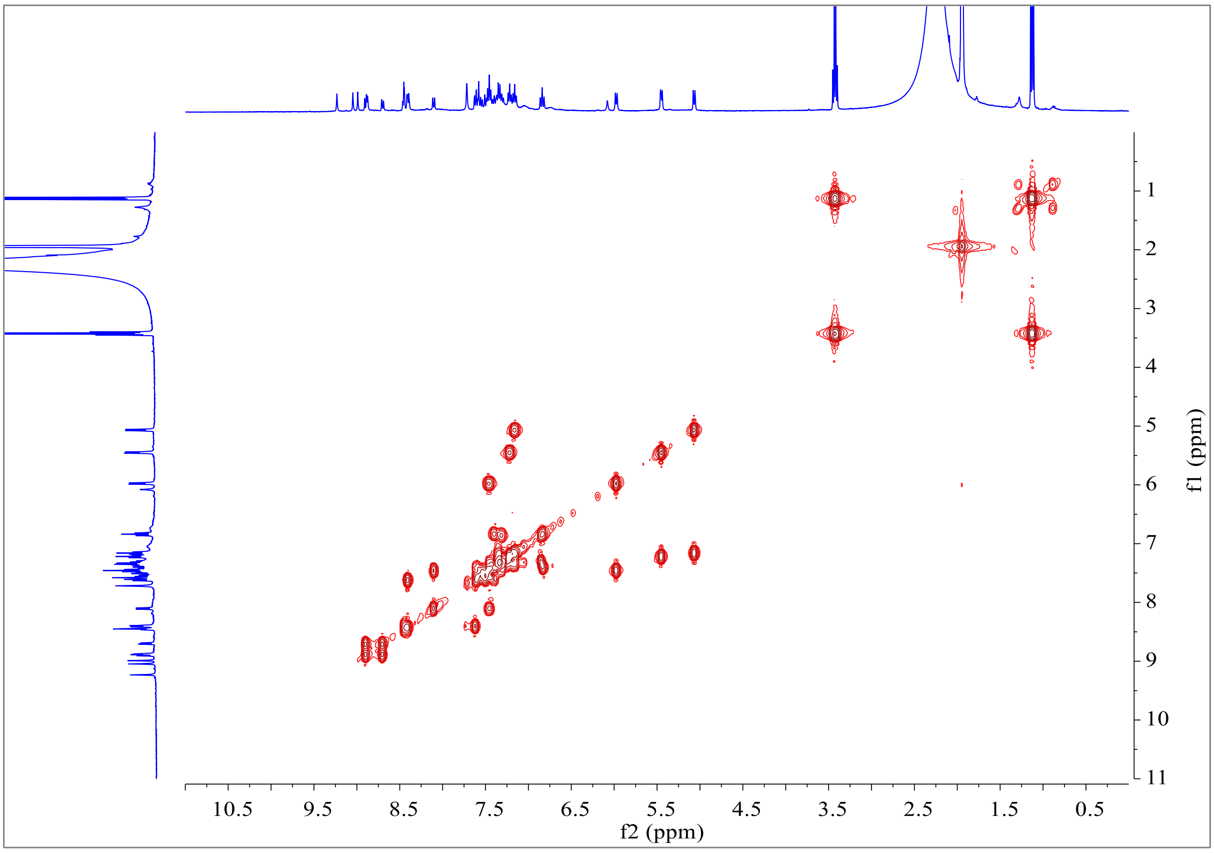

Figure S27. $^{1}H\{^{1}H\}$ COSY NMR spectrum of *ΛΛ*-Fe-**5** (400 MHz, CD_3CN, 298 K).

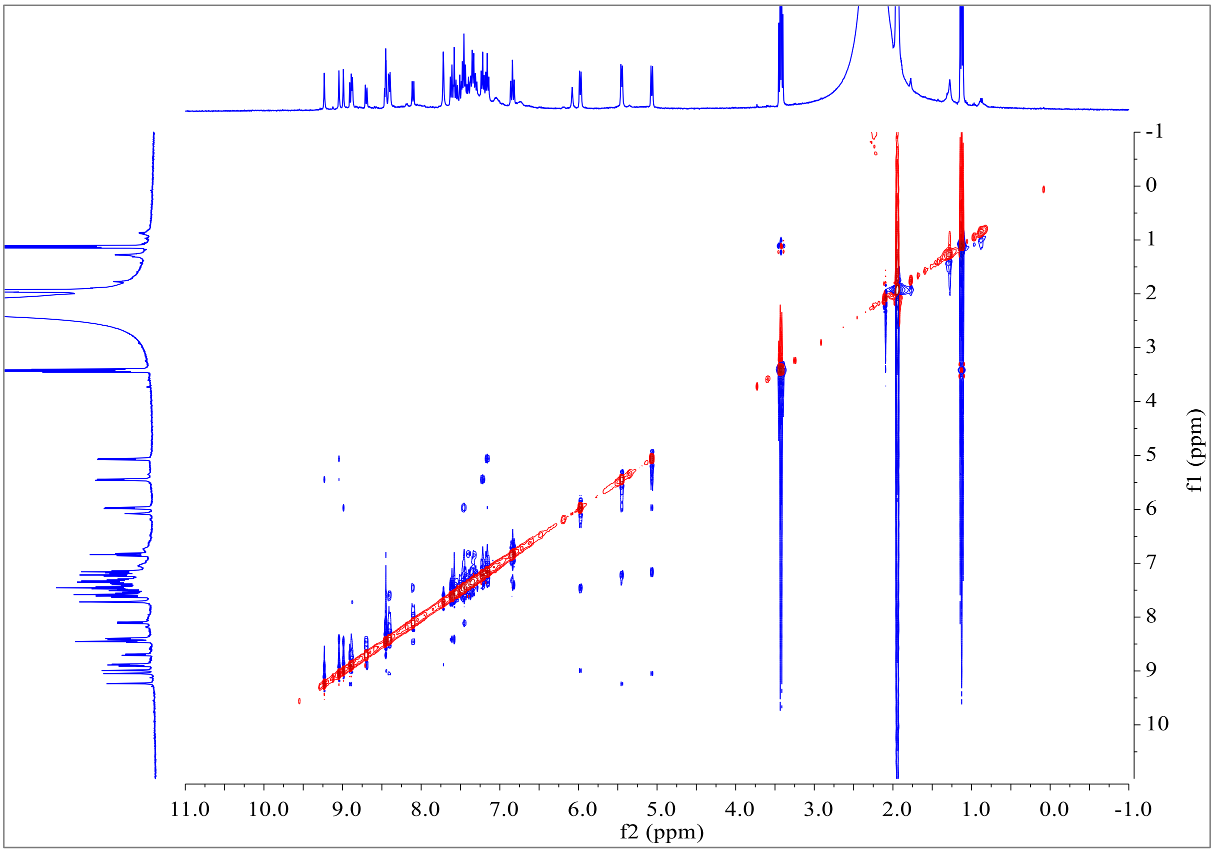

Figure S28. $^{1}H\{^{1}H\}$ NOESY NMR spectrum of *ΛΛ*-Fe-**5** (400 MHz, CD_3CN, 298 K).

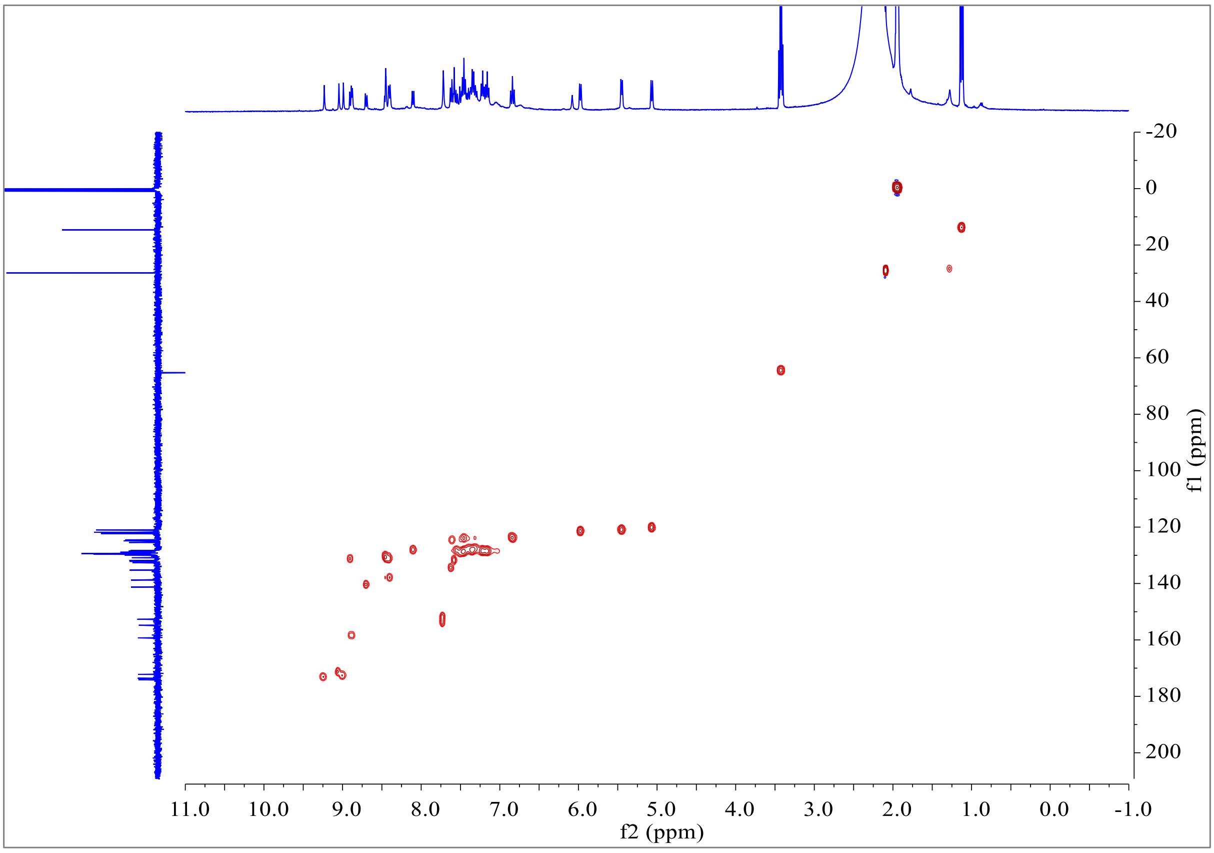

Figure S29. $^{1}H\{^{13}C\}$ HSQC NMR spectrum of *ΛΛ*-Fe-**5** (400 MHz, CD_3CN, 298 K).

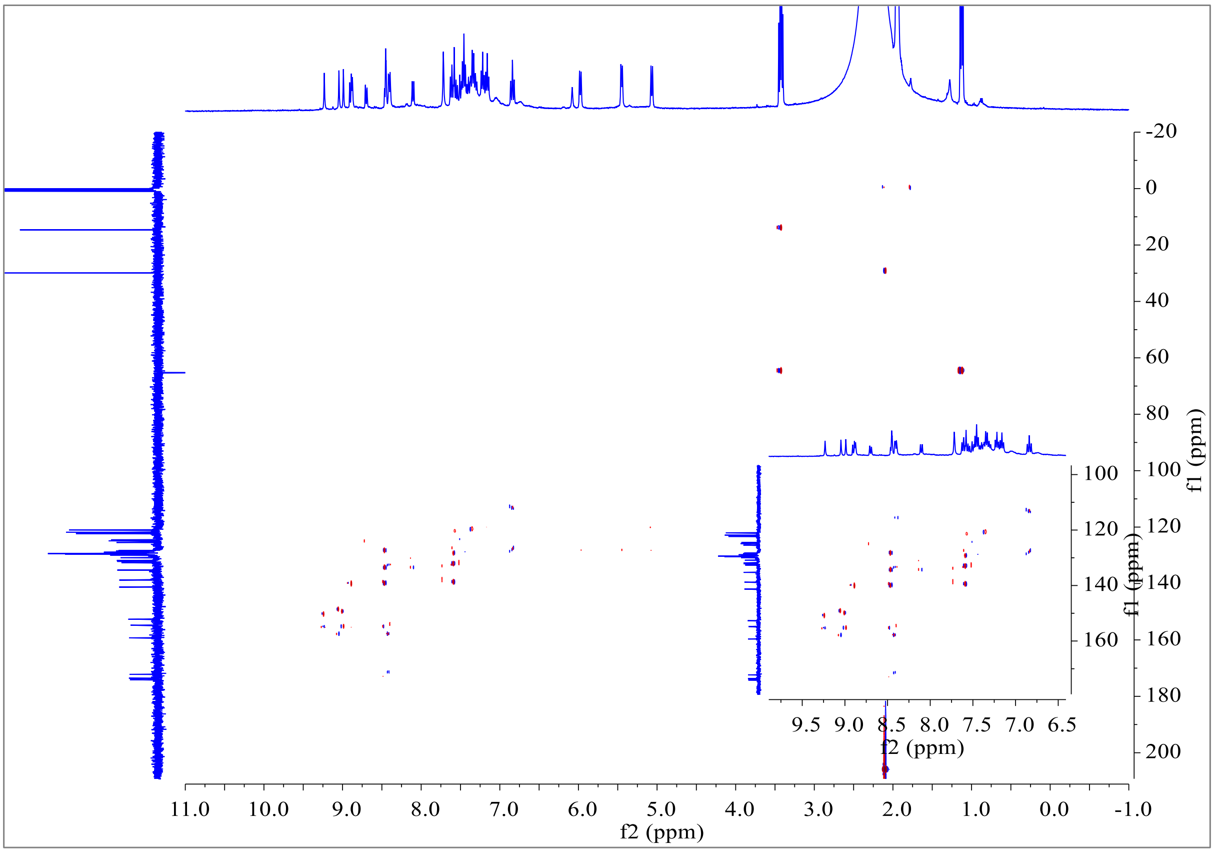

Figure S30. $^{1}H\{^{13}C\}$ HMBC NMR spectrum of *ΛΛ*-Fe-**5** (400 MHz, CD_3CN, 298 K).

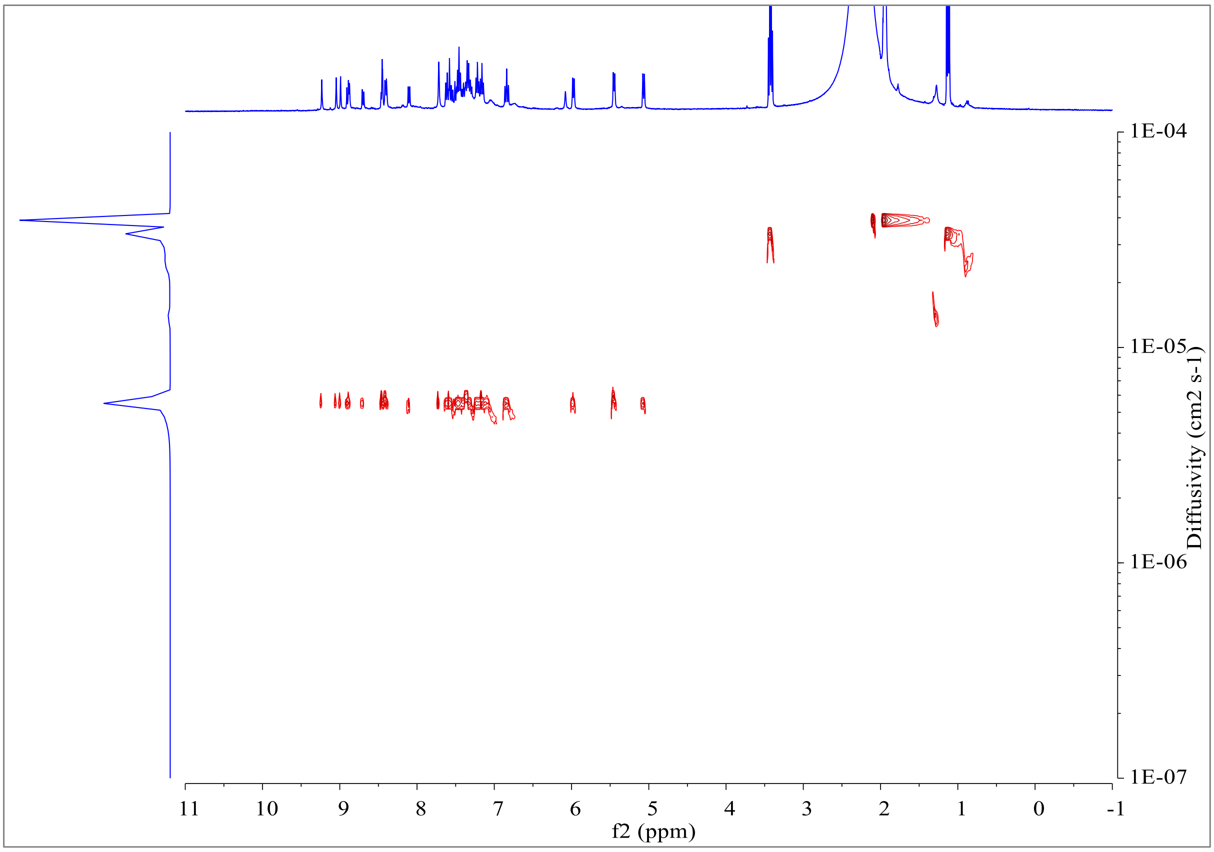

Figure S31. ^{1}H DOSY NMR spectrum of *ΛΛ*-Fe-**5** (400 MHz, CD_3CN, 298 K).

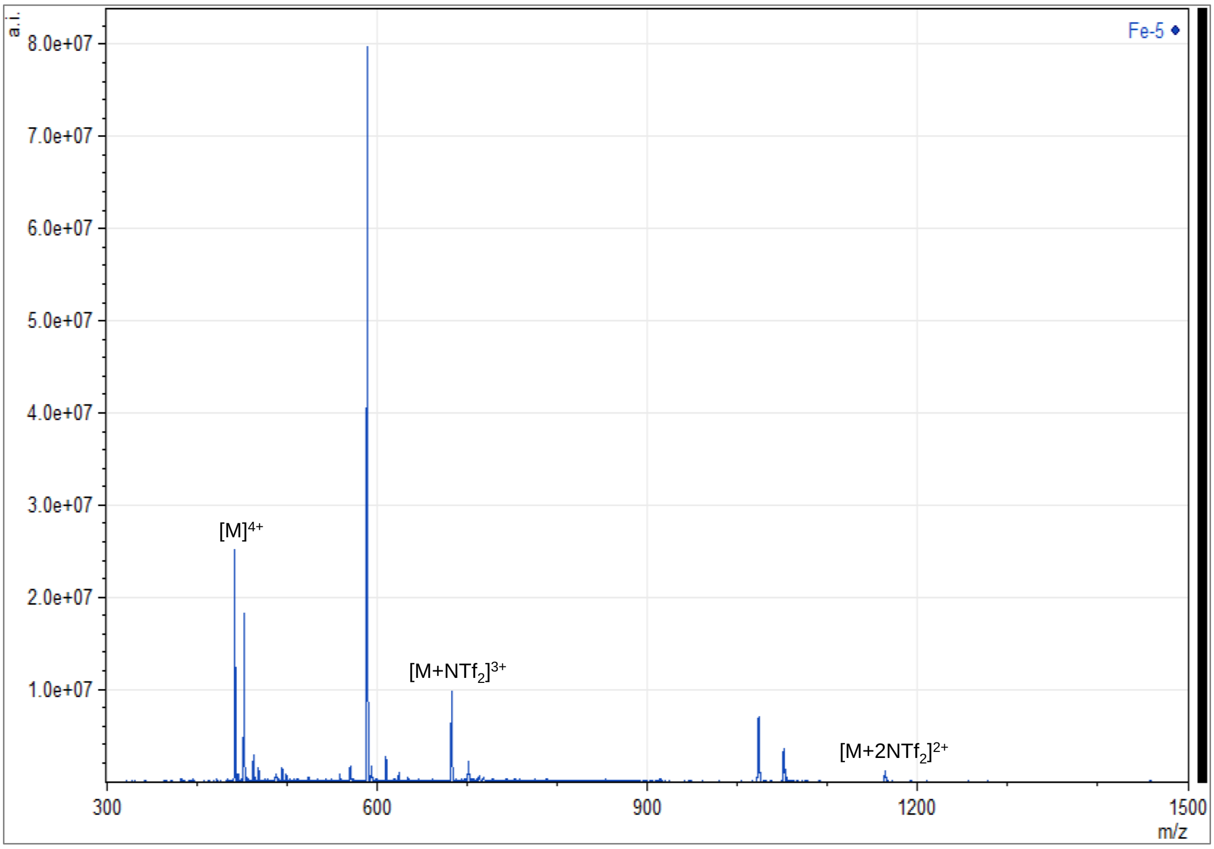

Figure S32. Low-resolution ESI-mass spectrum of *ΛΛ*-Fe-**5**.

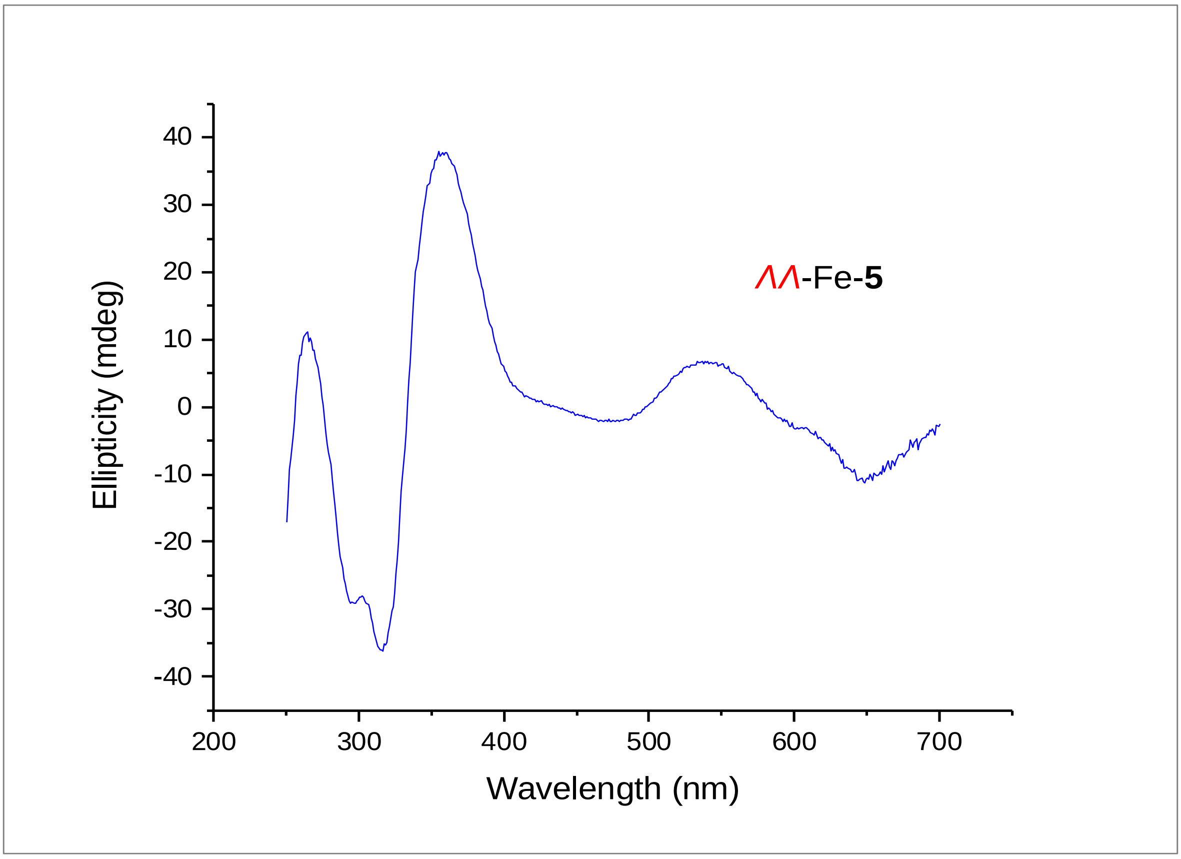

Figure S33. Circular dichroism (CD) spectrum of *Λ*Λ-Fe-**5**.

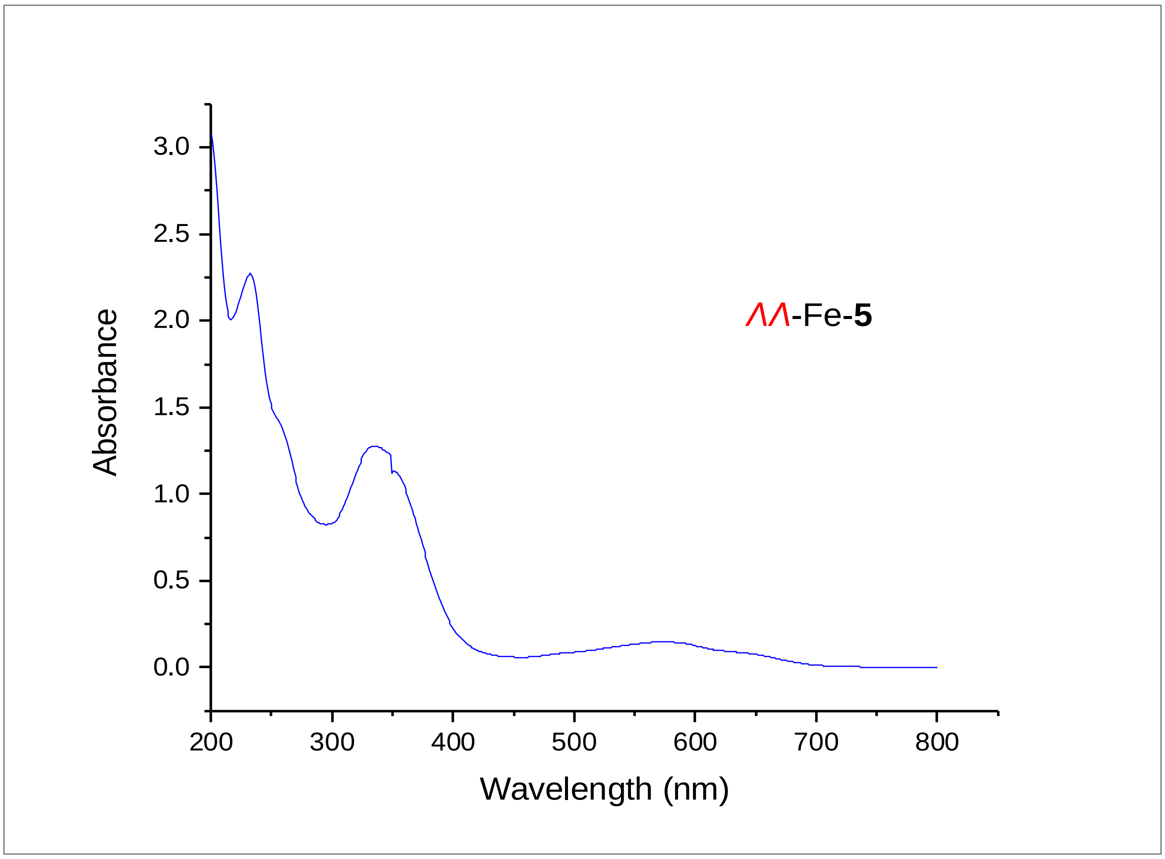

Figure S34. UV-Vis spectrum of *Λ*Λ-Fe-**5**.

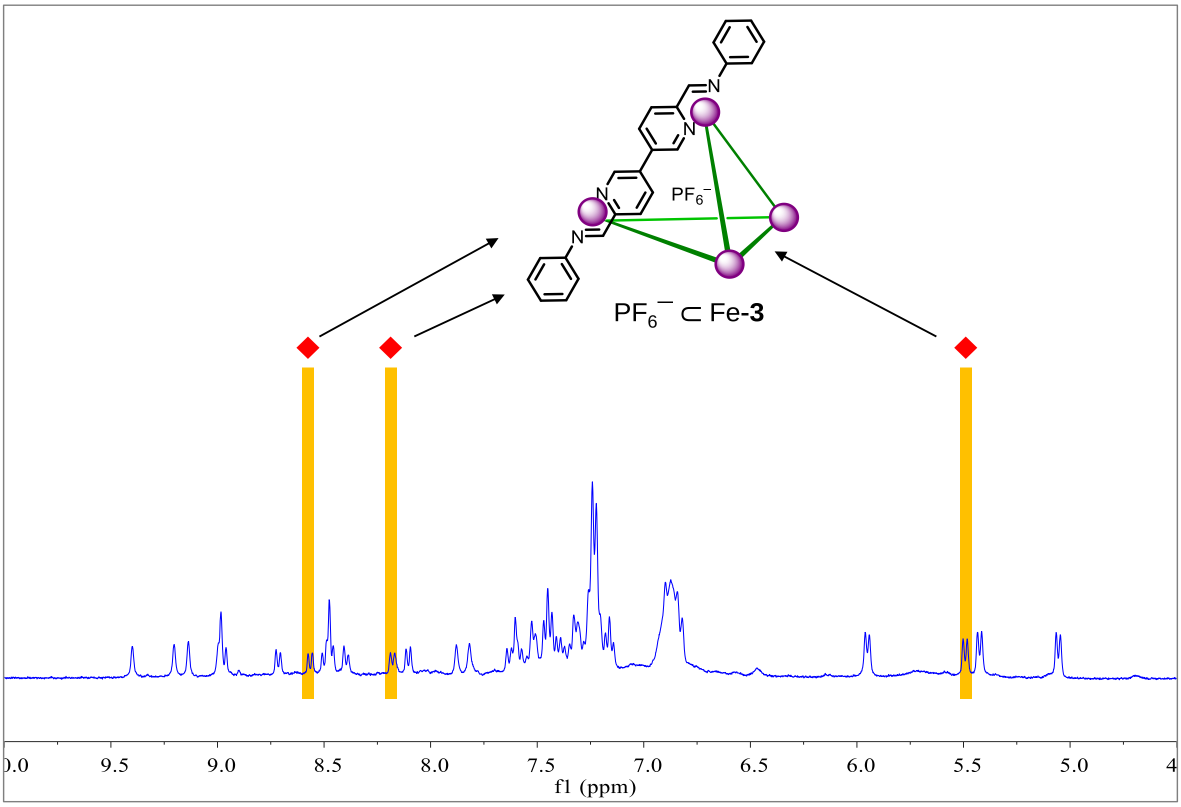

Figure S35. ^{1}H NMR spectrum of self-assebmly of **A**, **C** and $Fe(NTf_2)_2$ with excess Me_4NPF_6 at 70 °C for 24h (400 MHz, CD_3CN, 298 K).

3.2.2 using subcomponents A and C with Zn(II) salt

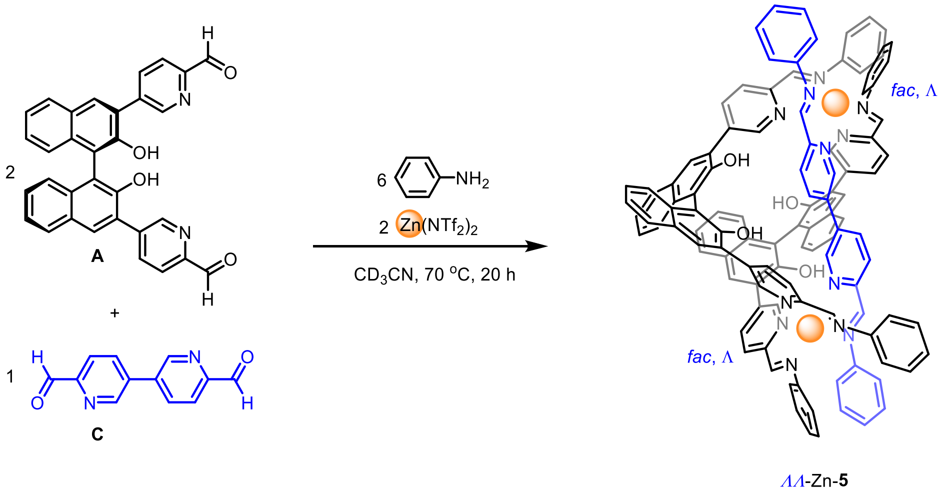

Subcomponents **A** (1.2 mg, 2.5 μmol, 1.0 equiv) and **C** (0.3 mg, 1.25 μmol, 0.5 equiv) were added to CD_3CN (0.6 mL) together with $Zn(NTf_2)_2$ (1.6 mg, 2.5 μmol, 1.0 equiv) and aniline (0.7 mg, 7.5 μmol, 3.0 equiv). The reaction mixture was stirred at 70 °C for 20h. Then the solvent was evaporated and diethyl ether was then added. The residue was resuspended and then centrifuged and the diethyl ether decanted. This was repeated three times with fresh diethyl ether. The residue was then dried in vacuo to afford the desired product (*ΛΛ*-Zn-**5**) as a pale yellow solid (2.6 mg, 72% yield).

Characterization of *ΛΛ*-Zn-5:

^{1}H NMR (500 MHz, CD_3CN, 298 K) δ (ppm) = 9.46 (d, *J* = 2.0 Hz, 2H), 8.98 (m, 2H), 8.74 (s, 2H), 8.60 (m, 2H), 8.53 (s, 2H), 8.52 (s, 2H), 8.44 (m, 2H), 8.34 (s, 2H), 8.15 – 8.11 (m, 4H), 8.05 (d, *J* = 2.0 Hz, 2H), 7.98 (s, 2H), 7.92 (m, 2H), 7.80 (d, *J* = 7.7 Hz, 2H), 7.65 (s, 2H), 7.58 (d, *J* = 3.2 Hz, 4H), 7.48 (m, 4H), 7.40 (m, 4H), 7.36 – 7.32 (m, 4H), 7.29 – 7.27 (m, 2H), 7.26 – 7.22 (m, 4H), 7.18 – 7.14 (m, 8H), 6.88 (m, 6H), 6.83 (m, 2H), 6.65 (s, 2H), 6.18 – 6.11 (m, 4H), 5.84 – 5.80 (m, 4H).

^{13}C NMR (125 MHz, CD_3CN, 298 K) δ (ppm) = 165.8, 165.2, 162.3, 152.8, 150.3, 150.0, 148.5, 148.3, 148.2, 147.8, 146.8, 145.9, 145.8, 144.5, 141.9, 141.6, 140.8, 139.6, 137.1, 136.4, 135.4, 133.7, 133.5, 133.1, 132.7, 131.1, 130.6, 130.5, 130.4, 130.1, 130.0, 129.9, 129.8, 129.5, 129.4, 129.0, 126.5, 126.2, 126.2, 126.1, 125.9, 122.9, 122.2, 122.2, 122.1, 119.6, 114.8, 114.7.

ESI-MS *m/z* 446.3 $[M]^{4+}$, 689.0 $[M+NTf_2]^{3+}$, 1172.5 $[M+2NTf_2]^{2+}$.

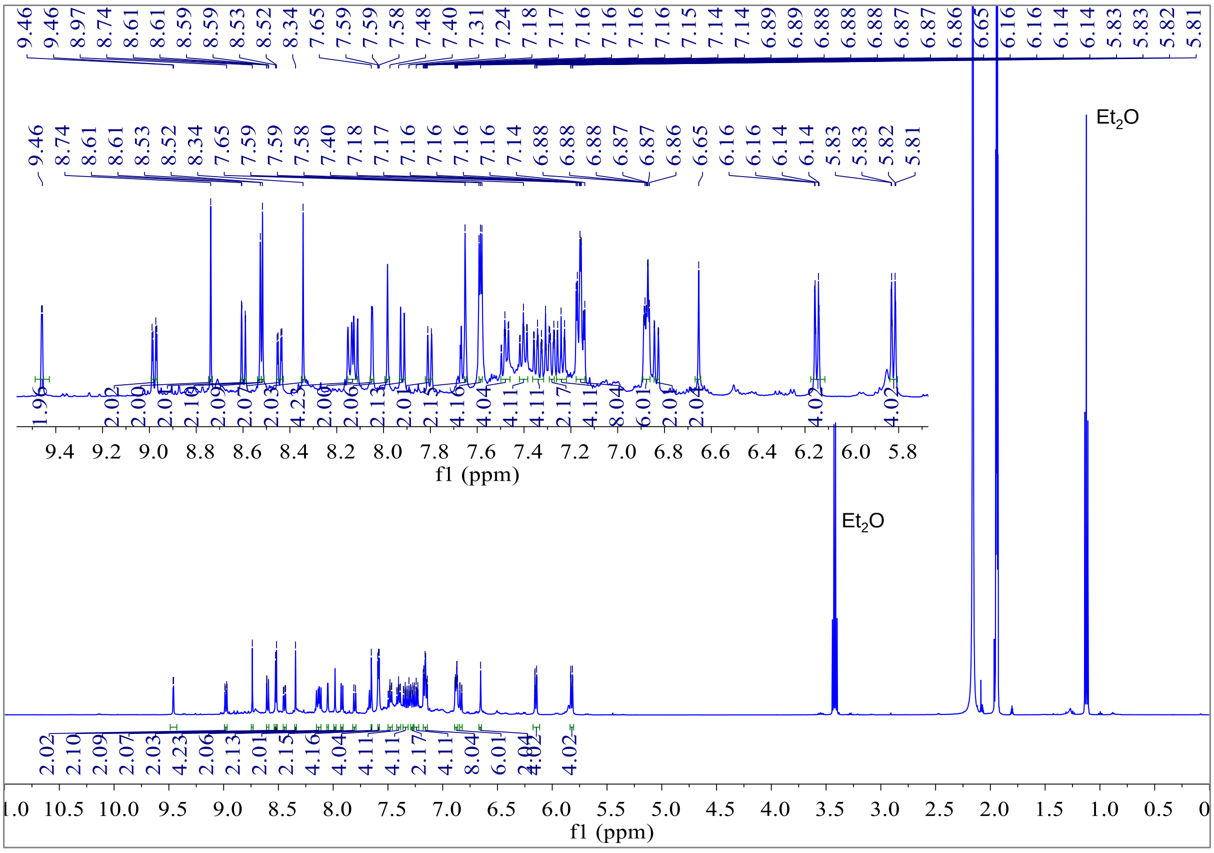

Figure S36. ^{1}H NMR spectrum of *ΛΛ*-Zn-**5** (500 MHz, CD_3CN, 298 K).

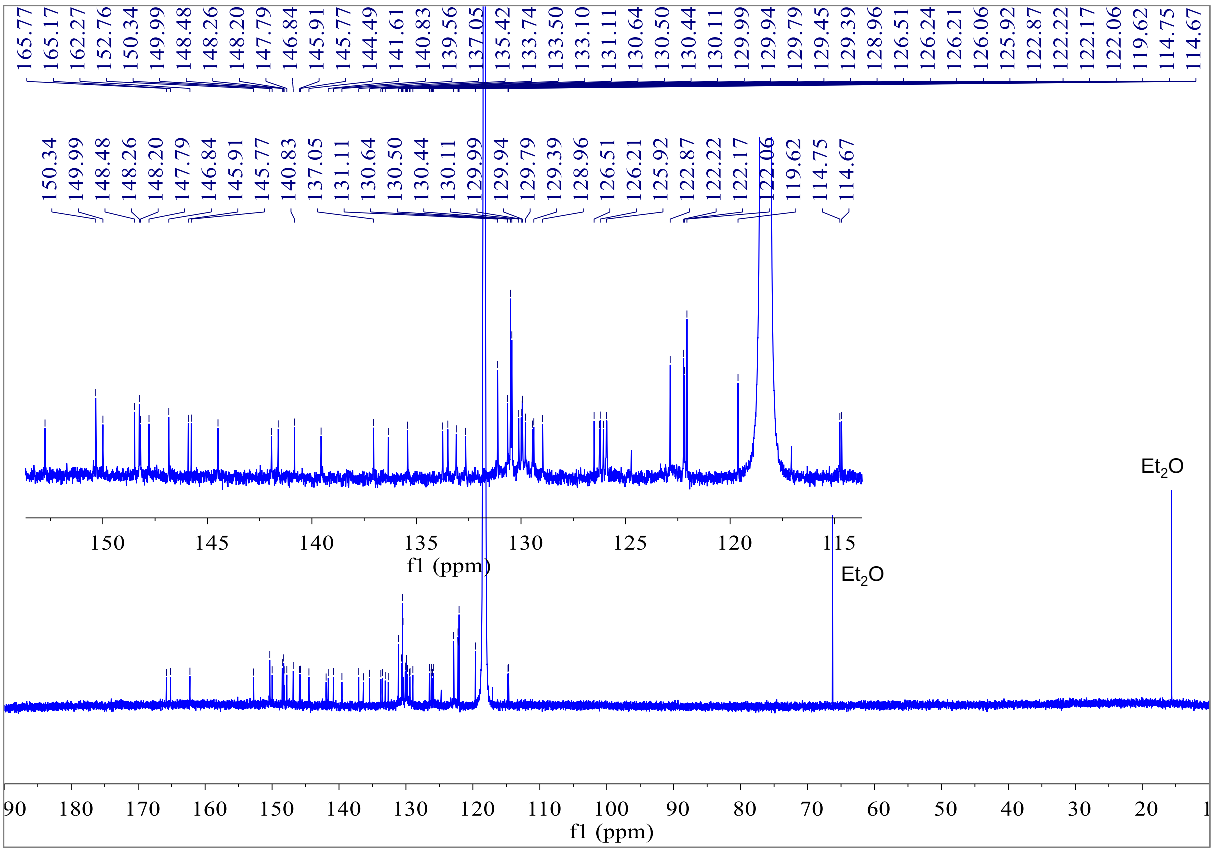

Figure S37. ^{13}C NMR spectrum of *ΛΛ*-Zn-**5** (125 MHz, CD_3CN, 298 K).

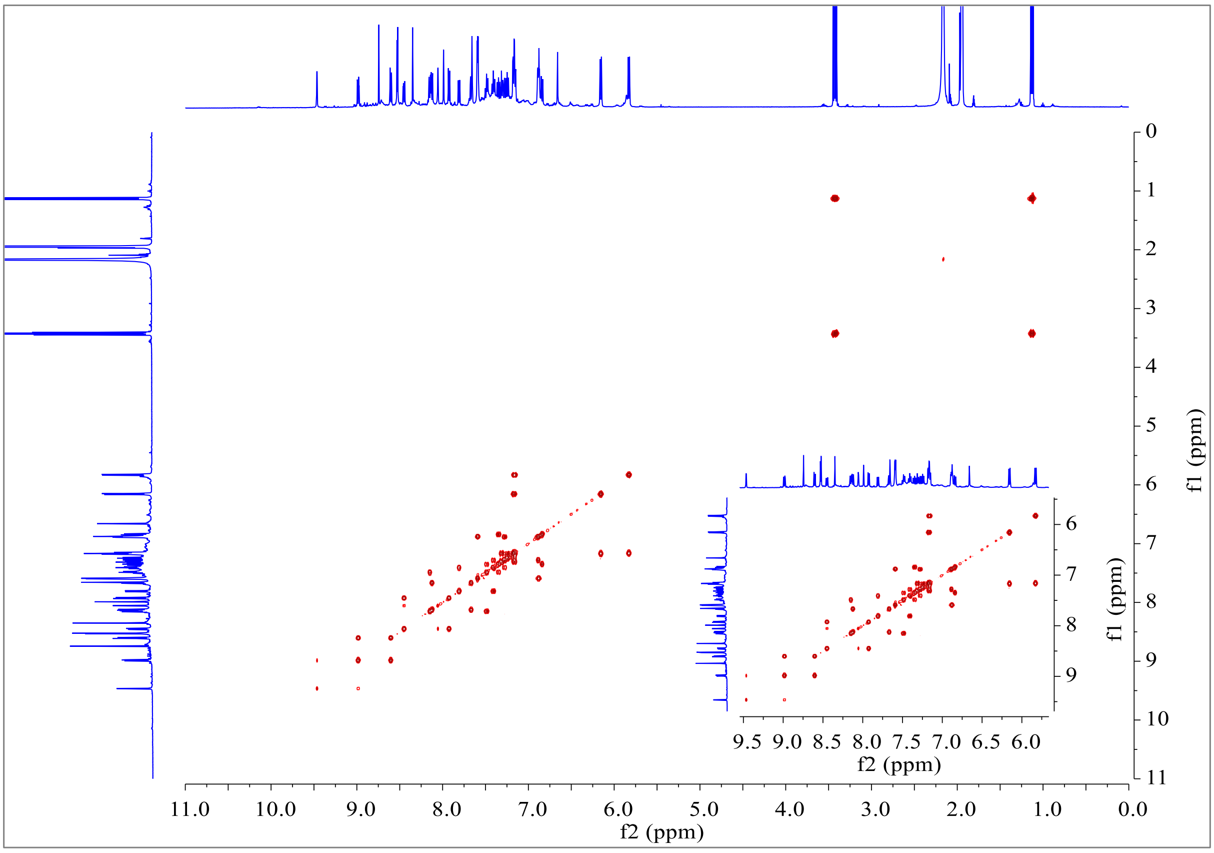

Figure S38. $^{1}H\{^{1}H\}$ COSY NMR spectrum of *ΛΛ*-Zn-**5** (500 MHz, CD_3CN, 298 K).

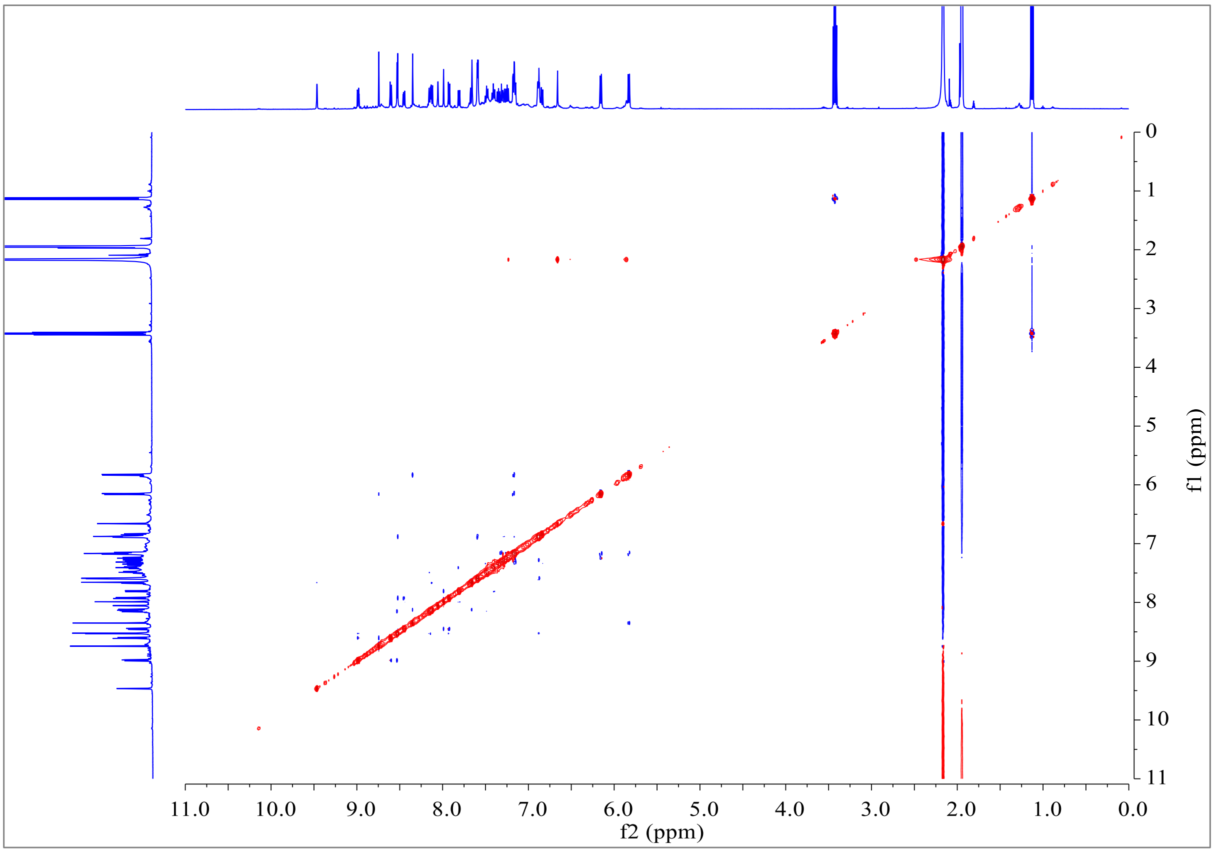

Figure S39. $^{1}H\{^{1}H\}$ NOESY NMR spectrum of *ΛΛ*-Zn-**5** (500 MHz, CD_3CN, 298 K).

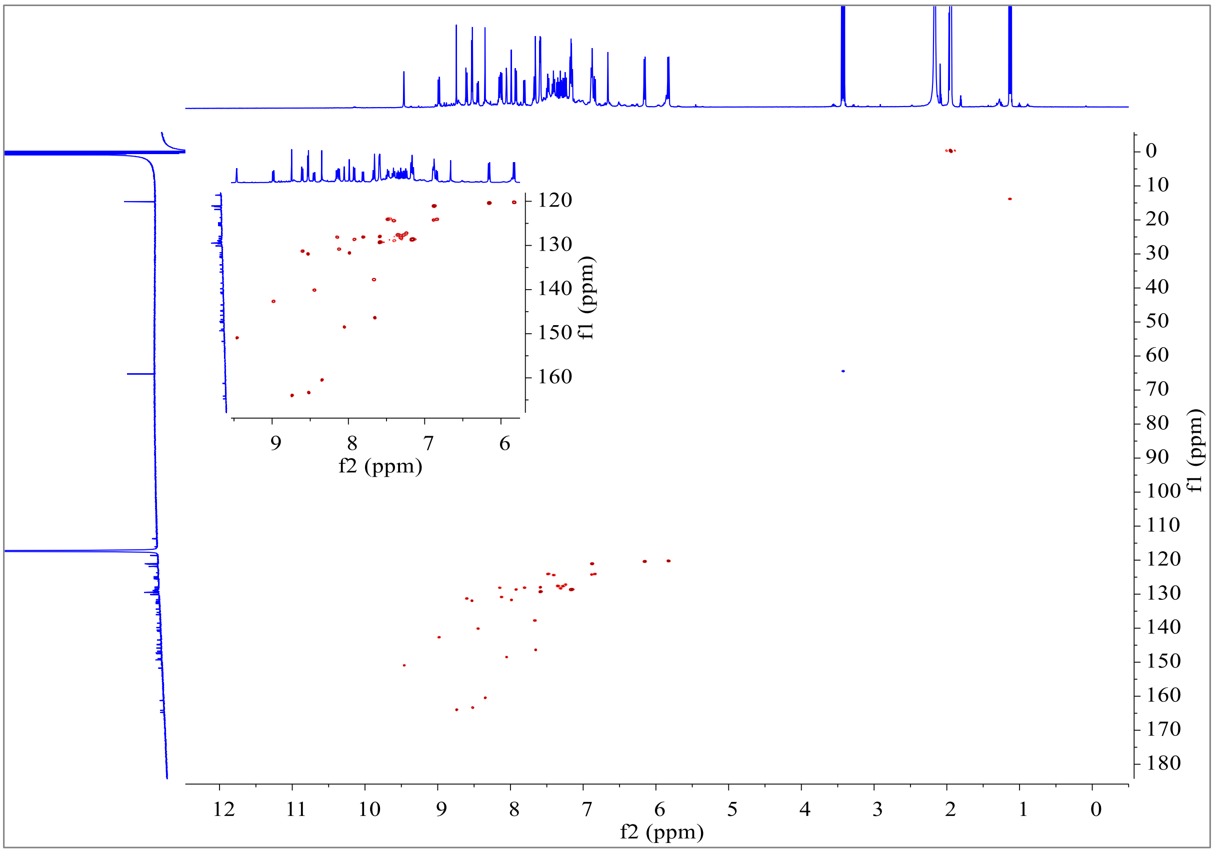

Figure S40. $^{1}H\{^{13}C\}$ HSQC NMR spectrum of *ΛΛ*-Zn-**5** (500 MHz, CD_3CN, 298 K).

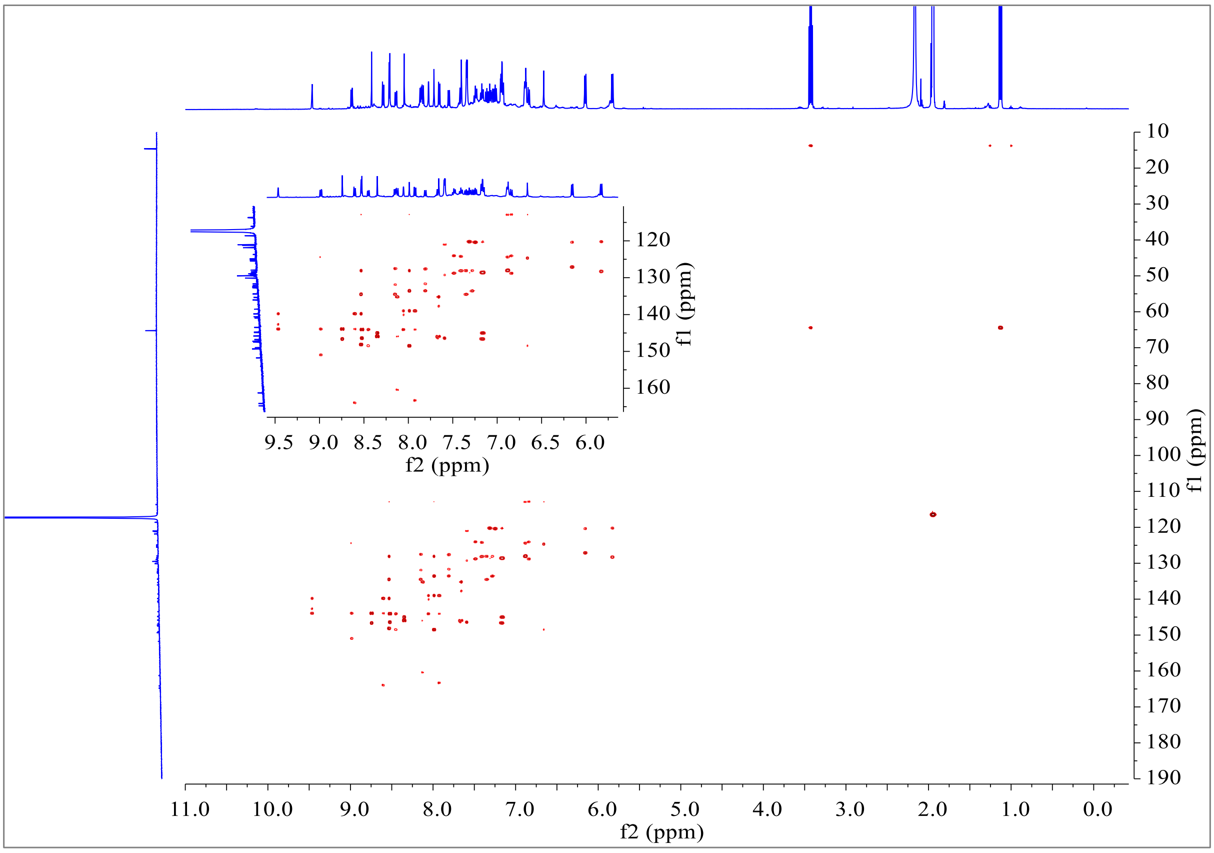

Figure S41. $^{1}H\{^{13}C\}$ HMBC NMR spectrum of *ΛΛ*-Zn-**5** (500 MHz, CD_3CN, 298 K).

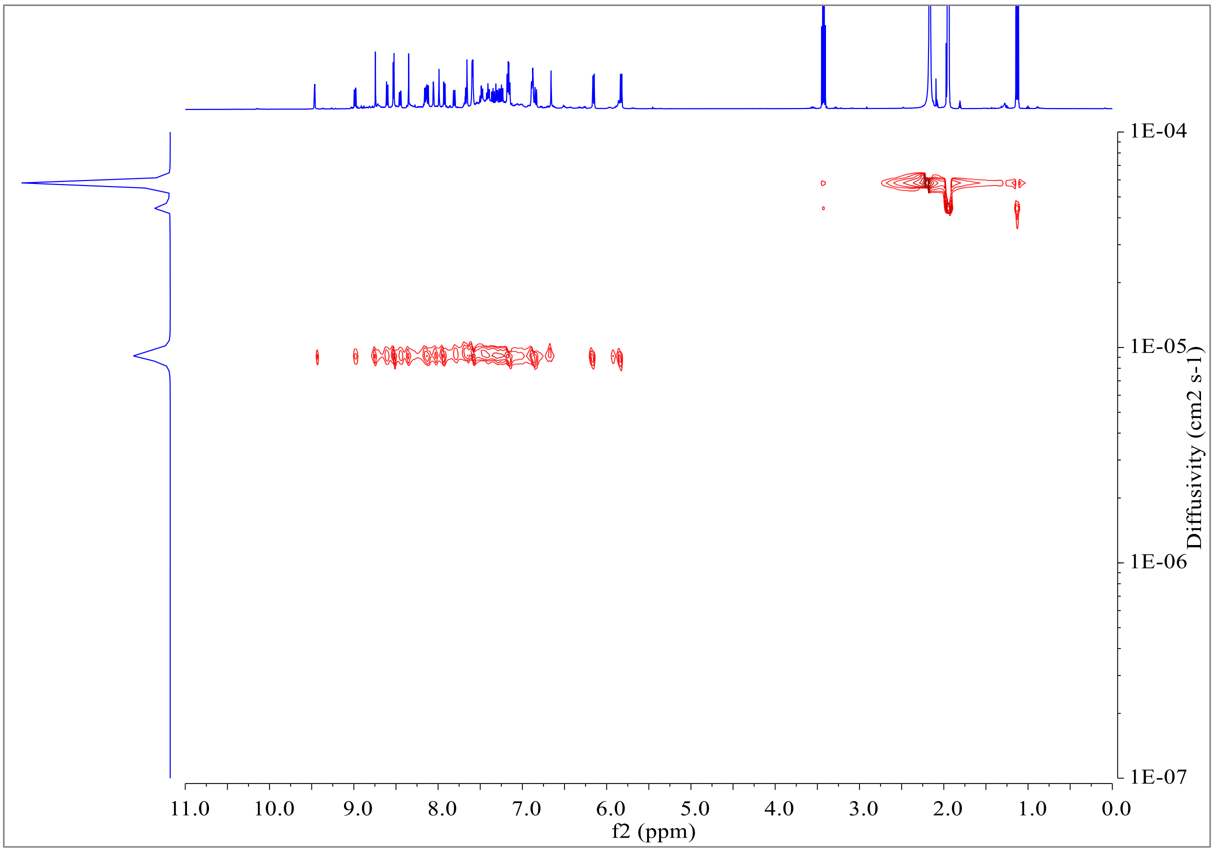

Figure S42. ^{1}H DOSY NMR spectrum of *ΛΛ*-Zn-**5** (400 MHz, CD_3CN, 298 K).

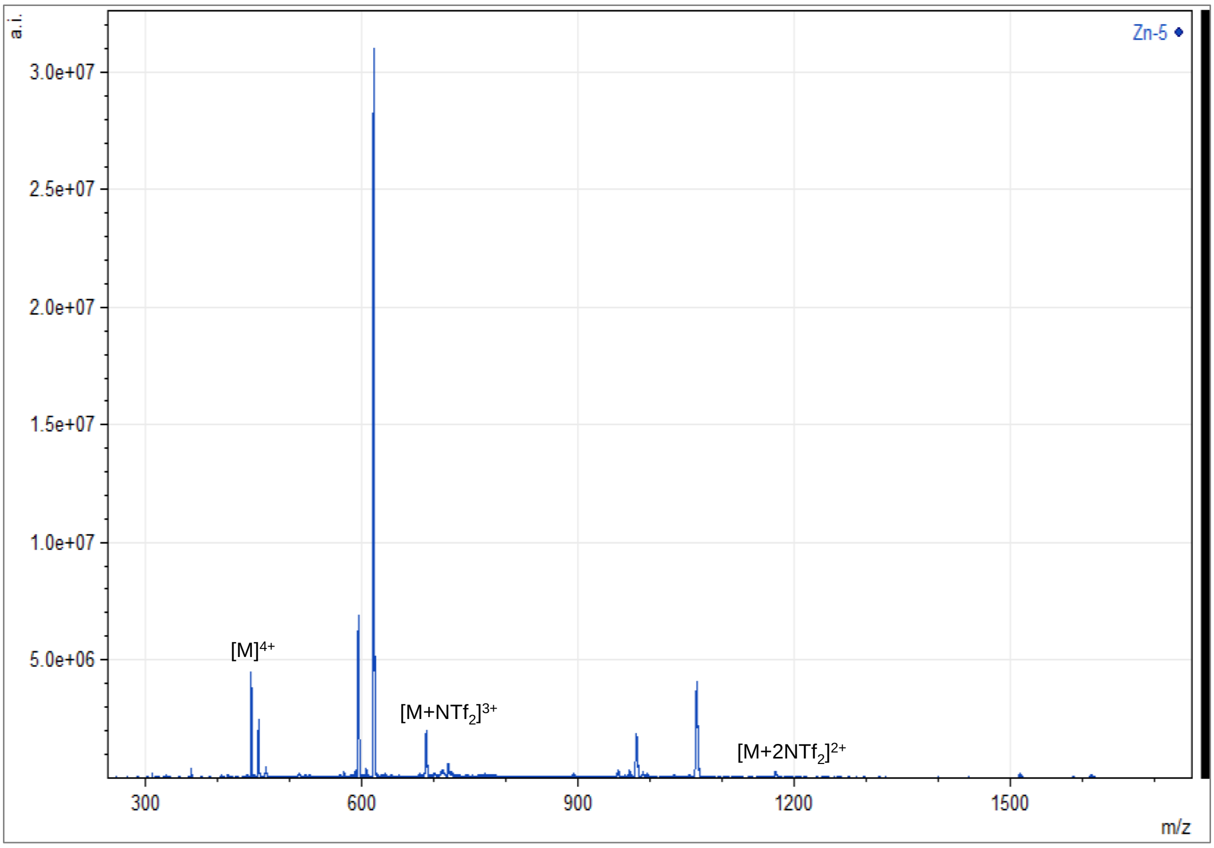

Figure S43. Low-resolution ESI-mass spectrum of *ΛΛ*-Zn-**5**.

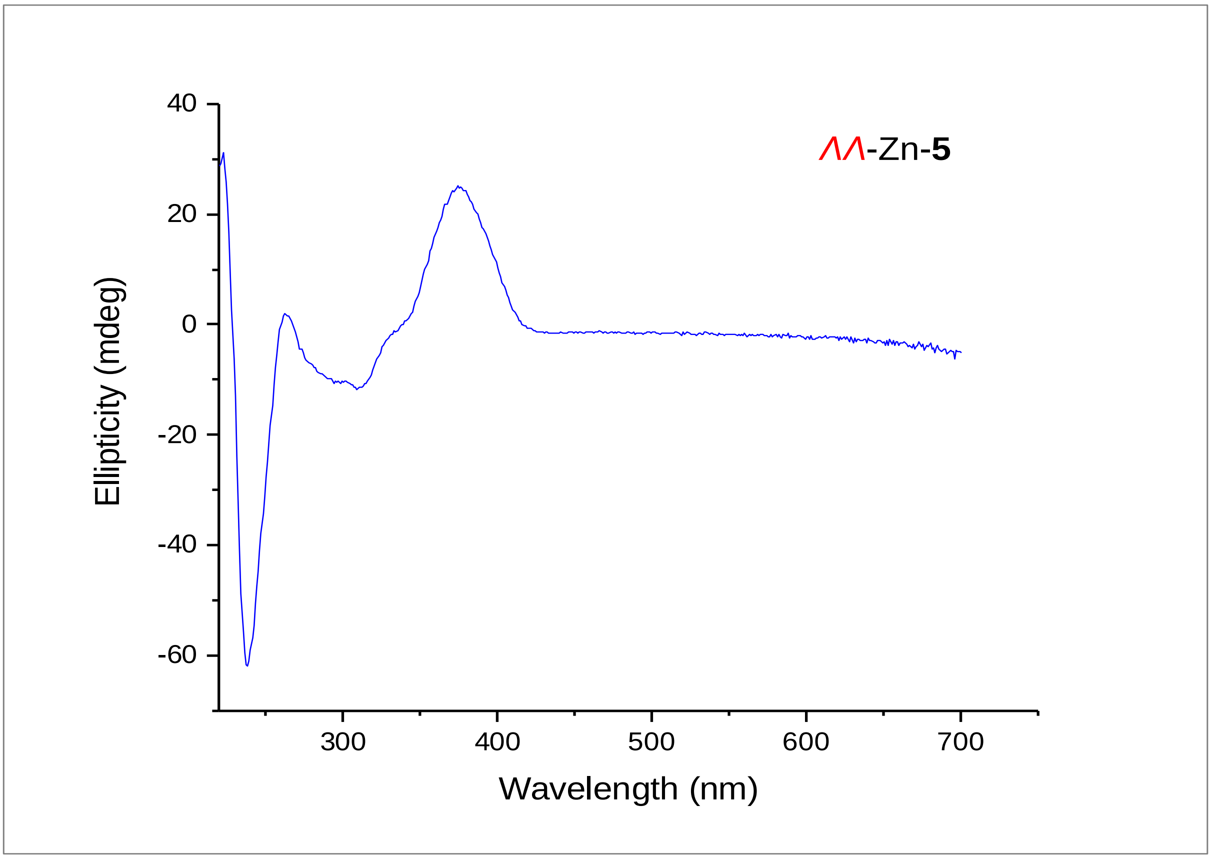

Figure S44. Circular dichroism (CD) spectrum of *ΛΛ*-Zn-**5**.

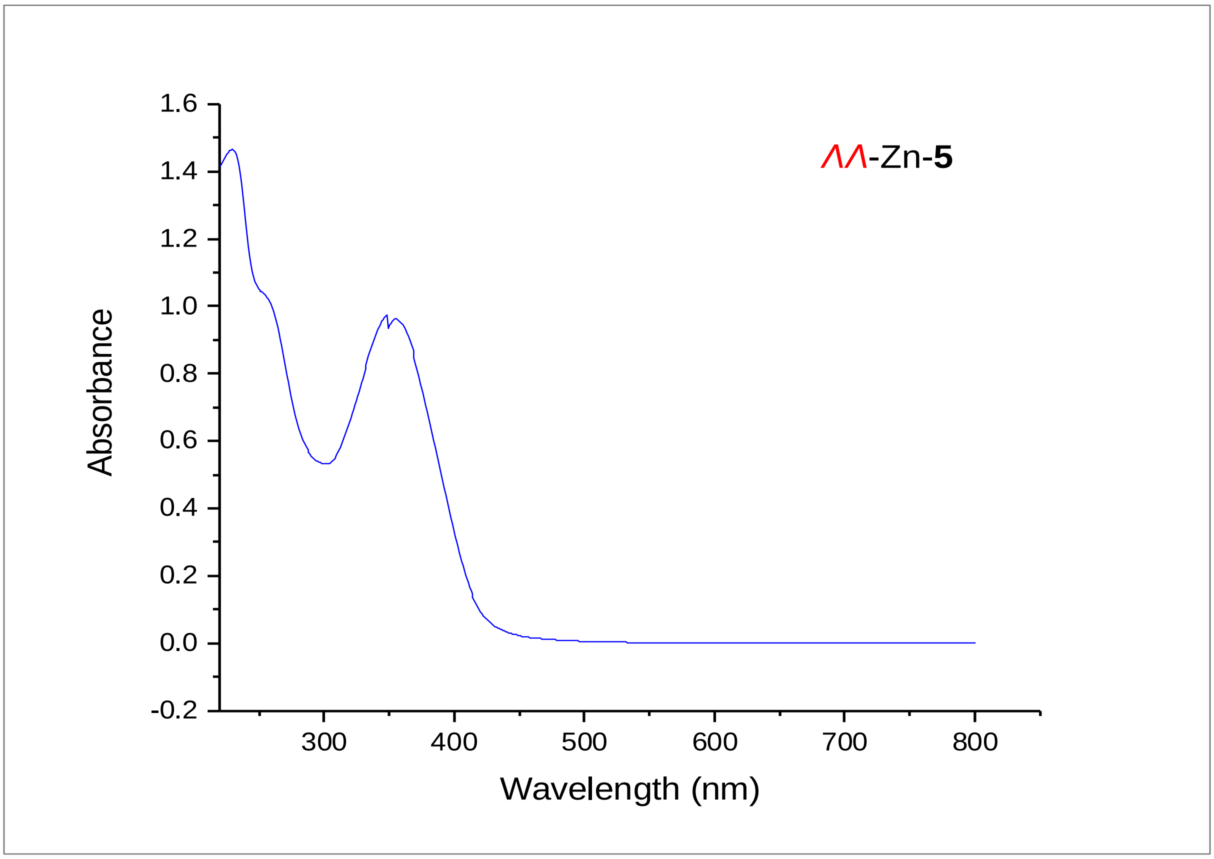

Figure S45. UV-Vis spectrum of *ΛΛ*-Zn-**5**.

3.2.3 using subcomponents A and D with Fe(II) salt

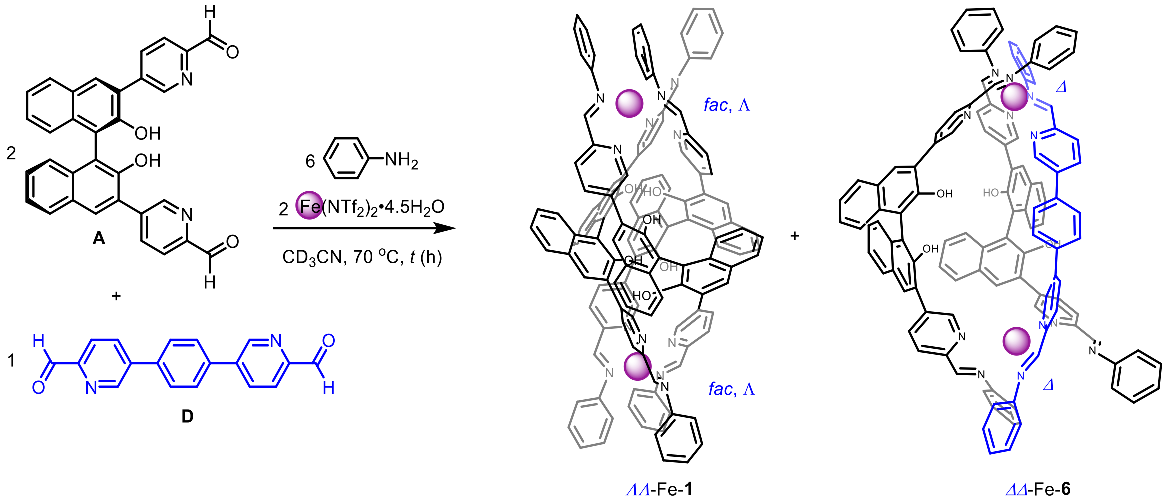

Subcomponents **A** (1.2 mg, 2.5 μmol, 1.0 equiv) and **D** (0.36 mg, 1.25 μmol, 0.5 equiv) were added to CD_3CN (0.6 mL) together with $Fe(NTf_2)_2$·4.5H_2O (1.7 mg, 2.5 μmol, 1.0 equiv) and aniline (0.7 mg, 7.5 μmol, 3.0 equiv). The reaction mixture was stirred at 70 °C and monitored by ^{1}H NMR. As shown in Figure S46, a new species (red, triangle) appearing at δ = 5.77 (d), 5.52 (d) and 5.41 (d), ppm was formed as the major product after 1 hour. The ^{1}H NMR spectrum was very close to that of *ΛΛ*-Fe-**5**, which indicated that Fe-**6**, assembled from two equivalents of **A** and one quivalent of **D** was formed. *Δ* Handedness of the two metal vertices in Fe-**6** was recorded from the CD and UV-vis spectrum (Figure S55 and S56). It is worth mentioning that only a small amout of *ΛΛ*-Fe-**1** was generated within 1 hour (blue, square). Another new species (black, star) appearing at δ = 8.83 (s) in the ^{1}H NMR spectrum was the homoleptic assembly from **D**.

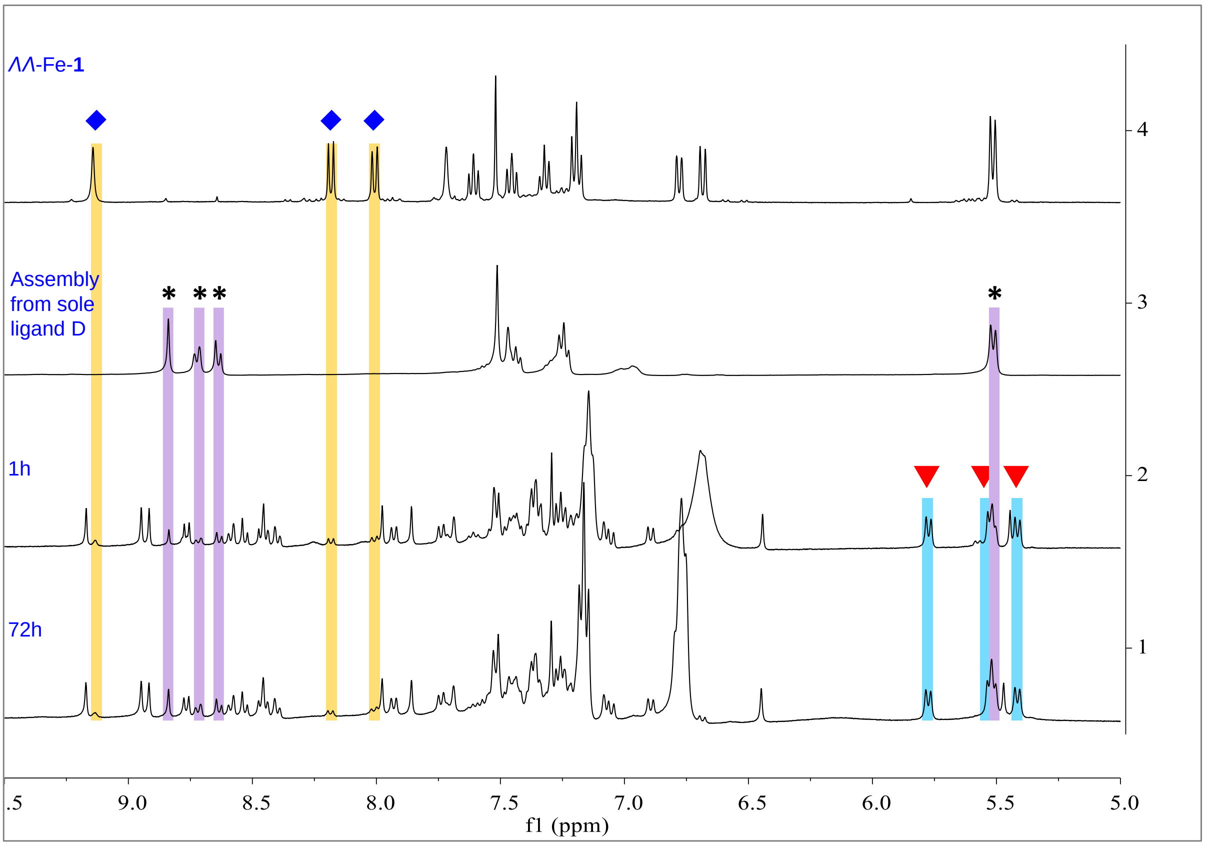

Figure S46. Crude ^{1}H NMR spectrum of self-assembly of **A**, **D** and aniline with $Fe(NTf_2)_2$ (400 MHz, CD_3CN, 298 K) (red triangle: *ΔΔ*-Fe-**6**; blue square: *ΛΛ*-Fe-**1**; black star: assemblies from sole **D**).

Characterization of *ΔΔ*-Fe-6:

^{1}H NMR (500 MHz, CD_3CN, 298 K) δ (ppm) = δ 9.17 (s, 2H), 8.94 (s, 2H), 8.91 (s, 2H), 8.76 (d, *J* = 7.9 Hz, 2H), 8.58 (m, 2H), 8.52 (d, *J* = 8.2 Hz, 2H), 8.47 – 8.42 (m, 4H), 8.39 (m, 2H), 7.97 (s, 2H), 7.92 (m, 2H), 7.85 (s, 2H), 7.76 – 7.72 (m, 2H), 7.68 (d, *J* = 1.7 Hz, 2H), 7.52 – 7.04 (m. 34H), 6.89 (m, 4H), 6.43 (s, 2H), 5.79 – 5.75 (m, 4H), 5.51 (t, *J* = 1.2 Hz, 4H), 5.43 (s, 2H), 5.43 – 5.39 (m, 4H). (Note: due to the overlap with the asseblies from sole **D**, ranges from δ 7.04 to 7.54 ppm were not integrated).

^{13}C NMR (125 MHz, CD_3CN, 298 K) δ (ppm) = 175.5, 175.3, 174.5, 154.9, 142.6, 142.6, 141.4, 138.6, 133.1, 132.4, 131.7, 131.7, 131.3, 130.9, 129.7, 129.3, 124.8, 122.7, 122.4, 122.4, 118.9. (Note: due to the overlap with the asseblies from **D**, not all ^{13}C NMR signals could be distinguished).

ESI-MS *m/z* 460.8 $[M]^{4+}$, 707.8 $[M+NTf_2]^{3+}$, 1201.6 $[M+2NTf_2]^{2+}$.

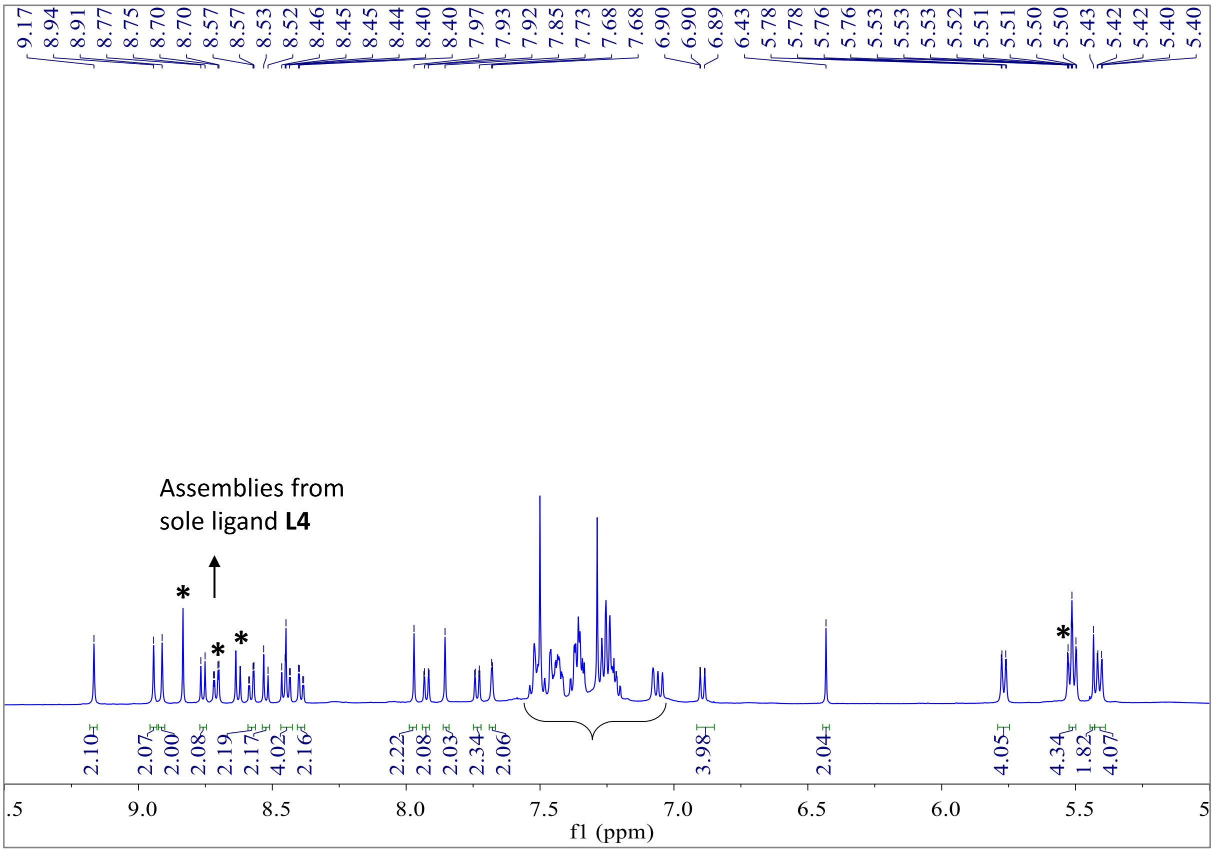

Figure S47. ^{1}H NMR spectrum of *ΔΔ*-Fe-**6** (500 MHz, CD_3CN, 298 K).

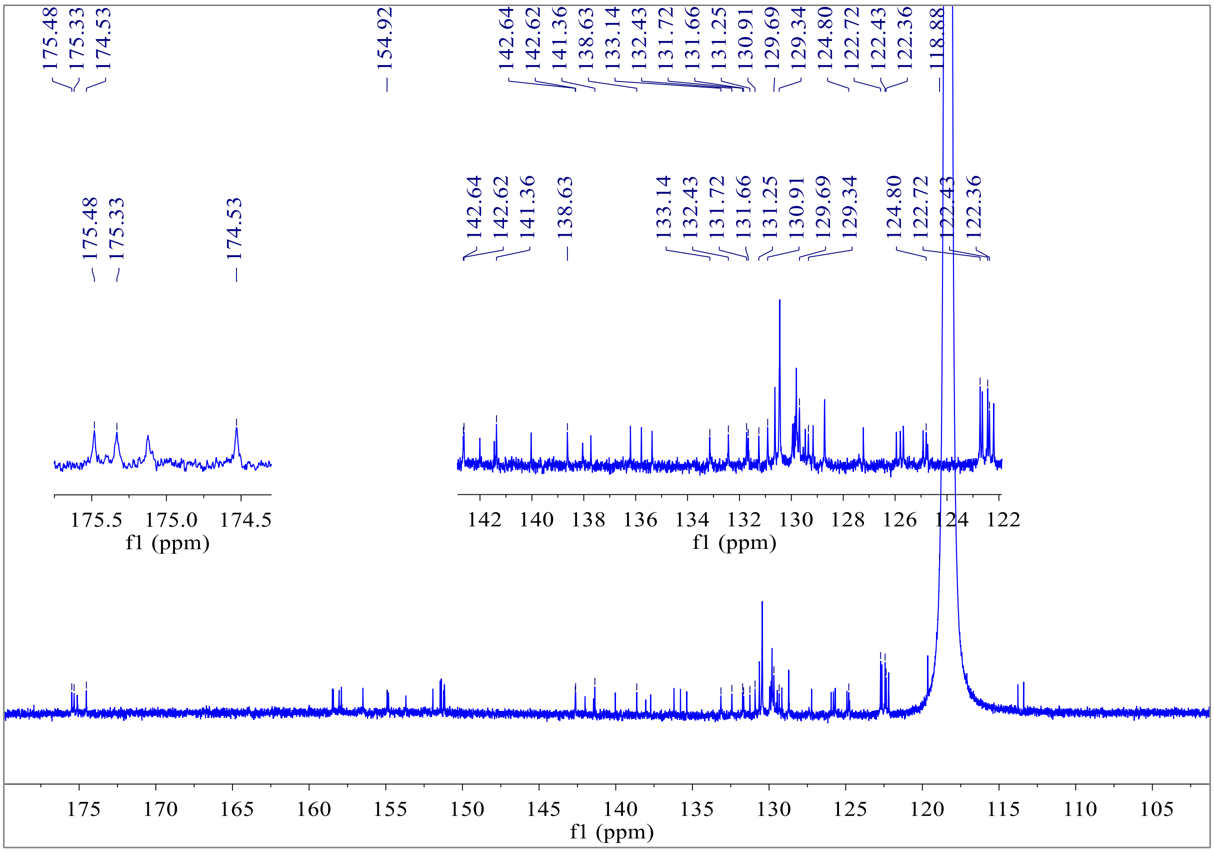

Figure S48. ^{13}C NMR spectrum of *ΔΔ*-Fe-**6** (125 MHz, CD_3CN, 298 K).

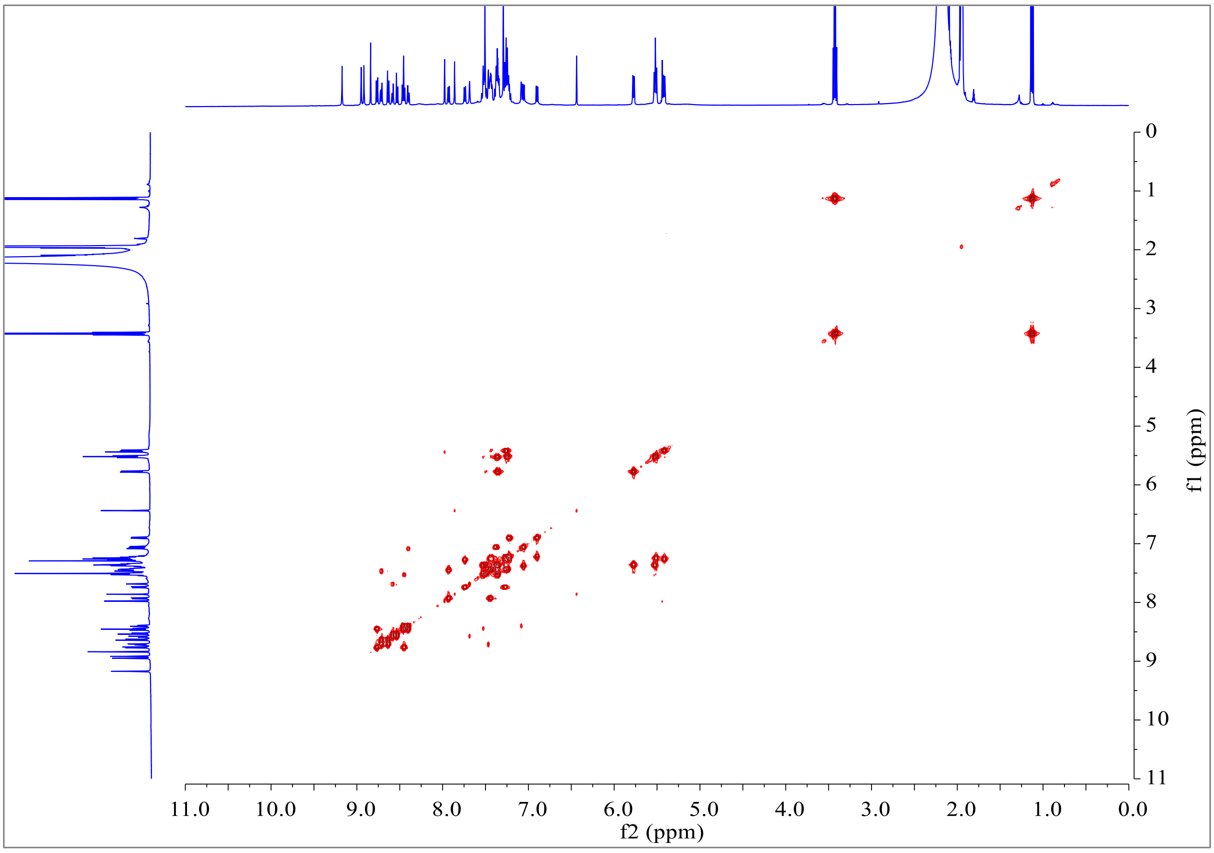

Figure S49. $^{1}H\{^{1}H\}$ COSY NMR spectrum of *ΔΔ*-Fe-**6** (500 MHz, CD_3CN, 298 K).

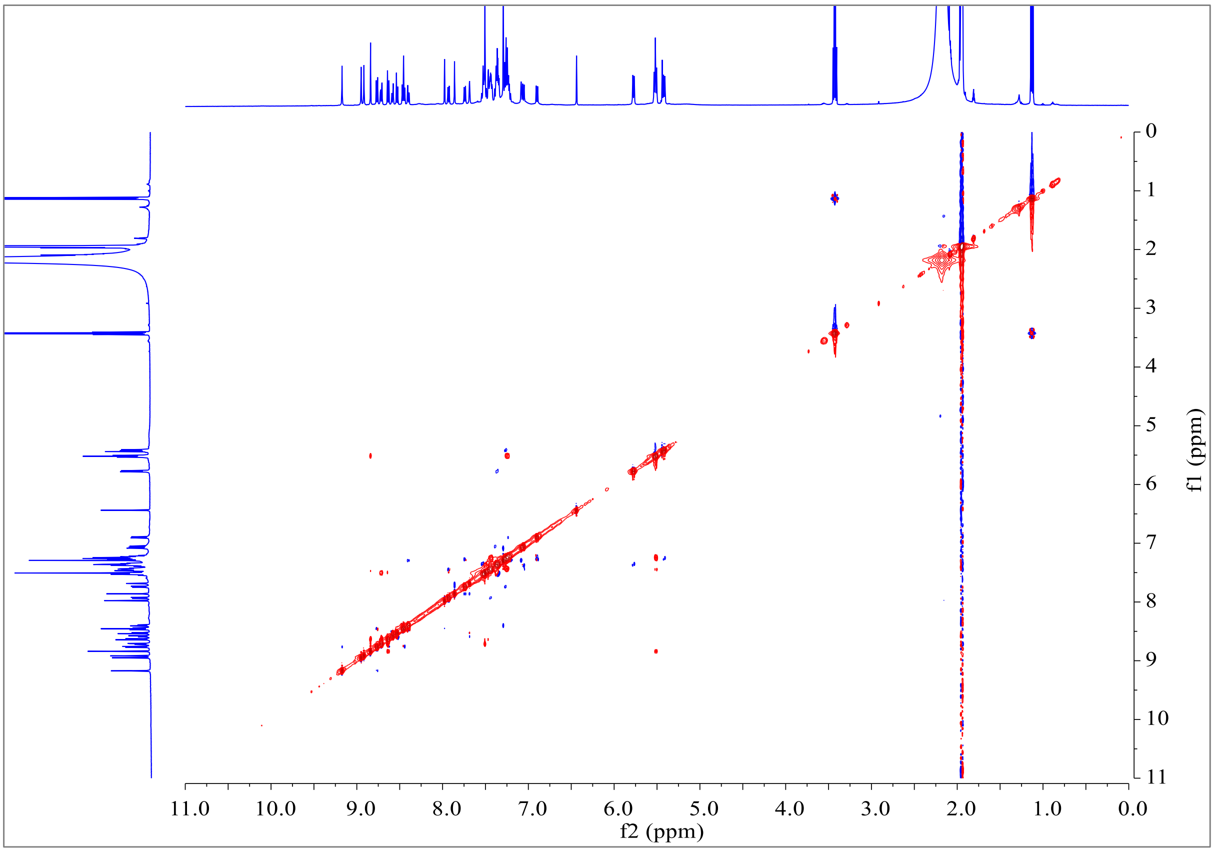

Figure S50. $^{1}H\{^{1}H\}$ NOESY NMR spectrum of *ΔΔ*-Fe-**6** (500 MHz, CD_3CN, 298 K).

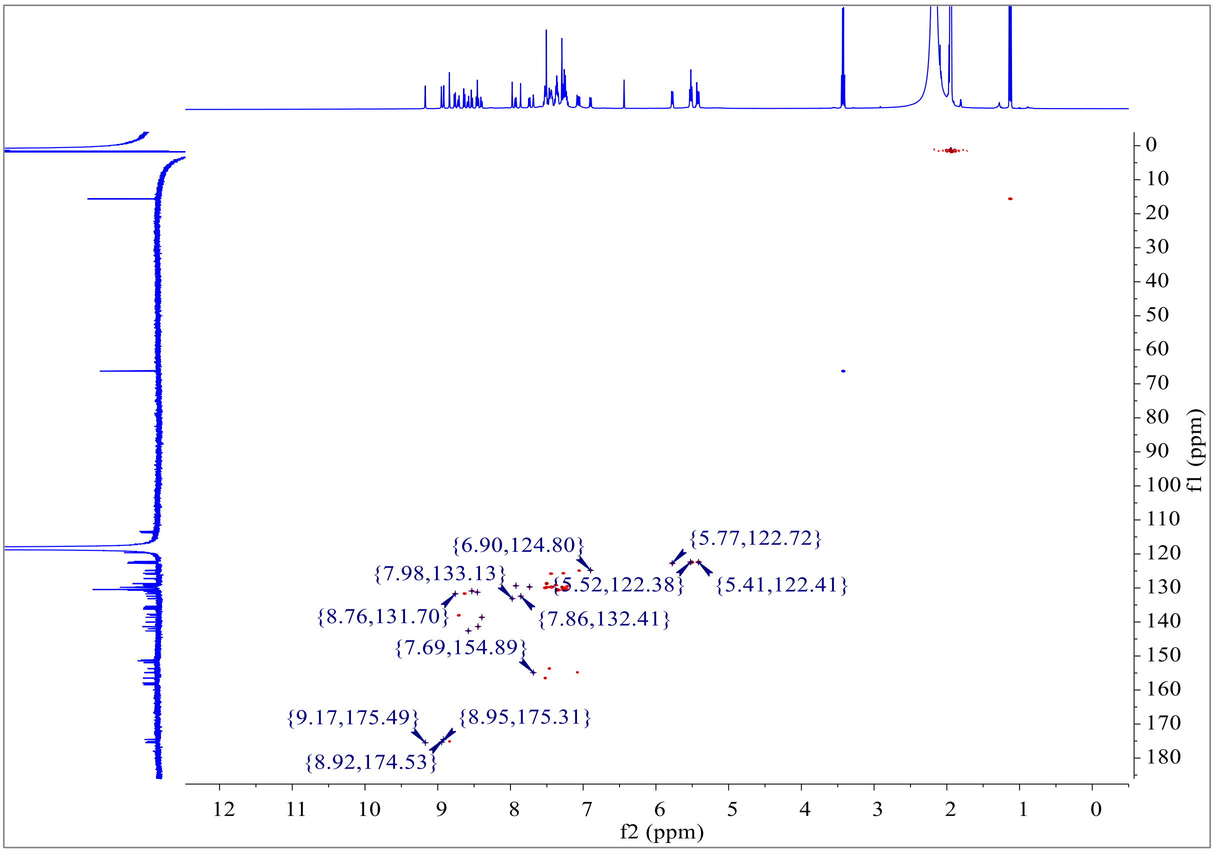

Figure S51. $^{1}H\{^{13}C\}$ HSQC NMR spectrum of *ΔΔ*-Fe-**6** (500 MHz, CD_3CN, 298 K).

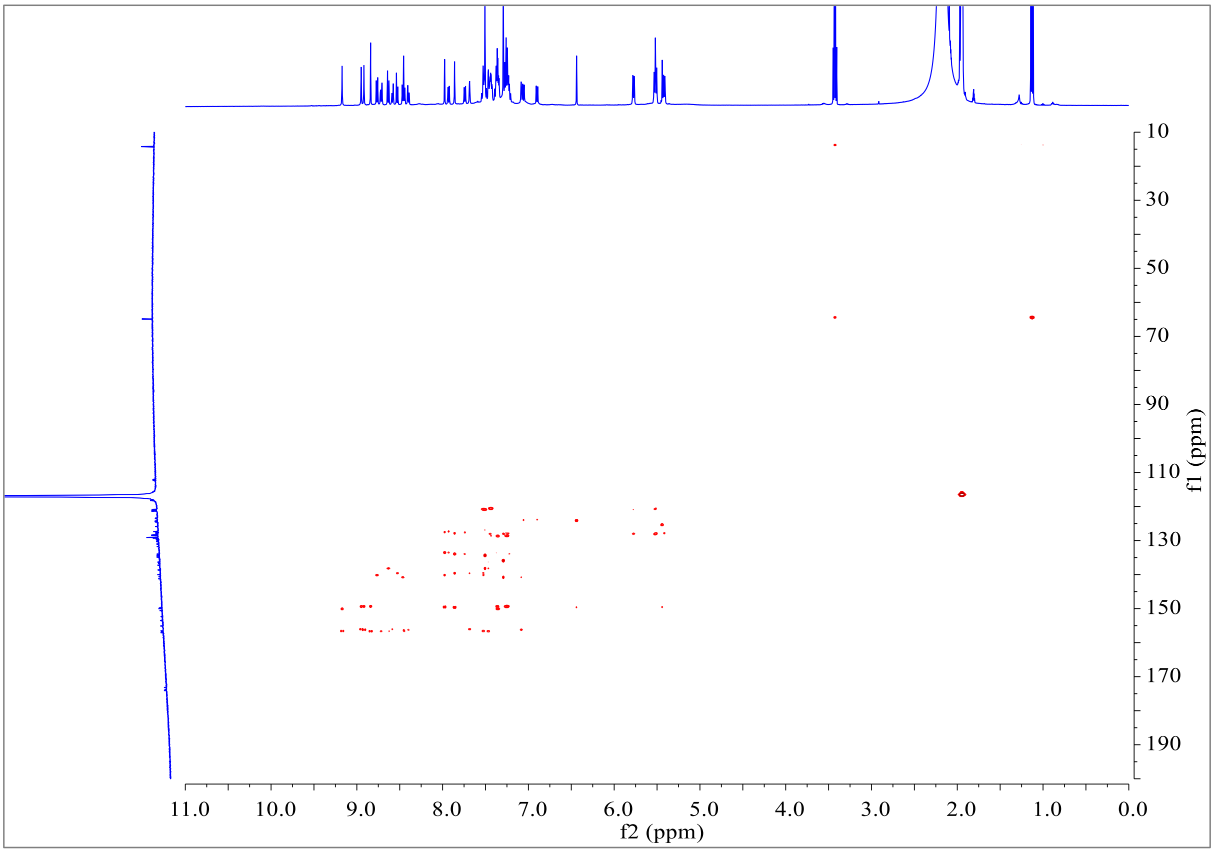

Figure S52. $^{1}H\{^{13}C\}$ HMBC NMR spectrum of *ΔΔ*-Fe-**6** (500 MHz, CD_3CN, 298 K).

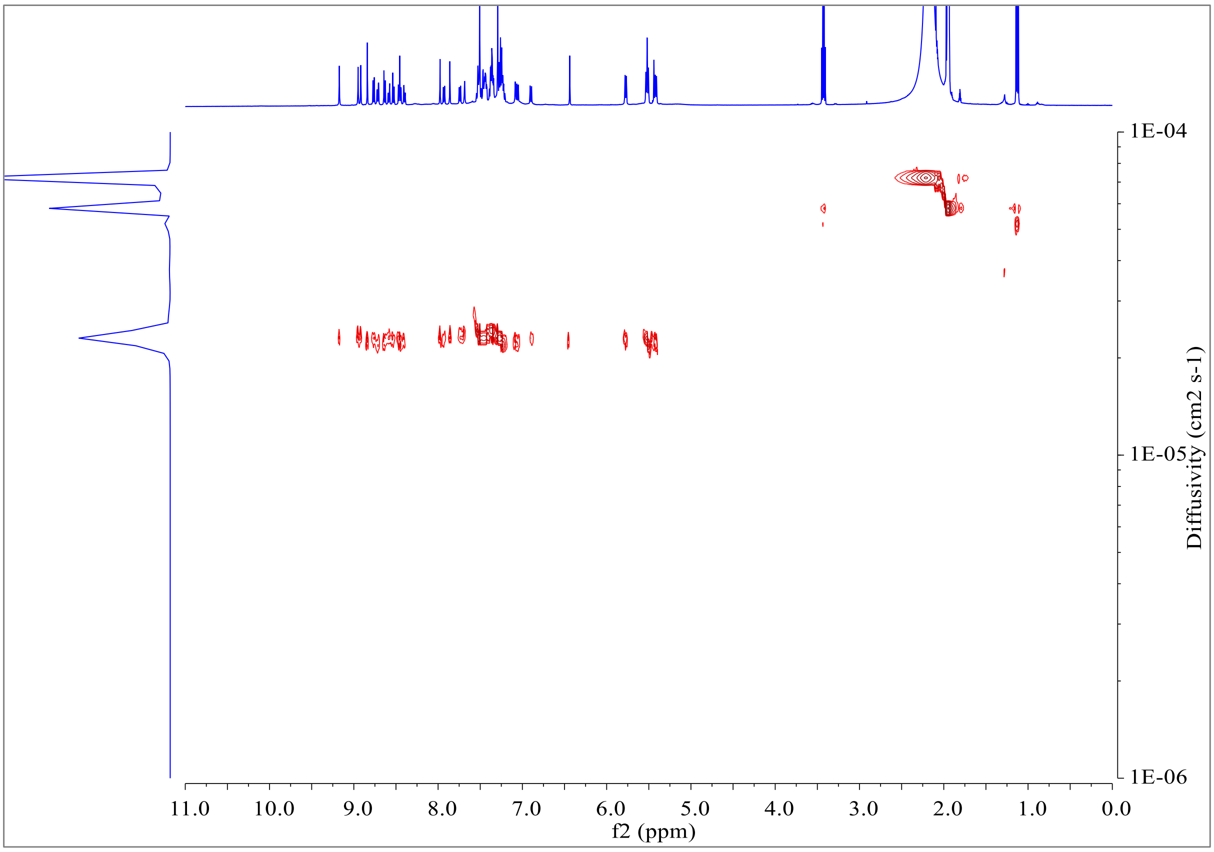

Figure S53. ^{1}H DOSY NMR spectrum of *ΔΔ*-Fe-**6** (400 MHz, CD_3CN, 298 K).

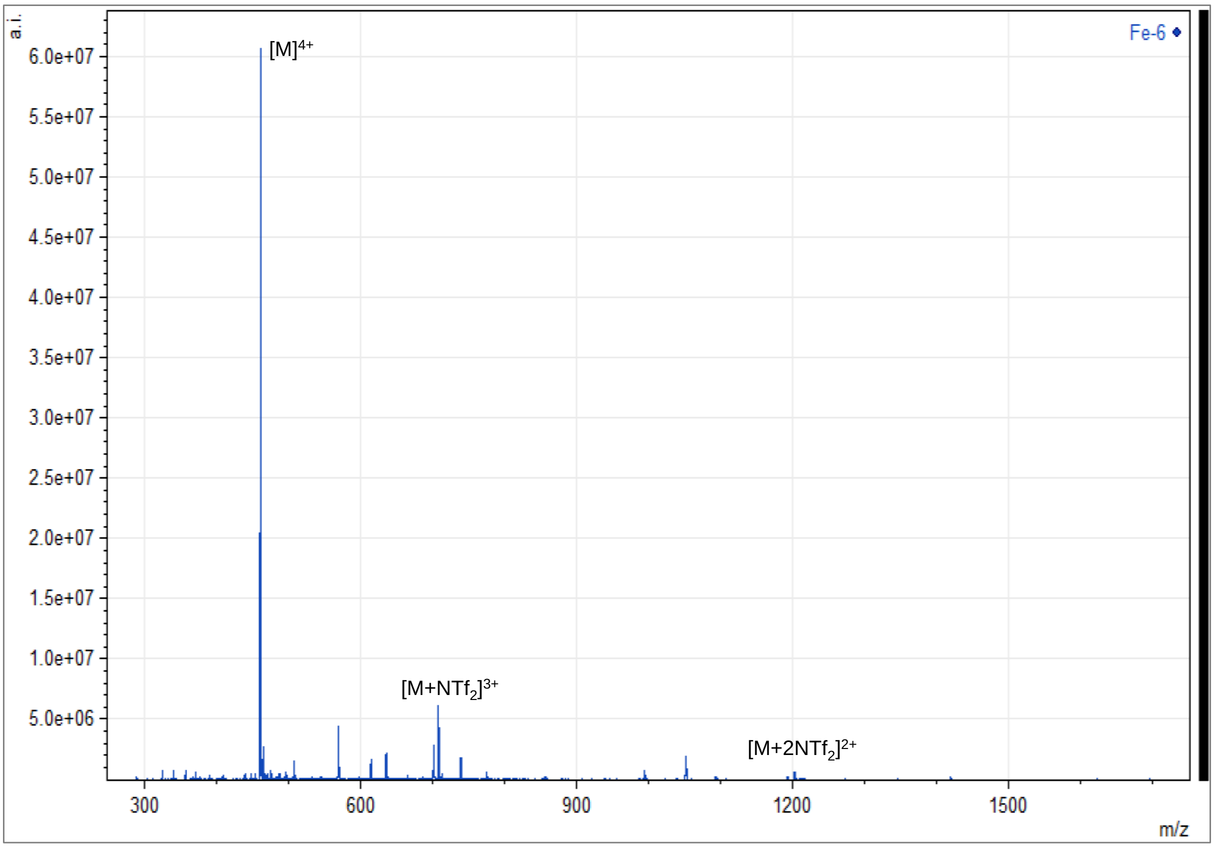

Figure S54. Low-resolution ESI-mass spectrum of *ΔΔ*-Fe-**6**.

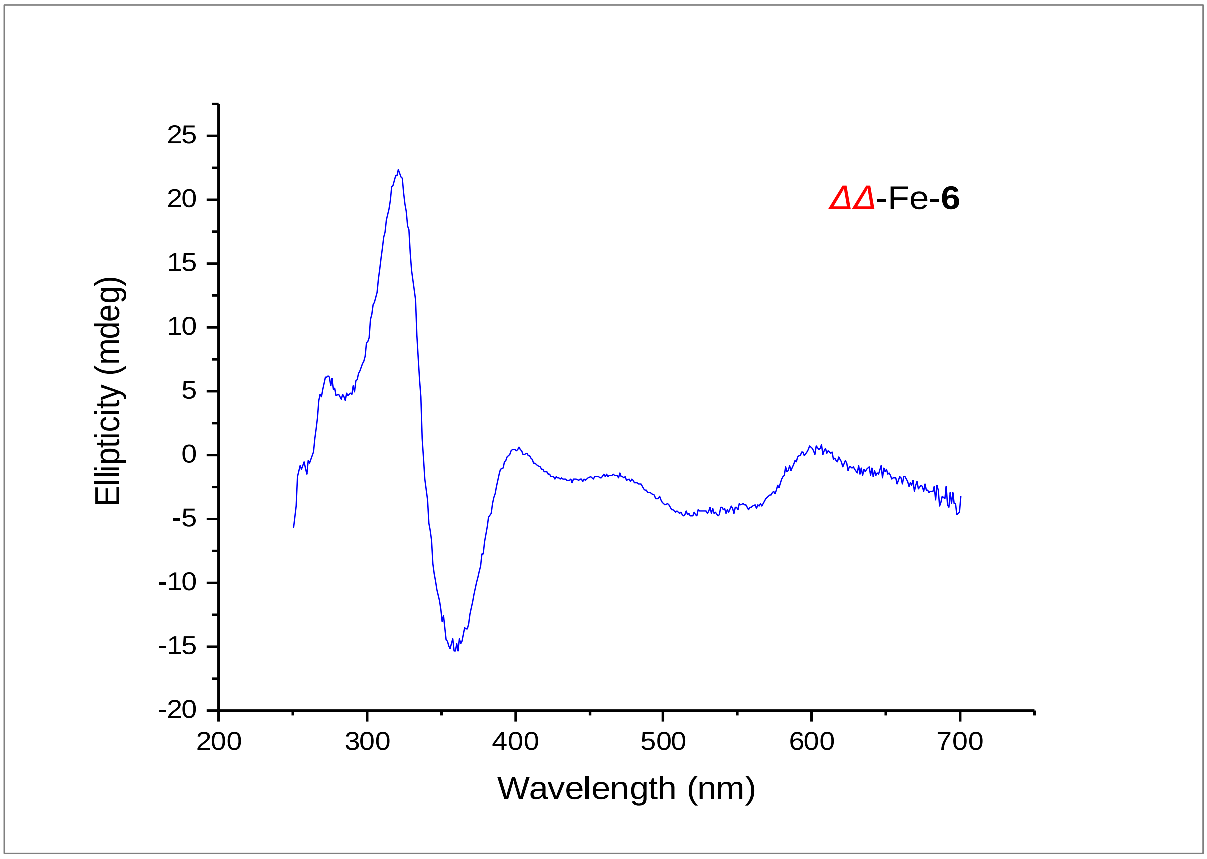

Figure S55. Circular dichroism (CD) spectrum of *ΔΔ*-Fe-**6**.

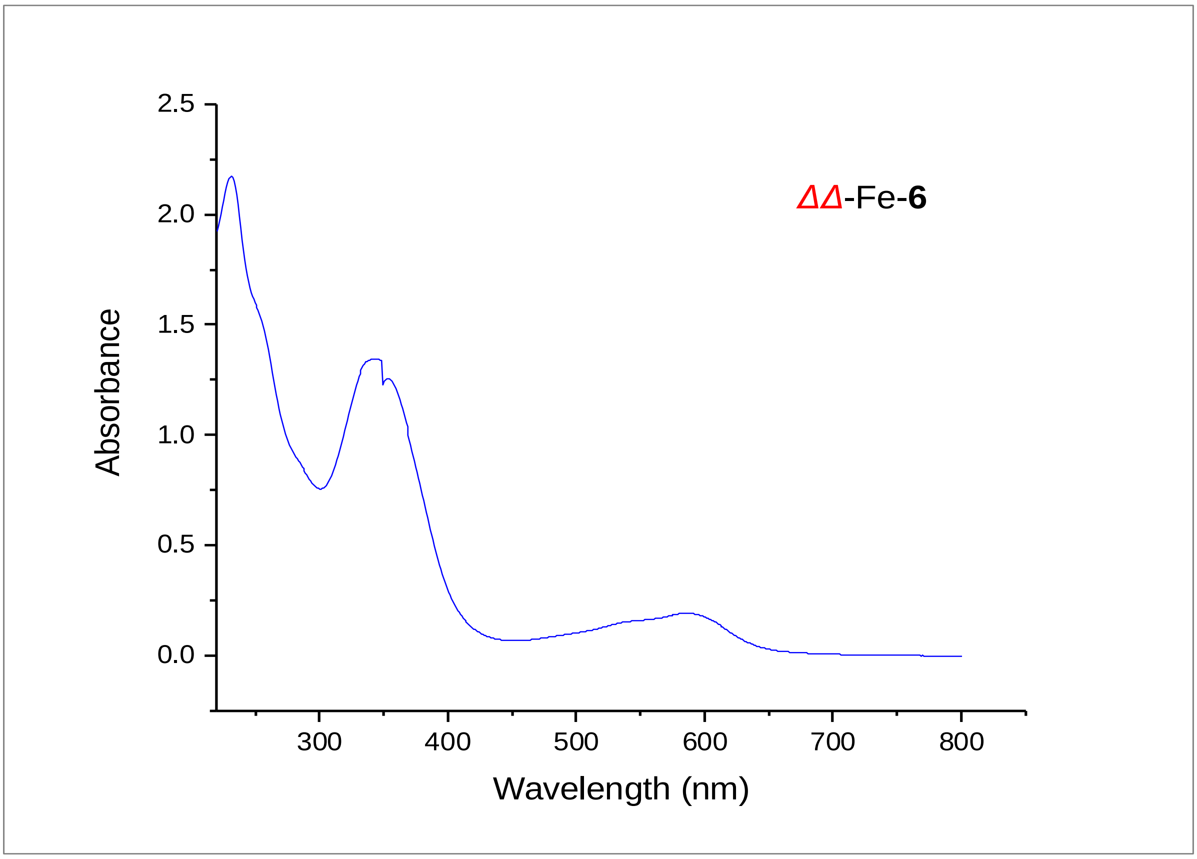

Figure S56. UV-Vis spectrum of *ΔΔ*-Fe-**6**.

3.2.4 using subcomponents A and D with Zn(II) salt

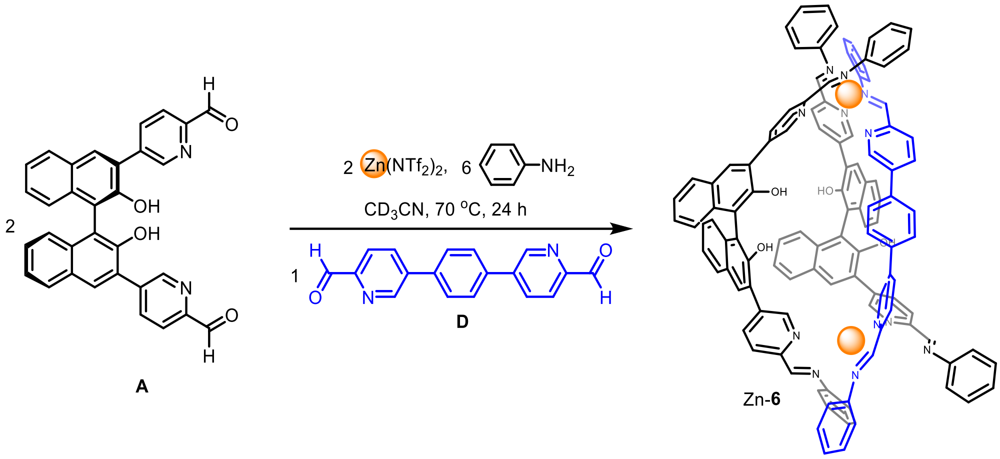

Subcomponents **A** (1.2 mg, 2.5 μmol, 1.0 equiv) and **D** (0.36 mg, 1.25 μmol, 0.5 equiv) were added to CD_3CN (0.6 mL) together with $Zn(NTf_2)_2$ (1.6 mg, 2.5 μmol, 1.0 equiv) and aniline (0.7 mg, 7.5 μmol, 3.0 equiv). The reaction mixture was stirred at 70 °C for 24 hours. As shown in Figure S57, a new species appearing at δ = 6.24 (d) and 6.27 (d) ppm was formed. The ^1H NMR spectrum was very close to that of Zn-**4**, which indicated that Zn-**6**, assembled from two equivalents of **A** and one quivalent of **D** with $Zn(NTf_2)_2$ was formed. The formation of Zn-**6** was further confirmed by MS analysis (Figure S58). (Note: due to the overlap with some impurities, the ^{13}C and 2D NMR spectra of Zn-**6** were not performed).

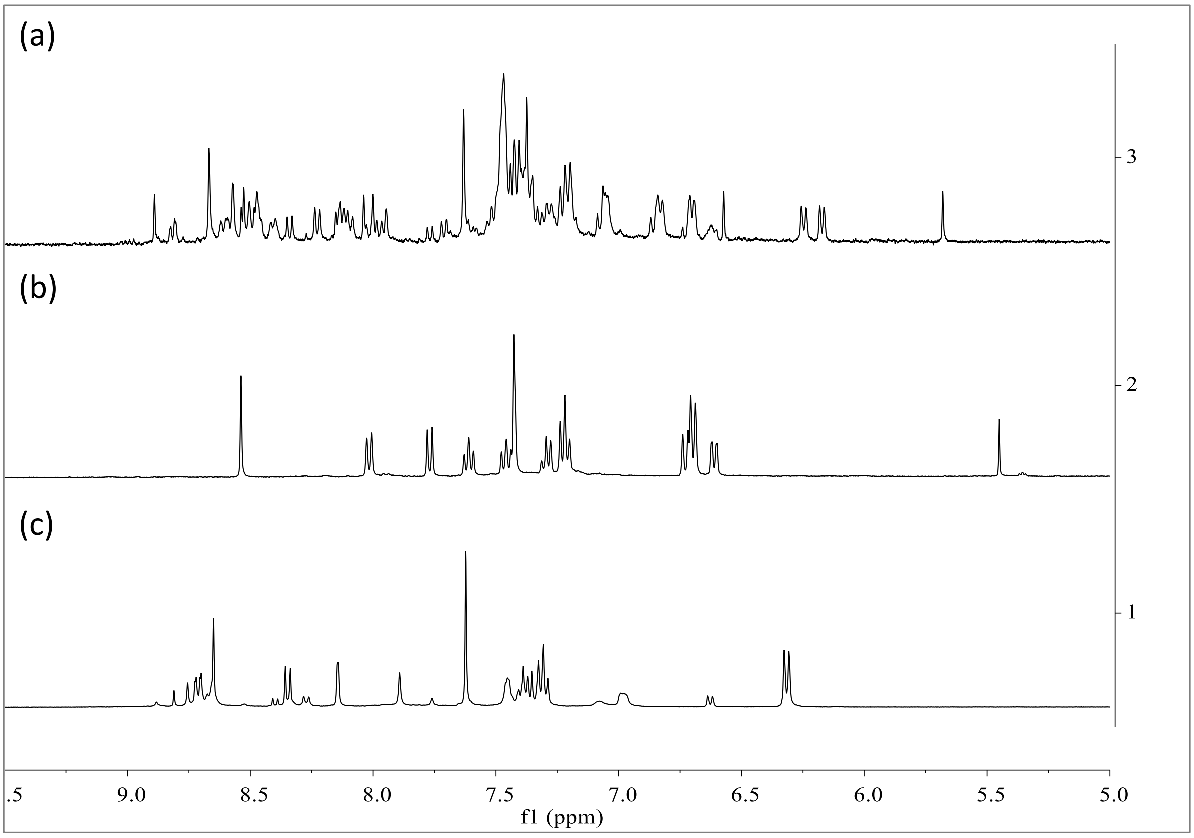

Figure S57. (a) ^{1}H NMR of self-assembly of a mixture of **A** and **D** (2:1) with aniline and $Zn(NTf_2)_2$. (b) ^{1}H NMR of cage Zn-**1**. (c) ^{1}H NMR of self-assembly of **D** with aniline and $Zn(NTf_2)_2$. (400M NMR, CD_3CN, 298K).

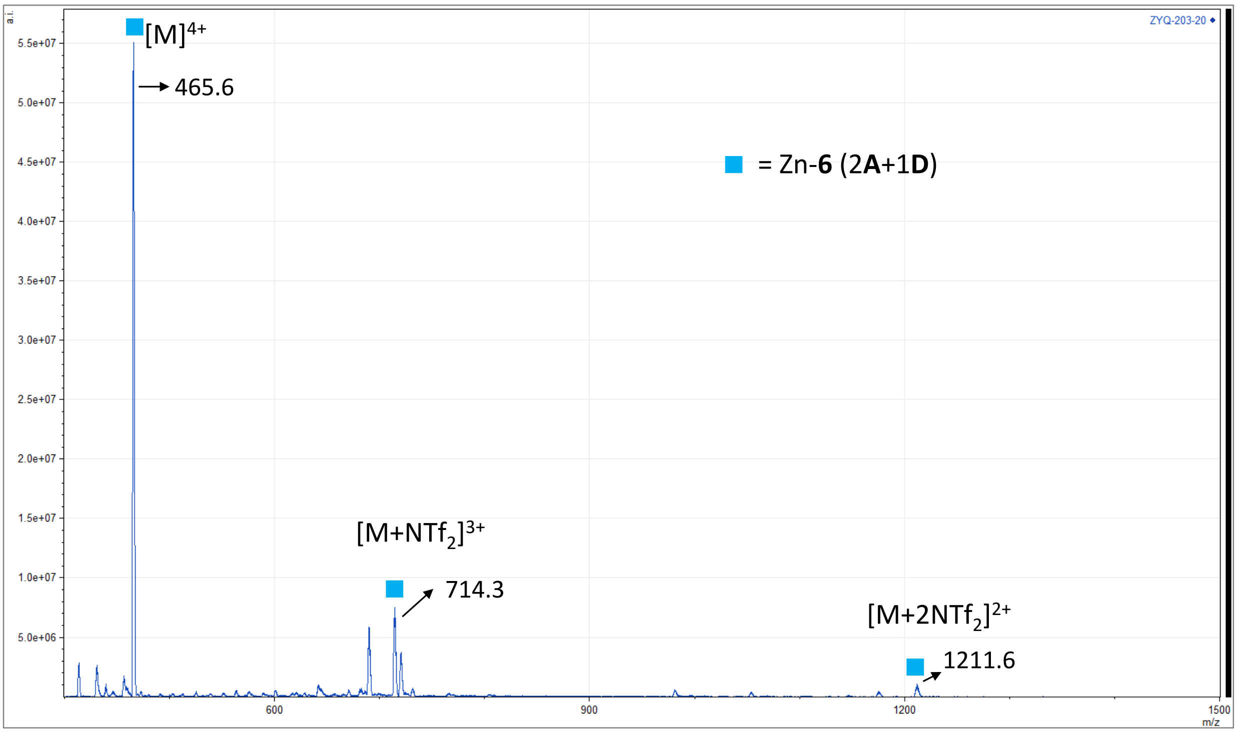

Figure S58. Low-resolution ESI-mass spectrum of Zn-**6**.

4. Self-assembly using subcomponent B

4.1 Sole subcomponent B

4.1.1 with Zn(II) salt

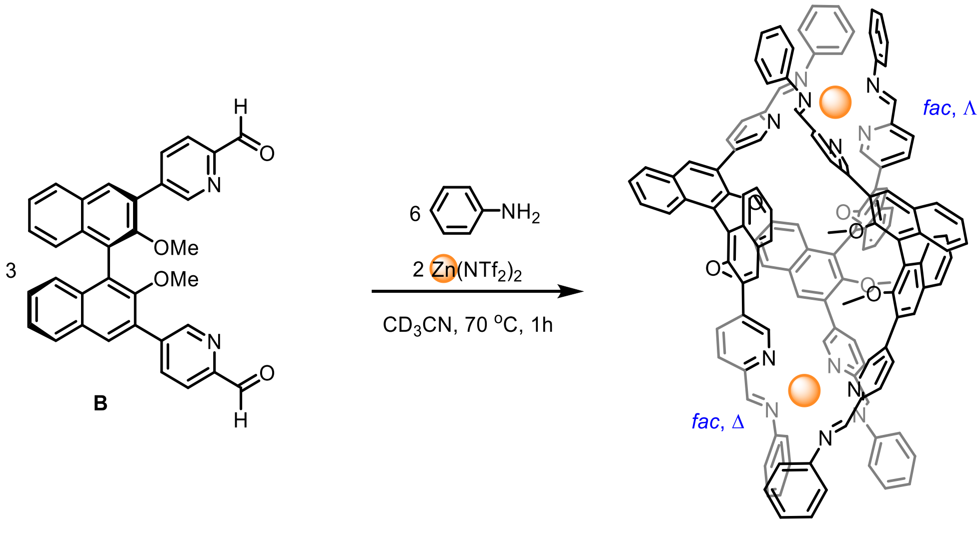

Subcomponent **B** (2.0 mg, 3.8 µmol, 1.5 equiv) was added to CD_3CN (0.6 mL) together with $Zn(NTf_2)_2$ (1.6 mg, 2.5 µmol, 1.0 equiv) and aniline (0.7 mg, 7.5 µmol, 3.0 equiv). The reaction mixture was stirred at 70 °C for 1h. After cooling to room temperature, the solvent was evaporated and diethyl ether was then added. The residue resuspended and then centrifuged and the diethyl ether decanted. This was repeated three times with fresh diethyl ether. The residue was then dried in vacuo to afford the desired product (*ΛΔ*-Zn-**2**) as a pale yellow solid (3.5 mg, 85% yield).

Characterization of ***ΛΔ*****-Zn-2:**

^{1}H NMR (400 MHz, CD_3CN, 298 K) δ (ppm) = 8.80 (s, 3H), 8.76 (d, *J* = 8.2 Hz, 3H), 8.64 (m, 3H), 8.44 (t, *J* = 4.0 Hz, 6H), 8.22 (d, *J* = 7.9 Hz, 3H), 8.17 (s, 6H), 8.05 (d, *J* = 8.2 Hz, 3H), 7.87 (s, 3H), 7.73 (s, 3H), 7.52 (d, *J* = 6.9 Hz, 6H), 7.45 (m, 6H), 7.37 (m, 15H), 7.23 (d, *J* = 7.8 Hz, 6H), 7.14 (t, *J* = 7.7 Hz, 3H), 6.74 (d, *J* = 8.5 Hz, 3H), 6.34 (d, *J* = 7.7 Hz, 6H), 6.23 (d, *J* = 7.5 Hz, 6H), 2.97 (s, 9H), 1.72 (s, 9H).

^{13}C{^{1}H} DEPT-135 (100 MHz, CD_3CN, 298 K) δ (ppm) = 165.4, 164.4, 149.8, 148.1, 144.3, 141.9, 134.2, 131.6, 131.2, 130.6, 130.6, 130.1, 129.5, 129.3, 129.3, 129.0, 128.7, 127.1, 126.5, 126.4, 125.5, 122.4, 122.0, 61.7, 60.4.

ESI-MS *m/z* 538.6 $[M]^{4+}$, 811.6 $[M+NTf_2]^{3+}$, 1357.4 $[M+2NTf_2]^{2+}$.

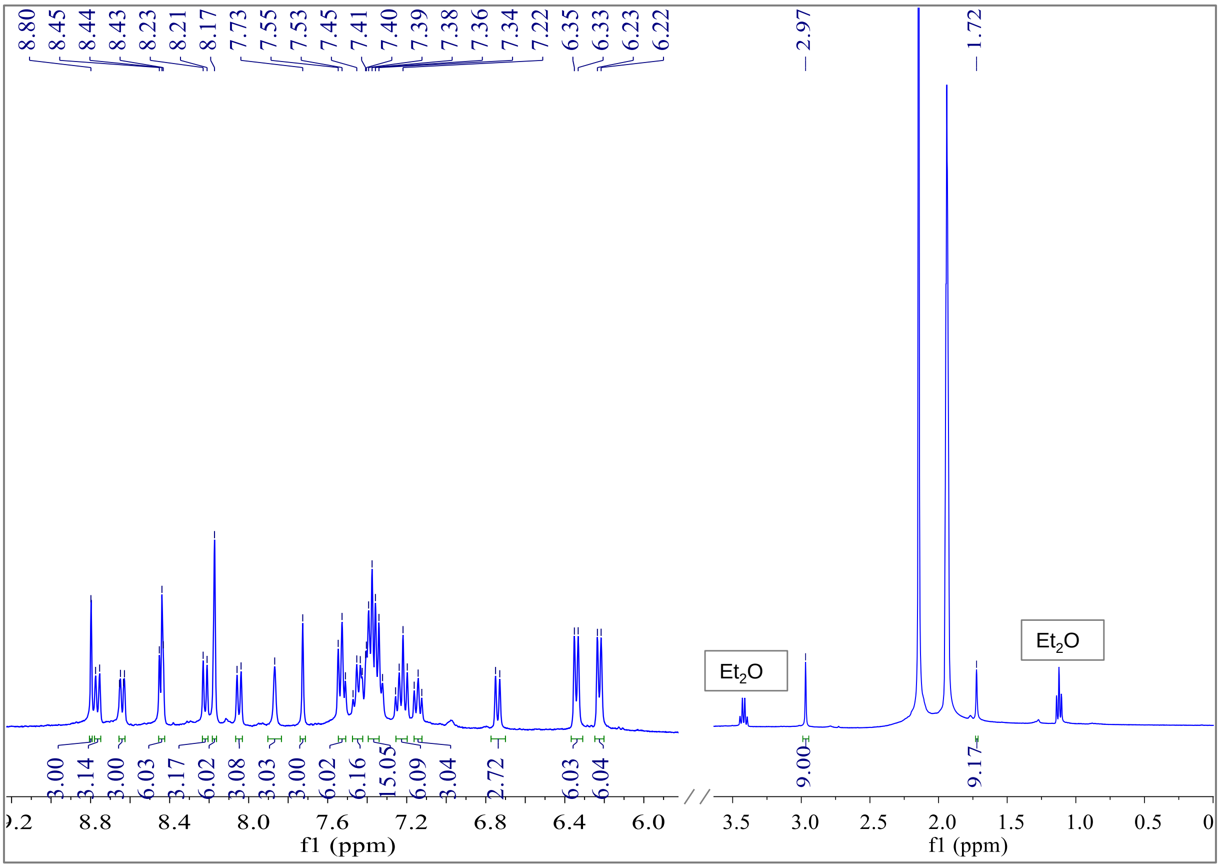

Figure S59. ^{1}H NMR spectrum of *Λ∆*-Zn-**2** (400 MHz, CD3CN, 298 K).

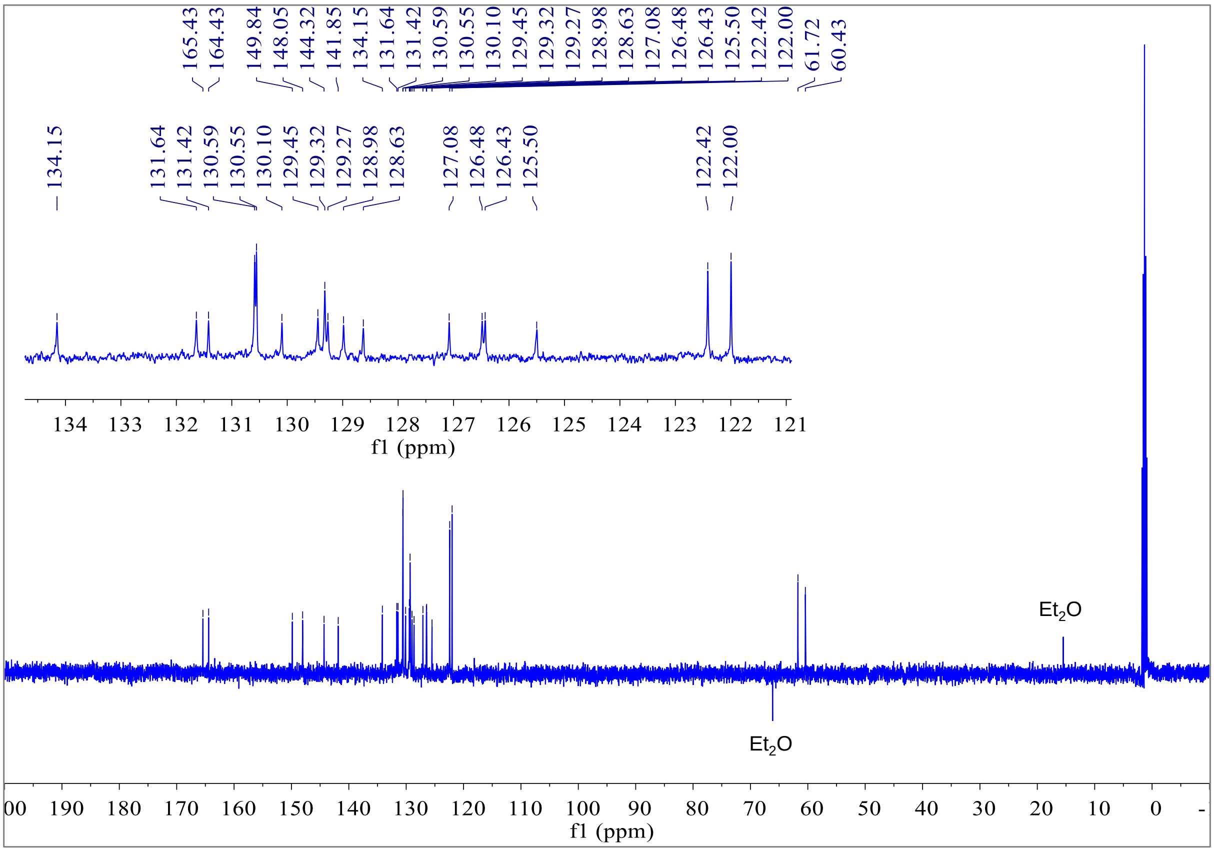

Figure S60. ^{13}C{^{1}H} DEPT-135 NMR spectrum of *Λ∆*-Zn-**2** (100 MHz, CD3CN, 298 K).

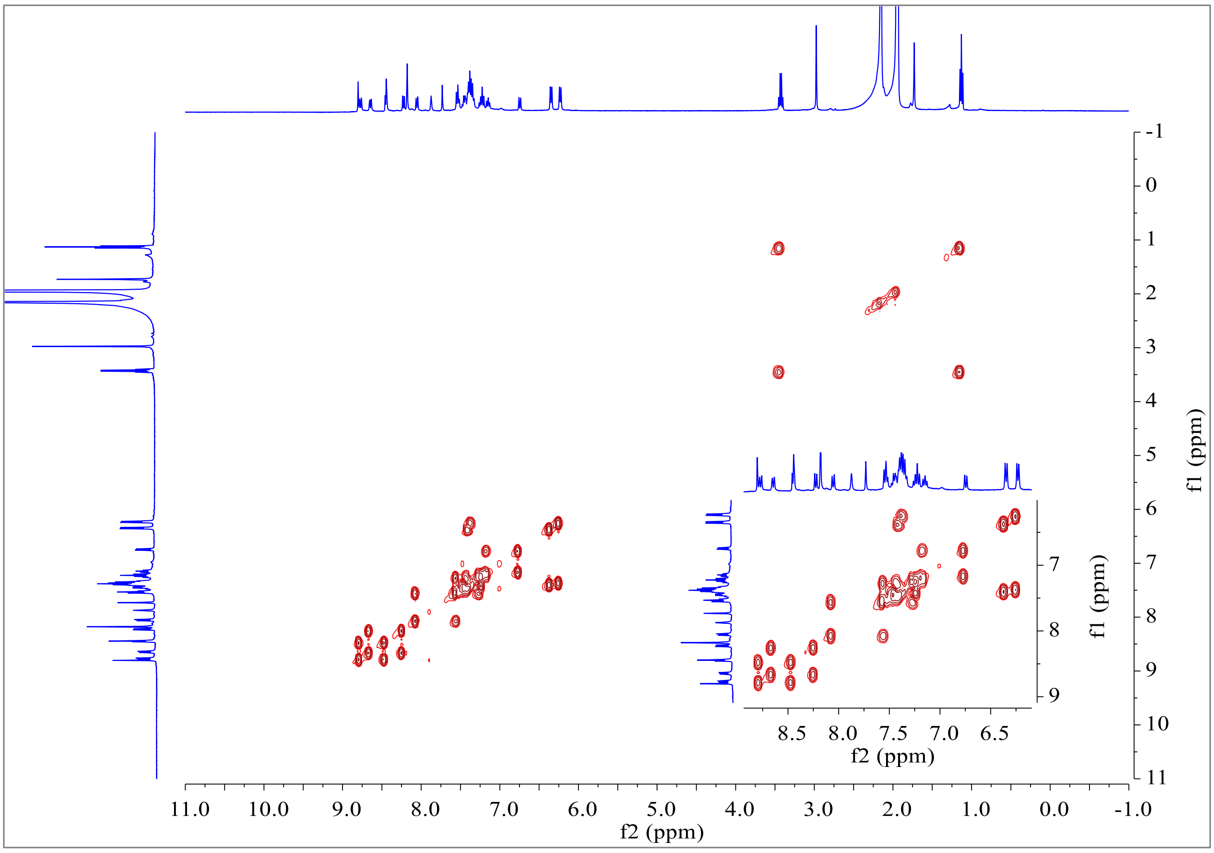

Figure S61. $^{1}H\{^{1}H\}$ COSY NMR spectrum of *Λ**Δ*-Zn-**2** (400 MHz, CD_3CN, 298 K).

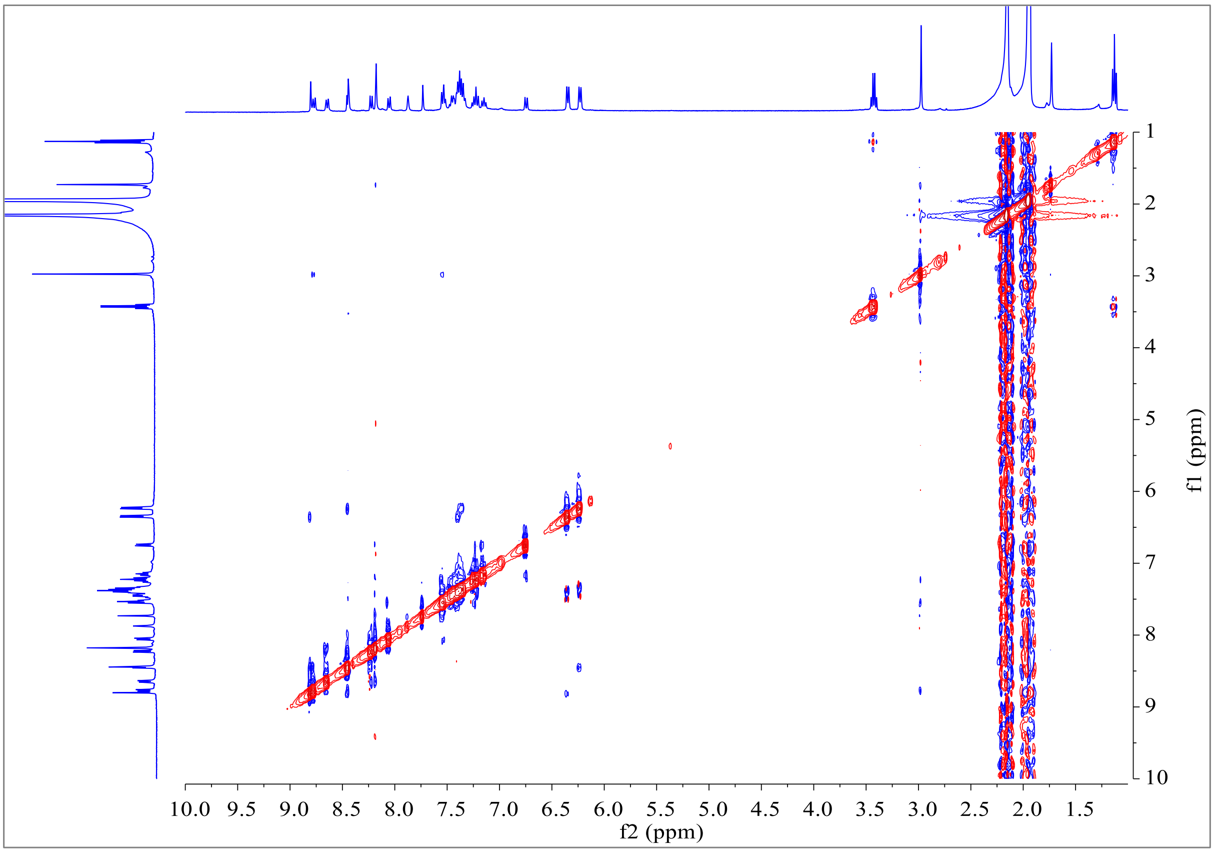

Figure S62. $^{1}H\{^{1}H\}$ NOESY NMR spectrum of *Λ**Δ*-Zn-**2** (400 MHz, CD_3CN, 298 K).

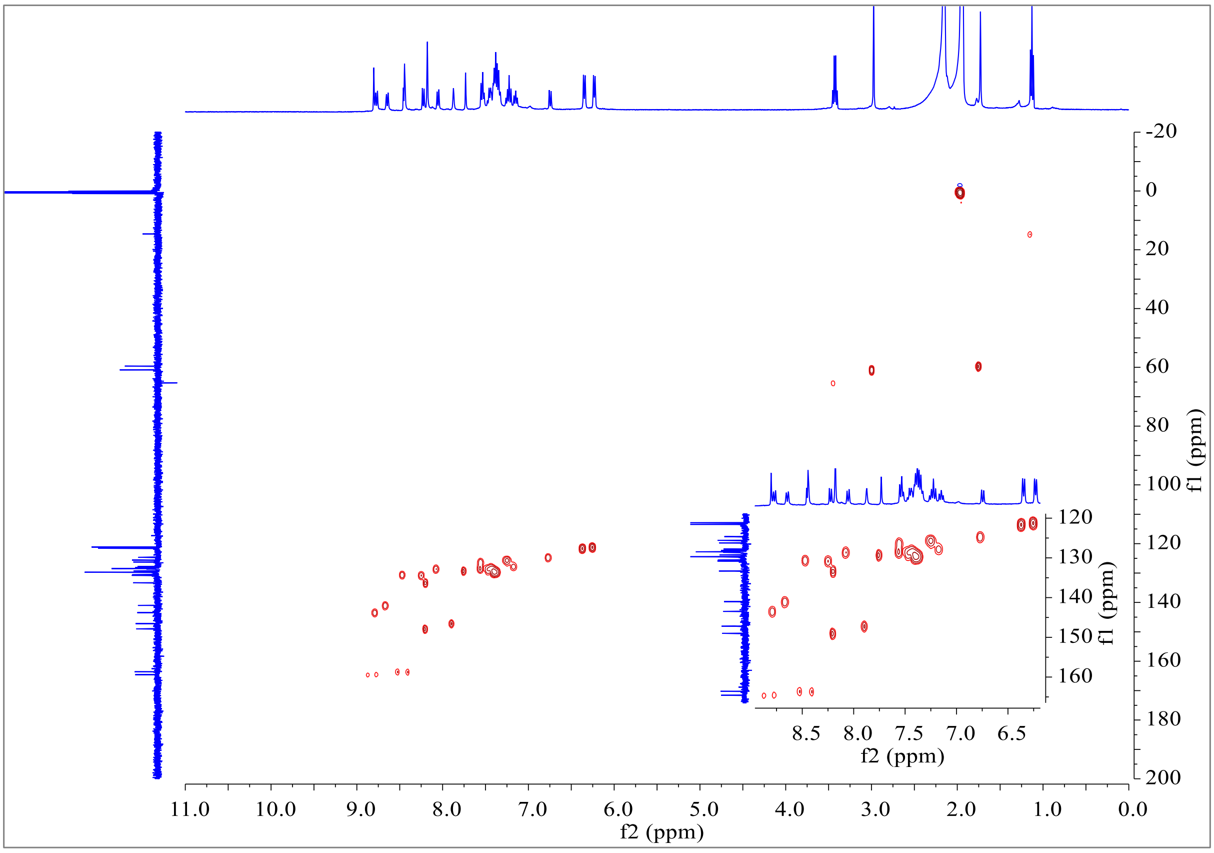

Figure S63. ¹H{¹³C} HSQC NMR spectrum of *Λ*Δ-Zn-**2** (400 MHz, CD_3CN, 298 K).

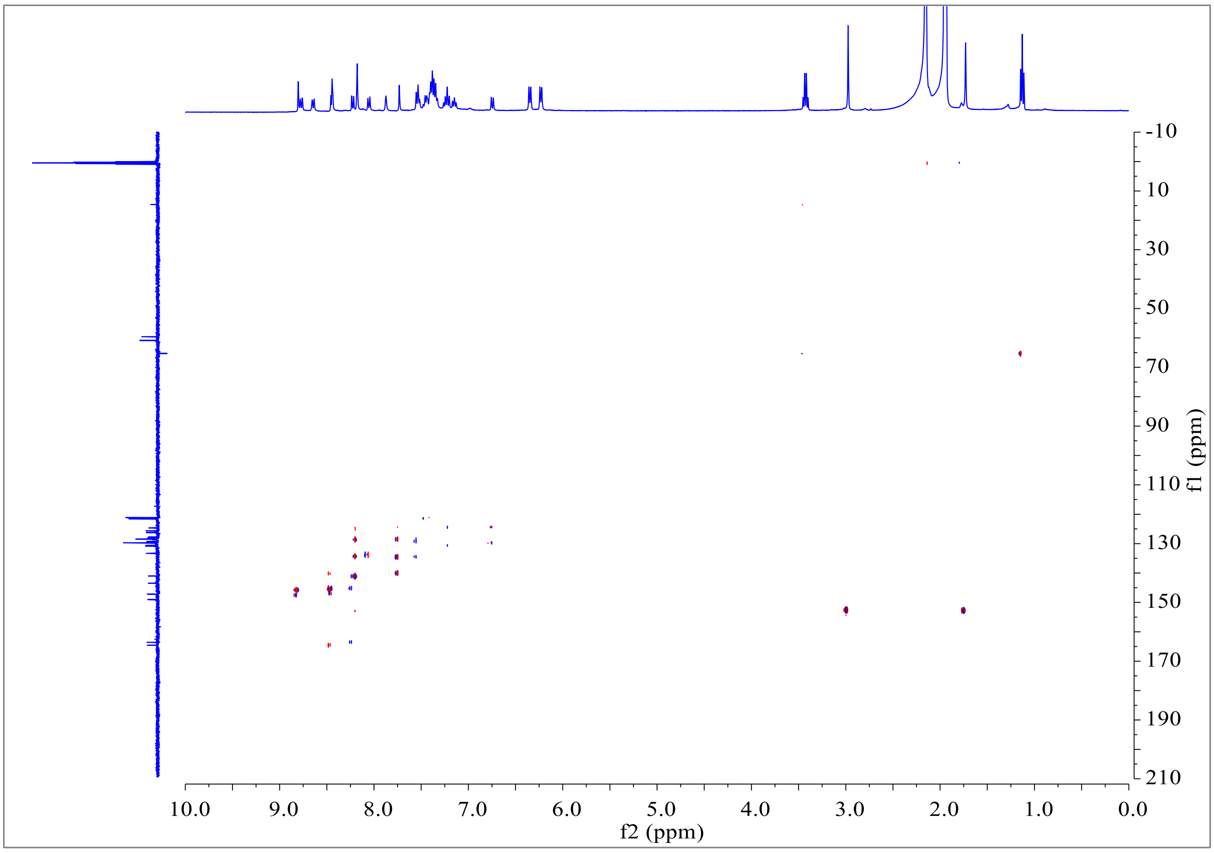

Figure S64. ¹H{¹³C} HMBC NMR spectrum of *Λ*Δ-Zn-**2** (400 MHz, CD_3CN, 298 K).

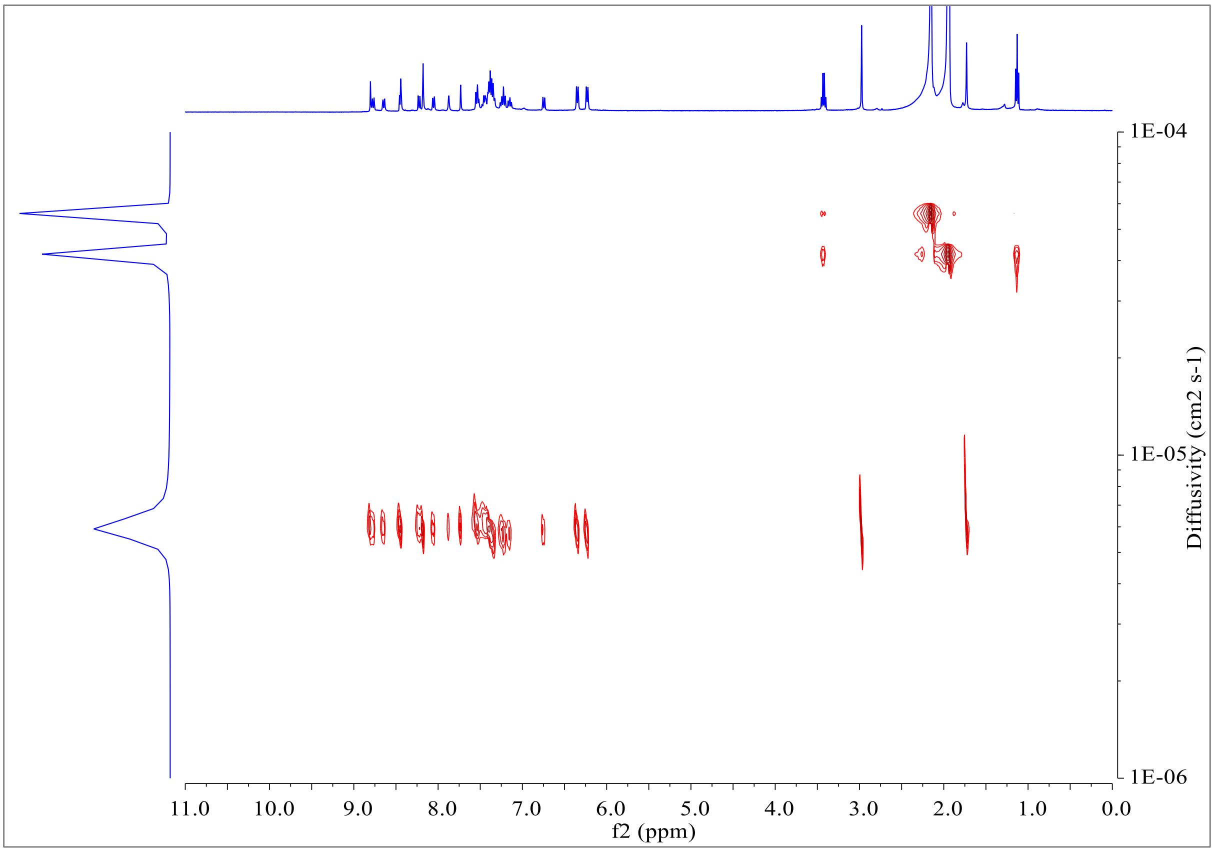

Figure S65. ^{1}H DOSY NMR spectrum of *ΛΔ*-Zn-**2** (400 MHz, CD_3CN, 298 K).

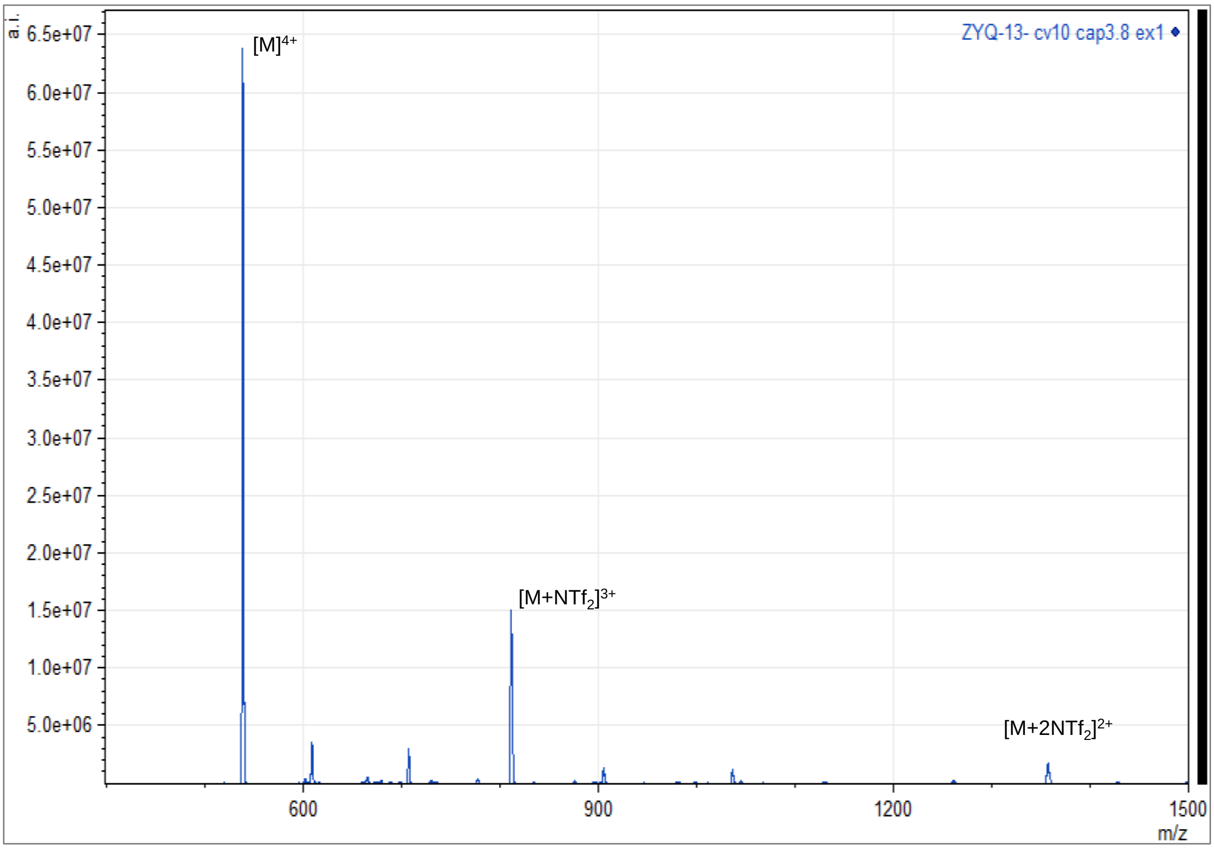

Figure S66. Low-resolution ESI-mass spectrum of *ΛΔ*-Zn-**2**.

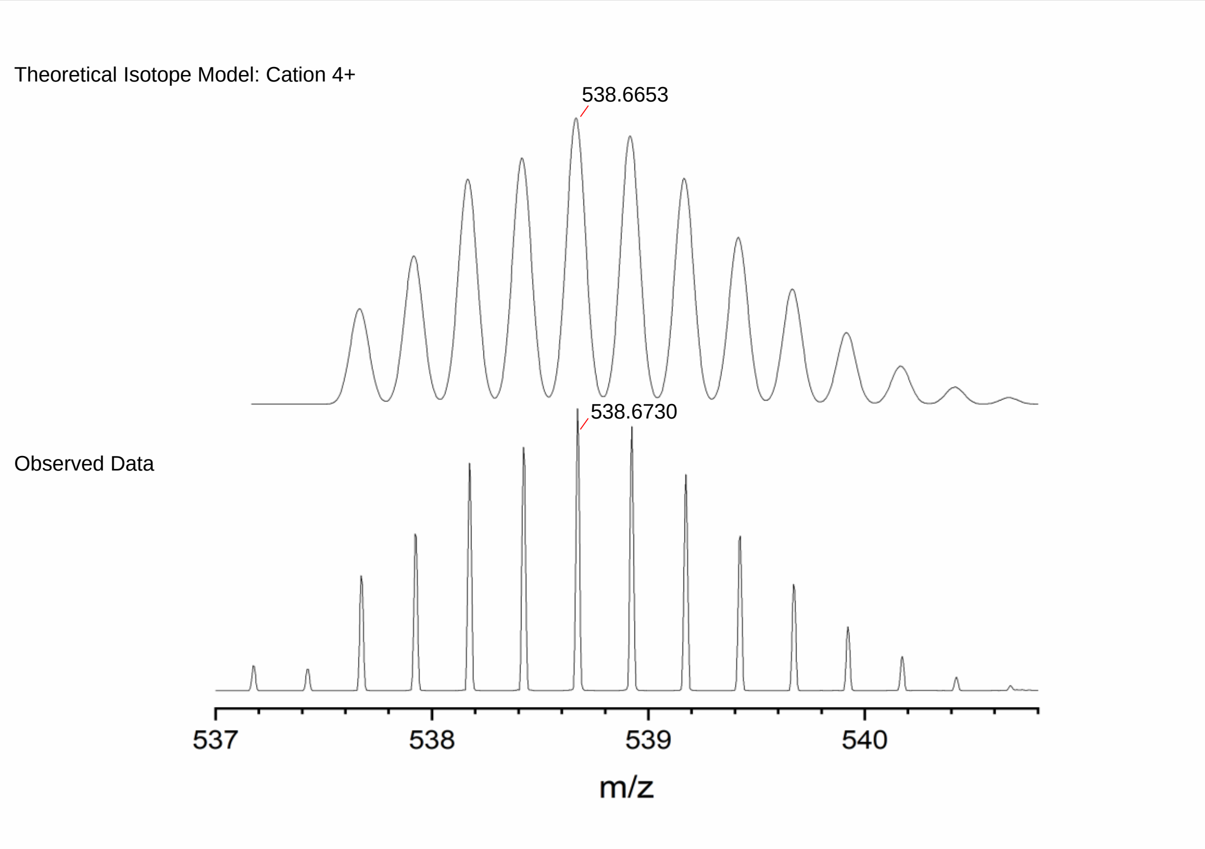

Figure S67. High-resolution ESI-mass spectral analysis of *ΛΔ*-Zn-**2** showing the +4 peak.

4.1.2 with Fe(II) salt

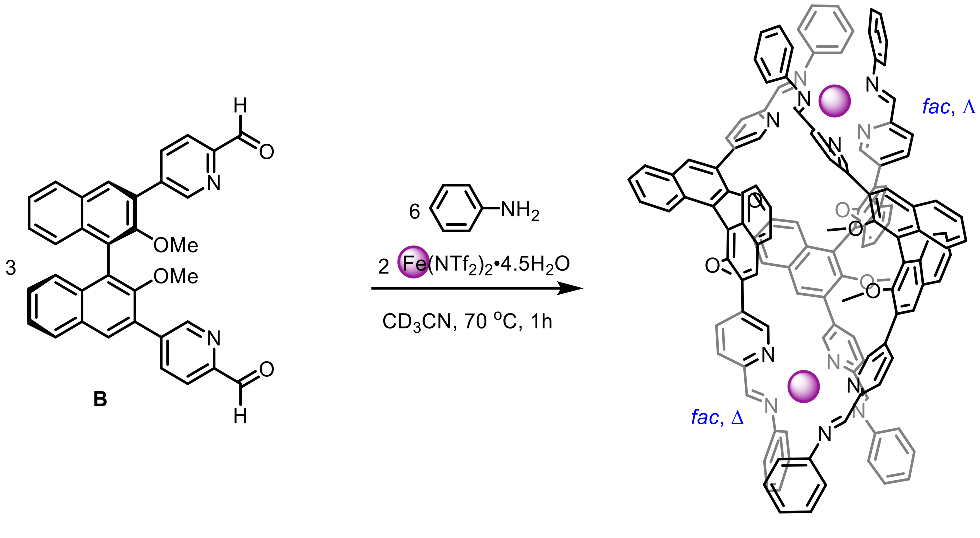

Subcomponent **B** (2.0 mg, 3.8 μmol, 1.5 equiv) was added to CD_3CN (0.6 mL) together with $Fe(NTf_2)_2$·4.5H_2O (1.7 mg, 2.5 μmol, 1.0 equiv) and aniline (0.7 mg, 7.5 μmol, 3.0 equiv). The reaction mixture was stirred at 70 °C for 1h. After cooling to room temperature, the solvent was evaporated and diethyl ether was then added. The residue was resuspended and then centrifuged and the diethyl ether decanted. This was repeated three times with fresh diethyl ether. The residue was then dried in vacuo to afford the desired product (*ΛΔ*-Fe-**2**) as a purple solid (3.4 mg, 83% yield).

Characterization of *ΛΔ*-Fe-2:

^{1}H NMR (400 MHz, CD_3CN, 298 K) δ (ppm) = 9.03 (s, 3H), 8.94 (s, 3H), 8.67 (q, *J* = 8.2 Hz, 6H), 8.55 (d, *J* = 8.0 Hz, 3H), 8.47 – 8.42 (m, 3H), 8.11 (s, 3H), 8.02 (d, *J* = 8.2 Hz, 3H), 7.81 (s, 3H), 7.69 (s, 3H), 7.63 (m, 3H), 7.54 – 7.47 (m, 9H), 7.42 – 7.24 (m, 24H), 7.17 (d, *J* = 8.5 Hz, 3H), 6.74 (d, *J* = 7.9 Hz, 3H), 5.60 (d, *J* = 7.8 Hz, 6H), 5.34 (d, *J* = 7.8 Hz, 6H), 2.85 (s, 9H), 1.72 (s, 9H).

^{13}C{^{1}H} DEPT-135 (100 MHz, CD_3CN, 298 K) δ (ppm) = 175.5, 174.8, 156.7, 153.5, 141.5, 139.9, 134.4, 132.5, 131.2, 130.5, 130.3, 129.7, 129.7, 129.4, 129.3, 129.0, 128.9, 127.0, 126.6, 126.4, 125.5, 122.4, 122.1, 61.3, 60.3.

ESI-MS *m/z* 533.8 $[M]^{4+}$, 805.1 $[M+NTf_2]^{3+}$, 1347.7 $[M+2NTf_2]^{2+}$.

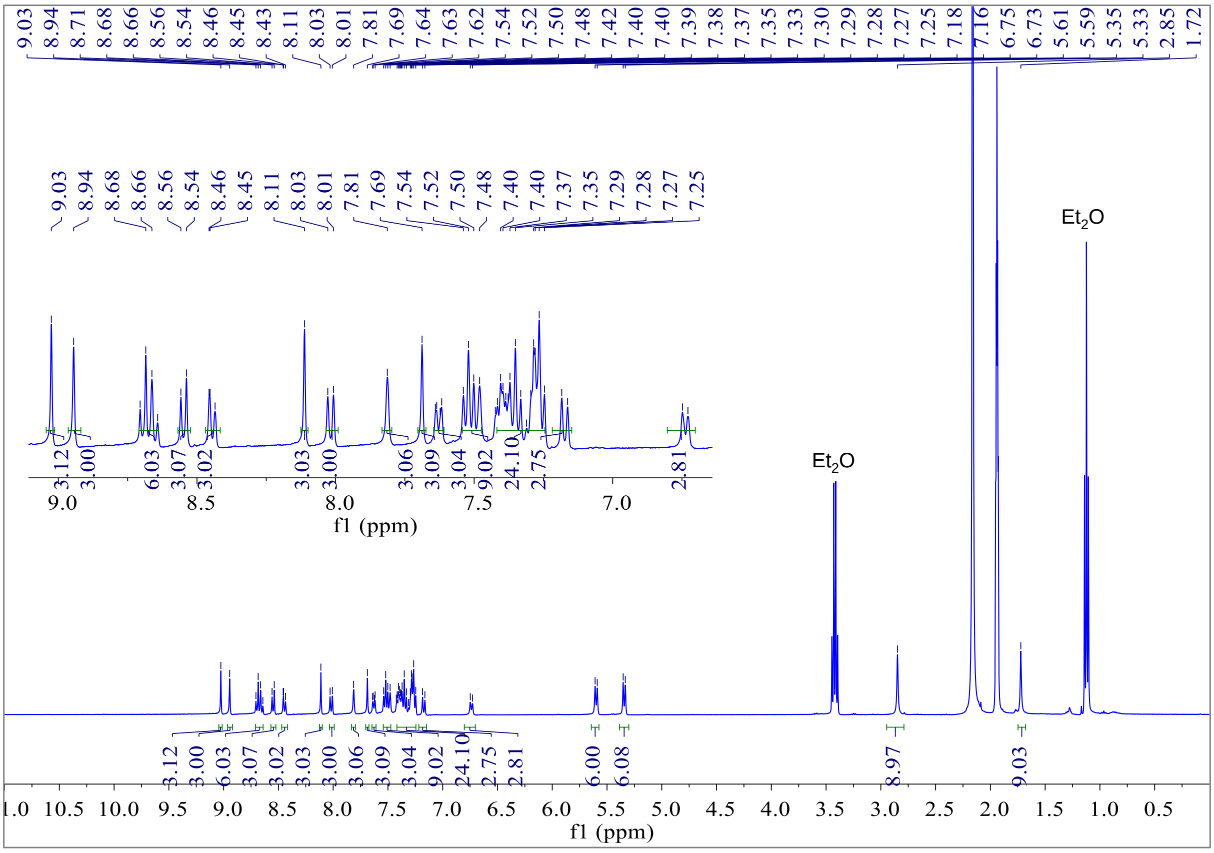

Figure S68. ^{1}H NMR spectrum of *ΛΔ*-Fe-**2** (400 MHz, CD_3CN, 298 K).

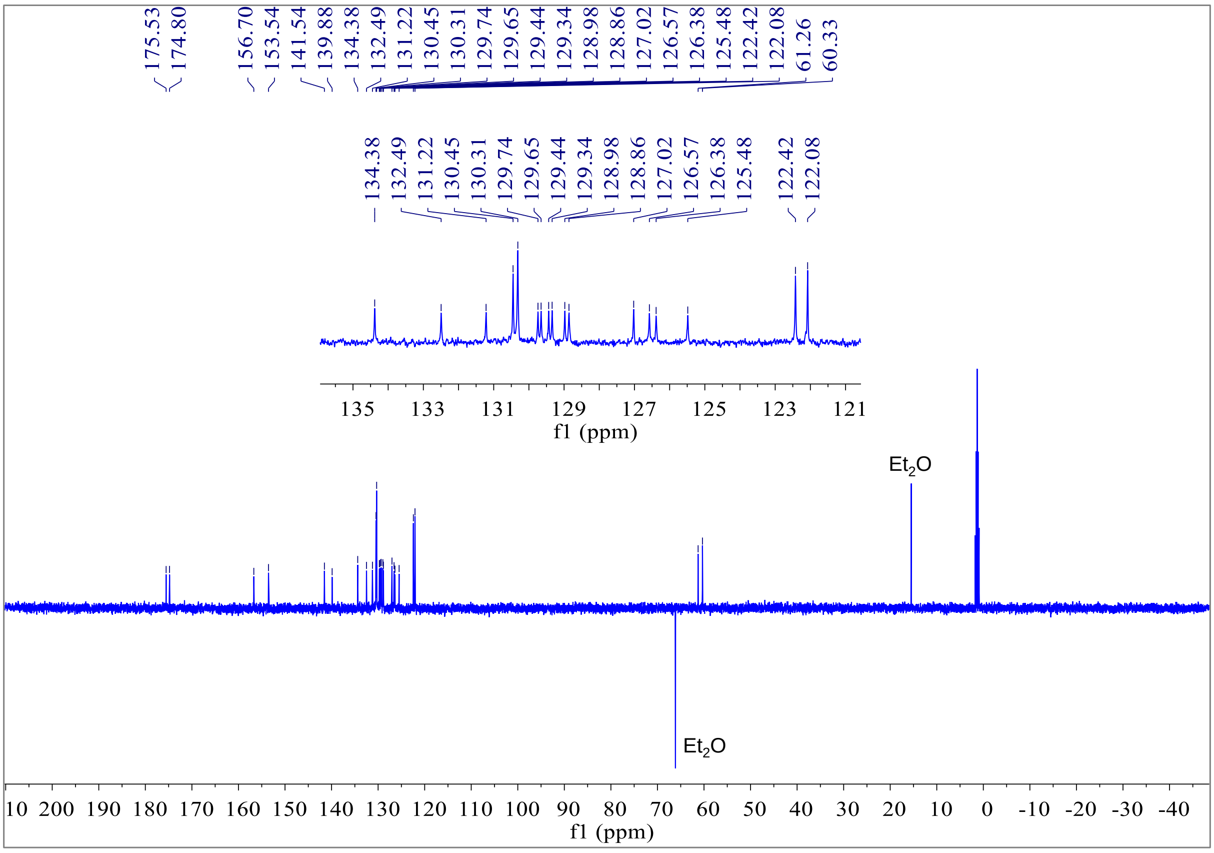

Figure S69. $^{13}C\{^{1}H\}$ DEPT-135 NMR spectrum of *ΛΔ*-Fe-**2** (100 MHz, CD_3CN, 298 K).

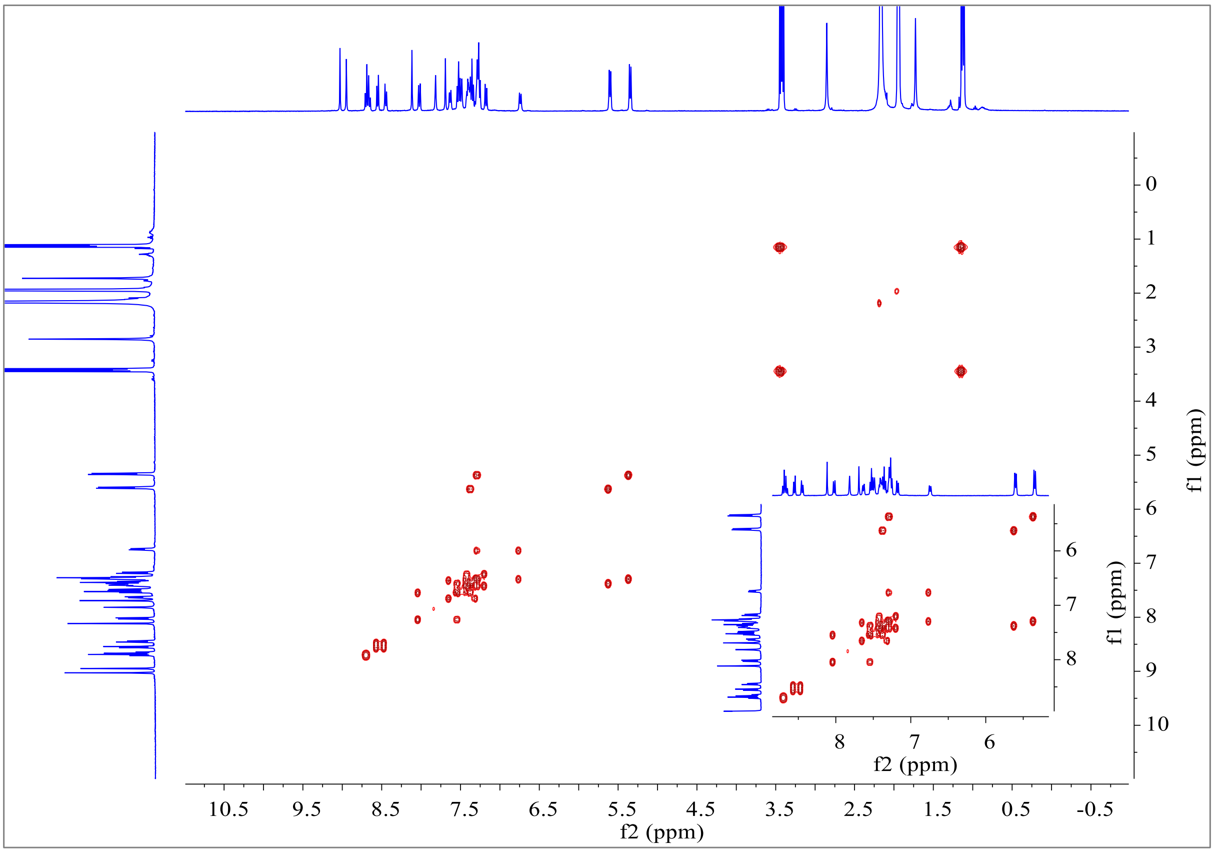

Figure S70. $^{1}H\{^{1}H\}$ COSY NMR spectrum of *ΛΔ*-Fe-**2** (400 MHz, CD_3CN, 298 K).

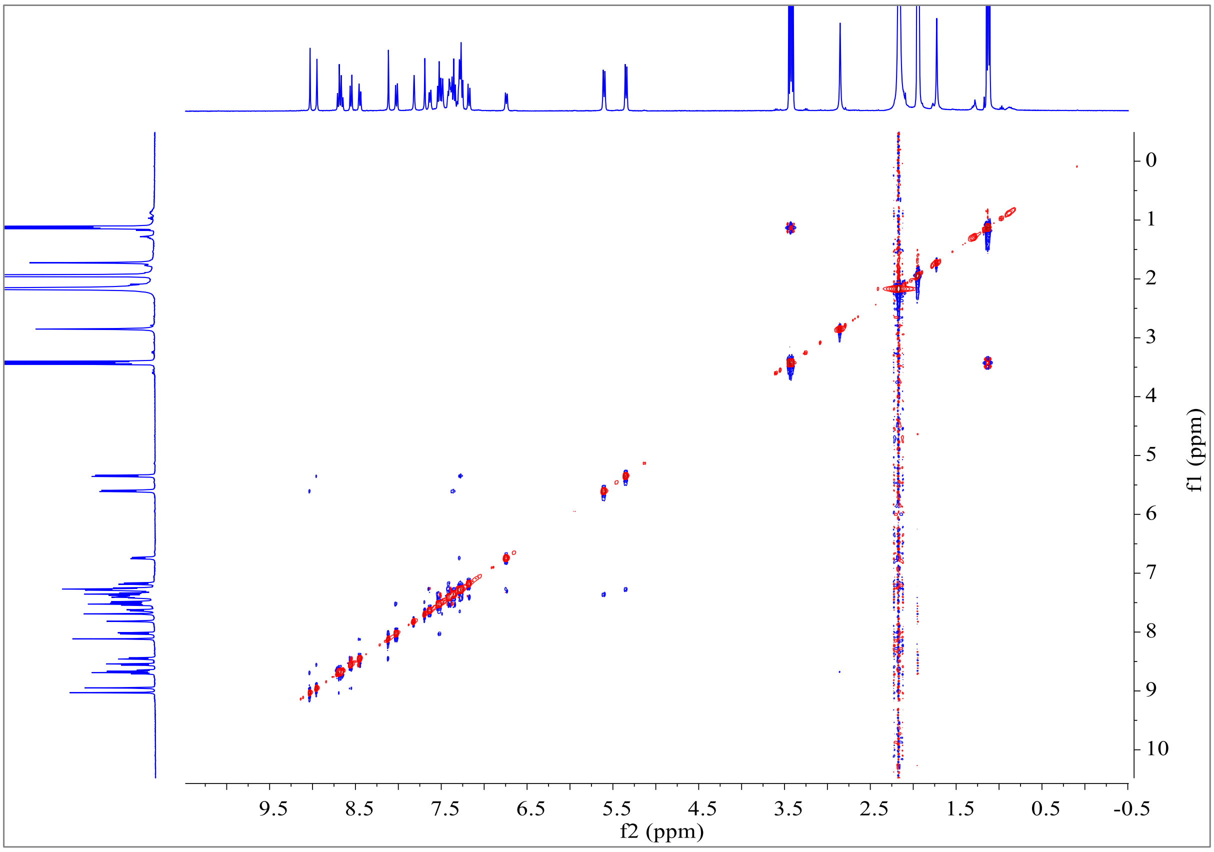

Figure S71. $^{1}H\{^{1}H\}$ NOESY NMR spectrum of *ΛΔ*-Fe-**2** (400 MHz, CD_3CN, 298 K).

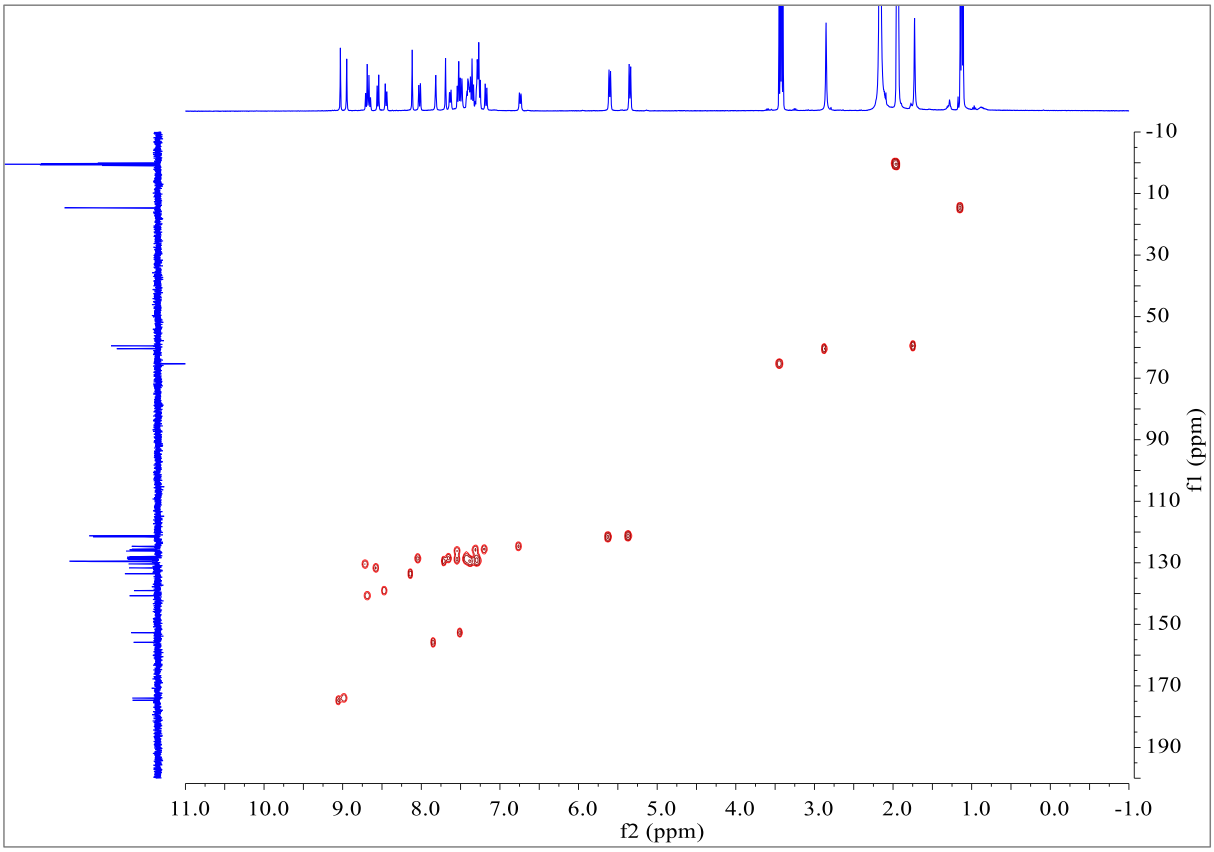

Figure S72. $^{1}H\{^{13}C\}$ HSQC NMR spectrum of *ΛΔ*-Fe-**2** (400 MHz, CD_3CN, 298 K).

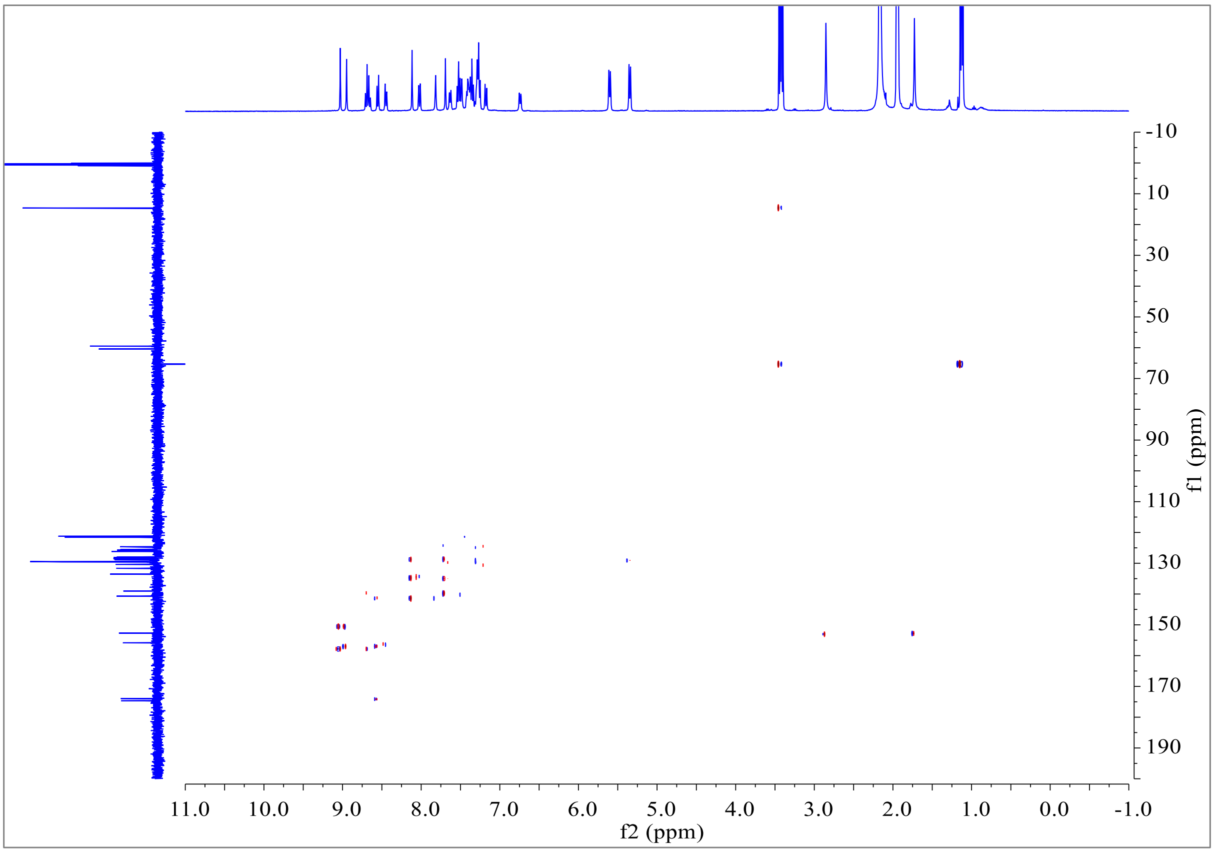

Figure S73. $^{1}H\{^{13}C\}$ HMBC NMR spectrum of *ΛΔ*-Fe-**2** (400 MHz, CD_3CN, 298 K).

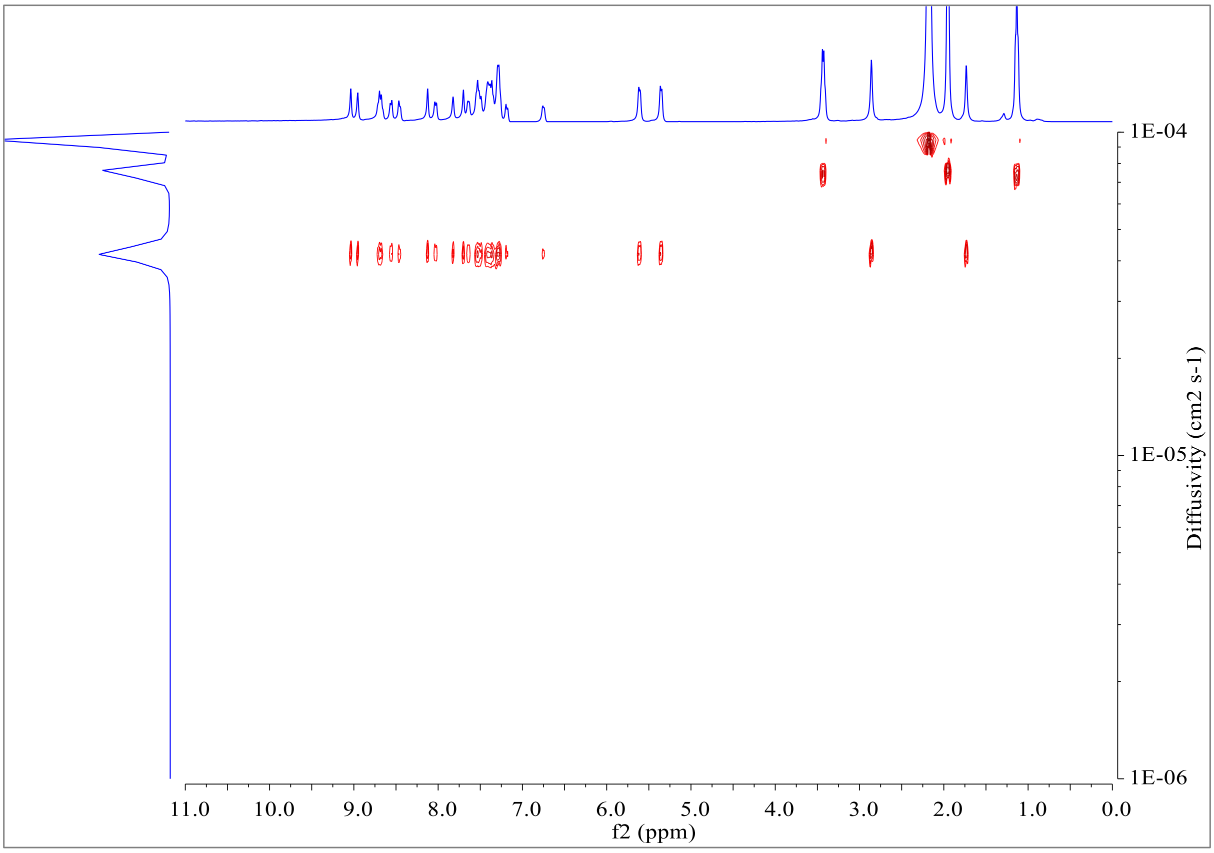

Figure S74. ^{1}H DOSY NMR spectrum of *ΛΔ*-Fe-**2** (400 MHz, CD_3CN, 298 K).

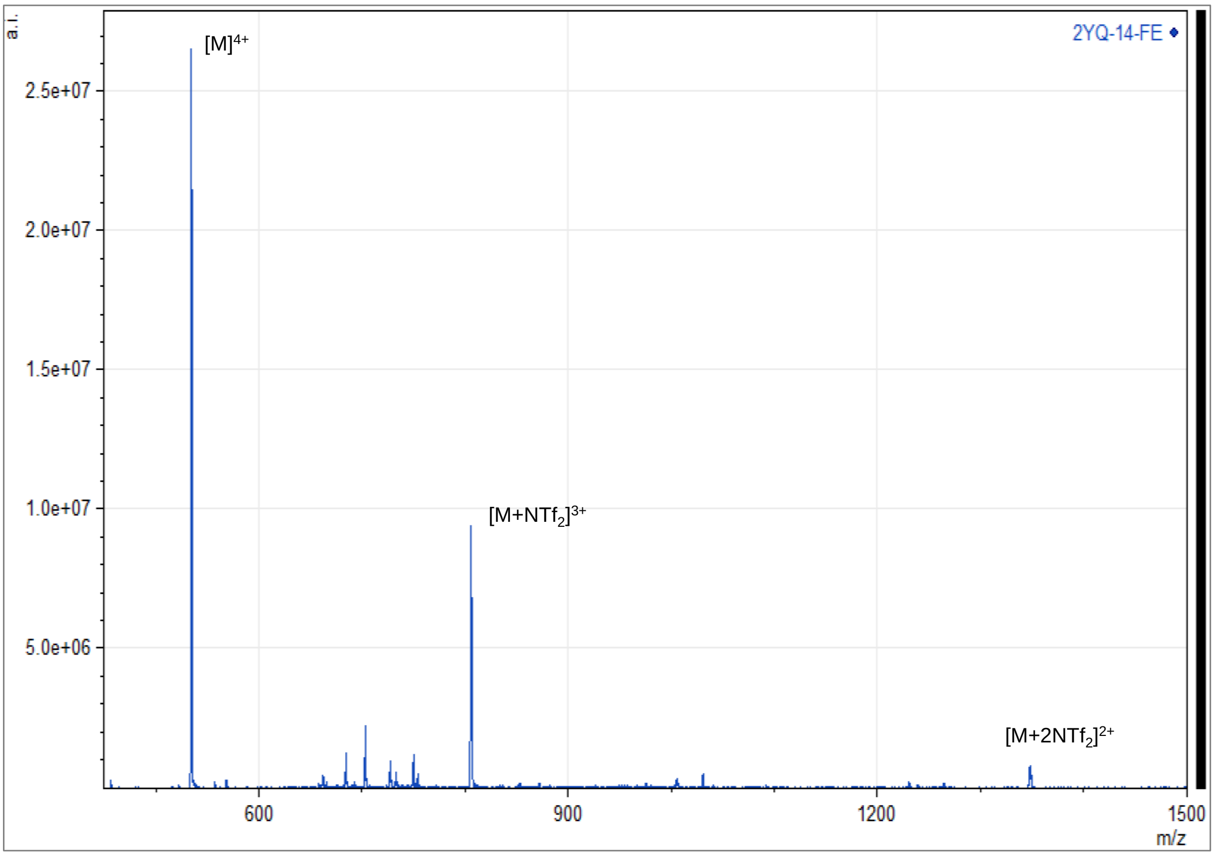

Figure S75. Low-resolution ESI-mass spectrum of *ΛΔ*-Fe-**2**.

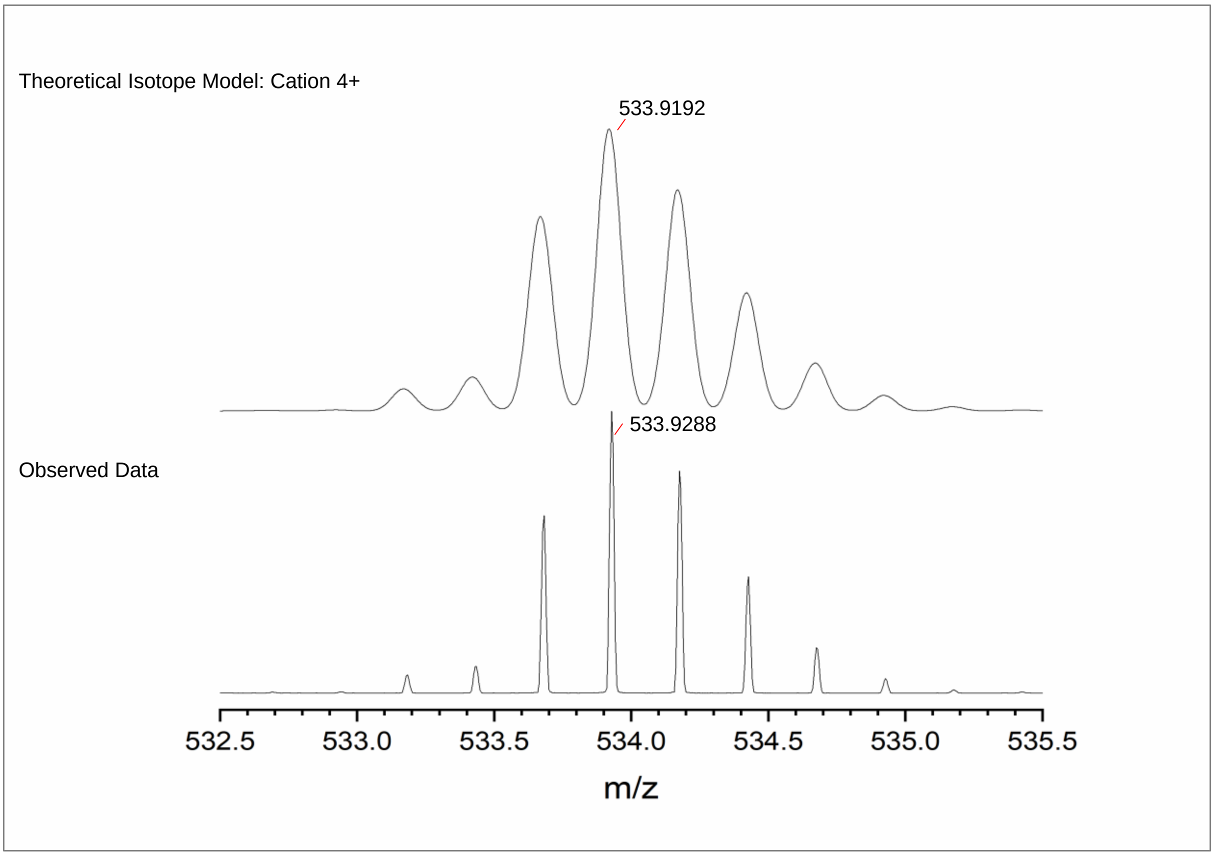

Figure S76. High-resolution ESI-mass spectrometral analysis of *ΛΔ*-Fe-**2** showing the +4 peak.

4.1.3 with Co(II) salt

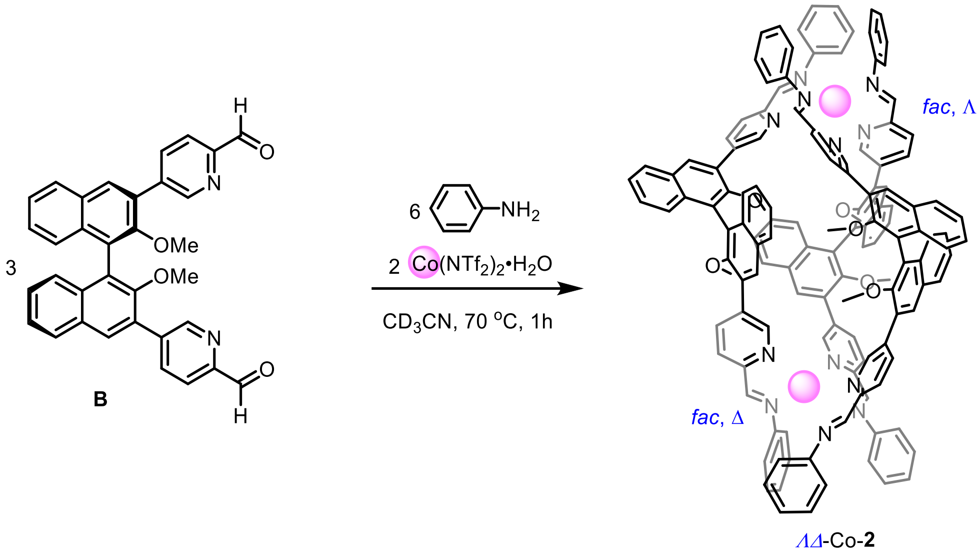

Subcomponent **B** (2.0 mg, 3.8 μmol, 1.5 equiv) was added to CD_3CN (0.6 mL) together with $Co(NTf_2)_2 \cdot H_2O$ (1.6 mg, 2.5 μmol, 1.0 equiv) and aniline (0.7 mg, 7.5 μmol, 3.0 equiv). The reaction mixture was stirred at 70 °C for 1h. After cooling to room temperature, the solvent was evaporated and diethyl ether was then added. The residue was resuspended and then centrifuged and the diethyl ether decanted. This was repeated three times with fresh diethyl ether. The residue was then dried in vacuo to afford the desired product (*ΛΔ*-Co-**2**) as a pale yellow solid (3.5 mg, 85% yield).

Characterization of *ΛΔ*-Co-**2:**

^{1}H NMR (400 MHz, CD_3CN, 298 K) δ (ppm) = 245.5, 238.0, 95.4, 86.7, 73.3, 70.3, 13.3, 12.6, 10.3, 9.9, 9.4, 8.4, 7.1, 7.0, 6.6, 5.4, 4.8, 4.1, -6.2, -11.3, -13.6, -18.5, -24.6, -25.9.

ESI-MS *m/z* 533.8 $[M]^{4+}$, 805.1 $[M+NTf_2]^{3+}$, 1347.7 $[M+2NTf_2]^{2+}$.

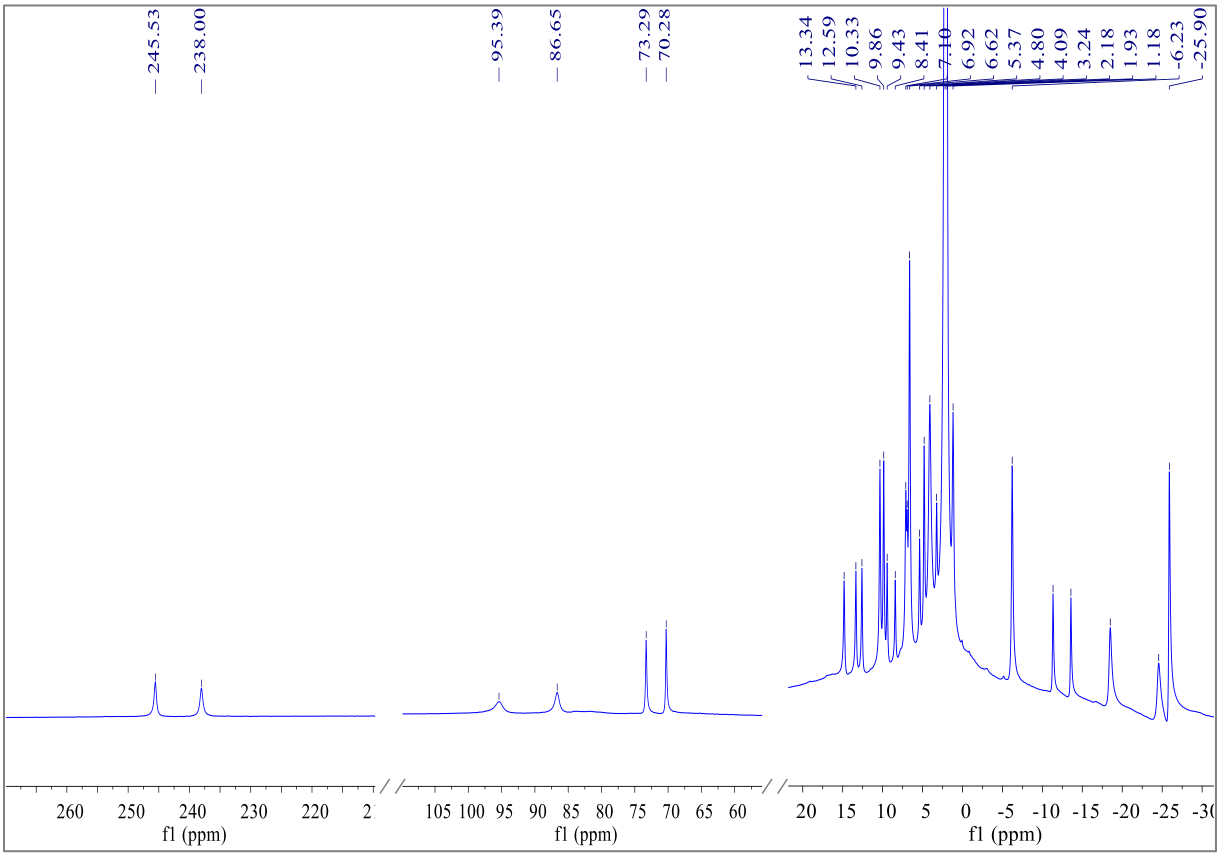

Figure S77. ^{1}H NMR spectrum of *ΛΔ*-Co-**2** (400 MHz, CD_3CN, 298 K).

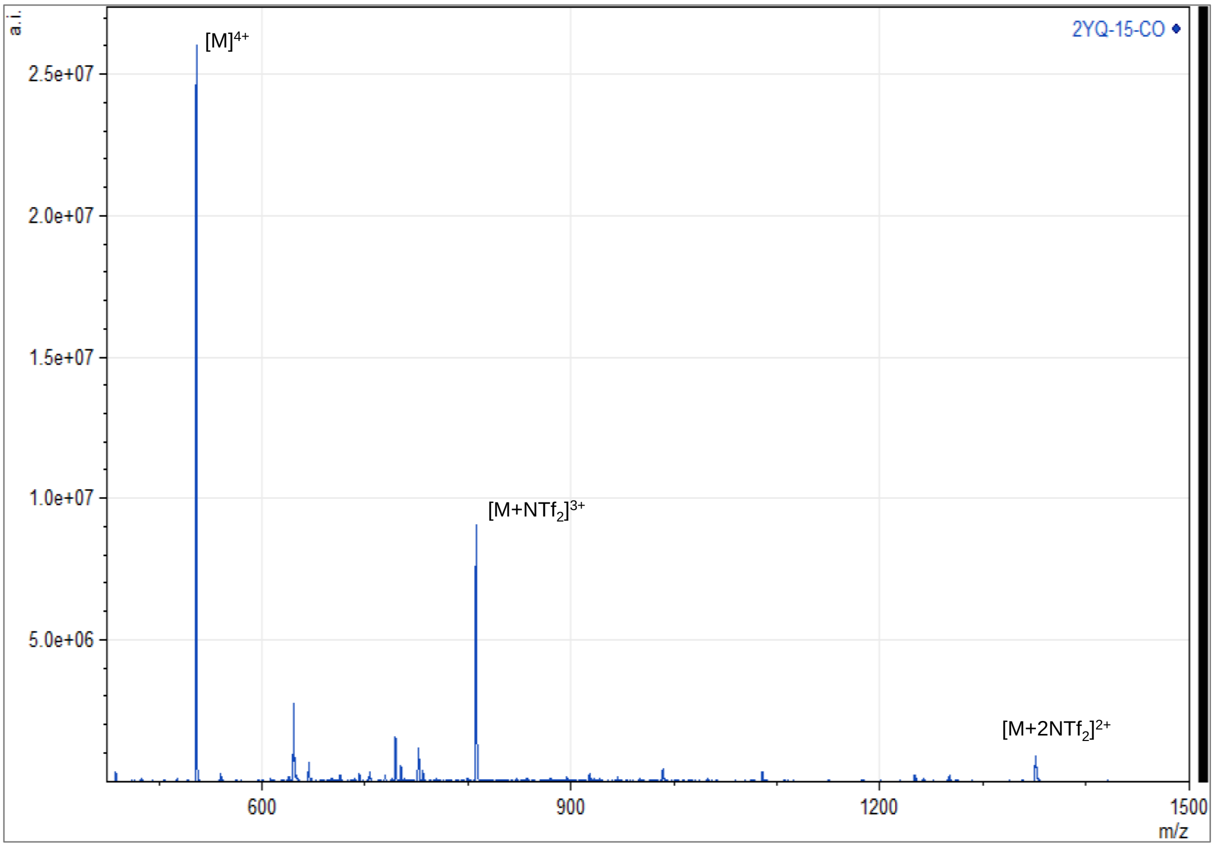

Figure S78. Low-resolution ESI-mass spectrum of *ΛΔ*-Co-**2**.

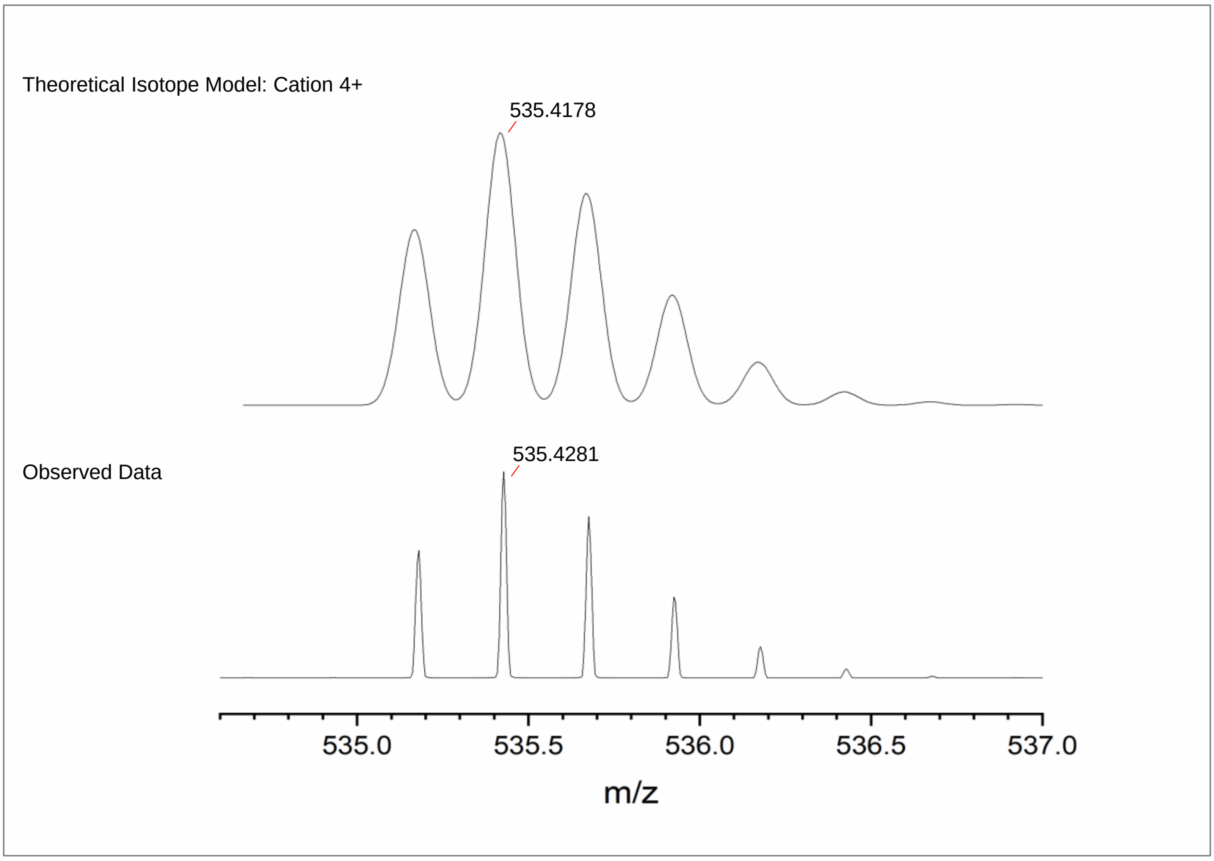

Figure S79. High-resolution ESI-mass spectrometral analysis of *ΛΔ*-Co-**2** showing the +4 peak.

4.2 Mixed subcomponents

4.2.1 using subcomponents B and C with Fe(II) salt

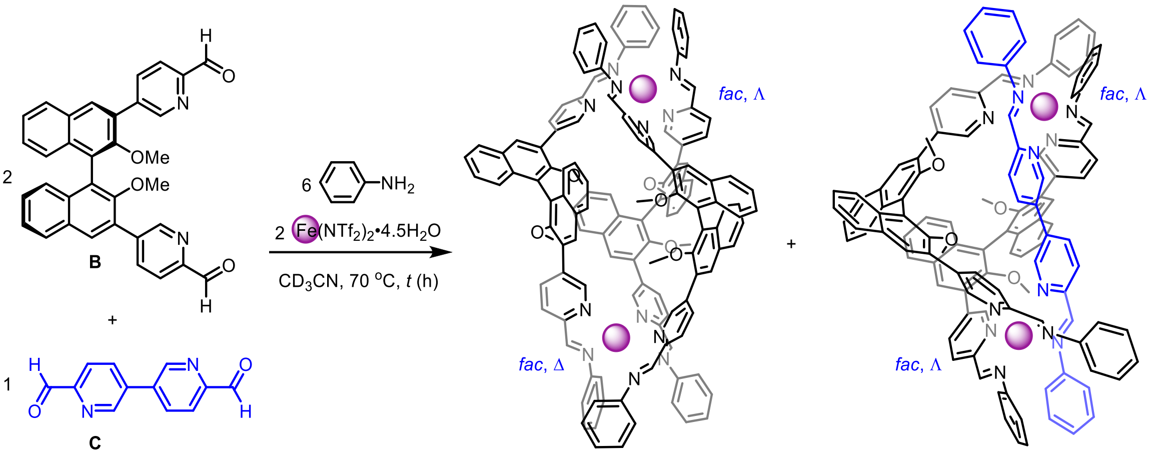

ΛΔ-Fe-**2** ΛΛ-Fe-**4**

Subcomponents **B** (1.3 mg, 2.5 μmol, 1.0 equiv) and **C** (0.3 mg, 1.25 μmol, 0.5 equiv) were added to CD_3CN (0.6 mL) together with $Fe(NTf_2)_2$·$4.5H_2O$ (1.7 mg, 2.5 μmol, 1.0 equiv) and aniline (0.7 mg, 7.5 μmol, 3.0 equiv). The reaction mixture was stirred at 70 °C and monitored by ^{1}H NMR. As shown in Figure S80, a new species (red, triangle) appearing at δ = 5.94 (d), 5.45 (d), 5.13 (d), 2.79 (s) and 1.89 (s) ppm was formed as the major product after 1 hour. The structure of this new formed species was futher confirmed to be the *ΛΛ*-Fe-**4**. It is worth mentioning that only a small amout of *ΛΔ*-Fe-**2** was generated within 1 hour (blue, square). Interestingly, as the reaction proceeded, *ΛΔ*-Fe-**2** gradually converted to *ΛΛ*-Fe-**4**. After 96 hours, *ΛΔ*-Fe-**2** had almost fully converted to *ΛΛ*-Fe-**4**. After cooling to room temperature, the solvent was evaporated and diethyl ether was then added. The residue resuspended and then centrifuged and the diethyl ether decanted. This was repeated three times with fresh diethyl ether. The residue was then dried in vacuo to afford the desired product (*ΛΛ*-Fe-**4**) as a purple solid (3.0 mg, 81% yield).

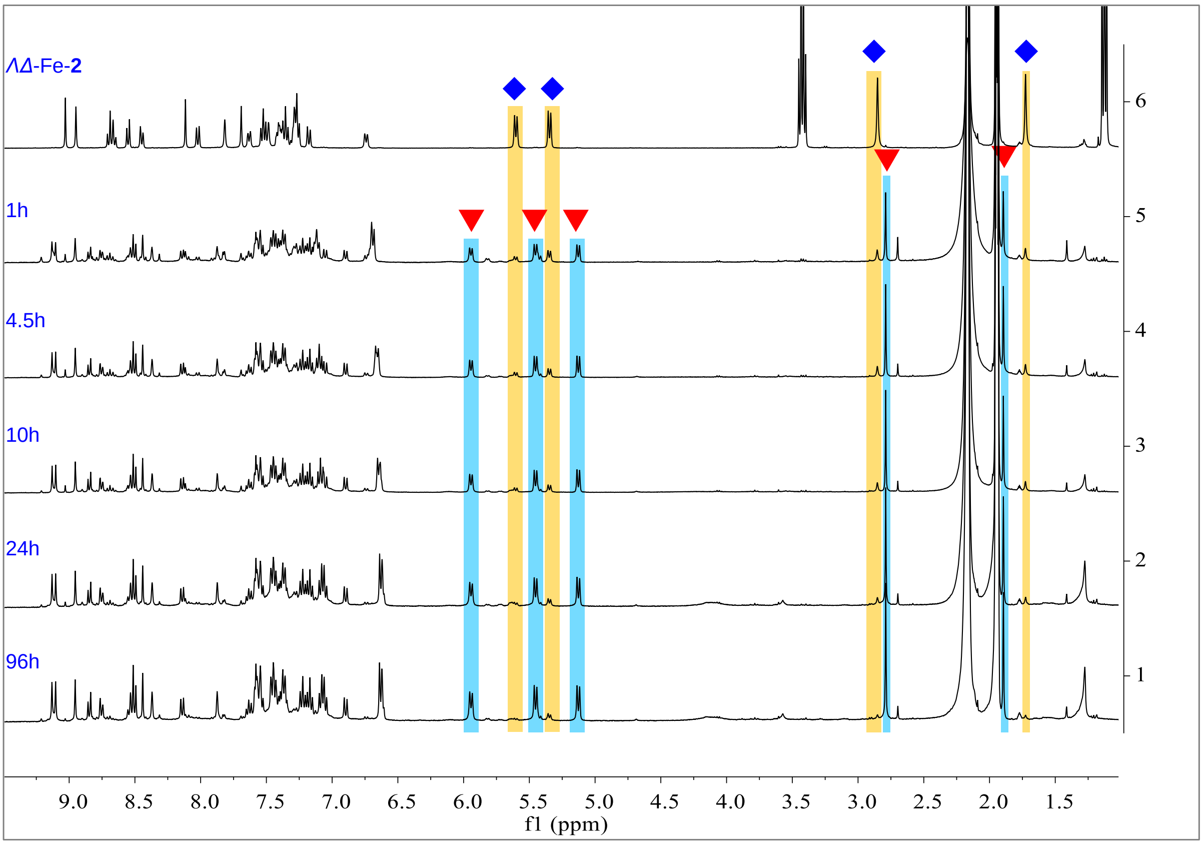

Figure S80. Crude ^{1}H NMR spectrum of self-assembly of **B**, **C** and aniline with $Fe(NTf_2)_2$ (400 MHz, CD_3CN, 298 K) (red triangle: *ΛΛ*-Fe-**4**; blue quare: *ΛΔ*-Fe-**2**).

Characterization of *ΛΛ*-Fe-4:

^{1}H NMR (400 MHz, CD_3CN, 298 K) δ (ppm) = 9.13 (s, 2H), 9.10 (s, 2H), 8.95 (s, 2H), 8.84 (d, *J* = 8.0 Hz, 2H), 8.75 (d, *J* = 7.9 Hz, 2H), 8.52 (d, *J* = 8.2 Hz, 4H), 8.43 (s, 2H), 8.36 (s, 2H), 8.14 (d, *J* = 8.2 Hz, 2H), 7.87 (s, 2H), 7.58 – 7.52 (m, 10H), 7.41 (ddd, *J* = 27.6, 11.1, 6.7 Hz, 18H), 7.23 (d, *J* = 7.8 Hz, 4H), 7.15 (d, *J* = 7.6 Hz, 4H), 7.05 (d, *J* = 8.6 Hz, 2H), 6.89 (d, *J* = 8.6 Hz, 2H), 5.94 (d, *J* = 7.8 Hz, 4H), 5.45 (d, *J* = 7.8 Hz, 4H), 5.12 (d, *J* = 7.8 Hz, 4H), 2.78 (s, 6H), 1.89 (s, 6H).

^{13}C NMR (125 MHz, CD_3CN, 298 K) δ (ppm) = 174.8, 174.0, 172.6, 159.0, 157.5, 156.3, 156.2, 154.4, 152.5, 152.4, 152.1, 151.2, 150.4, 149.8, 142.7, 140.7, 140.1, 139.1, 135.8, 134.5, 134.0, 133.5, 132.7, 131.7, 131.6, 131.5, 131.2, 130.5, 130.1, 129.7, 129.5, 129.5, 129.2, 129.1, 129.0, 128.9, 128.8, 128.5, 128.3, 127.1, 127.0, 126.1, 125.0, 124.9, 124.5, 123.1, 122.1, 121.7, 121.2, 120.8, 60.9, 60.8.

ESI-MS *m/z* 455.9 $[M]^{4+}$, 701.3 $[M+NTf_2]^{3+}$, 1192.1 $[M+2NTf_2]^{2+}$.

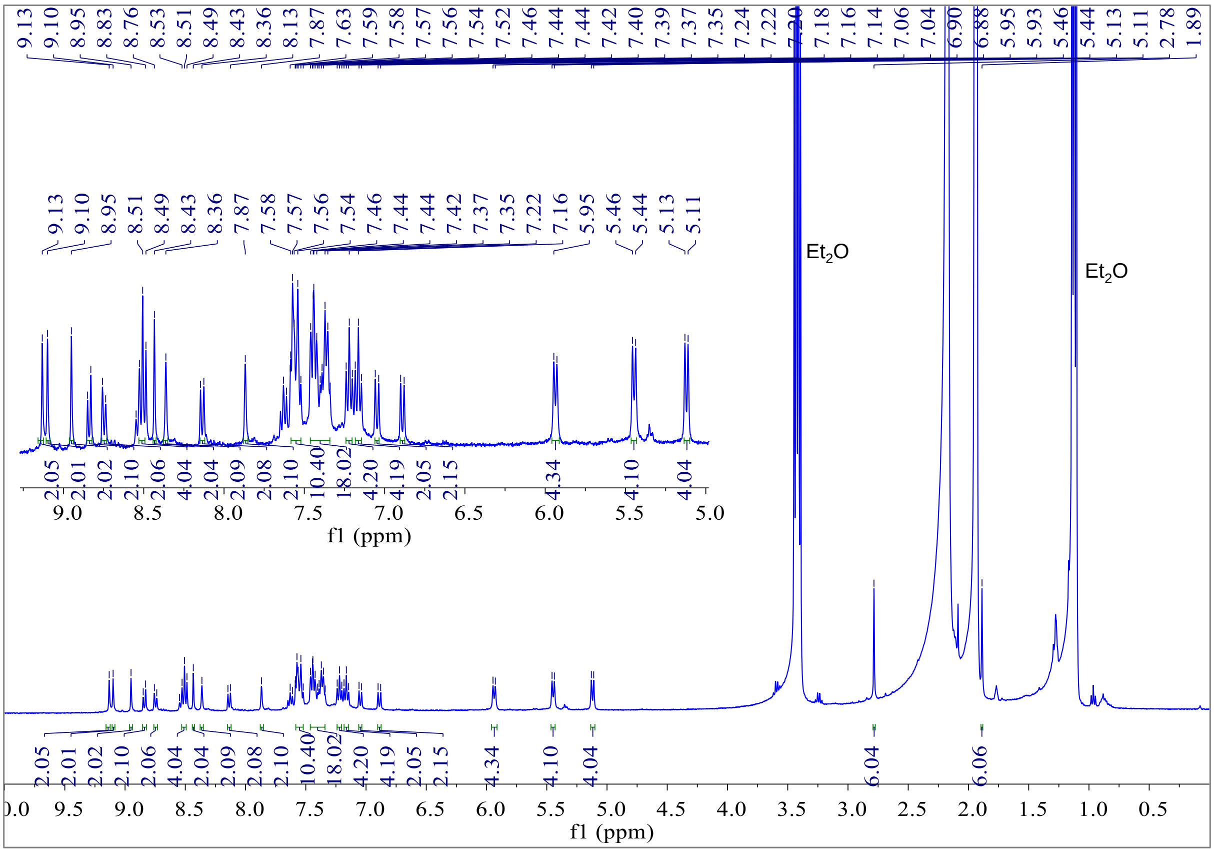

Figure S81. ^1H NMR spectrum of *ΛΛ*-Fe-**4** (400 MHz, CD_3CN, 298 K).

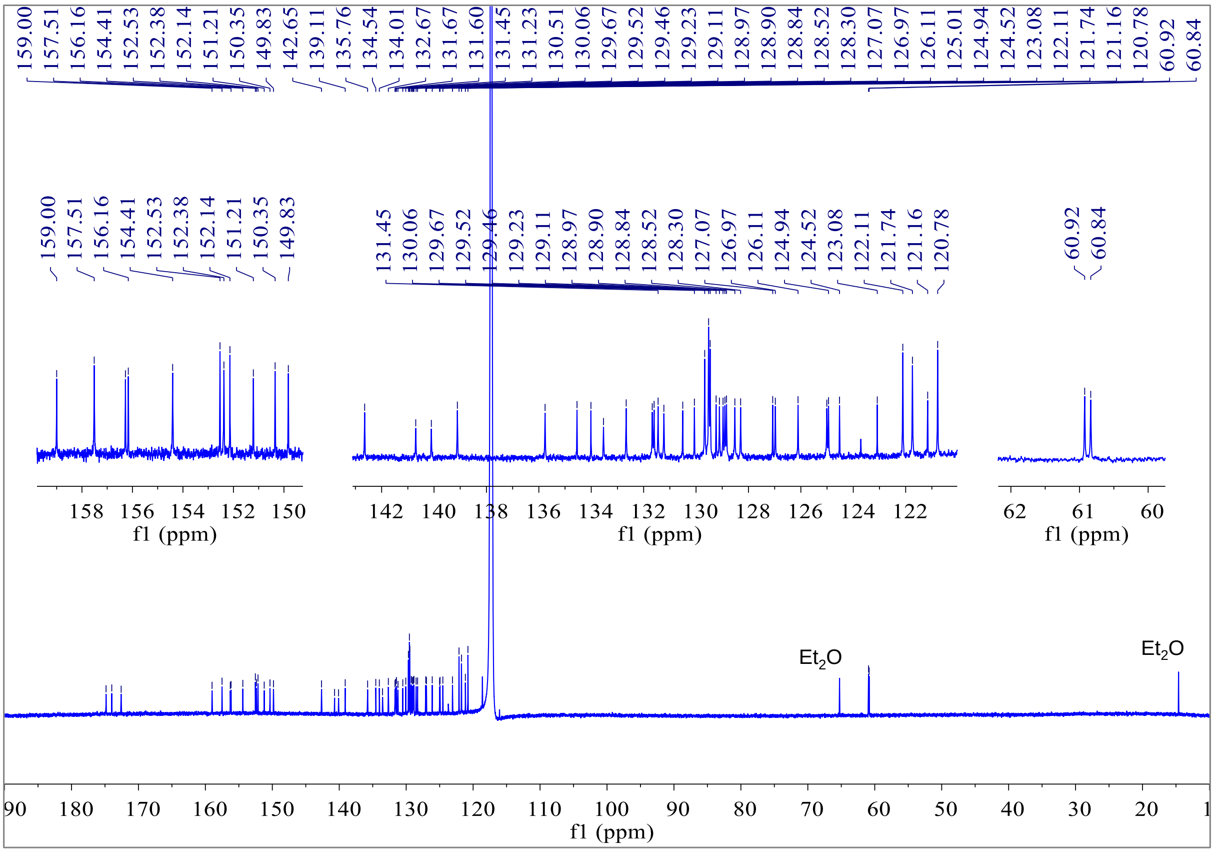

Figure S82. ^{13}C NMR spectrum of *ΛΛ*-Fe-**4** (125 MHz, CD_3CN, 298 K).

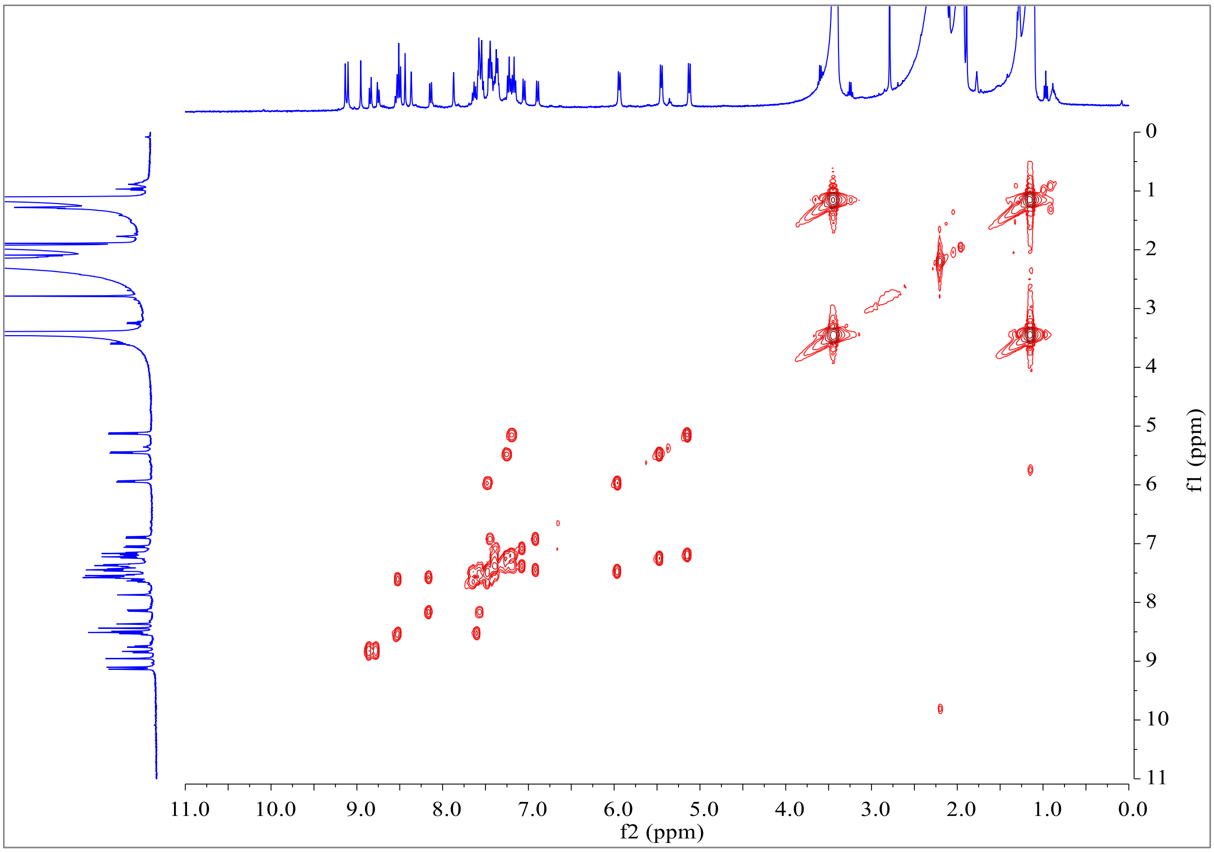

Figure S83. $^{1}H\{^{1}H\}$ COSY NMR spectrum of *ΛΛ*-Fe-**4** (400 MHz, CD_3CN, 298 K).

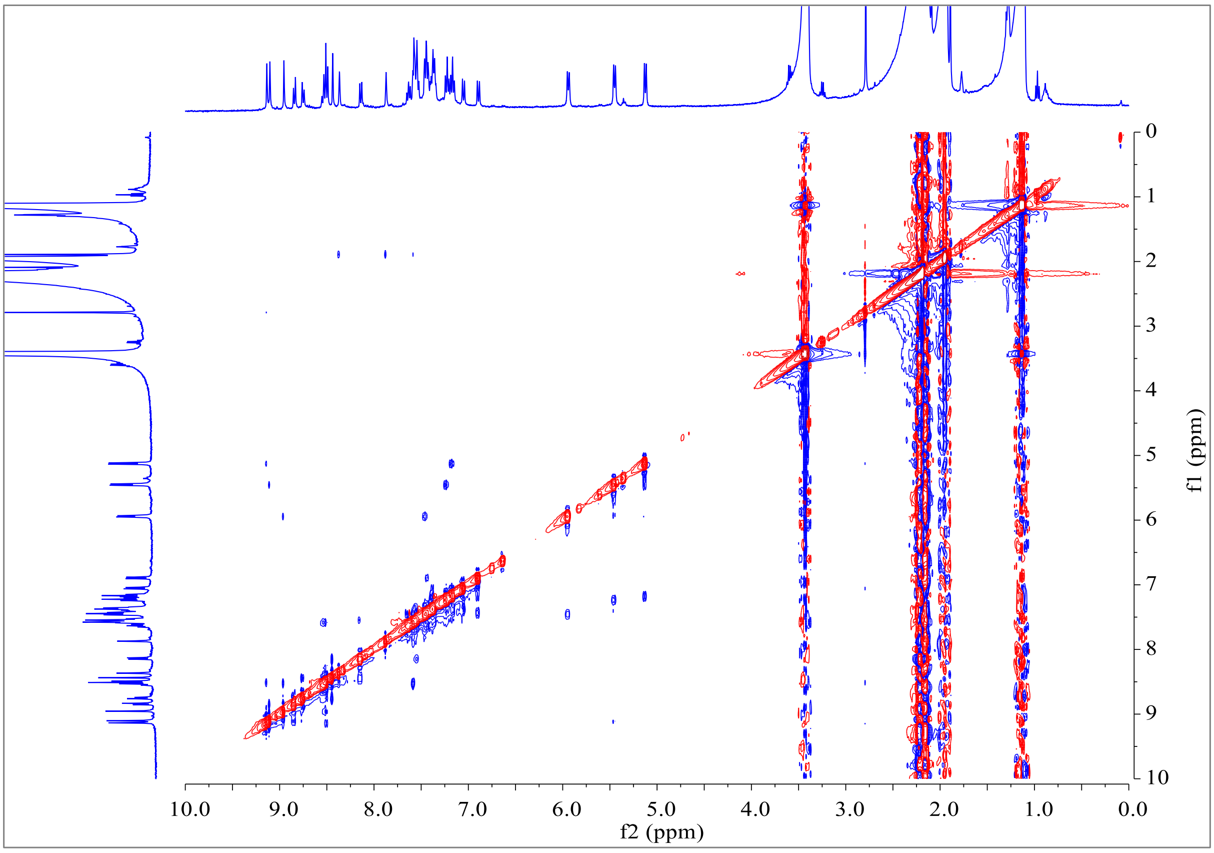

Figure S84. $^{1}H\{^{1}H\}$ NOESY NMR spectrum of *ΛΛ*-Fe-**4** at (400 MHz, CD_3CN, 298 K).

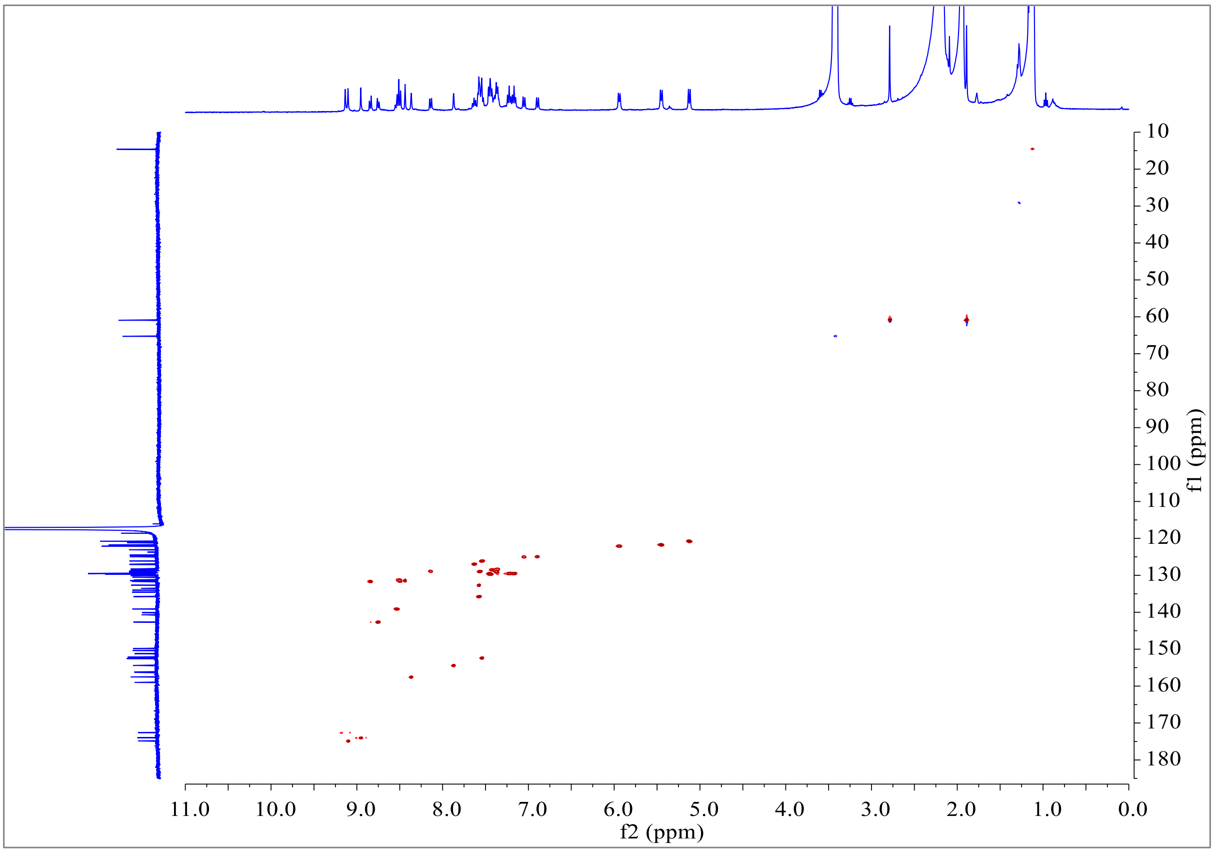

Figure S85. $^{1}H\{^{13}C\}$ HSQC NMR spectrum of *ΛΛ*-Fe-**4** (500 MHz, CD_3CN, 298 K).

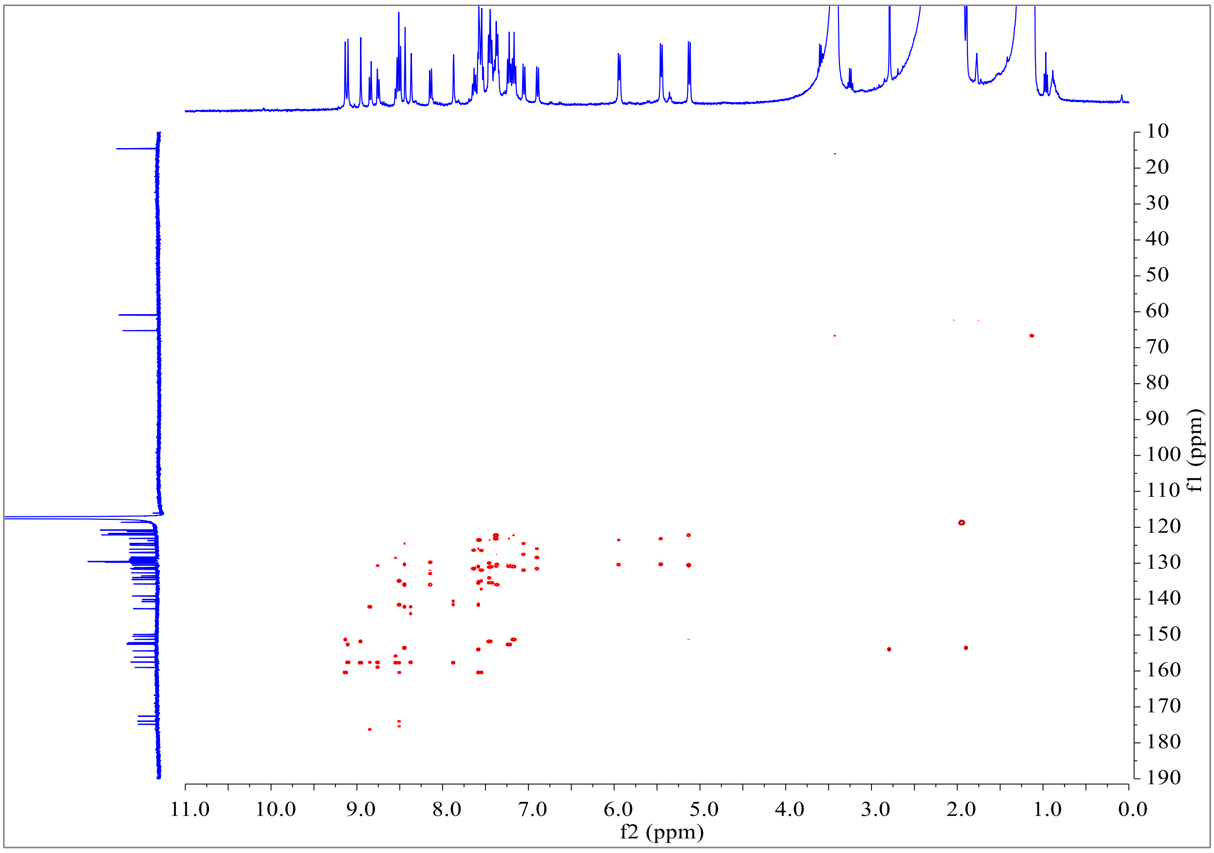

Figure S86. $^{1}H\{^{13}C\}$ HMBC NMR spectrum of *ΛΛ*-Fe-**4** (500 MHz, CD_3CN, 298 K).

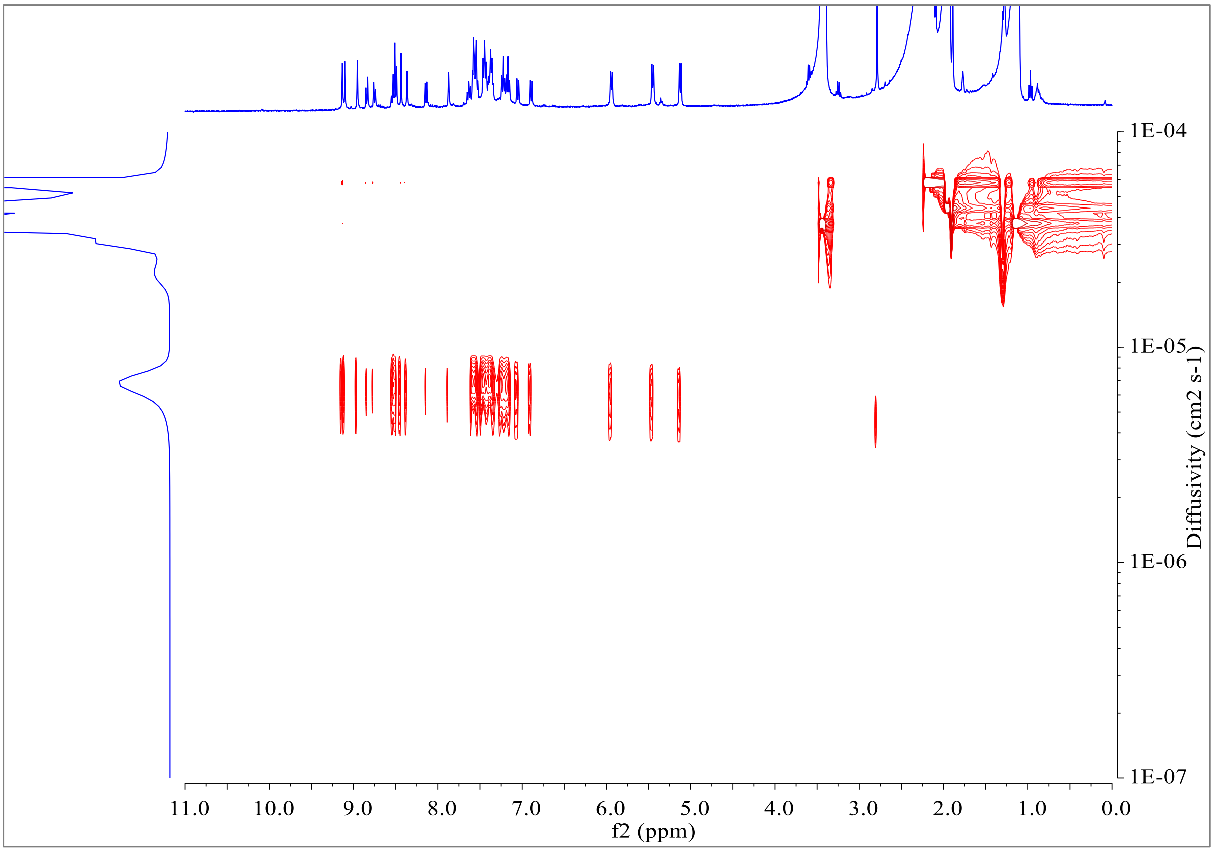

Figure S87. ^{1}H DOSY NMR spectrum of *ΛΛ*-Fe-**4** (400 MHz, CD_3CN, 298 K).

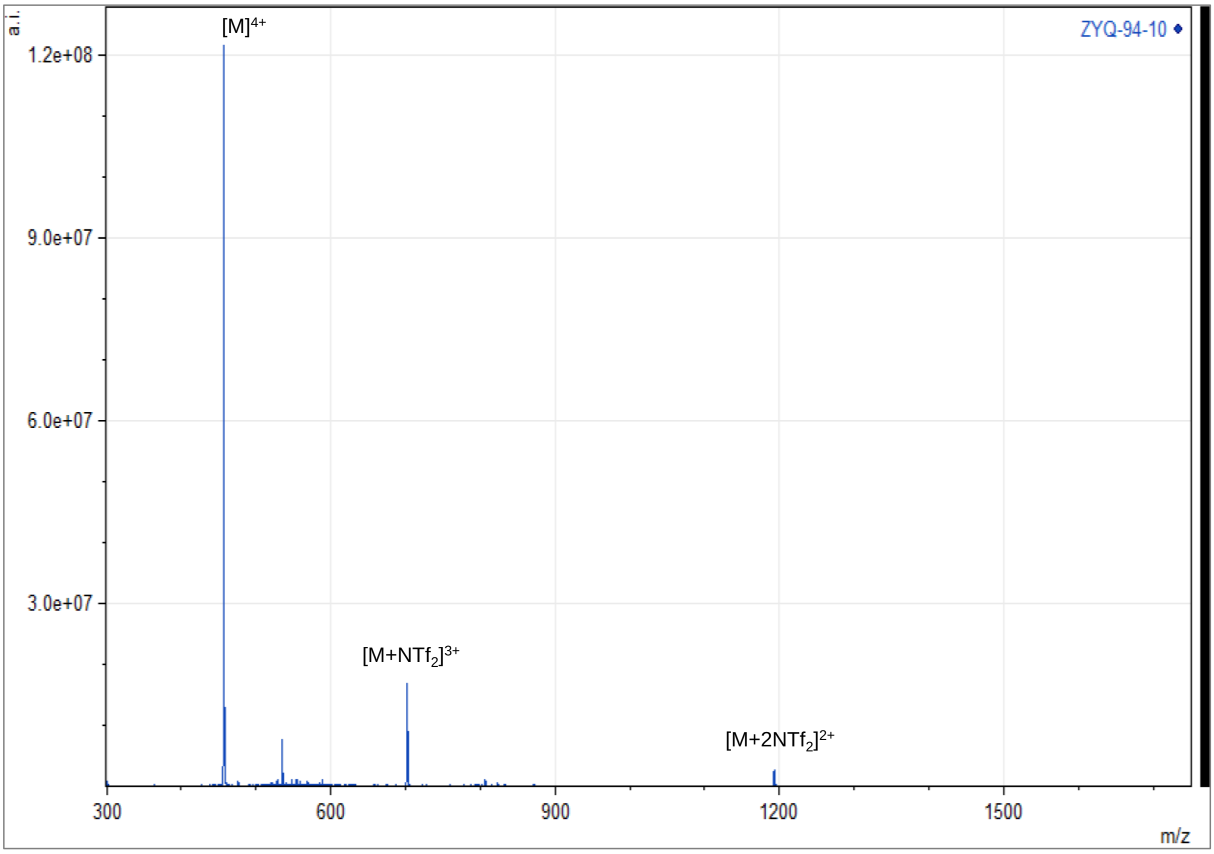

Figure S88. Low-resolution ESI-mass spectrum of *ΛΛ*-Fe-**4**.

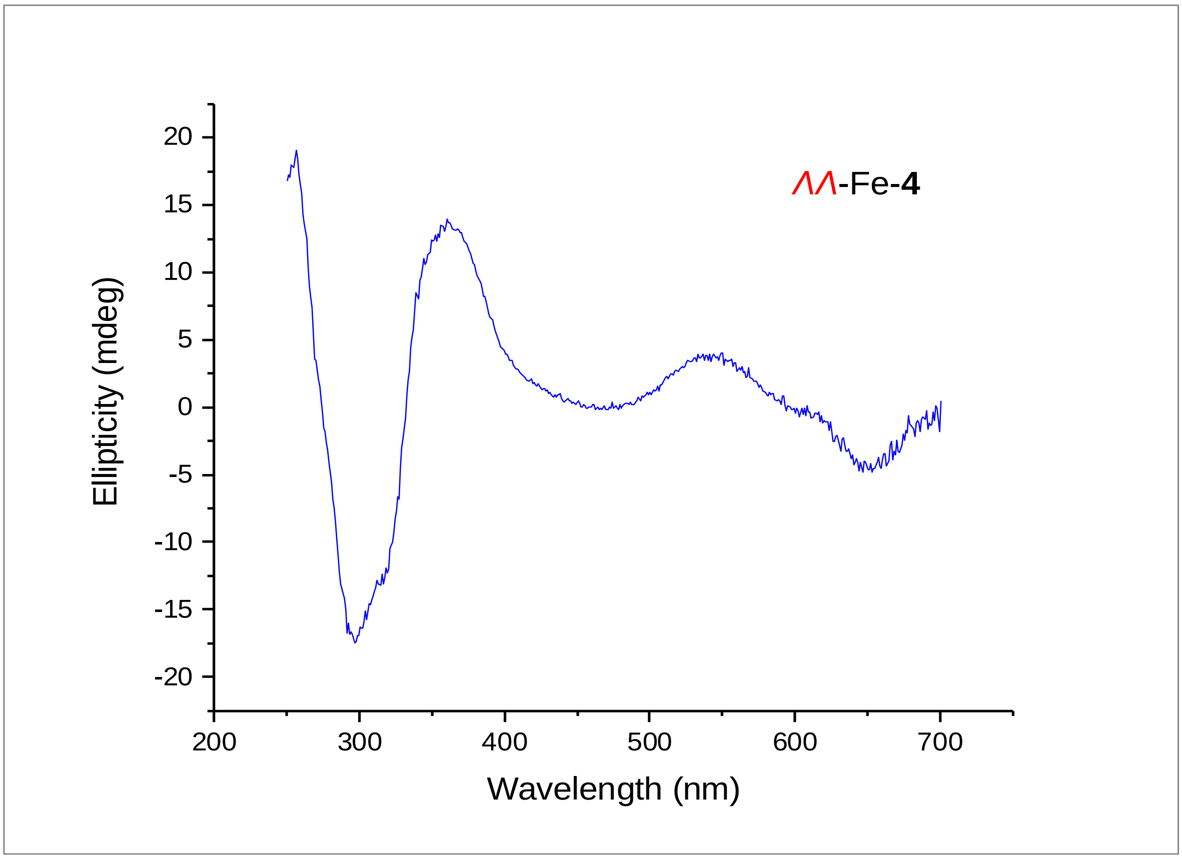

Figure S89. Circular dichroism (CD) spectrum of *Λ*Λ-Fe-**4**.

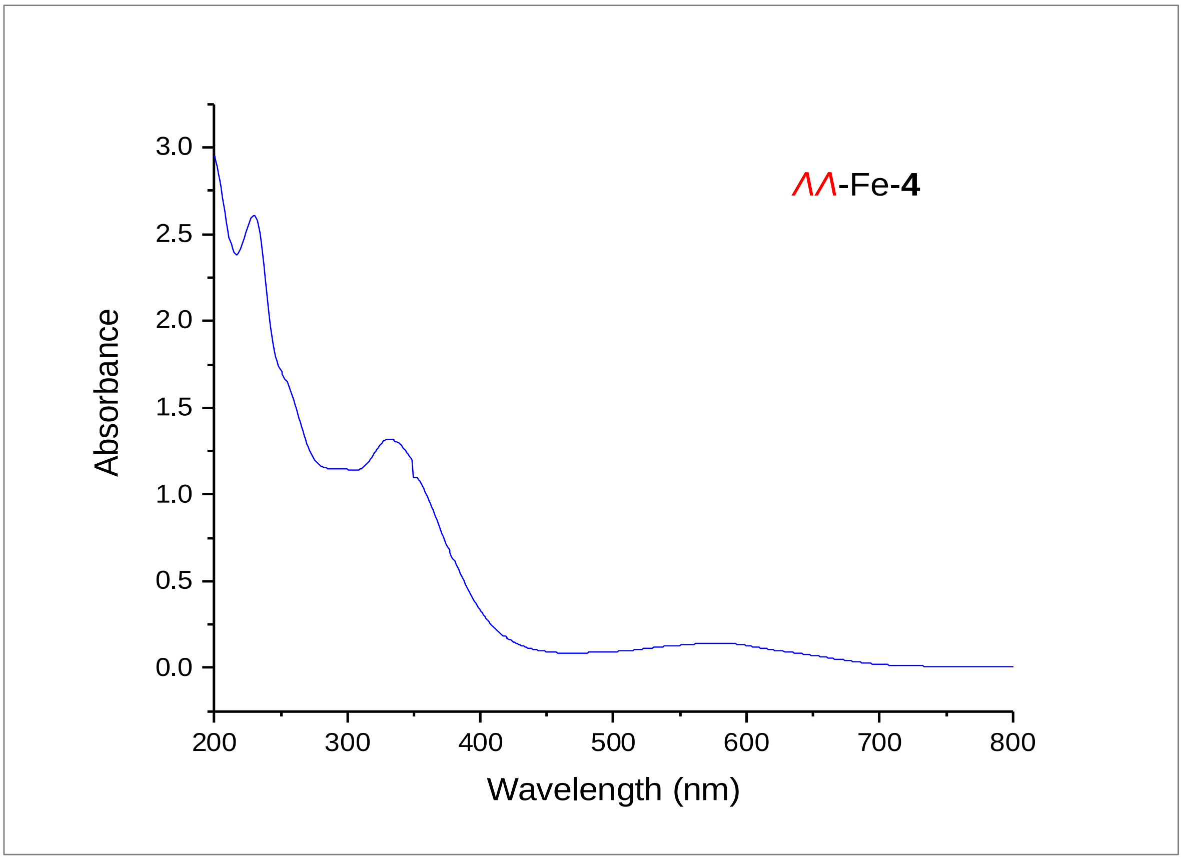

Figure S90. UV-Vis spectrum of *Λ*Λ-Fe-**4**.

4.2.2 using subcomponents B and C with Zn(II) salt

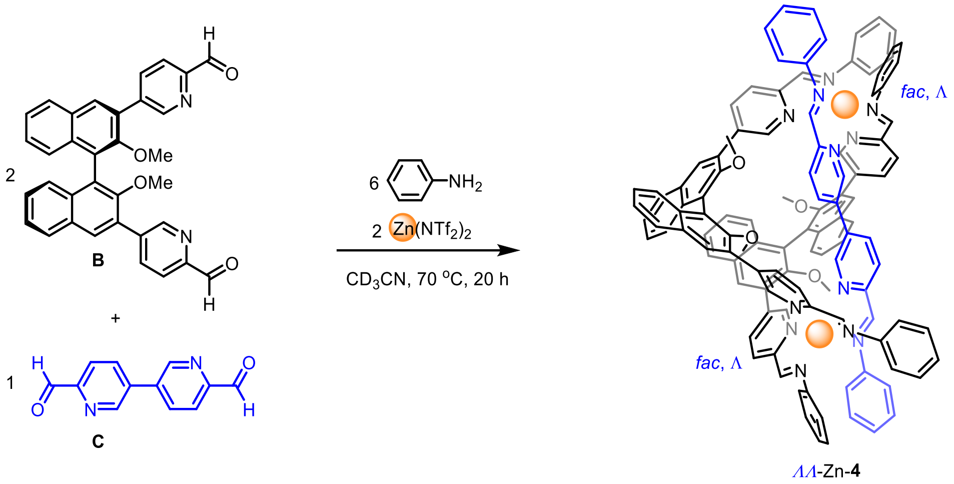

Subcomponents **B** (1.3 mg, 2.5 μmol, 1.0 equiv) and **C** (0.3 mg, 1.25 μmol, 0.5 equiv) were added to CD_3CN (0.6 mL) together with $Zn(NTf_2)_2$ (1.6 mg, 2.5 μmol, 1.0 equiv) and aniline (0.7 mg, 7.5 μmol, 3.0 equiv). The reaction mixture was stirred at 70 °C for 20h. Then the solvent was evaporated and diethyl ether was then added. The residue resuspended and then centrifuged and the diethyl ether decanted. This was repeated three times with fresh diethyl ether. The residue was then dried in vacuo to afford the desired product (*ΛΛ*-Zn-**4**) as a pale yellow solid (2.3 mg, 62% yield).

Characterization of *ΛΛ*-Zn-4:

^{1}H NMR (500 MHz, CD_3CN, 298 K) δ (ppm) = 8.80 (d, *J* = 0.6 Hz, 2H), 8.66 (s, 4H), 8.61 (d, *J* = 0.8 Hz, 2H), 8.38 (s, 2H), 8.21 – 8.18 (m, 4H), 8.12 – 8.10 (m, 2H), 8.04 (d, *J* = 2.1 Hz, 2H), 6.81 – 6.79 (m, 4H), 6.29 – 6.25 (m, 4H), 6.13 – 6.10 (m, 4H), 2.73 (s, 6H), 2.13 (s, 6H). (Note: due to the overlap with some impurities, ranges from 6.9 – 7.7 ppm were not integrated).

^{13}C NMR (125 MHz, CD_3CN, 298 K) δ (ppm) = 164.9, 164.2, 161.9, 152.2, 150.5, 147.3, 147.1, 147.1, 146.4, 141.0, 139.1, 135.2, 134.7, 132.0, 131.7, 130.9, 130.1, 130.0, 129.7, 129.5, 129.3, 129.0, 128.8, 128.7, 128.5, 128.3, 128.2, 127.2, 126.7, 125.0, 124.8, 123.7, 122.6, 121.9, 121.5, 121.4, 121.2, 60.6, 60.2. (Note: due to the overlap with some impurities, not all ^{13}C NMR signals could be distinguished).

ESI-MS *m/z* 460.4 $[M]^{4+}$, 707.1 $[M+NTf_2]^{3+}$, 1200.6 $[M+2NTf_2]^{2+}$.

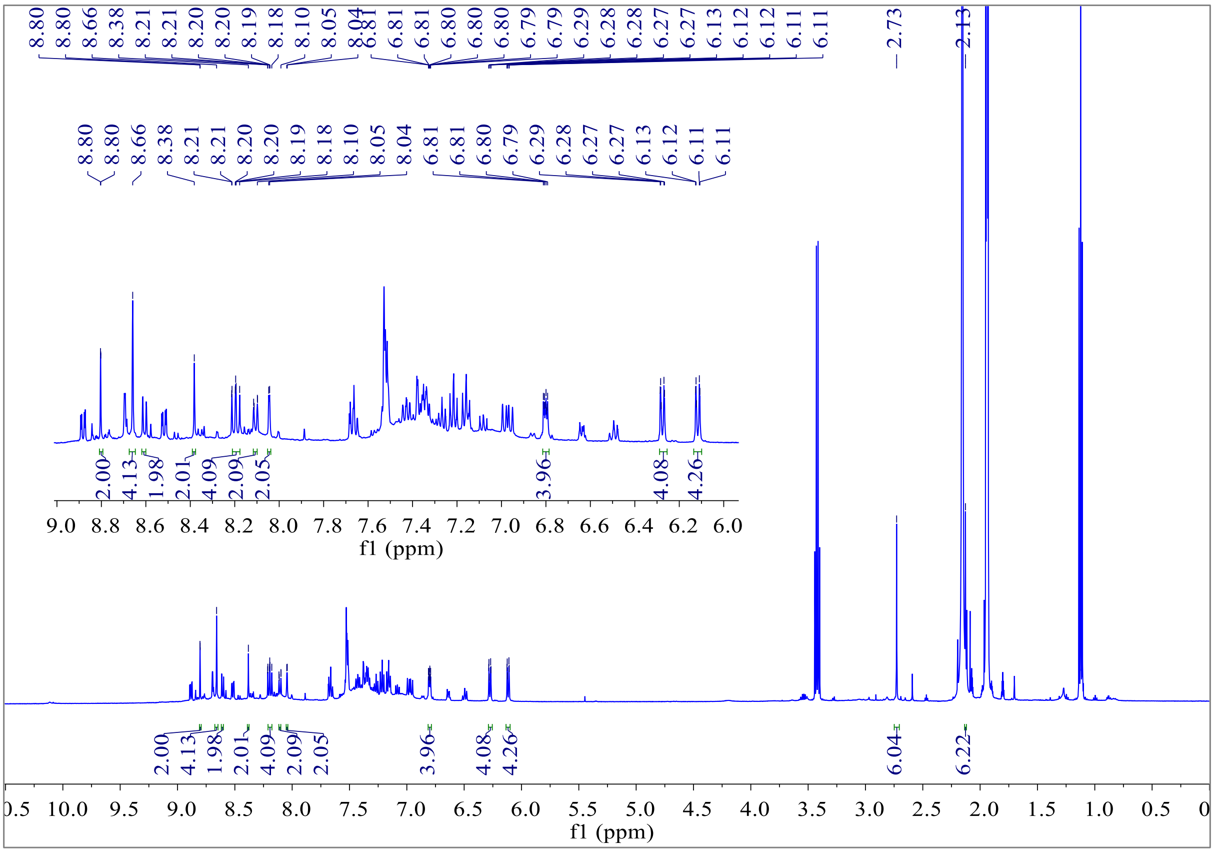

Figure S91. ^{1}H NMR spectrum of *ΛΛ*-Zn-**4** (500 MHz, CD_3CN, 298 K).

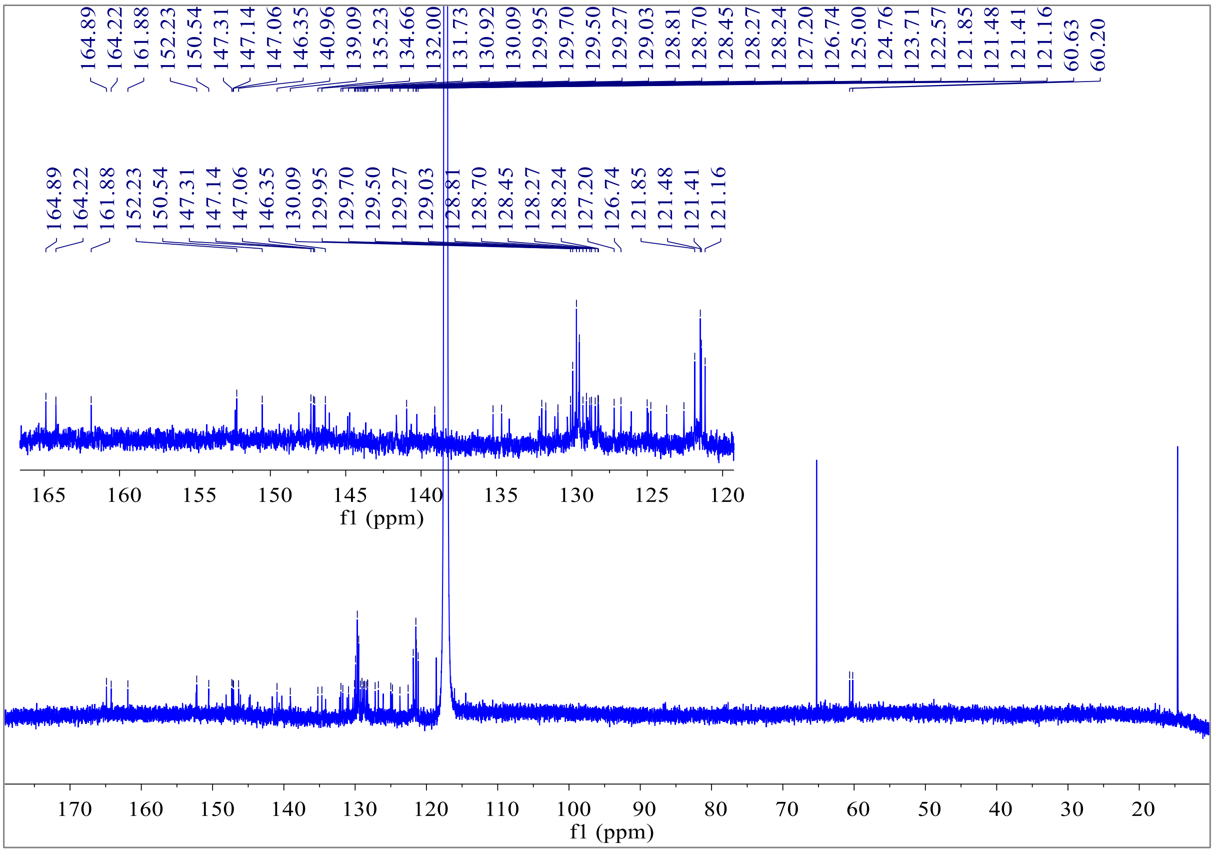

Figure S92. ^{13}C NMR spectrum of *ΛΛ*-Zn-**4** (125 MHz, CD_3CN, 298 K).

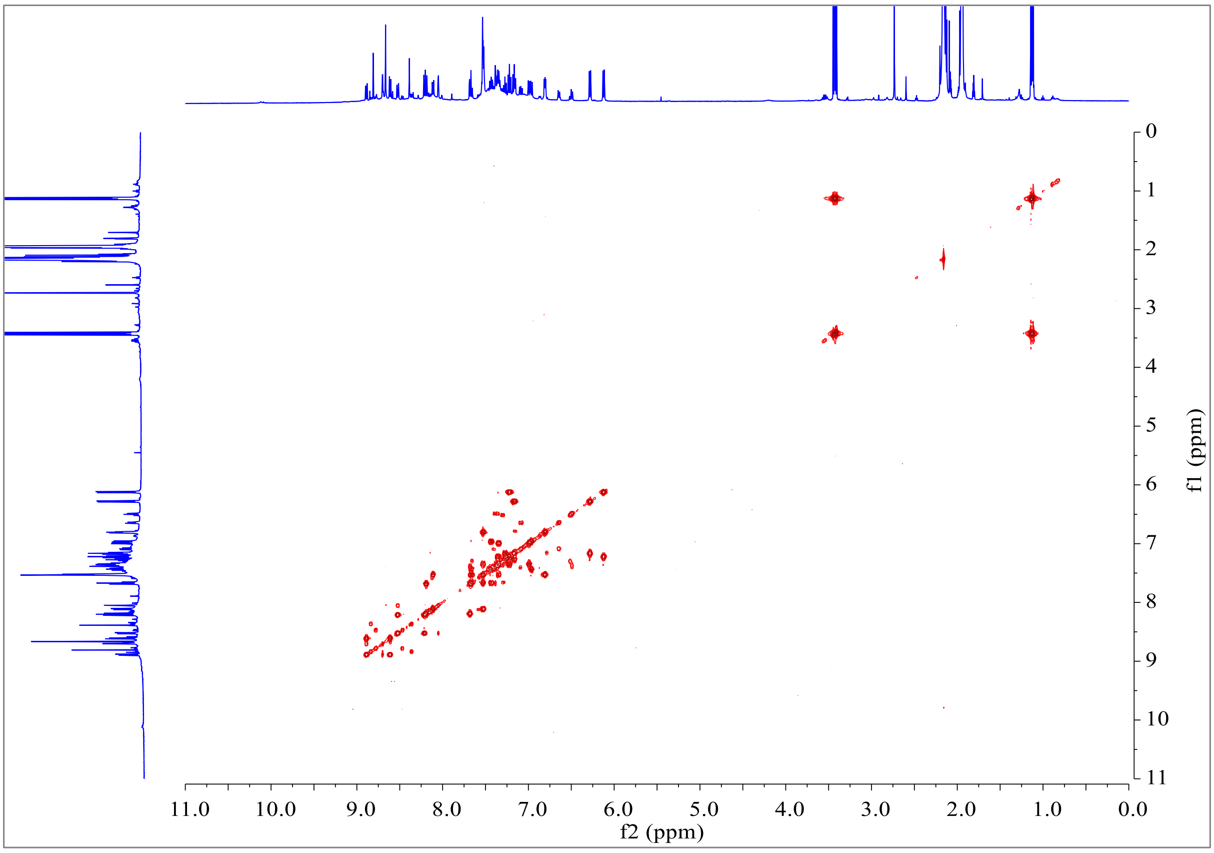

Figure S93. $^{1}H\{^{1}H\}$ COSY NMR spectrum of *ΛΛ*-Zn-**4** (500 MHz, CD_3CN, 298 K).

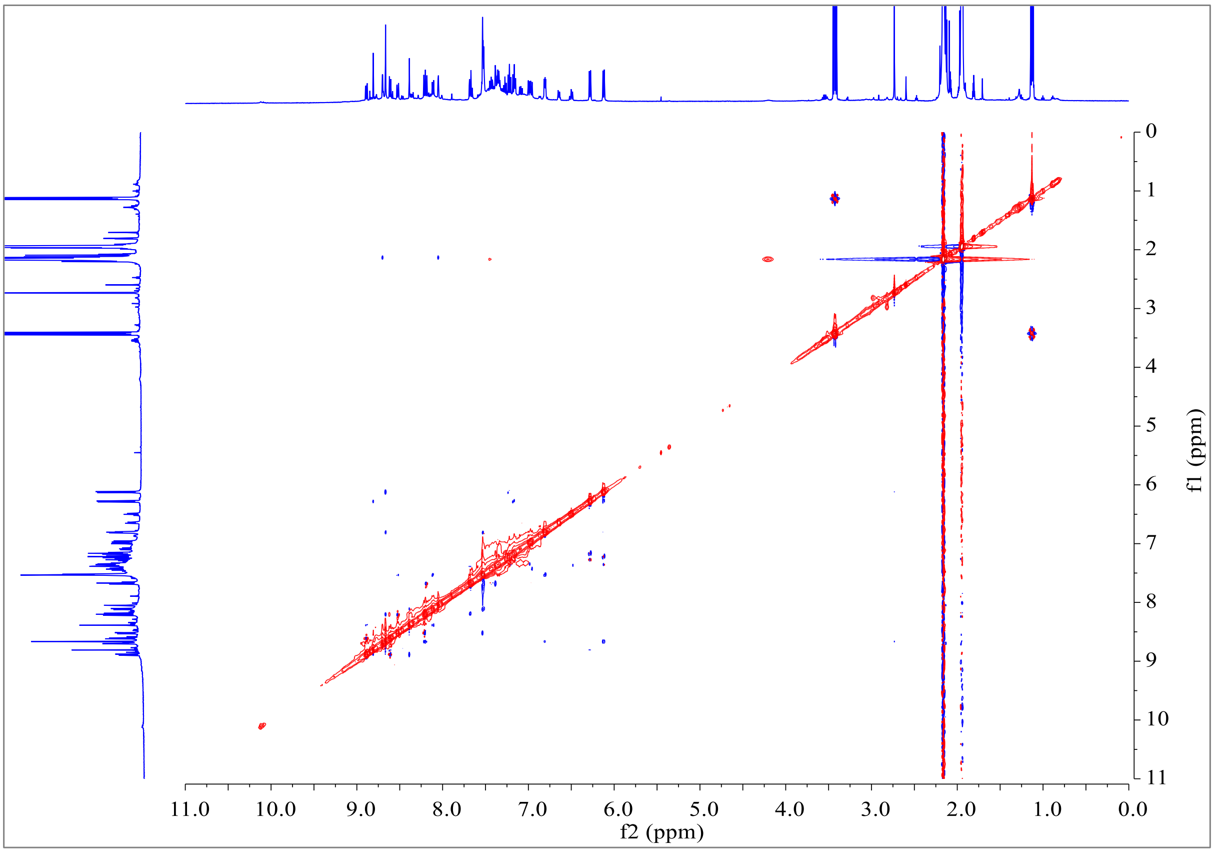

Figure S94. $^{1}H\{^{1}H\}$ NOESY NMR spectrum of *ΛΛ*-Zn-**4** at (500 MHz, CD_3CN, 298 K).

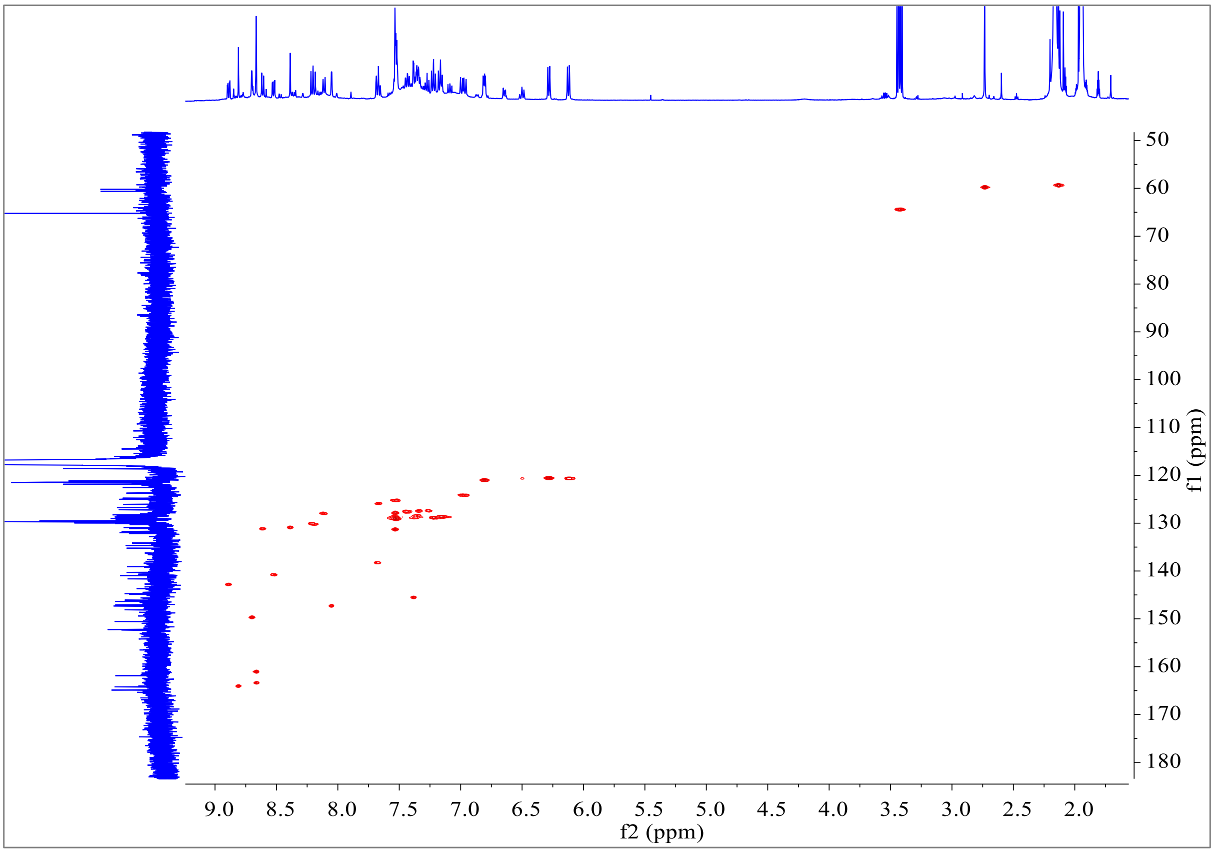

Figure S95. $^{1}H\{^{13}C\}$ HSQC NMR spectrum of *ΛΛ*-Zn-**4** (500 MHz, CD_3CN, 298 K).

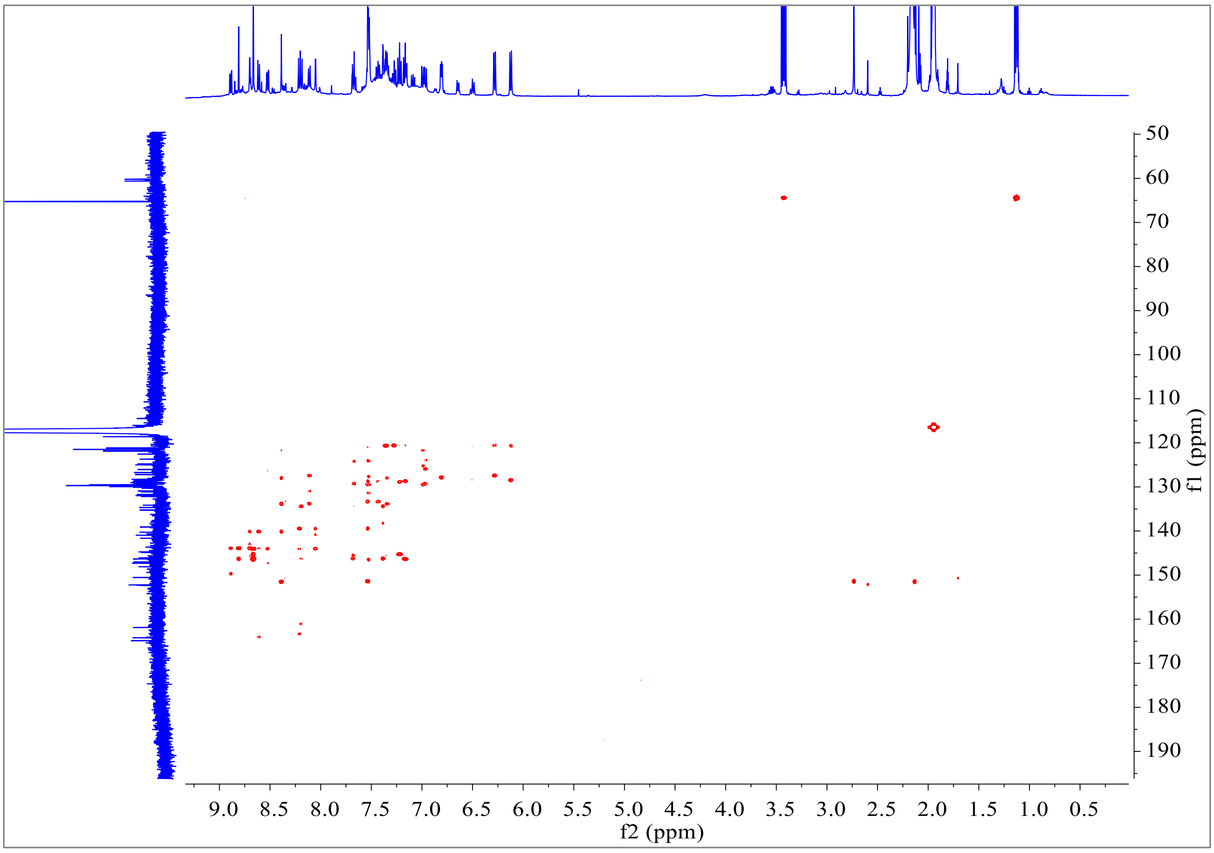

Figure S96. $^{1}H\{^{13}C\}$ HMBC NMR spectrum of *ΛΛ*-Zn-**4** (500 MHz, CD_3CN, 298 K).

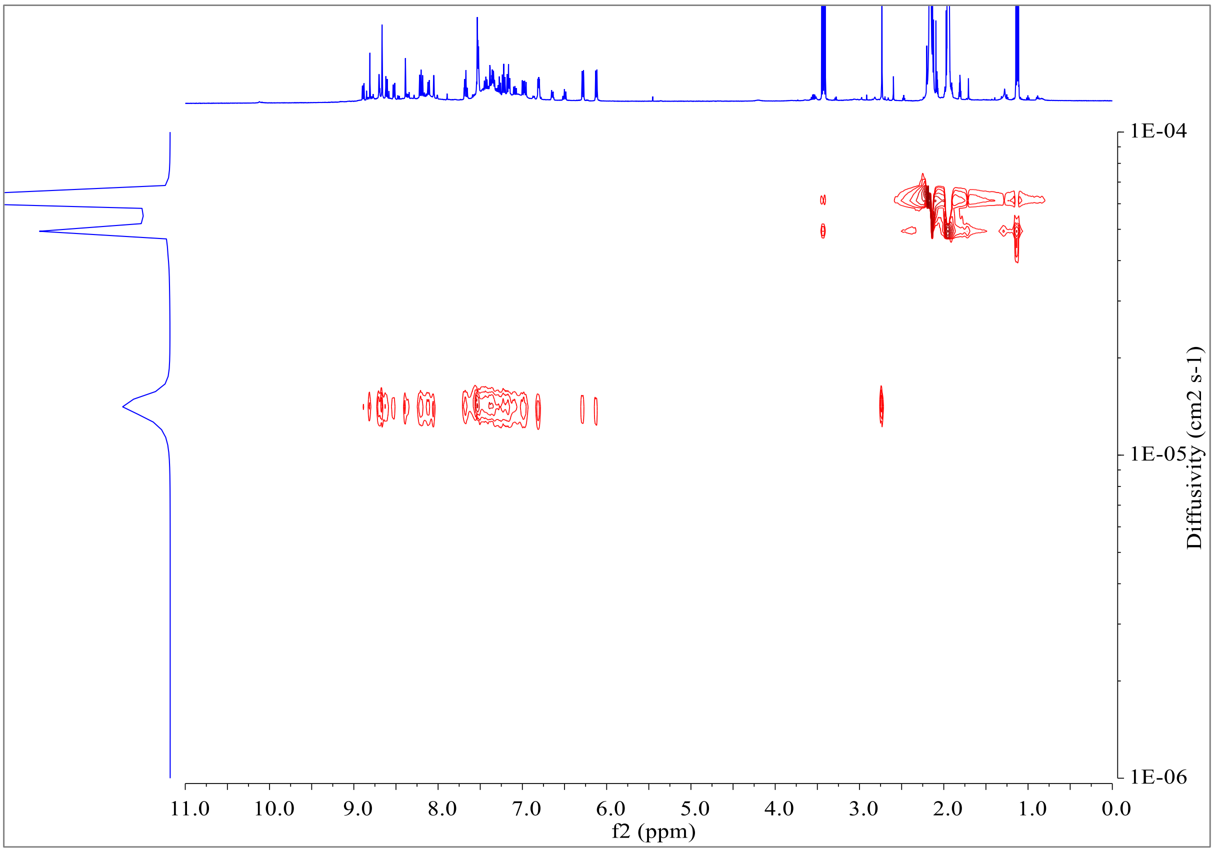

Figure S97. ^{1}H DOSY NMR spectrum of *ΛΛ*-Zn-**4** (400 MHz, CD_3CN, 298 K).

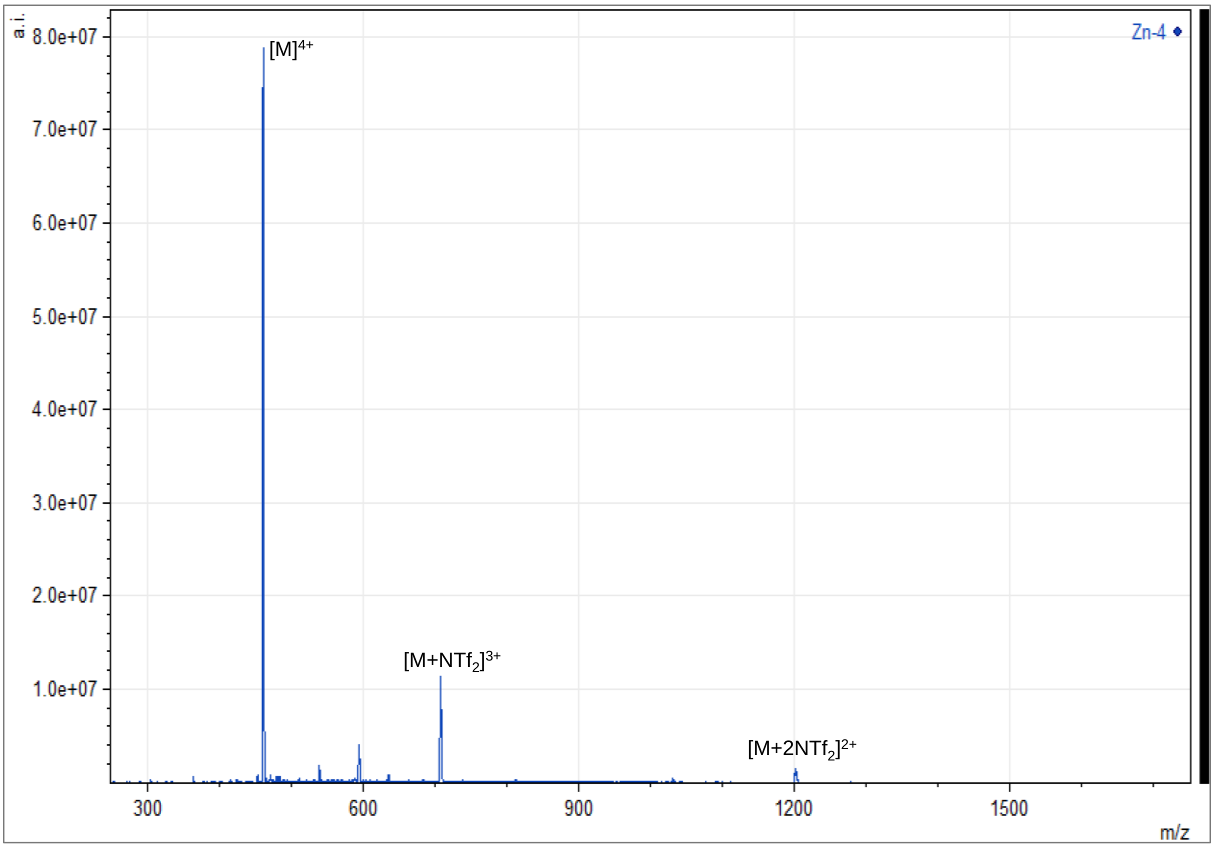

Figure S98. Low-resolution ESI-mass spectrum of *ΛΛ*-Zn-**4**.

4.2.3 using subcomponents B and D with Fe(II) salt

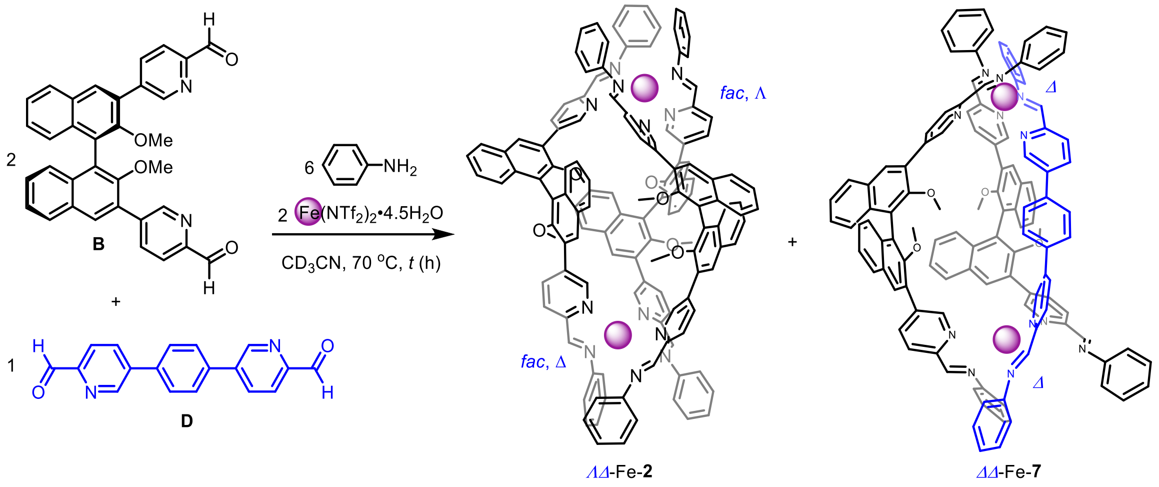

Subcomponents **B** (1.3 mg, 2.5 μmol, 1.0 equiv) and **D** (0.36 mg, 1.25 μmol, 0.5 equiv) were added to CD_3CN (0.6 mL) together with $Fe(NTf_2)_2{\cdot}4.5H_2O$ (1.7 mg, 2.5 μmol, 1.0 equiv) and aniline (0.7 mg, 7.5 μmol, 3.0 equiv). The reaction mixture was stirred at 70 °C and monitored by ^1H NMR. As shown in Figure S99, a new species (red, triangle) appearing at δ = 5.77 (d), 5.59 (d), 5.37 (d), 2.69 (s) and 1.82 (s) ppm was formed as the major product after 1 hour. The ^1H NMR spectrum was very close to that of *ΛΛ*-Fe-**4**, which indicated that Fe-**7**, assembled from two equivalents of **B** and one quivalent of **D** was formed. *Δ* Handedness of the two metal vertices in Fe-**7** was recorded from the CD and UV-vis spectrum (Figure S108 and S109). It is worth mentioning that only a small amount of *ΛΔ*-Fe-**2** was generated within 1 hour (blue, square). Another new species (black, star) appearing at δ = 5.51 (d) in the ^1H NMR spectrum was the homoleptic assembly from **D**. Interestingly, as the reaction proceeded, the homoleptic assemblies gradually converted to *ΛΛ*-Fe-**7**. After 96 hours *ΛΔ*-Fe-**2** was fully converted to *ΛΛ*-Fe-**7**. After cooling to room temperature, the solvent was evaporated and diethyl ether was then added. The residue was resuspended and then centrifuged and the diethyl ether decanted. This was repeated three times with fresh diethyl ether. The residue was then dried in vacuo to afford the desired product (*ΛΛ*-Fe-**7**) as a purple solid (2.9 mg, 76% yield).

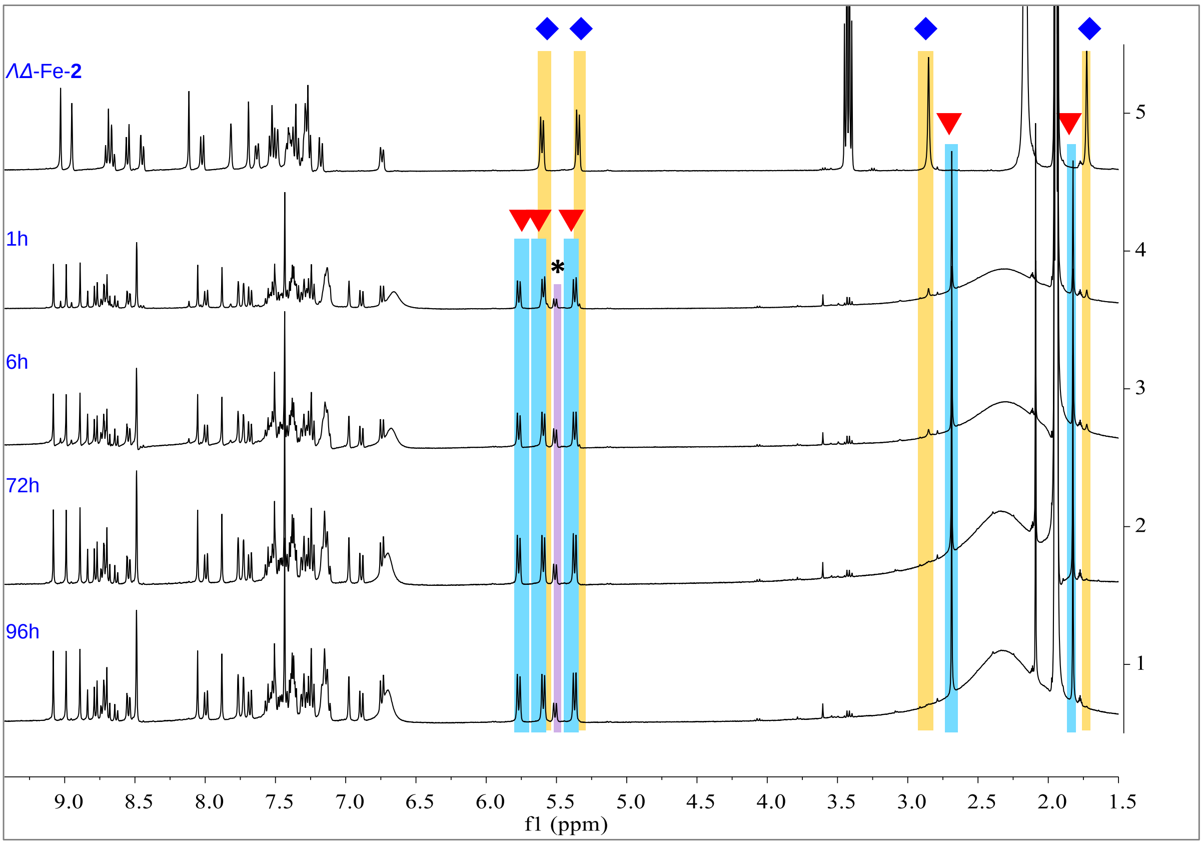

Figure S99. Crude ^{1}H NMR spectrum (400 MHz, CD_3CN, 298 K) (red triangle: *ΔΔ*-Fe-**7**; blue square: *ΛΔ*-Fe-**2**; black star: homoleptic assemblies from **D**).

Characterization of *ΔΔ*-Fe-7:

^{1}H NMR (500 MHz, CD_3CN, 298 K) δ (ppm) = 9.08 (s, 2H), 8.98 (s, 2H), 8.89 (s, 2H), 8.77 (d, *J* = 7.9 Hz, 2H), 8.71 (d, *J* = 1.8 Hz, 1H), 8.68 (d, *J* = 8.2 Hz, 2H), 8.63 (d, *J* = 8.2 Hz, 1H), 8.54 (m, 2H), 8.48 (d, *J* = 1.2 Hz, 2H), 8.05 (s, 2H), 7.99 (m, 2H), 7.88 (s, 2H), 7.76 (d, *J* = 2.0 Hz, 2H), 7.72 (d, *J* = 1.8 Hz, 2H), 7.69 – 7.66 (m, 2H), 7.56 – 7.11 (m, 32H), 6.97 (s, 2H), 6.88 (m, 2H), 6.74 (m, 2H), 5.80 – 5.74 (m, 4H), 5.62 – 5.56 (m, 4H), 5.39 – 5.34 (m, 4H), 2.68 (s, 6H), 1.82 (s, 6H). (Note: due to overlap with the homoleptic assemblies from **D**, the range from δ 7.11 to 7.56 ppm was not integrated).

^{13}C NMR (125 MHz, CD_3CN, 298 K) δ (ppm) = 176.0, 175.3, 174.9, 158.8, 158.8, 158.2, 156.4, 154.5, 153.9, 153.0, 151.6, 151.1, 150.8, 142.5, 142.2, 142.2, 141.5, 140.8, 138.0, 137.1, 135.3, 135.2, 132.3, 131.9, 131.8, 131.3, 131.2, 130.6, 130.5, 130.4, 129.9, 129.9, 129.8, 129.2, 129.0, 128.9, 128.7, 127.3, 127.1, 126.2, 126.2, 125.8, 122.8, 122.5, 122.3, 122.2, 122.2, 61.7, 60.4. (Note: due to the overlap with the homoleptic assemblies from subcomponent **D**, not all ^{13}C NMR signals could be distinguished).

ESI-MS *m/z* 474.8 $[M]^{4+}$, 726.3 $[M+NTf_2]^{3+}$, 1229.4 $[M+2NTf_2]^{2+}$.

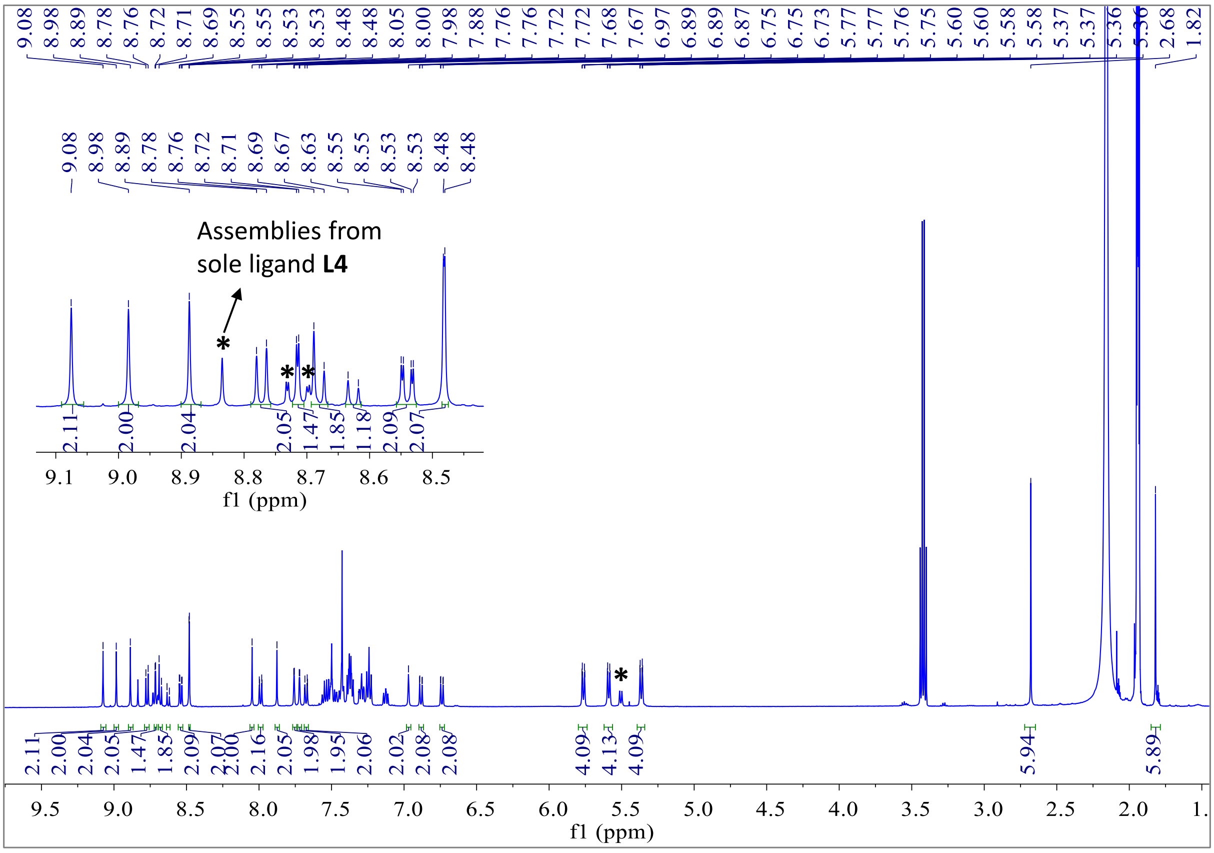

Figure S100. ^{1}H NMR spectrum of *ΔΔ*-Fe-**7** (500 MHz, CD_3CN, 298 K).

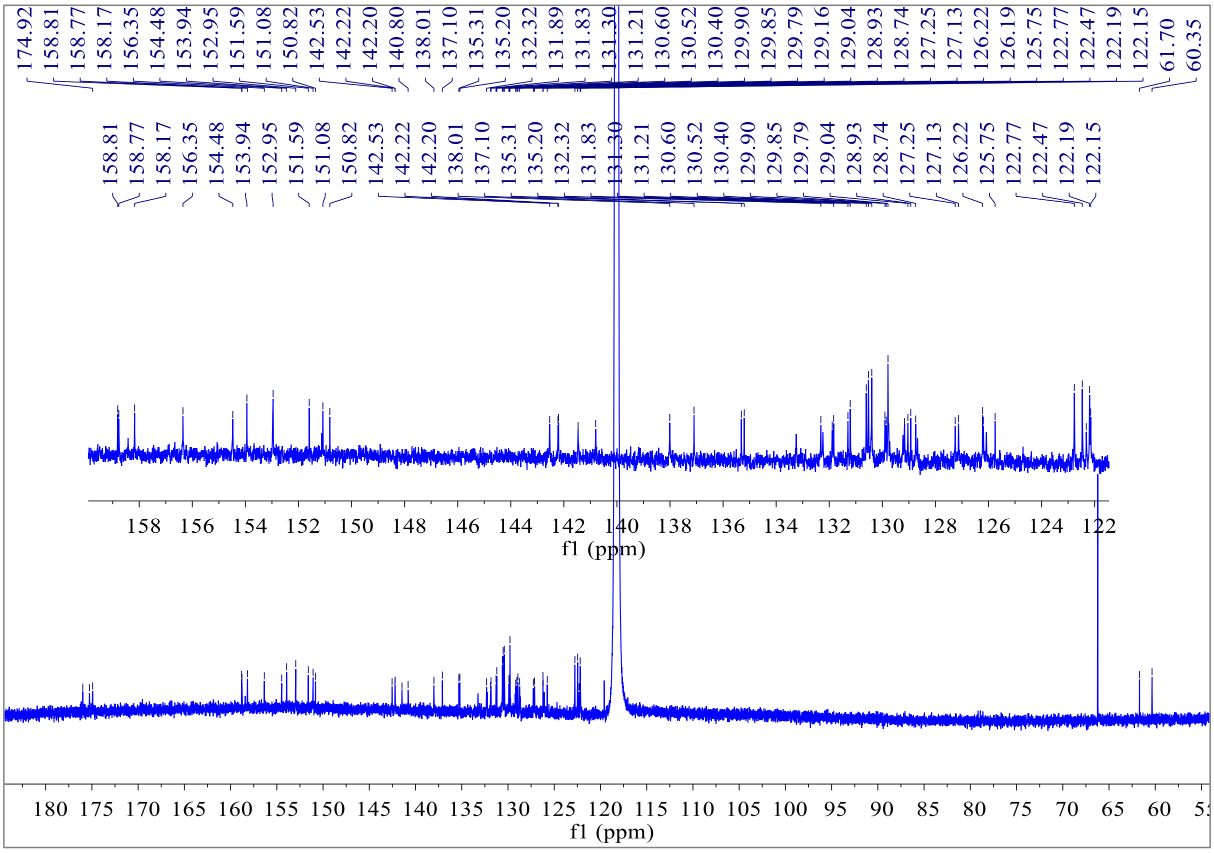

Figure S101. ^{13}C NMR spectrum of *ΔΔ*-Fe-**7** (125 MHz, CD_3CN, 298 K).

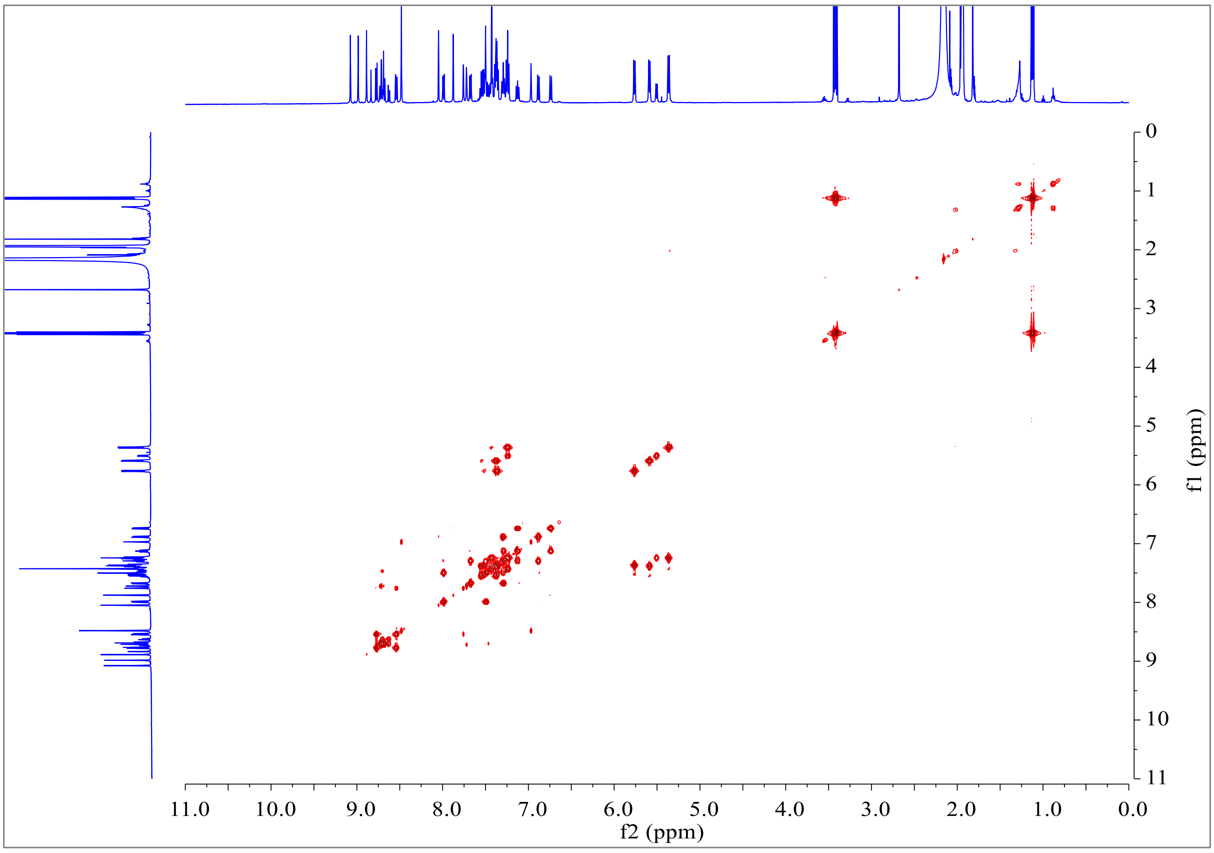

Figure S102. $^{1}H\{^{1}H\}$ COSY NMR spectrum of *ΔΔ*-Fe-**7** (500 MHz, CD_3CN, 298 K).

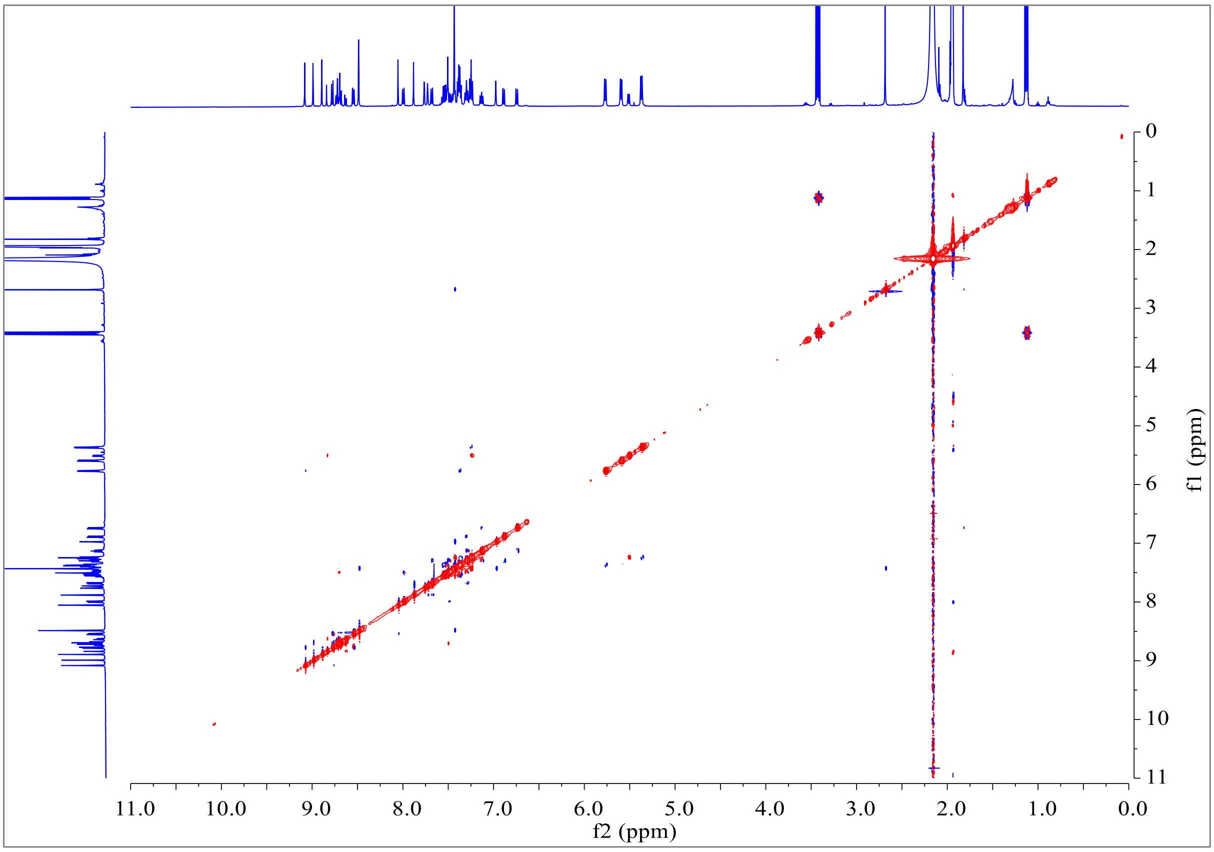

Figure S103. $^{1}H\{^{1}H\}$ NOESY NMR spectrum of *ΔΔ*-Fe-**7** at (500 MHz, CD_3CN, 298 K).

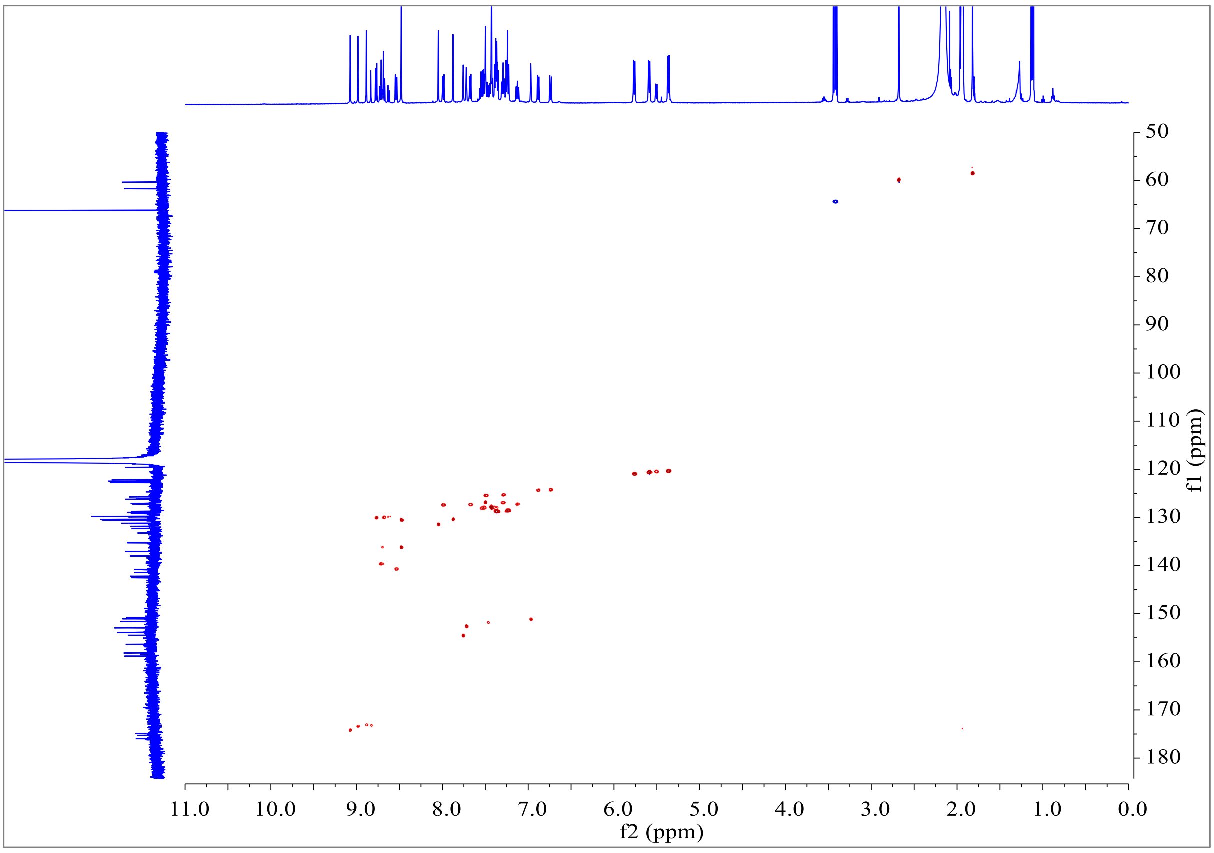

Figure S104. $^1H\{^{13}C\}$ HSQC NMR spectrum of *ΔΔ*-Fe-**7** (500 MHz, CD_3CN, 298 K).

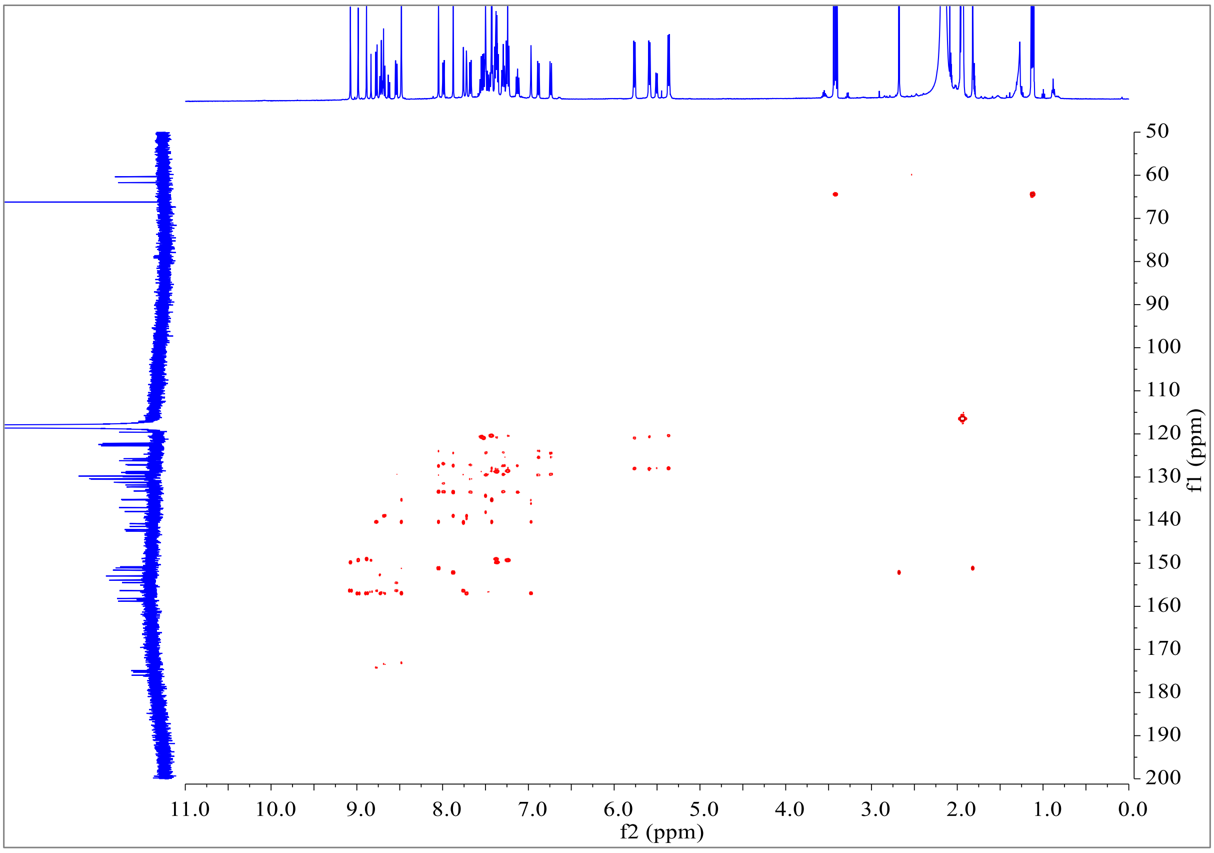

Figure S105. $^1H\{^{13}C\}$ HMBC NMR spectrum of *ΔΔ*-Fe-**7** (500 MHz, CD_3CN, 298 K).

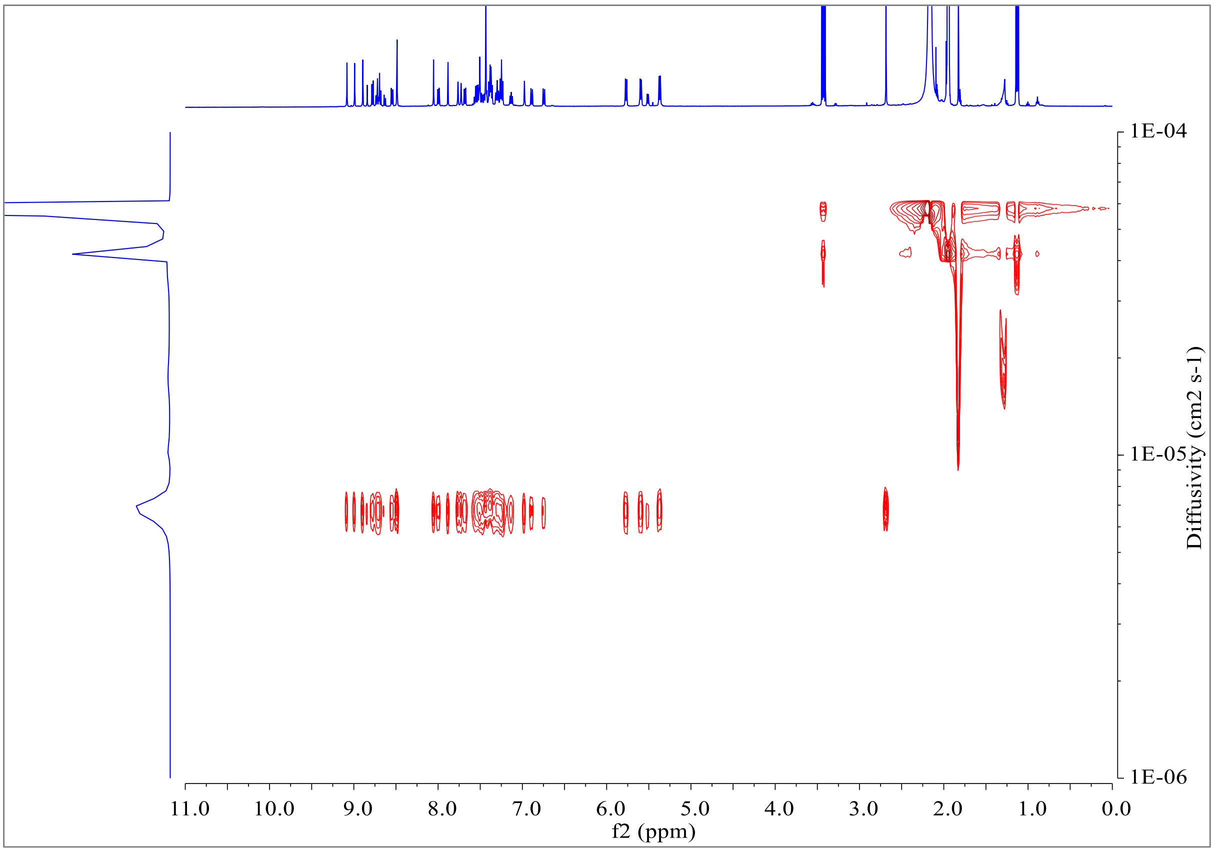

Figure S106. ^{1}H DOSY NMR spectrum of *ΔΔ*-Fe-**7** (400 MHz, CD_3CN, 298 K).

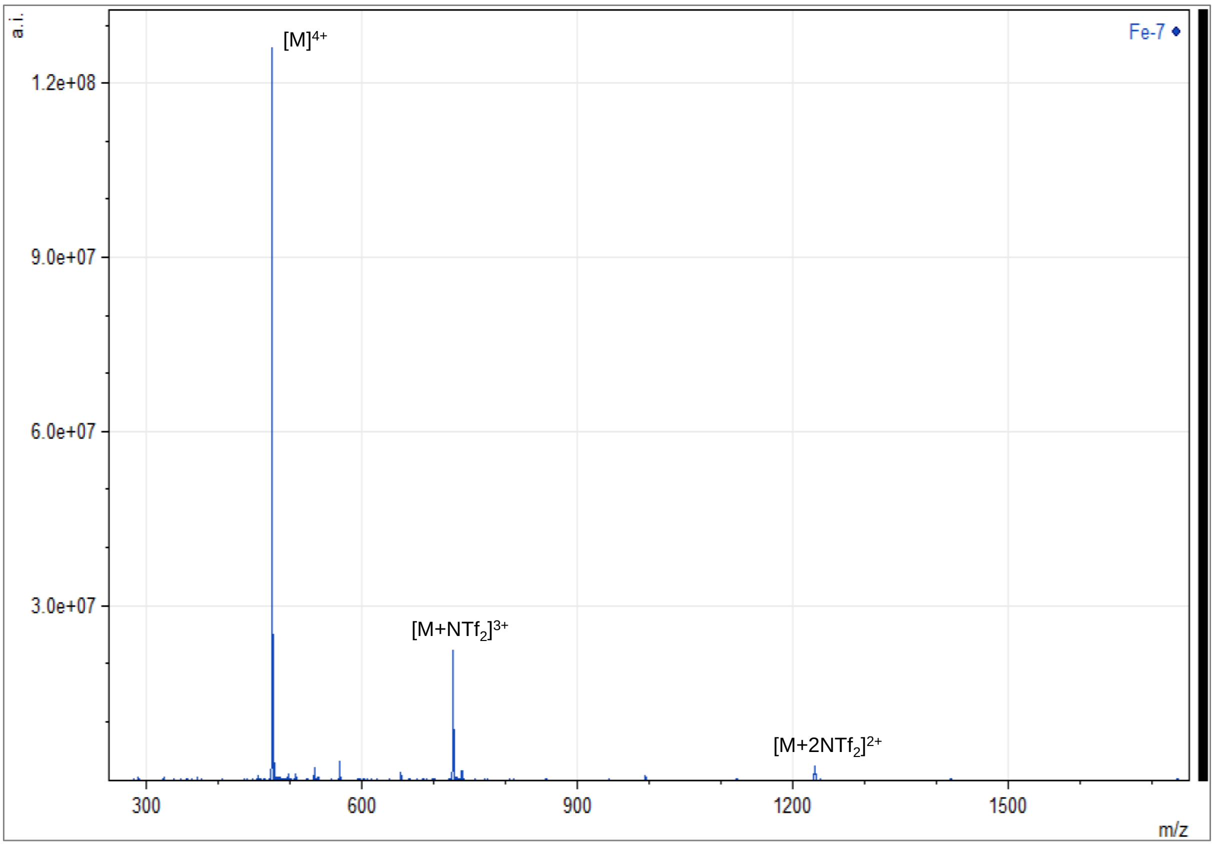

Figure S107. Low-resolution ESI-mass spectrum of *ΔΔ*-Fe-**7**.

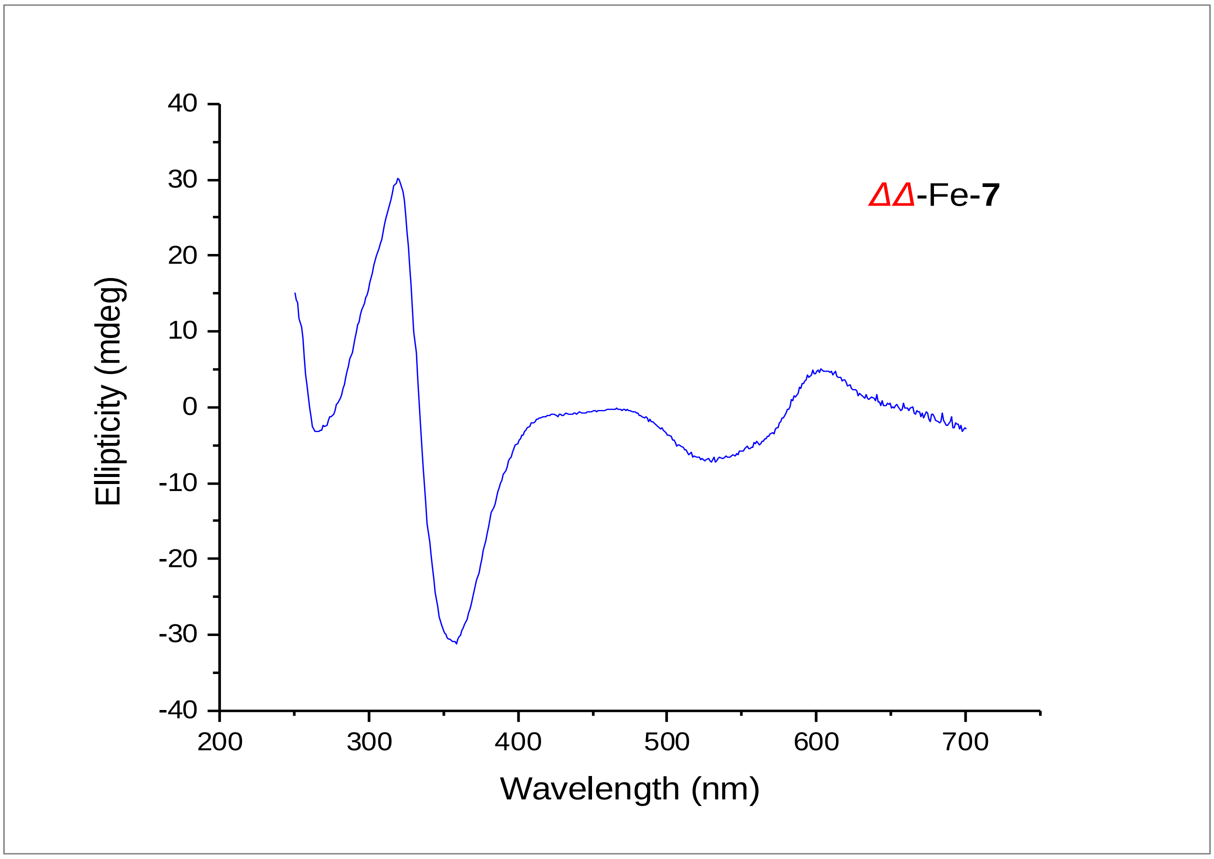

Figure S108. Circular dichroism (CD) spectrum of *ΔΔ*-Fe-**7**.

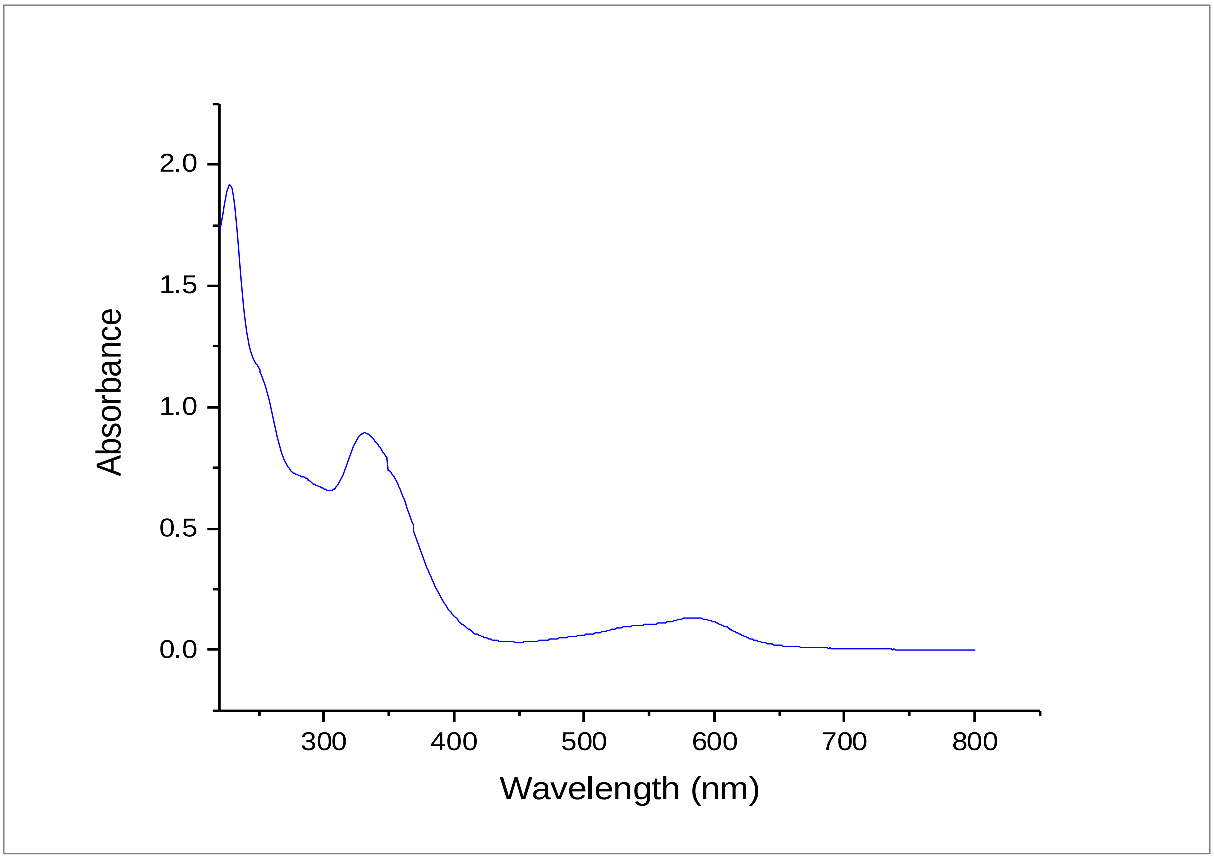

Figure S109. UV-Vis spectrum of *ΔΔ*-Fe-**7**.

4.2.4 using subcomponents B and D with Zn(II) salt

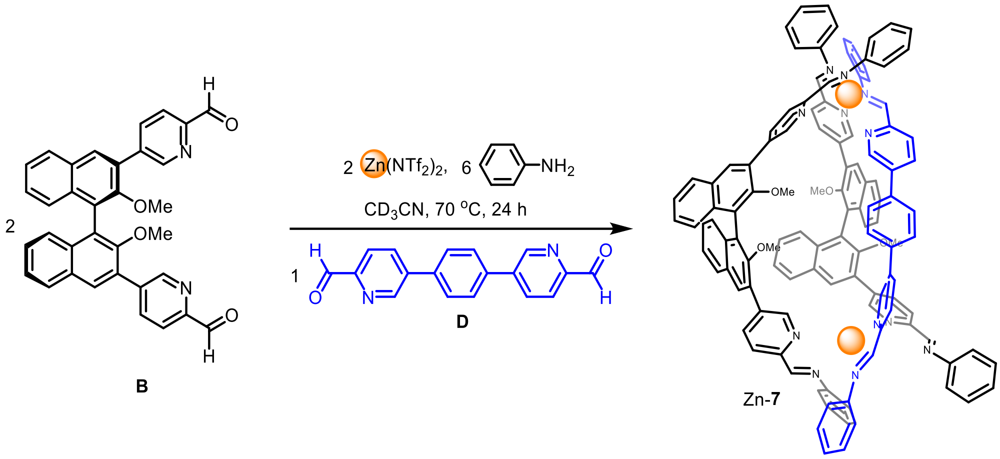

Subcomponents **B** (1.3 mg, 2.5 μmol, 1.0 equiv) and **D** (0.36 mg, 1.25 μmol, 0.5 equiv) were added to CD_3CN (0.6 mL) together with $Zn(NTf_2)_2$ (1.6 mg, 2.5 μmol, 1.0 equiv) and aniline (0.7 mg, 7.5 μmol, 3.0 equiv). The reaction mixture was stirred at 70 °C for 24 hours. As shown in Figure S110, a new species appearing at δ = 5.77 (d), 5.54 (d), 5.35 (d) and 6.27 (d) ppm was formed. The ^{1}H NMR spectrum was very close to that of *ΛΛ*-Fe-**7**, which indicated that Zn-**7**, assembled from two equivalents of **B** and one quivalent of **D** with $Zn(NTf_2)_2$ was formed. After cooling to room temperature, the solvent was evaporated and diethyl ether was then added. The residue was resuspended and then centrifuged and the diethyl ether decanted. This was repeated three times with fresh diethyl ether. The residue was then dried in vacuo to afford the desired product Zn-**7** as a pale yellow solid (3.1 mg, 82% yield).

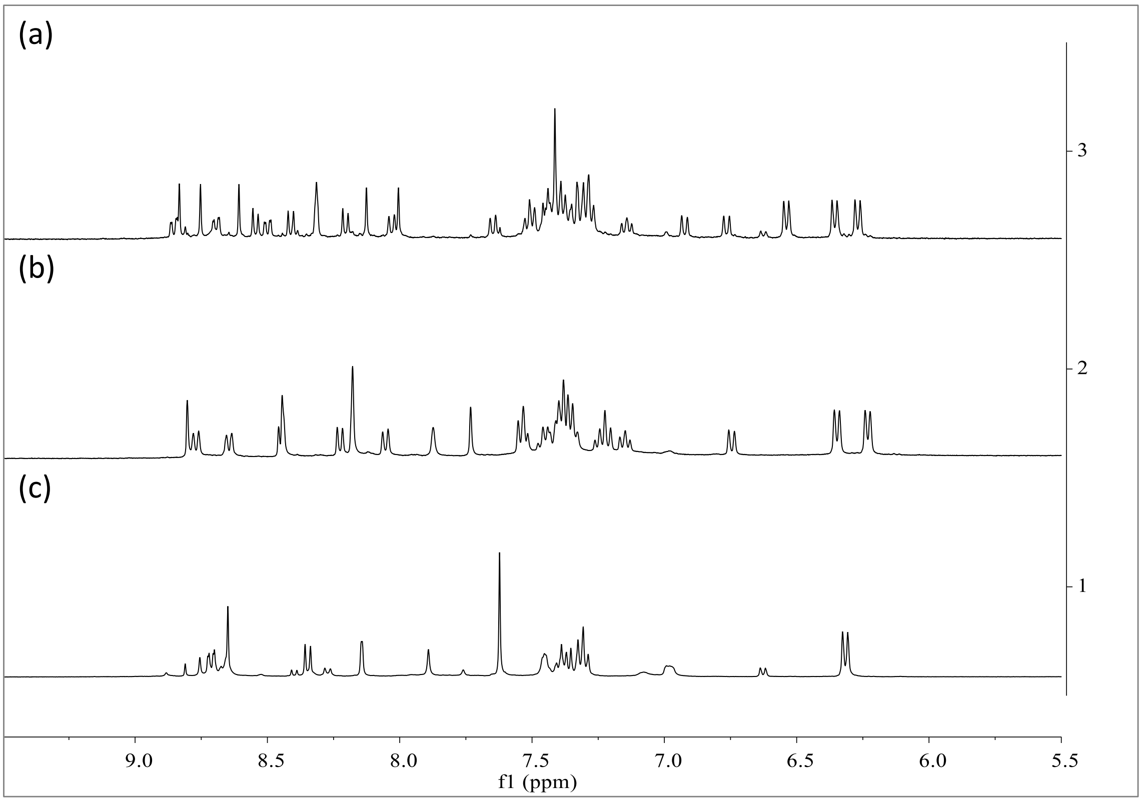

Figure S110. (a) ^{1}H NMR of self-assembly of a mixture of **B** and **D** (2:1) with aniline and $Zn(NTf_2)_2$. (b) ^{1}H NMR of cage Zn-**2**. (c) ^{1}H NMR of self-assembly of **D** with aniline and $Zn(NTf_2)_2$. (400M NMR, CD_3CN, 298K).

Characterization of ***ΔΔ*****-Zn-7:**

^{1}H NMR (500 MHz, CD_3CN, 298 K) δ (ppm) = 8.85 (m, 2H), 8.83 (s, 2H), 8.75 (s, 2H), 8.69 (m, 2H), 8.61 (s, 2H), 8.54 (d, *J* = 7.9 Hz, 2H), 8.50 (m, 2H), 8.41 (d, *J* = 8.1 Hz, 2H), 8.31 (s, 4H), 8.20 (d, *J* = 7.9 Hz, 2H), 8.12 (s, 2H), 8.03 (d, *J* = 8.2 Hz, 2H), 8.00 (s, 2H), 7.64 (d, *J* = 8.2 Hz, 2H), 7.51 (t, *J* = 7.3 Hz, 4H), 7.44 – 7.29 (m, 26H), 7.16 – 7.12 (m, 2H), 6.92 (d, *J* = 8.6 Hz, 2H), 6.76 (d, *J* = 8.6 Hz, 2H), 6.54 (d, *J* = 7.7 Hz, 4H), 6.35 (d, *J* = 7.5 Hz, 4H), 6.27 (d, *J* = 7.9 Hz, 4H), 2.84 (s, 6H), 2.10 (s, 6H).

^{13}C NMR (125 MHz, CD_3CN, 298 K) δ (ppm) = 165.70, 164.92, 164.80, 153.95, 153.00, 150.16, 148.18, 148.11, 148.09, 148.05, 147.10, 146.76, 146.68, 146.65, 144.64, 144.14, 142.89, 142.73, 141.24, 140.87, 137.36, 135.32, 135.23, 133.40, 132.71, 132.09, 131.81, 131.50, 131.19, 130.96, 130.73, 130.68, 130.63, 130.56, 129.56, 129.45, 129.30, 129.08, 128.99, 128.86, 128.82, 127.27, 127.17, 126.46, 126.14, 125.92, 122.75, 122.54, 122.39, 122.14, 119.59, 61.62, 60.50.

ESI-MS *m/z* 479.7 $[M]^{4+}$, 733.0 $[M+NTf_2]^{3+}$, 1239.4 $[M+2NTf_2]^{2+}$.

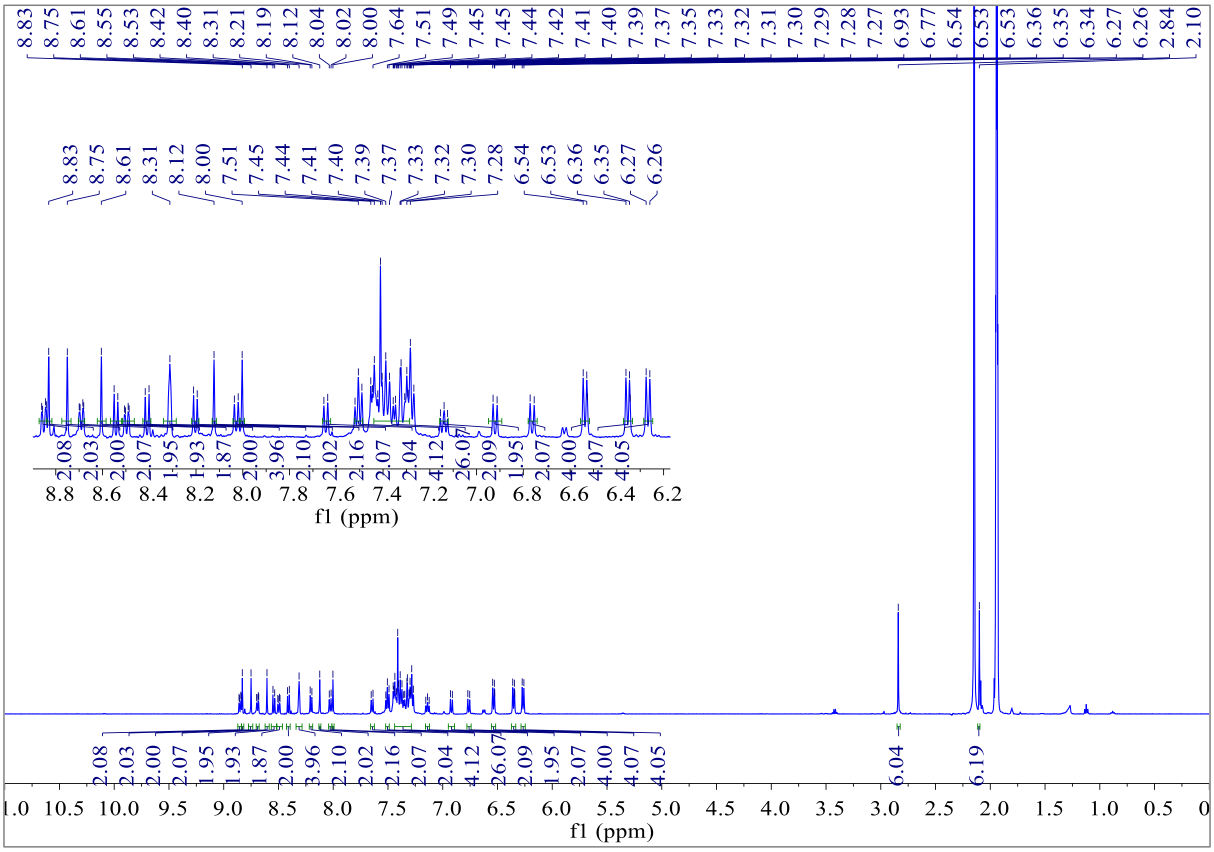

Figure S111. ^{1}H NMR spectrum of *ΔΔ*-Zn-**7** (500 MHz, CD_3CN, 298 K).

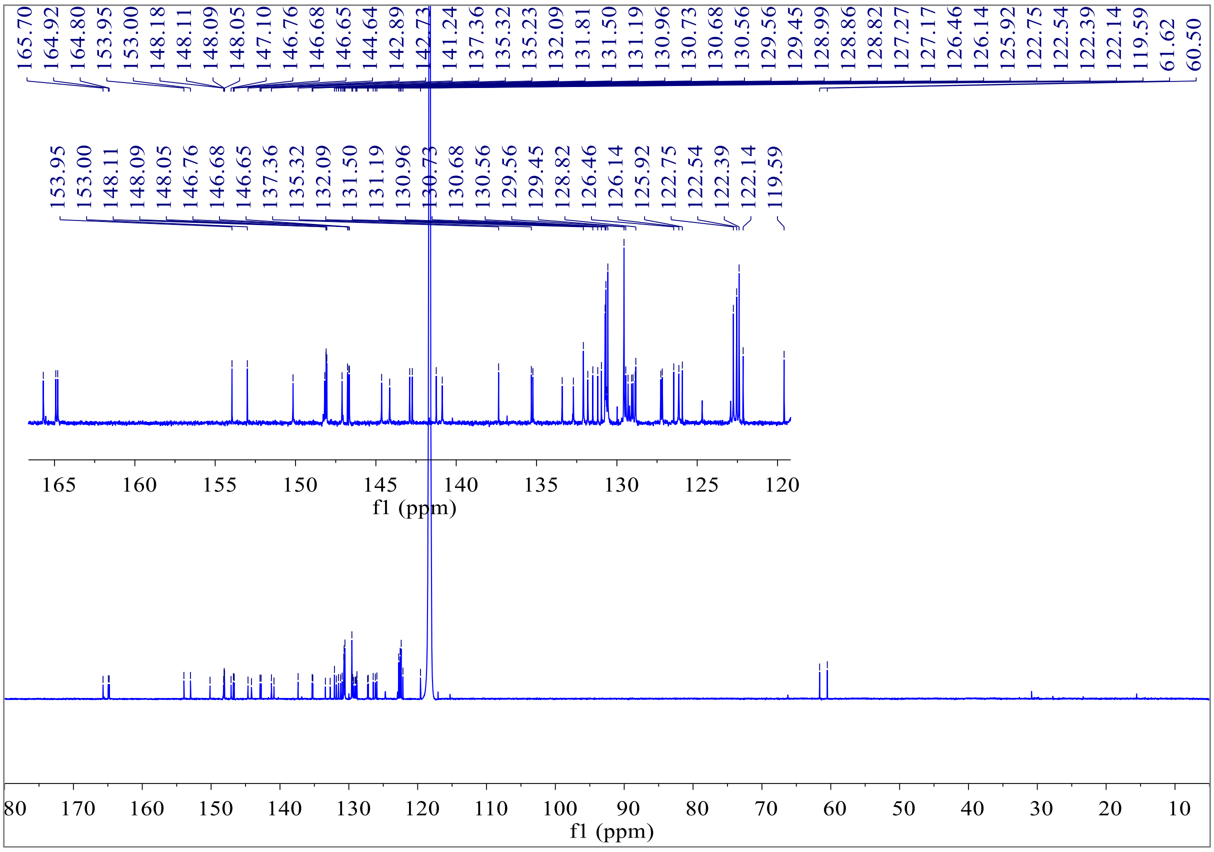

Figure S112. ^{13}C NMR spectrum of *ΔΔ*-Zn-**7** (125 MHz, CD_3CN, 298 K).

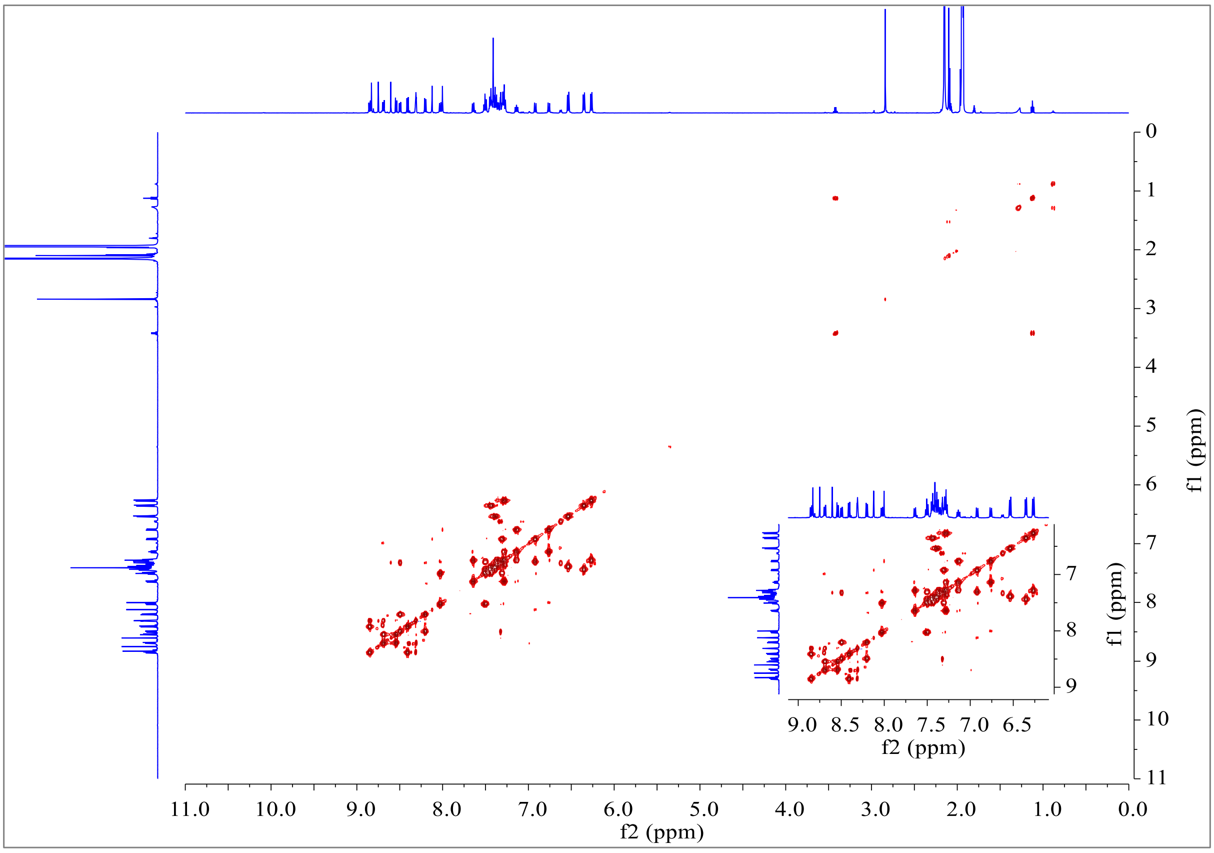

Figure S113. $^{1}H\{^{1}H\}$ COSY NMR spectrum of *ΔΔ*-Zn-**7** (500 MHz, CD_3CN, 298 K).

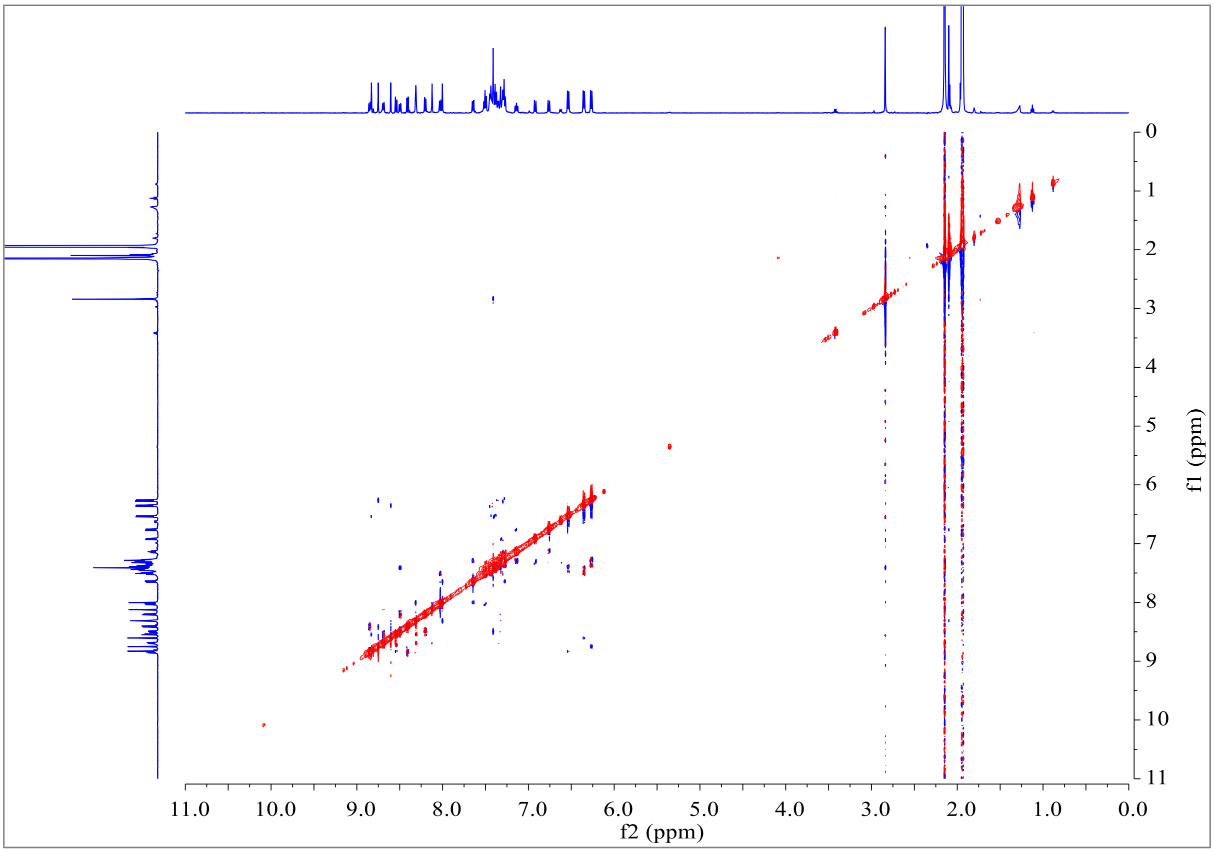

Figure S114. $^{1}H\{^{1}H\}$ NOESY NMR spectrum of *ΔΔ*-Zn-**7** at (500 MHz, CD_3CN, 298 K).

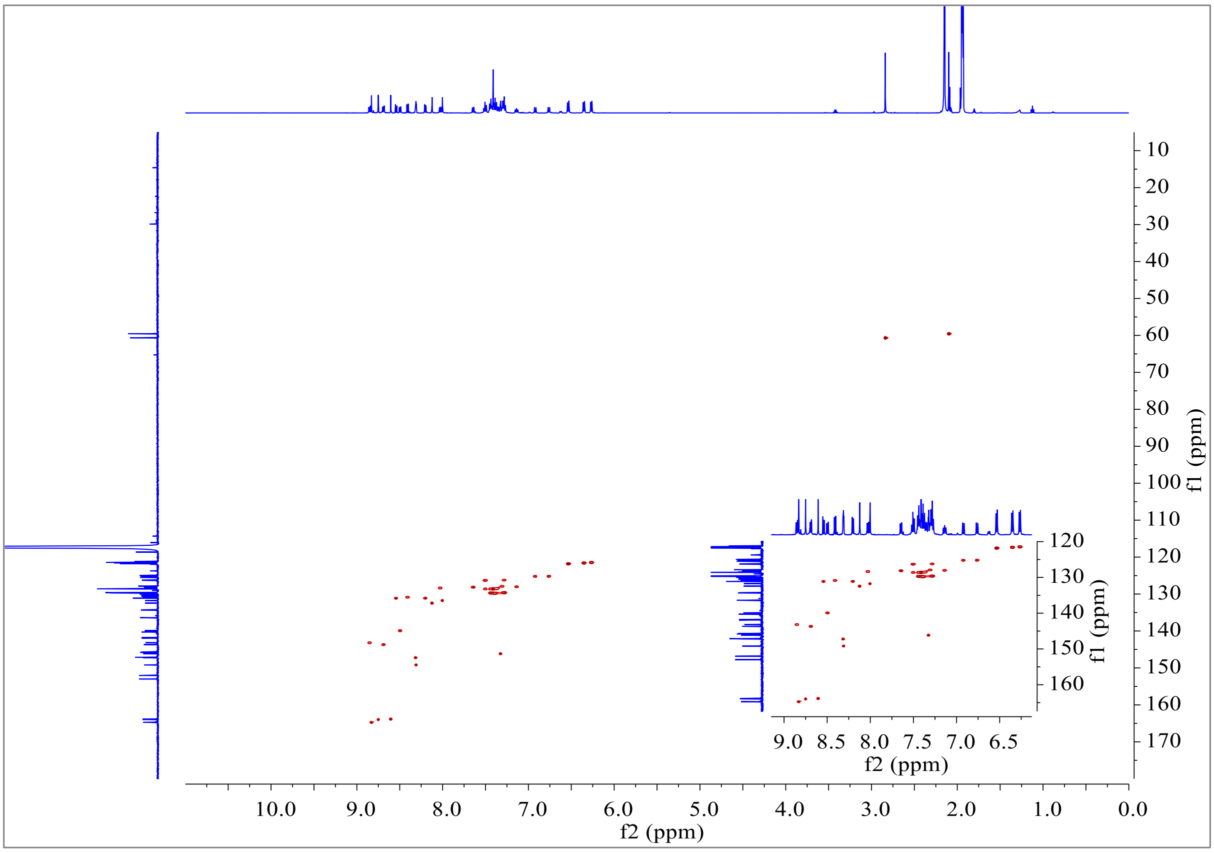

Figure S115. $^{1}H\{^{13}C\}$ HSQC NMR spectrum of *ΔΔ*-Zn-**7** (500 MHz, CD_3CN, 298 K).

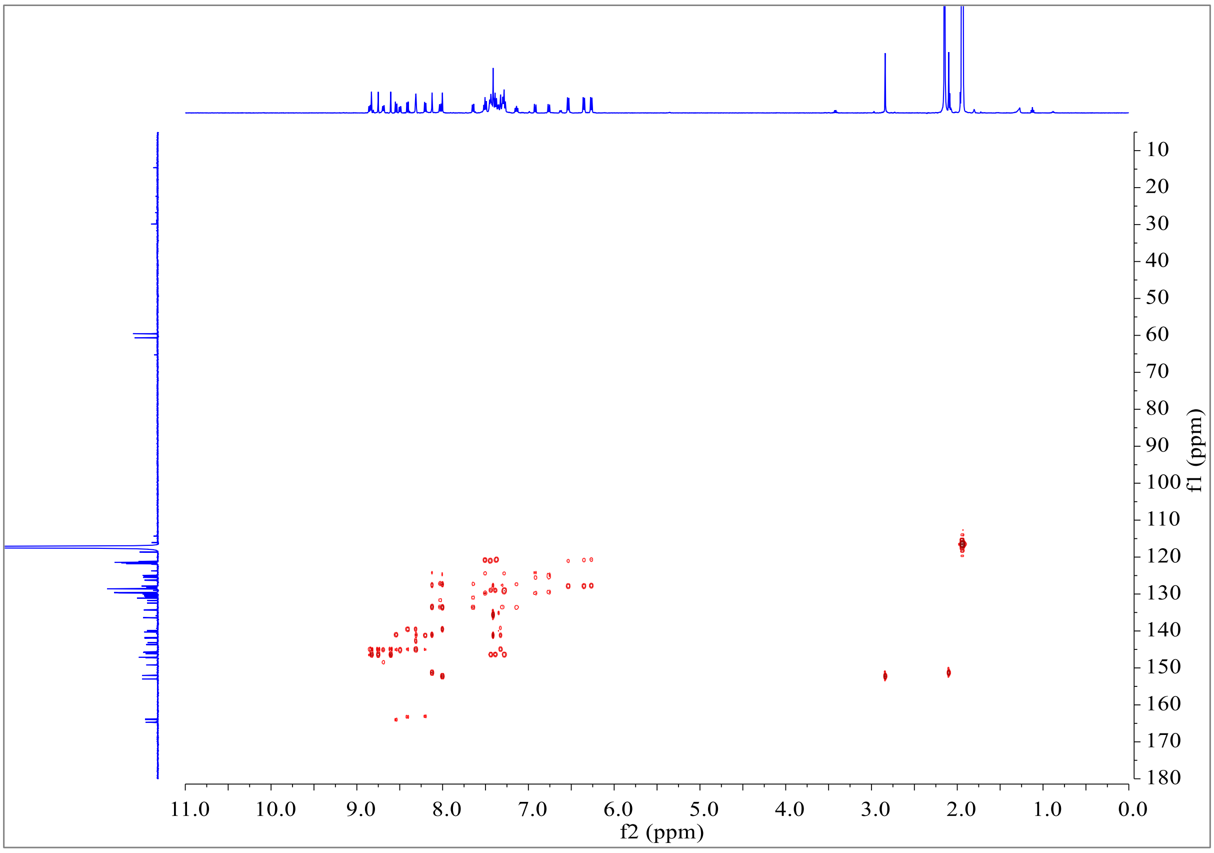

Figure S116. $^{1}H\{^{13}C\}$ HMBC NMR spectrum of *ΔΔ*-Zn-**7** (500 MHz, CD_3CN, 298 K).

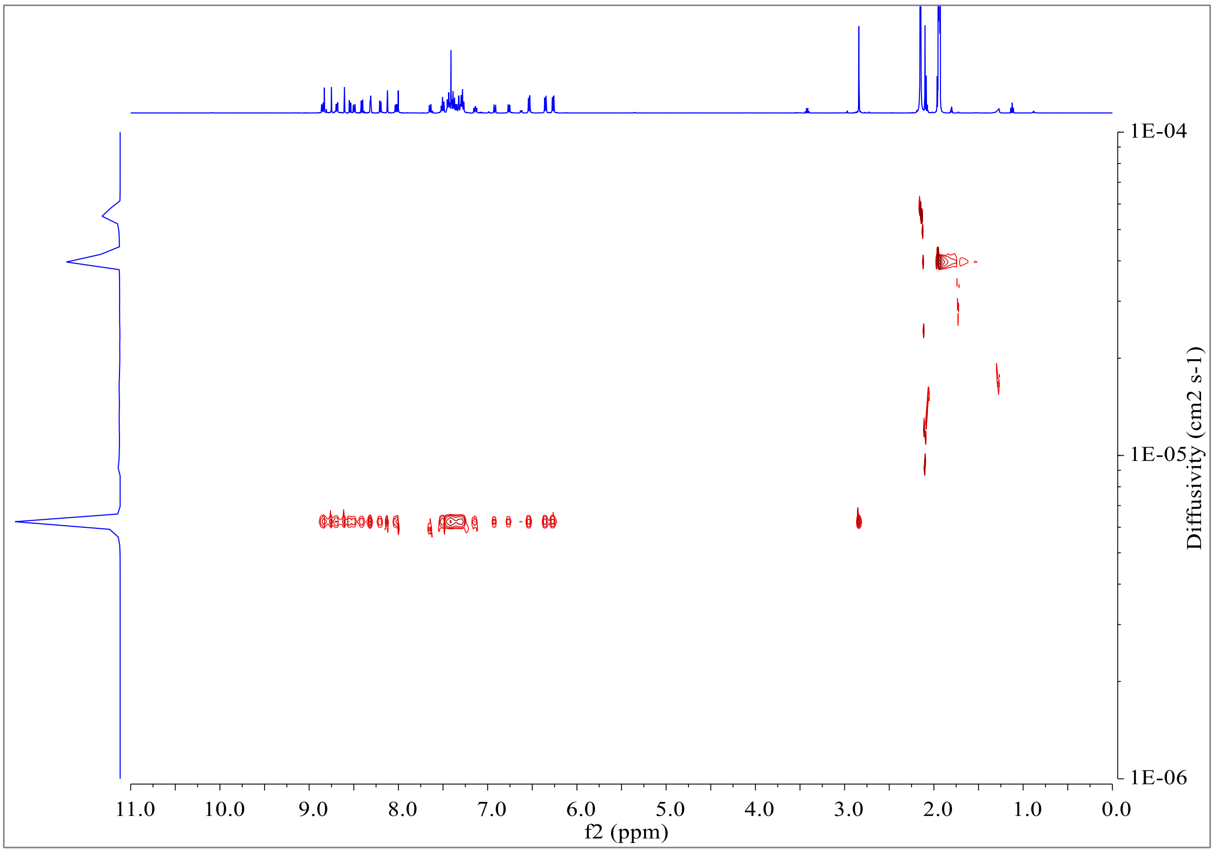

Figure S117. ^{1}H DOSY NMR spectrum of *ΔΔ*-Zn-**7** (400 MHz, CD_3CN, 298 K).

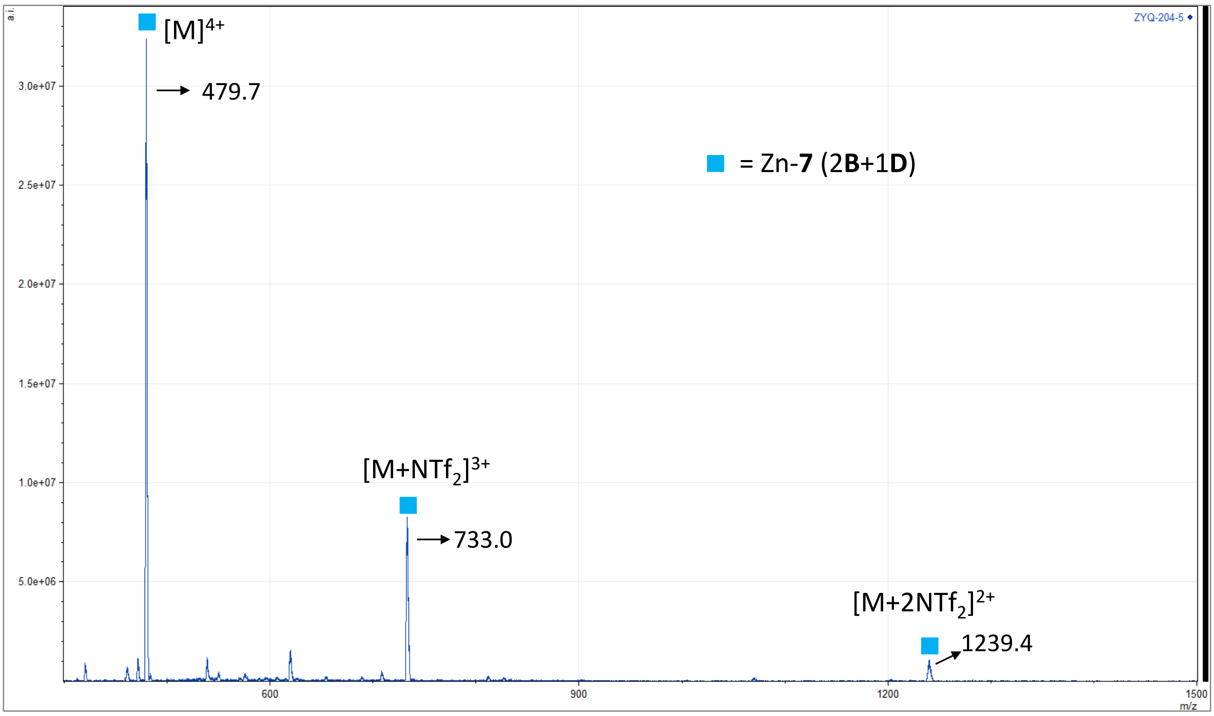

Figure S118. Low-resolution ESI-mass spectrum of *ΔΔ*-Zn-**7**.

5. X-ray crystallography

Data were collected at Beamline I19 of Diamond Light Source employing silicon double crystal monochromated synchrotron radiation (0.6889 Å) with ω and ψ scans at 100(2) K.[6] In all cases the crystals were flash cooled in liquid nitrogen prior to data collection. Data integration and reduction were undertaken with Xia2.[7] Subsequent computations were carried out using the WinGX-32 graphical user interface.[8] Multi-scan empirical absorption corrections were applied to the data using the AIMLESS[9] tool in the CCP4 suite.[10] The structures were solved by intrinsic phasing using SHELXT[11] then refined and extended with SHELXL.[12] In general, non-hydrogen atoms with occupancies greater than 0.5 were refined anisotropically. Carbon-bound hydrogen atoms were included in idealised positions and refined using a riding model. Oxygen-bound hydrogen atoms were first located in the difference Fourier map before refinement. Disorder was modelled using standard crystallographic methods including constraints, restraints and rigid bodies where necessary. Crystallographic data along with specific details pertaining to the refinement follow. Crystallographic data have been deposited with the CCDC (2060201-2060205).

[*ΛΛ*-Zn-1]·4NTf$_2$ [+ solvent]

Formula $C_{140}H_{90}F_{24}N_{16}O_{22}S_8Zn_2$, *M* 3191.49, Tetragonal, space group $P4_32_12$ (#96), *a* 15.00690(5), *b* 15.00690(5), *c* 64.9038(3) Å, *V* 14616.79(12) Å^3, D_c 1.450 g cm^{-3}, *Z* 4, crystal size 0.030 by 0.020 by 0.015 mm, colour yellow, habit block, temperature 100(2) Kelvin, *l*(Synchrotron) 0.6889 Å, *m*(Synchrotron) 0.498 mm^{-1}, *T*(Analytical)$_{min,max}$ 0.9881324151087472, 1.0, 2q_{max} 53.14, *hkl* range -19 17, -18 19, -83 77, *N* 107463, N_{ind} 16708(R_{merge} 0.0452), N_{obs} 11101(I > 2s(I)), N_{var} 1231, residuals* *R*1(*F*) 0.0761, *wR*2(F^2) 0.2527, GoF(all) 1.019, D$r_{min,max}$ -0.522, 0.531 e$^-$ Å^{-3}.

*$R1 = S||F_o| - |F_c||/S|F_o|$ for $F_o > 2s(F_o)$; $wR2 = (Sw(F_o^2 - F_c^2)^2/S(wF_c^2)^2)^{1/2}$ all reflections

$w=1/[s^2(F_o^2)+(0.1900P)^2]$ where $P=(F_o^2+2F_c^2)/3$

Specific refinement details:

The crystals of [*ΛΛ*-Zn-**1**]·4NTf$_2$ [+ solvent] were grown by diffusion of diethyl ether into an acetontrile solution of the complex. The asymmetric unit was found to contain half of a Zn_2L_3 assembly and associated counterions. Due to significant thermal motion within the structure bond lengths and angles within pairs of chemically identical organic ligands arms were restrained to be

similar to each other (SAME) and thermal parameter restraints (SIMU, RIGU) were applied to all atoms except for zinc. One complete naphthol unit and one of the other hydroxyl groups were modelled as disordered over two orientations. Although the hydrogen atoms of the hydroxyl groups were located in the electron density map prior to refinement, the certainty in their positions is lowered by the disorder in two of these groups.

The anions within the structure show evidence of substantial disorder. Both triflimide anions were modelled as disordered over two or three locations with substantial bond length and thermal parameter restraints were applied to facilitate a reasonable refinement. The occupancies of all located anions were freely refined and low occupancy disordered positions were modelled with isotropic thermal parameters. Further minor occupancy positions of the disordered anions could not be located in the electron density map and are not included in the model.

Further reflecting the solvent loss there is a significant amount of void volume in the lattice containing smeared electron density from disordered solvent and 1.3 anions per Zn_2L_3 assembly. Consequently the SQUEEZE[13] function of PLATON[14] was employed to remove the contribution of the electron density associated with these remaining anions and further highly disordered solvent, which gave a potential solvent accessible void of 1941 Å^3 per unit cell (a total of approximately 406 electrons). The remaining anions are assigned as triflimide in the formula. The diffuse solvent molecules could not be assigned to acetonitrile or diethyl ether and are not included in the formula. Consequently, the molecular weight and density given above are underestimated.

The absolute configuration of the structure was confirmed using anomalous dispersion effects with the Flack parameter[15] refining to 0.034(5).

[ΛΛ-Co-1]·4NTf$_2$ [+ solvent]

Formula $C_{280}H_{180}Co_4F_{48}N_{32}O_{44}S_{16}$, *M* 6357.23, Tetragonal, space group P4_32_12 (#96), *a* 15.02960(5), *b* 15.02960(5), *c* 64.7226(3) Å, *V* 14620.12(12) Å^3, D_c 1.444 g cm^{-3}, *Z* 2, crystal size 0.035 by 0.025 by 0.010 mm, colour orange, habit block, temperature 100(2) Kelvin, *l*(Synchrotron) 0.6889 Å, *m*(Synchrotron) 0.404 mm^{-1}, T(Analytical)$_{min,max}$ 0.979083488092948, 1.0, 2q_{max} 53.14, *hkl* range -19 17, -19 19, -71 83, *N* 155017, N_{ind} 16798(R_{merge} 0.0464), N_{obs} 11057(I > 2s(I)), N_{var} 1052, residuals* $R1(F)$ 0.1044, $wR2(F^2)$ 0.3211, GoF(all) 1.256, D$r_{min,max}$ -0.730, 0.691 e$^-$ Å^{-3}.

*$R1 = S||F_o| - |F_c||/S|F_o|$ for $F_o > 2s(F_o)$; $wR2 = (Sw(F_o^2 - F_c^2)^2/S(wF_c^2)^2)^{1/2}$ all reflections

$w=1/[s^2(F_o^2)+(0.2000P)^2]$ where $P=(F_o^2+2F_c^2)/3$

Specific refinement details:

The crystals of [ΛΛ-Co-**1**]·4NTf$_2$ [+ solvent] were grown by diffusion of diethyl ether into an acetontrile solution of the complex. The asymmetric unit was found to contain half of a Co_2L_3 assembly and associated counterions. The structure is basically isomorphous to that of [ΛΛ-Zn-**1**]·4NTf$_2$ apart from some minor details related to disorder.

Due to significant thermal motion within the structure bond lengths and angles within pairs of chemically identical organic ligands arms were restrained to be similar to each other (SAME) and thermal parameter restraints (SIMU, RIGU) were applied to all atoms except for cobalt. One hydroxyl group was modelled as disordered over two orientations. There is evidence that the naphthalene ring to which it is attached is also disordered but no reasonable model for this disorder could be found generating one A level Checkcif alert for the unusual C-O-H angle of the minor occupancy oxygen. Although the hydrogen atoms of the hydroxyl groups were located in the electron density map prior to refinement, the certainty in their positions is lowered by the disorder in one of these groups.

The anions within the structure show evidence of substantial disorder. Both triflimide anions were modelled as disordered over two locations with substantial bond length and thermal parameter restraints applied to facilitate a reasonable refinement. The occupancies of all located anions were freely refined and low occupancy disordered positions were modelled with isotropic thermal parameters. Further minor occupancy positions of the disordered anions could not be located in the electron density map and are not included in the model.

Further reflecting the solvent loss and poor diffraction properties there is a significant amount of void volume in the lattice containing smeared electron density from disordered solvent and 1.6 anions per Co_2L_3 assembly. Consequently the SQUEEZE[13] function of PLATON[14] was employed to remove the contribution of the electron density associated with these remaining anions and further highly disordered solvent, which gave a potential solvent accessible void of 2205 Å^3 per unit cell (a total of approximately 641 electrons). The remaining anions are assigned as triflimide in the formula. The diffuse solvent molecules could not be assigned to acetonitrile or diethyl ether and are not included in the formula. Consequently, the molecular weight and density given above are underestimated.

The absolute configuration of the structure was confirmed using anomalous dispersion effects with the Flack parameter[15] refining to 0.066(6). The slight deviation from zero can be explained by the disorder and thermal motion within the structure.

[ΛΔ-Co-2]·4NTf$_2$·2Et$_2$O·4.5MeCN

Formula $C_{163}H_{135.50}Co_2F_{24}N_{20.50}O_{24}S_8$, *M* 3595.75, Orthorhombic, space group P2$_1$2$_1$2$_1$ (#19), *a* 14.69770(10), *b* 25.2307(2), *c* 44.8058(3) Å, *V* 16615.5(2) Å^3, D_c 1.437 g cm^{-3}, *Z* 4, crystal size 0.200 by 0.150 by 0.100 mm, colour orange, habit block, temperature 100(2) Kelvin, *l*(Synchrotron) 0.6889 Å, *m*(Synchrotron) 0.365 mm^{-1}, T(Analytical)$_{min,max}$ 0.9527951291361857, 1.0, 2q_{max} 70.07, *hkl* range -24 24, -41 41, -72 74, *N* 344609, N_{ind} 78492(R_{merge} 0.0424), N_{obs} 67315(I > 2s(I)), N_{var} 2221, residuals* *R*1(*F*) 0.0435, *wR*2(F^2) 0.1113, GoF(all) 1.015, D$r_{min,max}$ -1.199, 0.813 e$^-$ Å^{-3}.

*$R1 = S||F_o| - |F_c||/S|F_o|$ for $F_o > 2s(F_o)$; $wR2 = (Sw(F_o^2 - F_c^2)^2/S(wF_c^2)^2)^{1/2}$ all reflections

$w=1/[s^2(F_o^2)+(0.0540P)^2+5.6076P]$ where $P=(F_o^2+2F_c^2)/3$

Specific refinement details:

The crystals of [*ΛΔ*-Co-**2**]·4NTf$_2$·2Et$_2$O·4.5MeCN were grown by diffusion of diethyl ether into an acetonitrile solution of the complex. The asymmetric unit was found to contain one complete Co$_2$L$_3$ assembly and associated counterions and solvent molecules. The solvent molecules within the structure show evidence of substantial disorder. Both diethyl ether molecules were modelled as disordered over three locations with substantial bond length and thermal parameter restraints applied to facilitate a reasonable refinement. One acetonitrile was also modelled as partially disordered and a further one with partial occupancy.

The absolute configuration of the structure was confirmed using anomalous dispersion effects with the Flack parameter[15] refining to 0.0217(18).

[ΛΛ-Fe-4]·4NTf$_2$·Et$_2$O·0.5MeCN

Formula $C_{129}H_{97.50}F_{24}Fe_2N_{16.50}O_{21}S_8$, *M* 3038.91, Orthorhombic, space group P 21 21 2 (#18), *a* 23.55760(9), *b* 33.49870(15), *c* 17.75470(7) Å, *V* 14011.10(10) Å^3, D_c 1.441 g cm^{-3}, *Z* 4, crystal size 0.050 by 0.030 by 0.020 mm, colour purple, habit block, temperature 100(2) Kelvin, *l*(Synchrotron) 0.6889 Å, *m*(Synchrotron) 0.390 mm^{-1}, T(Analytical)$_{min,max}$ 0.9939085952900435, 1.0, 2q_{max} 64.00, *hkl* range -36 29, -49 51, -27 27, *N* 199641, N_{ind} 53079(R_{merge} 0.0524), N_{obs} 26036(I > 2s(I)), N_{var} 2000, residuals* *R*1(*F*) 0.0561, *wR*2(F^2) 0.1482, GoF(all) 0.840, D$r_{min,max}$ -0.597, 0.822 e$^-$ Å^{-3}.

$^{*}R1 = S||F_o| - |F_c||/S|F_o|$ for $F_o > 2s(F_o)$; $wR2 = (Sw(F_o^2 - F_c^2)^2/S(wF_c^2)^2)^{1/2}$ all reflections

$w=1/[s^2(F_o^2)+(0.0809P)^2]$ where $P=(F_o^2+2F_c^2)/3$

Specific refinement details:

The crystals of [*ΛΛ*-Fe-**4**]·4NTf$_2$·Et$_2$O·0.5MeCN were grown by diffusion of diethyl ether into an acetonitrile solution of the complex. The asymmetric unit was found to contain one complete Fe$_2$L$_2$L′ assembly and associated counterions and solvent molecules. Bond lengths and angles within the two chemically identical organic ligands were restrained to be similar to each other (SAME) and thermal parameter restraints (SIMU, RIGU) were applied to all atoms except for iron. The anions and solvents within the structure show evidence of substantial disorder. Two triflimide anions were modelled as disordered over two locations (one of these further disordered over a special position) while a further triflimide was disordered over a special position. Overall the four anions are disordered over five lattice sites, two of which are located on special positions with 50% occupancy. All solvent molecules were also modelled as disordered over two locations. Substantial bond length and thermal parameter restraints applied to facilitate a reasonable refinement of the disordered groups and low occupancy disordered moieties were modelled with isotropic thermal parameters. The hydrogen atoms of the disordered acetonitrile molecule could not be located in the electron density map and were therefore not included in the model.

There is a significant amount of void volume in the lattice containing smeared electron density from further highly disordered solvent molecules. Consequently the SQUEEZE[13] function of PLATON[14] was employed to remove the contribution of the electron density associated with these remaining anions and further highly disordered solvent, which gave a potential solvent accessible void of 1079 Å^3 per unit cell (a total of approximately 313 electrons). The diffuse solvent molecules could not be assigned to acetonitrile or diethyl ether and are not included in the formula. Consequently, the molecular weight and density given above are underestimated.

The absolute configuration of the structure was confirmed using anomalous dispersion effects with the Flack parameter[15] refining to 0.044(5). The slight deviation from zero can be explained by the disorder and thermal motion within the structure.

[*ΛΛ*-Zn-4]·4NTf$_2$·2Et$_2$O

Formula $C_{132}H_{106}F_{24}N_{16}O_{22}S_8Zn_2$, *M* 3111.54, Monoclinic, space group P 21 (#4), *a* 13.30080(14), *b* 29.7376(3), *c* 18.3356(2) Å, *b* 107.0580(10), *V* 6933.31(14) Å^3, D_c 1.490 g cm^{-3}, *Z* 2, crystal size 0.060 by 0.040 by 0.030 mm, colour yellow, habit block, temperature 100(2) Kelvin, *l*(synchrotron) 0.6889 Å, *m*(synchrotron) 0.523 mm^{-1}, T(Analytical)$_{min,max}$ 0.9775682631460881,

1.0, $2q_{max}$ 64.00, *hkl* range -20 20, -45 45, -28 28, *N* 131013, N_{ind} 51638(R_{merge} 0.0551), N_{obs} 32555(I > 2s(I)), N_{var} 2107, residuals* $R1(F)$ 0.0546, $wR2(F^2)$ 0.1455, GoF(all) 0.946, $Dr_{min,max}$ -0.677, 0.850 e^- $Å^{-3}$.

*$R1 = S||F_o| - |F_c||/S|F_o|$ for $F_o > 2s(F_o)$; $wR2 = (Sw(F_o^2 - F_c^2)^2/S(wF_c^2)^2)^{1/2}$ all reflections

$w=1/[s^2(F_o^2)+(0.0753P)^2]$ where $P=(F_o^2+2F_c^2)/3$

Specific refinement details:

The crystals of [*ΛΛ*-Zn-**4**]·4NTf₂·2Et₂O were grown by diffusion of diethyl ether into an acetonitrile solution of the complex. The asymmetric unit was found to contain one complete Zn_2L_2L' assembly and associated counterions and solvent molecules. Bond lengths and angles within the two chemically identical organic ligands were restrained to be similar to each other (SAME) and thermal parameter restraints (SIMU, RIGU) were applied to all atoms except for zinc. Two aniline residues were modelled as disordered over two orientations and were modelled as rigid groups (AFIX 66). The anions and solvents within the structure show evidence of substantial disorder. All triflimide anions and diethyl ether molecules were modelled as disordered over two or three locations with substantial bond length and thermal parameter restraints applied to facilitate a reasonable refinement. Low occupancy disordered moieties were modelled with isotropic thermal parameters.

The absolute configuration of the structure was confirmed using anomalous dispersion effects with the Flack parameter[15] refining to 0.000(3).

The symmetry elements of assemblies **Zn-1** and **Co-2** are shown as follows:

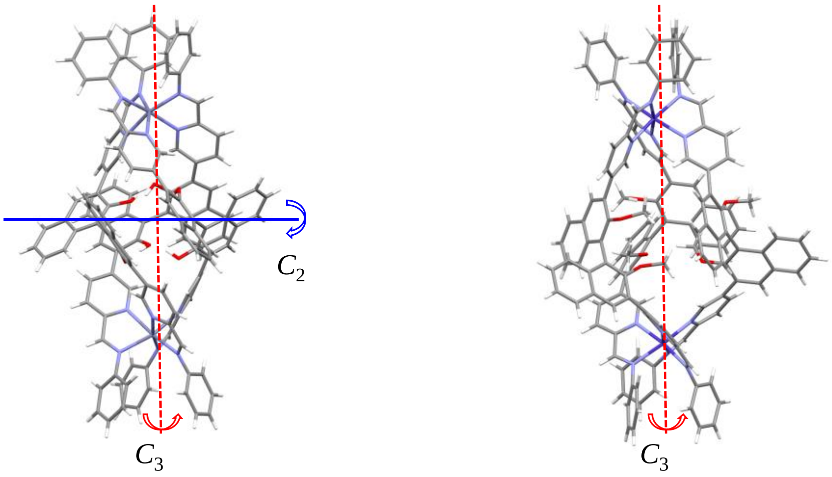

ΛΛ-Zn-**1** (*D_3*) *ΛΔ*-Co-**2** (*C_3*)

Figure S119. The symmetry elements of assemblies **Zn-1** and **Co-2**.

6. Density functional theory calculations

6.1 General

All density functional theory (DFT) calculations were performed in the ORCA 4.2.1 software.[16] Geometry optimisations were performed with the PBE0 functional,[17] the Ahlrichs def2-SVP basis set[18] and the dispersion correction based on tight binding partial charges (D4);[19] this method is termed PBE0/def2-SVP/D4. Subsequent single-point energy calculations (SPE) were performed using an equivalent method with the def2-TZVP basis set[18a] and D4 correction (termed PBE0/def2-TZVP/D4).

6.2 Structure generation and optimisation

All structures underwent the same process: 1) optimisation with GFN2-xTB, 2) optimisation with PBE0/def2-SVP/D4 and 3) SPE calculation with PBE0/def2-TZVP/D4. The xTB methods[20] are tight-binding quantum chemical methods for the geometry optimisation of systems containing elements up to Z = 82; these methods are a robust and cheap alternative to DFT for metal-containing species.[21] Version 6.4.0 was used throughout. Table 1 shows the origin of all structures that underwent this process. For *ΛΔ*-Zn-**1** a Conformer-Rotamer Ensemble Sampling Tool (CREST) conformer search[22] was performed prior to step 1 using default settings and version 2.10.1. All systems are treated as low spin, closed shell with a charge of +4 (+2 from each of the Zn(**II**) or Fe(**II**) metal centres). Figure S120 shows the DFT-optimised structures compared to x-ray structures for *ΛΛ*-Fe-**4** and *ΛΛ*-Zn-**1**, where DFT optimisation does not lead to significant structural change. Figure S121 shows all other DFT-optimised structures (xyz files available at the github link below). All images were generated with academic pymol (the PyMOL molecular graphics system, version 1.8). All input files and output files and structures are freely available at github.com/andrewtarzia/citable_data/tree/master/zou_2021.

Table S1: Origin of structures studied using DFT methods.

structure	origin
ΛΛ-Fe-**4**	x-ray structure
ΔΔ-Fe-**4**	structure modified from *ΔΔ*-Fe-**7**
ΛΛ-Fe-**6**	structure modified from *ΛΛ*-Fe-**7**
ΔΔ-Fe-**6**	PM3 model
ΛΛ-Fe-**7**	PM3 model
ΔΔ-Fe-**7**	PM3 model
ΛΛ-Zn-**1**	x-ray structure
ΛΔ-Zn-**1**	structure modified from *ΛΔ*-Zn-**2**
ΛΛ-Zn-**2**	structure modified from *ΛΛ*-Zn-**1**
ΛΔ-Zn-**2**	structure modified from x-ray structure of *ΛΔ*-Co-**2**

Table S2: Relative energies of structure pairs after step 1 and 3. All energies are reported from SPE calculations at the respective level of theory in kJmol^{-1}.

structure	GFN2-xTB	PBE0/def2-TZVP/D4
ΛΛ-Fe-**4**	0.0	0.0
ΔΔ-Fe-**4**	35.4	47.6
ΛΛ-Fe-**6**	48.5	63.8
ΔΔ-Fe-**6**	0.0	0.0
ΛΛ-Fe-**7**	10.9	16.3
ΔΔ-Fe-**7**	0.0	0.0
ΛΛ-Zn-**1**	0.0	0.0
ΛΔ-Zn-**1**	4.7	8.7
ΛΛ-Zn-**2**	17.2	20.4
ΛΔ-Zn-**2**	0.0	0.0

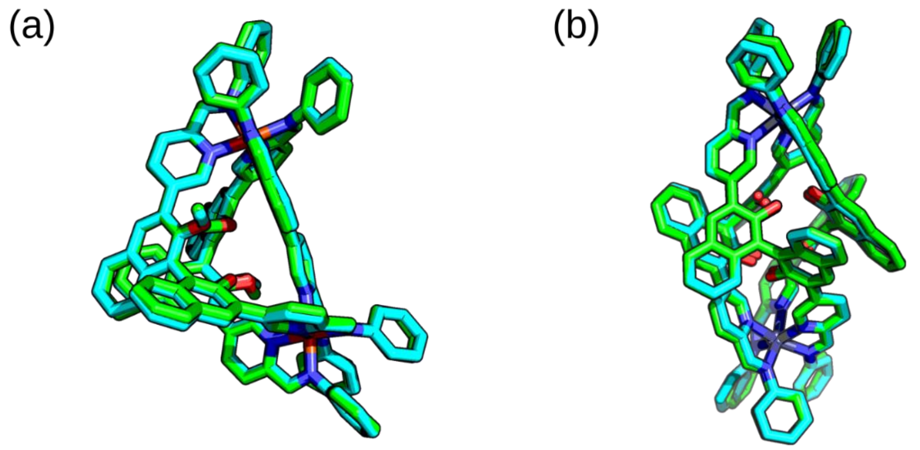

Figure S120. Comparison of x-ray (carbon: green) and DFT-optimised (carbon: cyan) structures of (a) *ΛΛ*-Fe-**4** and (b) *ΛΛ*-Zn-**1** (nitrogen: blue, oxygen: red, iron: orange, zinc: gray, hydrogen atoms omitted for clarity).

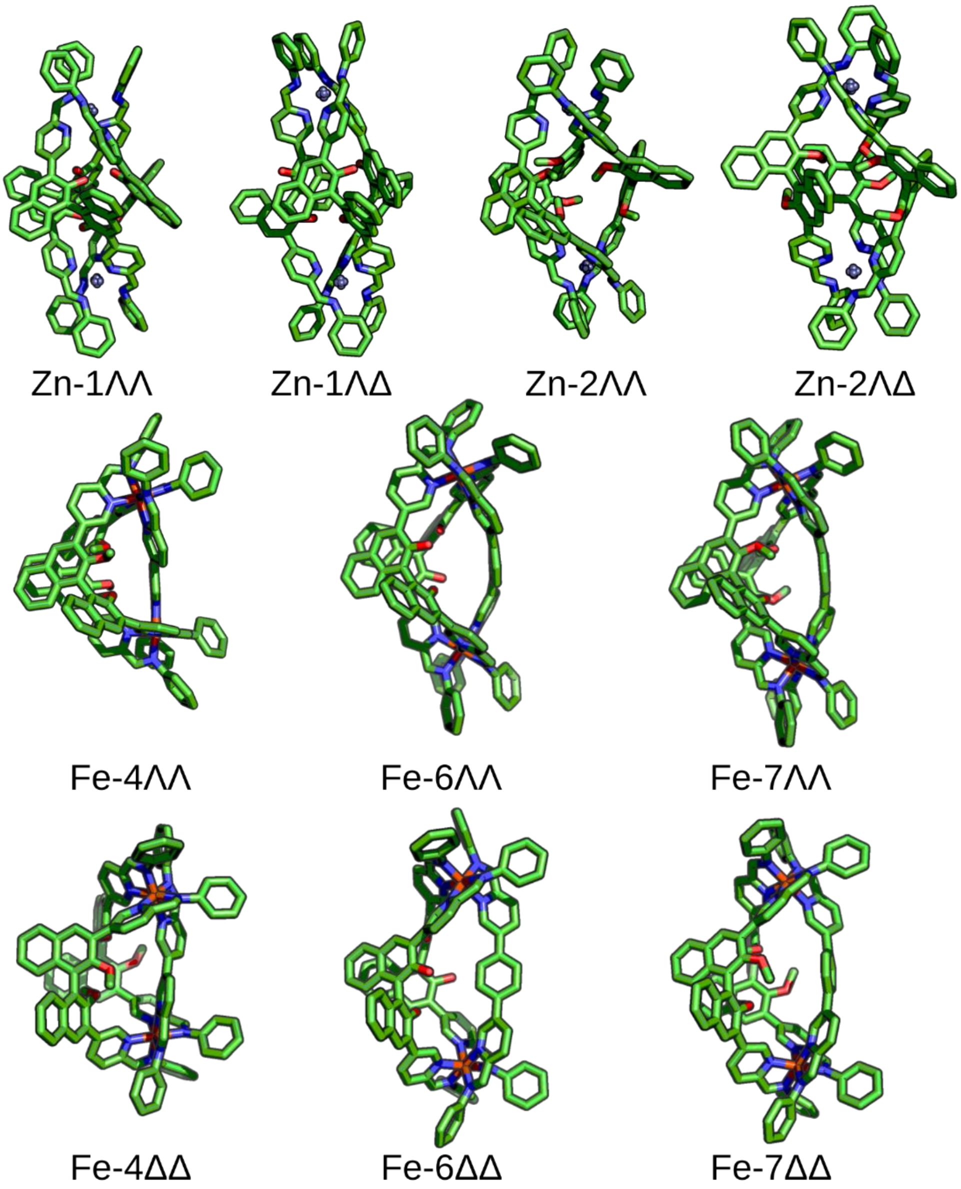

Figure S121. DFT optimised structures of all Zn and Fe assemblies. (carbon: green, nitrogen: blue, oxygen: red, iron: orange, zinc: gray, hydrogen atoms omitted for clarity).

6.3 Strain in heteroleptic helicates

We calculated the ligand strain energy for the heteroleptic helicates (Table S3) as $E_{strain} = E_{extracted} - E_{free}$, where $E_{extracted}$ is the energy of the ligand extracted from the DFT-optimised structure and E_{free} is the energy of the lowest energy conformer of that ligand (both energies are calculated at the PBE0/def2-TZVP/D4 level of theory). The strain energy is calculated with the "strain_energy_dft.py" script available at github.com/andrewtarzia/citable_data/tree/master/zou_2021. The lowest energy conformer of each ligand is calculated using the CREST software (version 2.10.1 with default settings) and the GFN2-xTB method. Each lowest energy conformer is then DFT optimised at the PBE0/def2-SVP/D4 level of theory.

Table S3: Ligand strain energies of BINOL-based ligands (**A** and **B**), linear ligands (**C** and **D**) and their sums. All energies are given in $kJmol^{-1}$.

structure	ligand **A**/**B**	ligand **C**/**D**	total
ΛΛ-Fe-**4**	92.2, 87.9	75.3	255.4
ΔΔ-Fe-**4**	91.2, 90.2	76.8	258.1
ΛΛ-Fe-**6**	125.9, 100.6	94.6	321.0
ΔΔ-Fe-**6**	93.5, 93.6	83.1	270.2
ΛΛ-Fe-**7**	102.6, 102.4	94.2	299.2
ΔΔ-Fe-**7**	89.1, 89.6	83.2	261.8

7. References

(1) Gottlieb, H. E.; Kotlyar, V.; Nudelman, A. NMR Chemical Shifts of Common Laboratory Solvents as Trace Impurities. *J. Org. Chem.* **1997**, *62*, 7512–7515.

(2) Simonsen, K. B.; Gothelf, K. V.; Jϕrgensen, K. A. A Simple Synthetic Approach to 3,3'-Diaryl BINOLs. *J. Org. Chem.* **1998**, *63*, 7536–7538.

(3) Pecho, F.; Zou, Y.-Q.; Gramüller, J.; Mori, T.; Huber, S. M.; Bauer, A.; Gschwind, R. M.; Bach, T. A Thioxanthone Sensitizer with a Chiral Phosphoric Acid Binding Site: Properties and Applications in Visible Light - Mediated Cycloadditions. *Chem. Eur. J.* **2020**, *26*, 5190-5194.

(4) Ojida, A.; Sakamoto, T.; Inoue, M.-a.; Fujishima, S.-h.; Lippens, G.; Haachi, T. Fluorescent BODIPY-Based Zn(II) Complex as a Molecular Probe for Selective Detection of Neurofibrillary Tangles in the Brains of Alzheimer's Disease Patients. *J. Am. Chem. Soc.* **2009**, *131*, 6543-6548.

(5) Ma, S.; Smulders, M. M. J.; Hristova, Y. R.; Clegg, J. K.; Ronson, T. K.; Zarra, S.; Nitschke, J. R. Chain-Reaction Anion Exchange between Metal–Organic Cages. *J. Am. Chem. Soc.* **2013**, *135*, 5678-5684.

(6) Allan, D.; Nowell, H.; Barnett, S.; Warren, M.; Wilcox, A.; Christensen, J.; Saunders, L.; Peach, A.; Hooper, M.; Zaja, L.; Patel, S.; Cahill, L.; Marshall, R.; Trimnell, S.; Foster, A.; Bates, T.; Lay, S.; Williams, M.; Hathaway, P.; Winter, G.; Gerstel, M.; Wooley, R. A Novel Dual Air-Bearing Fixed-χ Diffractometer for Small-Molecule Single-Crystal X-ray Diffraction on Beamline I19 at Diamond Light Source. *Crystals* **2017,** *7*, 336.

(7) (a) Collaborative Computational Project, N. The CCP4 Suite: Programs for Protein Crystallography. *Acta Cryst.* **1994,** *D50*, 760-763; (b) Evans, P., Scaling and Assessment of Aata Quality. *Acta Cryst.* **2006,** *D62*, 72-82; (c) Winter, G., xia2: an Expert Sstem for Macromolecular Crystallography Data Reduction. *J. Appl. Crystallogr.* **2010,** *43*, 186-190.

(8) Farrugia, L., WinGX and ORTEP for Windows: an update. *J. Appl. Crystallogr.* **2012,** *45*, 849.

(9) Evans, P. R.; Murshudov, G. N. How Good Are My Data and What Is the Resolution? *Acta Cryst.* **2013,** *D69*, 1204-1214.

(10) Winn, M. D.; Ballard, C. C.; Cowtan, K. D.; Dodson, E. J.; Emsley, P.; Evans, P. R.; Keegan, R. M.; Krissinel, E. B.; Leslie, A. G. W.; McCoy, A.; McNicholas, S. J.; Murshudov, G. N.; Pannu, N. S.; Potterton, E. A.; Powell, H. R.; Read, R. J.; Vagin, A.; Wilson, K. S. Overview of the CCP4 Suite and Current Developments. *Acta Cryst.* **2011,** *D67*, 235-242.

(11) Sheldrick, G. SHELXT - Integrated Space-group and Crystal-structure Determination. *Acta. Cryst.* **2015,** *A71*, 3-8.

(12) Sheldrick, G. M. Crystal Structure Refinement with SHELXL. *Acta. Cryst.* **2015,** *C71*, 3-8.

(13) van der Sluis, P.; Spek, A. L. BYPASS: an Effective Method for the Refinement of Crystal Structures Containing Disordered Solvent Regions. *Acta Cryst.* **1990,** *A46*, 194-201.

(14) Spek, A. L. *PLATON: A Multipurpose Crystallographic Tool*. Utrecht University: Utrecht, The Netherlands, 2008.

(15) Flack, H. D. Chiral and Achiral Crystal Structures. *Helv. Chim. Acta* **2003,** *86*, 905-921.

(16) Neese, F. Software Update: the ORCA Program System, Version 4.0. *WIREs Comput. Mol. Sci.* **2018**, *8*, e1327.

(17) Adamo, C. Toward Reliable Density Functional Methods without Adjustable Parameters: The PBE0 Model. *J. Chem. Phys.* **1999**, *110*, 6158-6169.

(18) (a) Weigend, F.; Ahlrichs, R. Balanced Basis Sets of Split Valence, Triple Zeta Valence and Quadruple Zeta Valence Quality for H to Rn: Design and Assessment of Accuracy. *Phys. Chem. Chem. Phys.* **2005**, *7*, 3297-3305. (b) Weigend, F. Accurate Coulomb-fitting Basis Sets for H to Rn. *Phys. Chem. Chem. Phys.* **2006**, *8*, 1057-1065.

(19) (a) Caldeweyher, E.; Bannwarth, C.; Grimme, S. Extension of the D3 Dispersion Coefficient Model. *J. Chem. Phys.* **2017**, *147*, 034112. (b) Caldeweyher, E.; Ehlert, S.; Hansen, A.; Neugebauer, H.; Spicher, S.; Bannwarth, C.; Grimme, S. A Generally Applicable Atomic-charge Dependent London Dispersion Correction. *J. Chem. Phys.* **2019**, *150*, 154122.

(20) Bannwarth, C.; Ehlert, S.; Grimme, S. GFN2-xTB—An Accurate and Broadly Parametrized Self-Consistent Tight-Binding Quantum Chemical Method with Multipole Electrostatics and Density-Dependent Dispersion Contributions. *J. Chem. Theory Comput.* **2019**, *15*, 1652-1671.

(21) Bursch, M.; Neugebauer, H.; Grimme, S. Structure Optimisation of Large Transition-Metal Complexes with Extended Tight-Binding Methods. *Angew. Chem. Int. Ed.* **2019**, *58*, 11078-11087.

(22) Pracht, P.; Bohle, F.; Grimme, S. Automated Exploration of the Low-energy Chemical Space with Fast Quantum Chemical Methods. *Phys. Chem. Chem. Phys.* **2020**, *22*, 7169-7192.
